# Supplementary material for: Modified MobileNetV2 transfer learning model to detect road potholes
Source: PeerJ Comput Sci. 2025 Jan 21;11:e2519. doi: 10.7717/peerj-cs.2519 (PMC11784875; doi:10.7717/peerj-cs.2519)
Supplement: Supplemental Information 1 [file peerj-cs-11-2519-s001.pdf]

In [7]:
```
import numpy as np # linear algebra
import os # operating system
```

In [8]:
```
 keras.utils import set_random_seed
set_random_seed(812)
from
```

```
WARNING:tensorflow:From C:\ProgramData\anaconda3\Lib\site-packages\keras\src\losse
s.py:2976: The name tf.losses.sparse_softmax_cross_entropy is deprecated. Please u
se tf.compat.v1.losses.sparse_softmax_cross_entropy instead.
```

In [17]:
```
import cv2
import matplotlib.pyplot as plt

def explore_dir(dir_path, count):
    for _, _, filenames in os.walk(dir_path):
        for i in range(count):
            img = cv2.imread(os.path.join(dir_path, filenames[i]))
            plt.imshow(img)
            plt.show()

normal_dir = 'D:/neha1/Normal'
potholes_dir = 'D:/neha1/Pothole'

explore_dir(normal_dir, 100)
explore_dir(potholes_dir, 10)
```

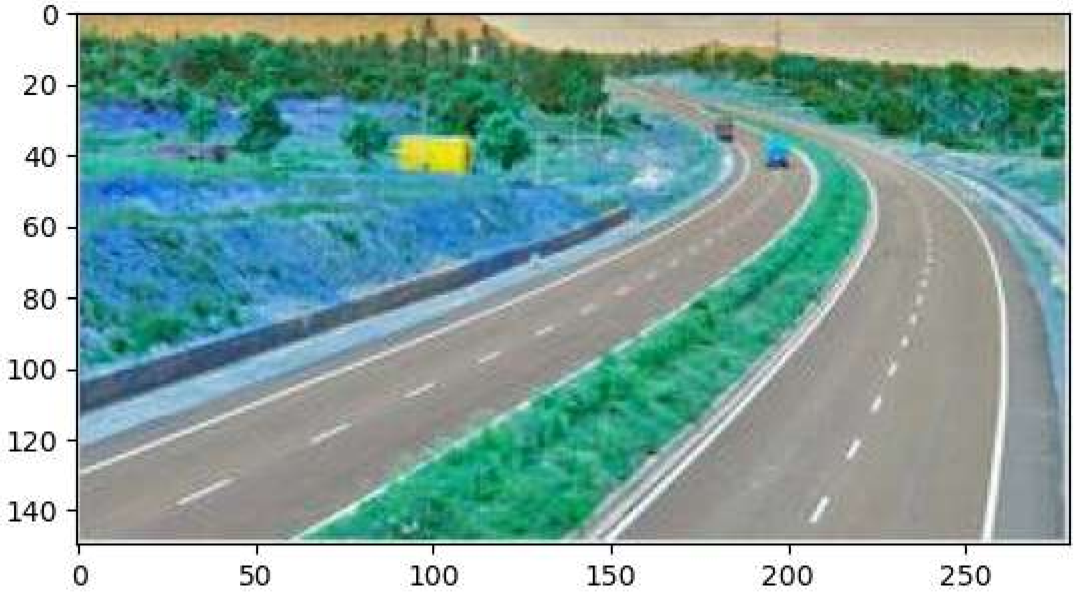

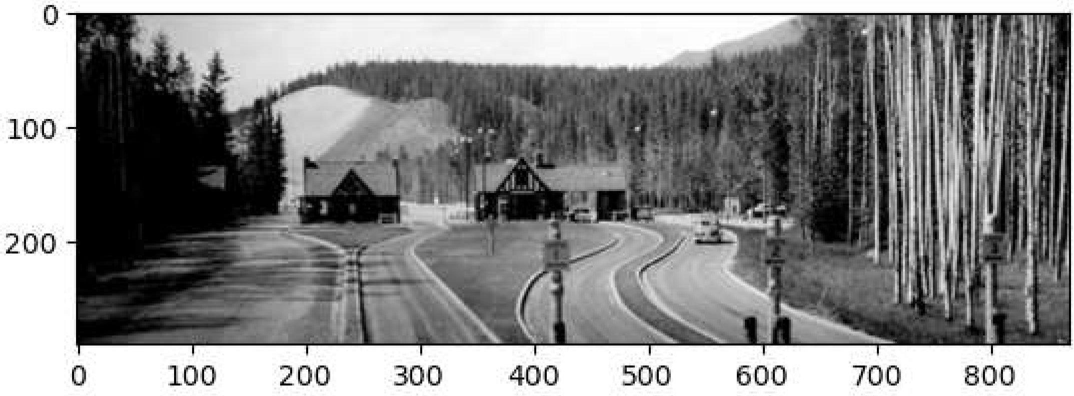

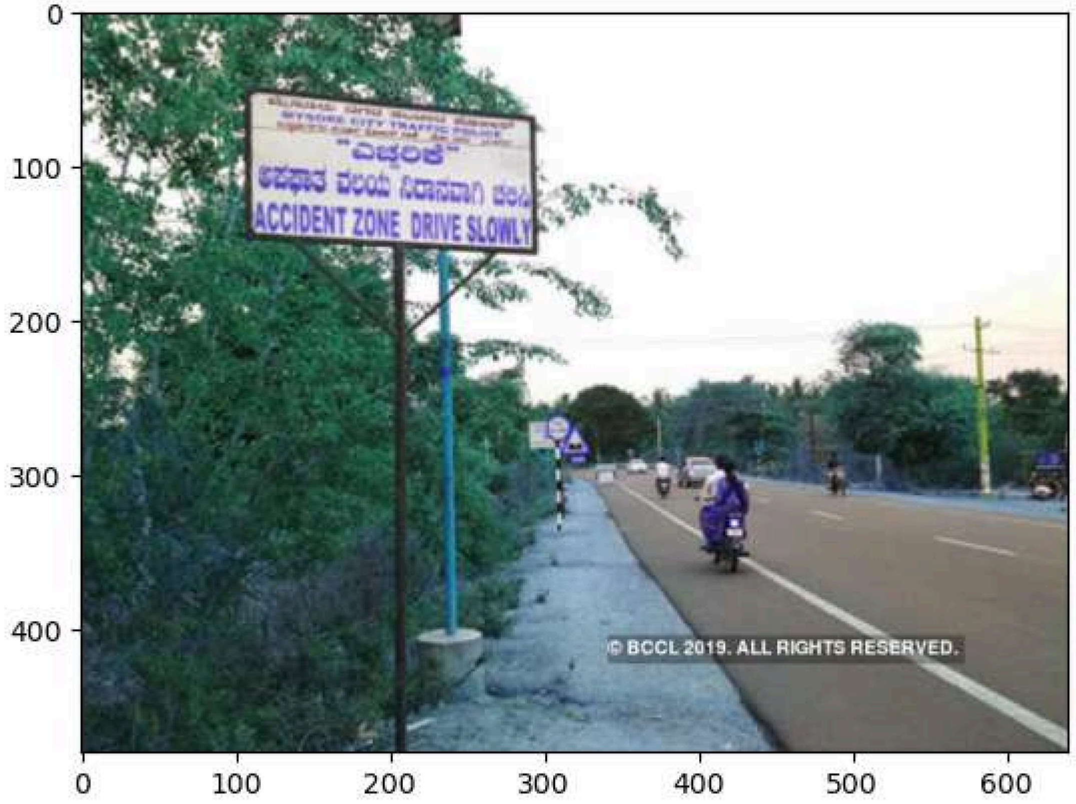
ACCIDENT ZONE DRIVE SLOWLY
© BCCL 2019. ALL RIGHTS RESERVED.
0
100
200
300
400
0
100
200
300
400
500
600

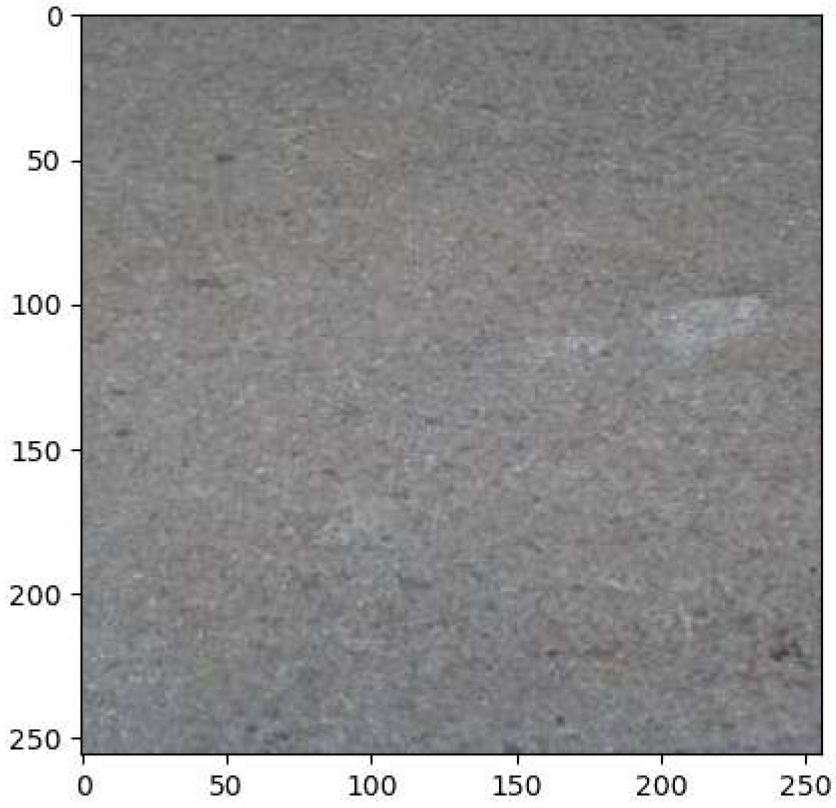
0
50
100
150
200
250
0
50
100
150
200
250

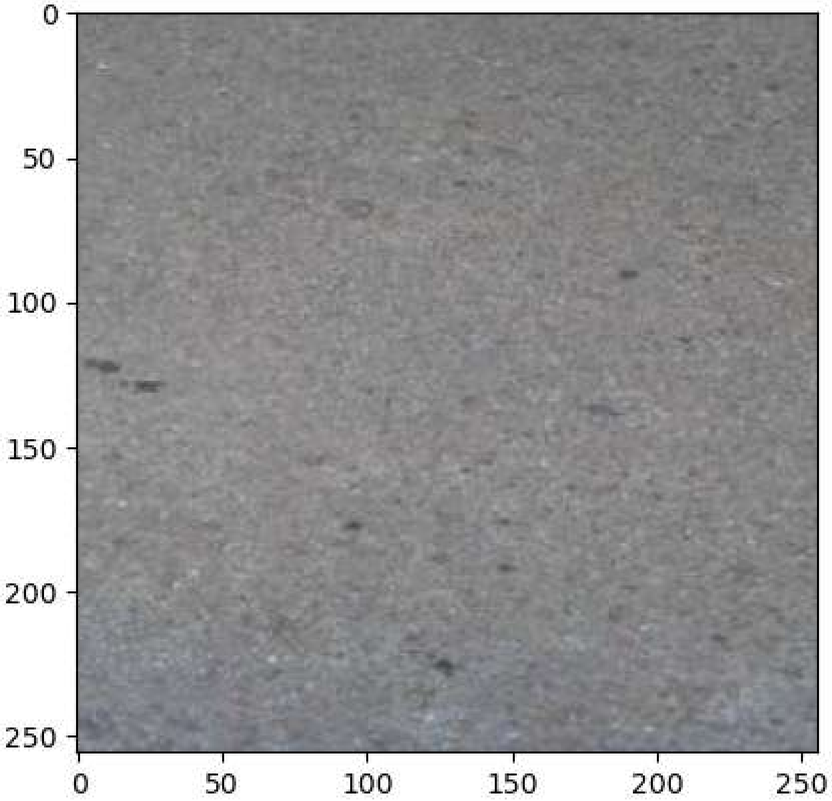

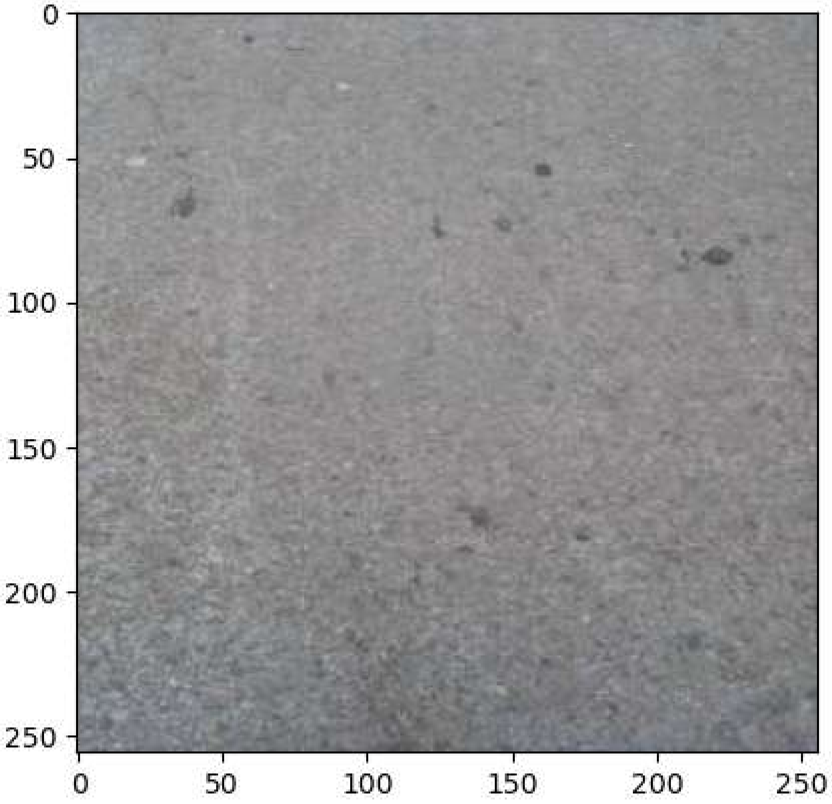

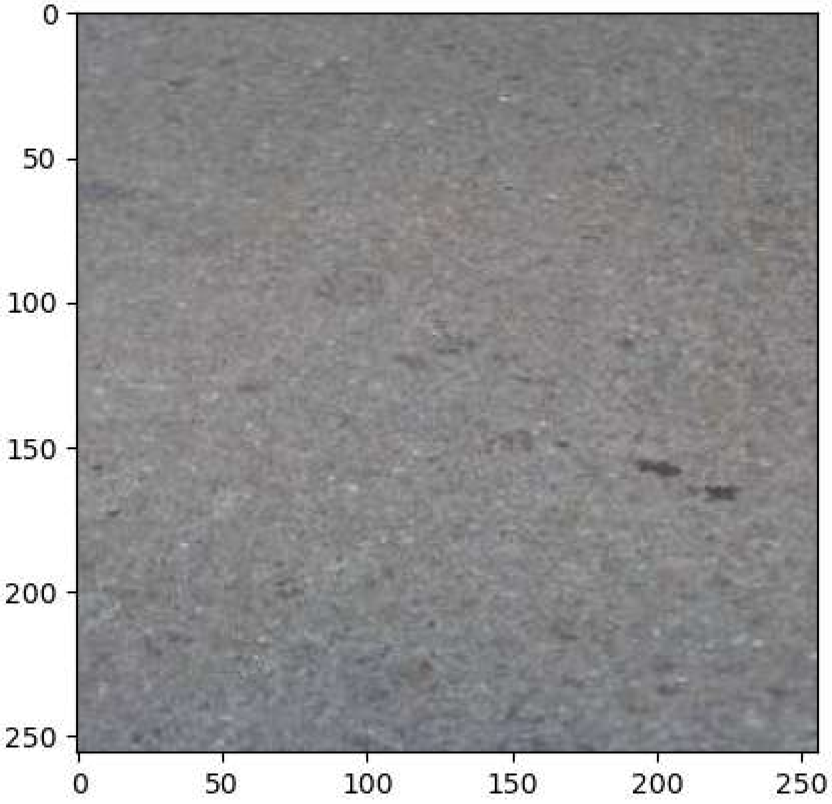
0
50
100
150
200
250

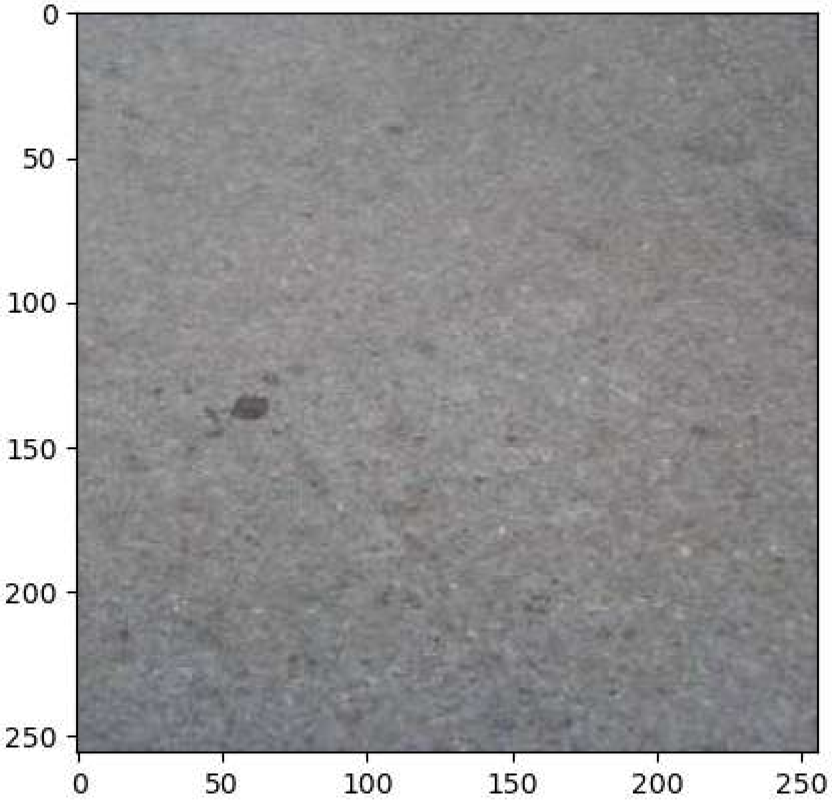
0
50
100
150
200
250

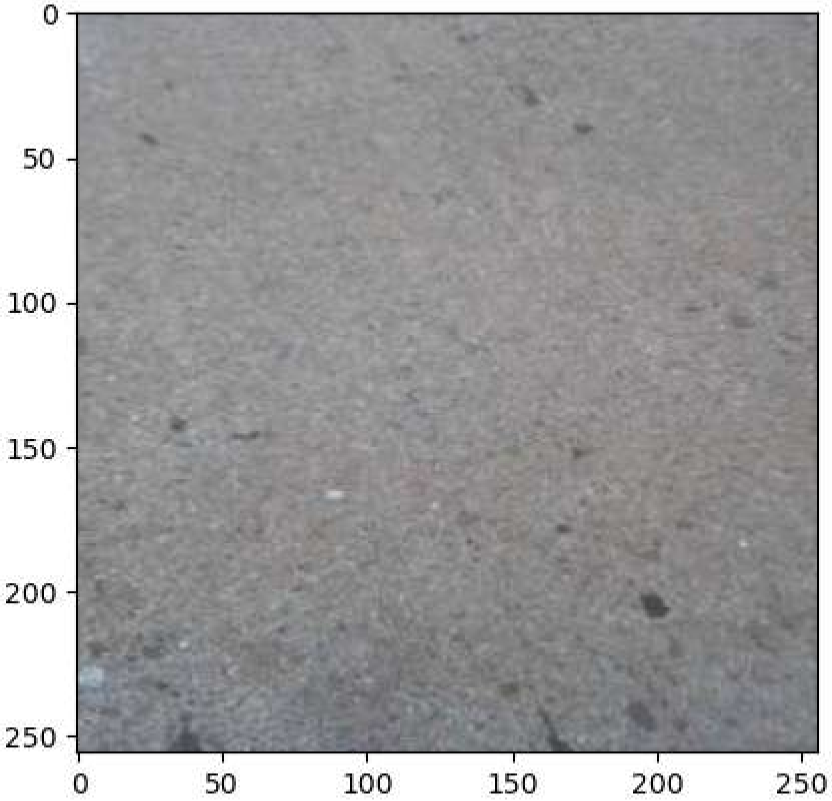
0
50
100
150
200
250
0
50
100
150
200
250

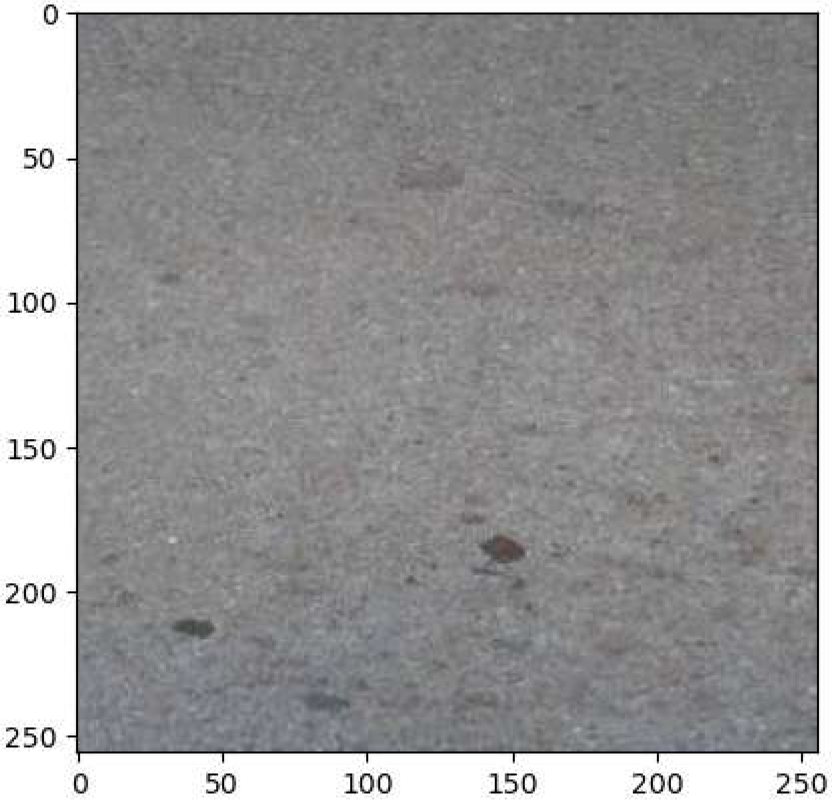
0
50
100
150
200
250
0
50
100
150
200
250

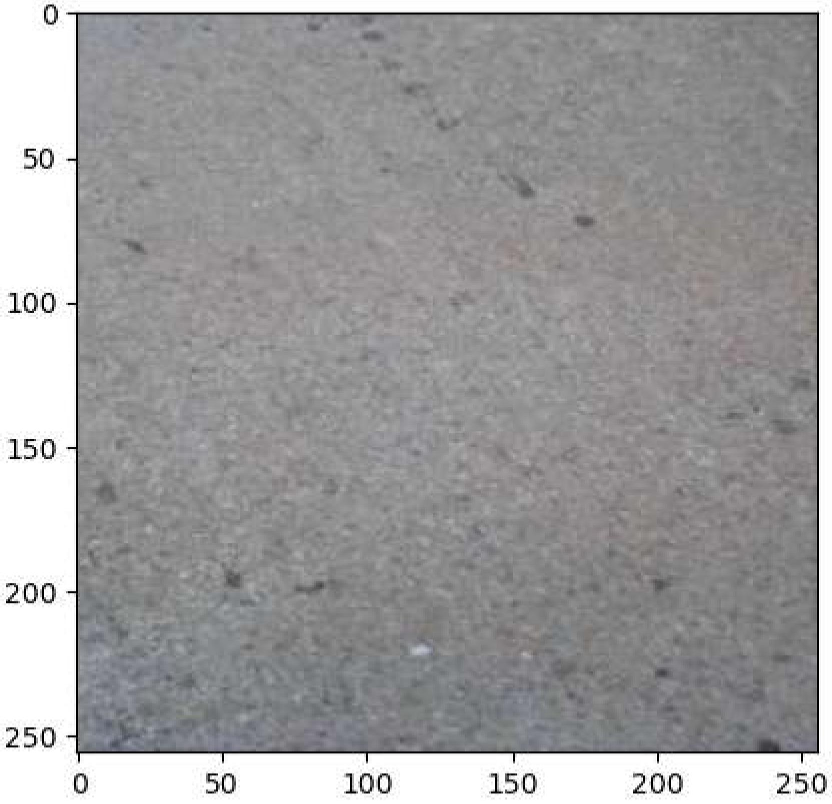
0
50
100
150
200
250
0
50
100
150
200
250

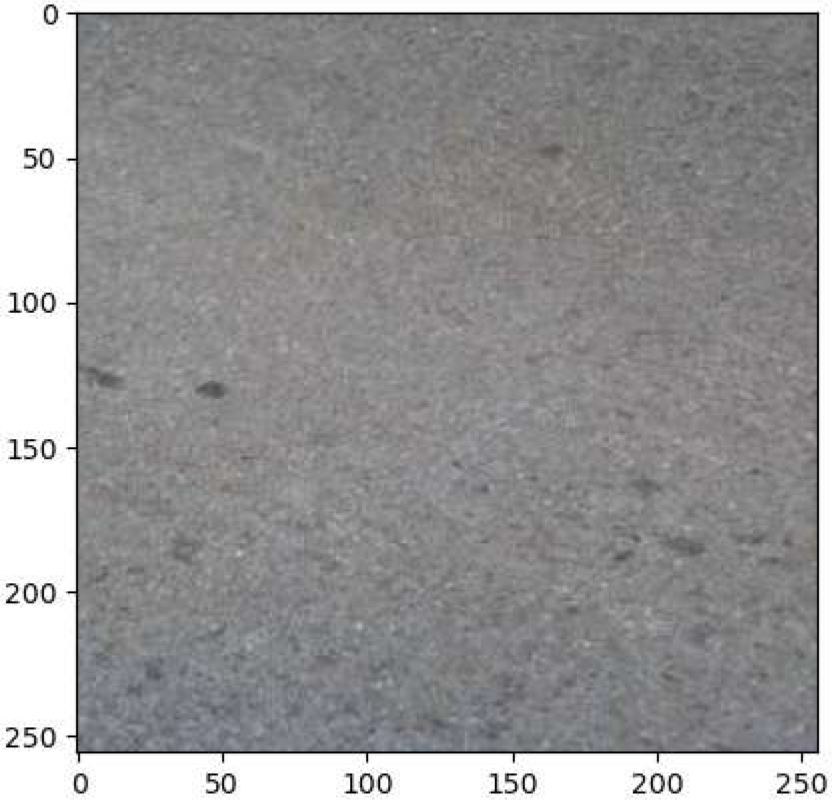
0
50
100
150
200
250
0
50
100
150
200
250

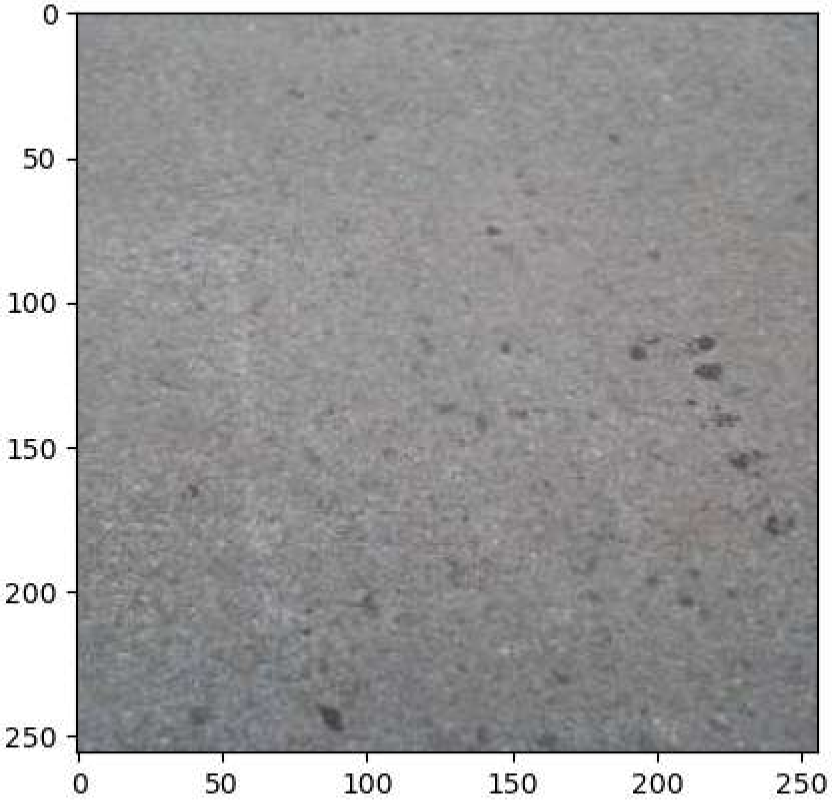
0
50
100
150
200
250
0
50
100
150
200
250

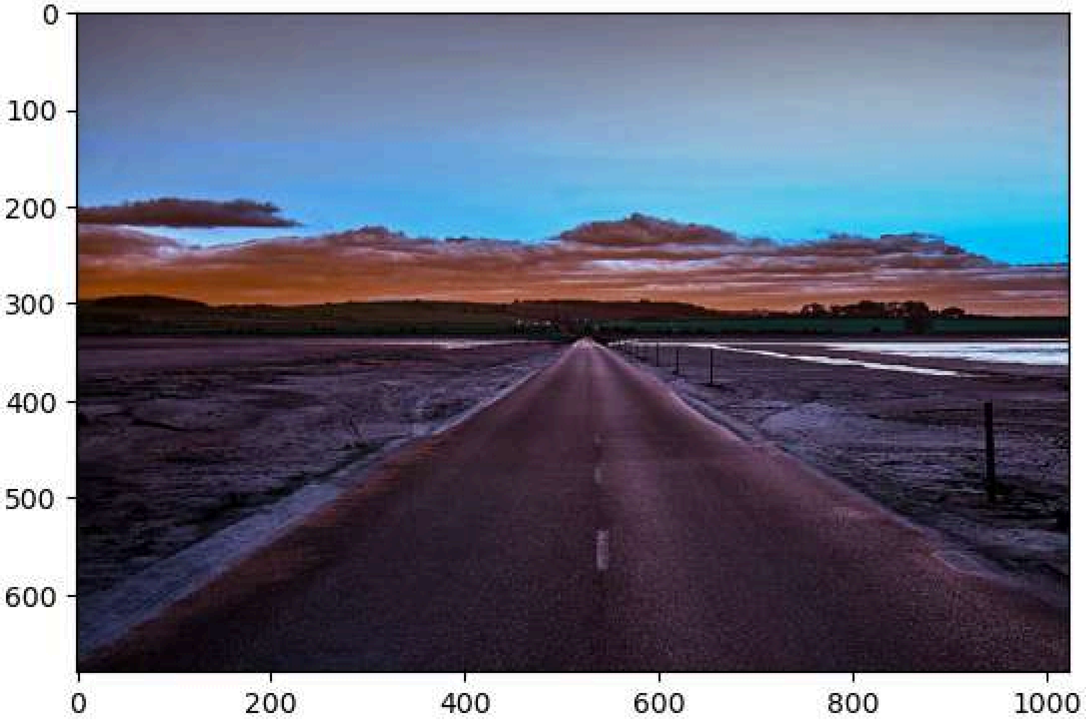
0
100
200
300
400
500
600
0
200
400
600
800
1000

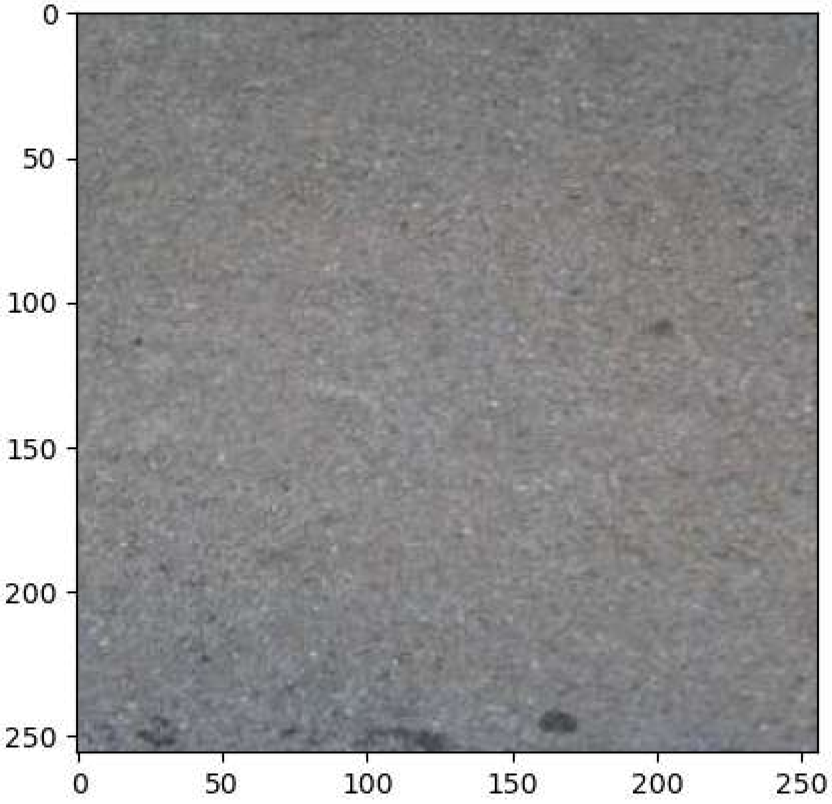
0
50
100
150
200
250
0
50
100
150
200
250

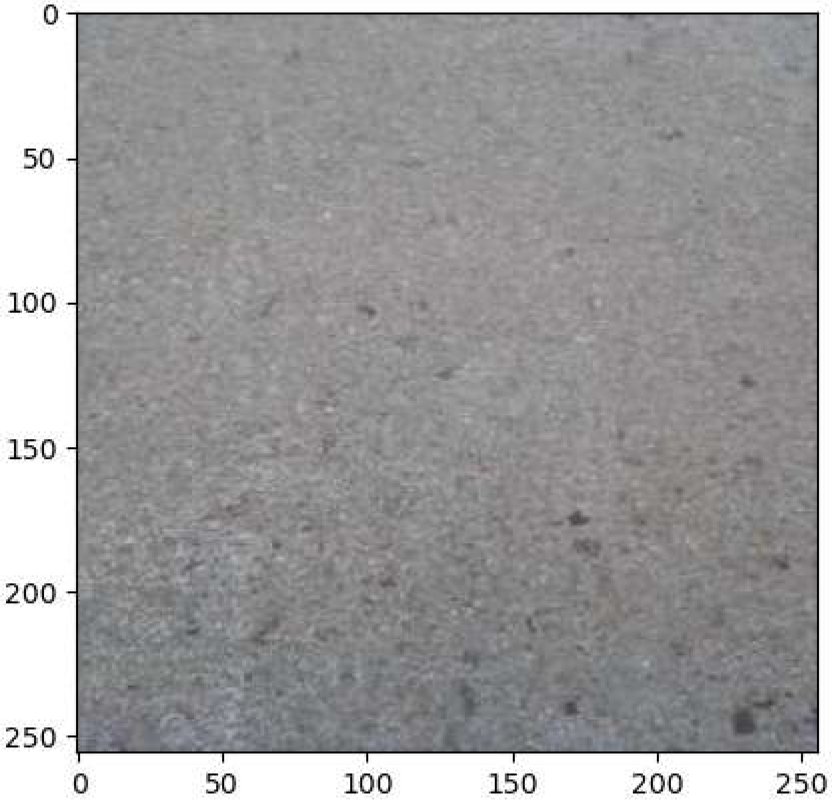
0
50
100
150
200
250
0
50
100
150
200
250

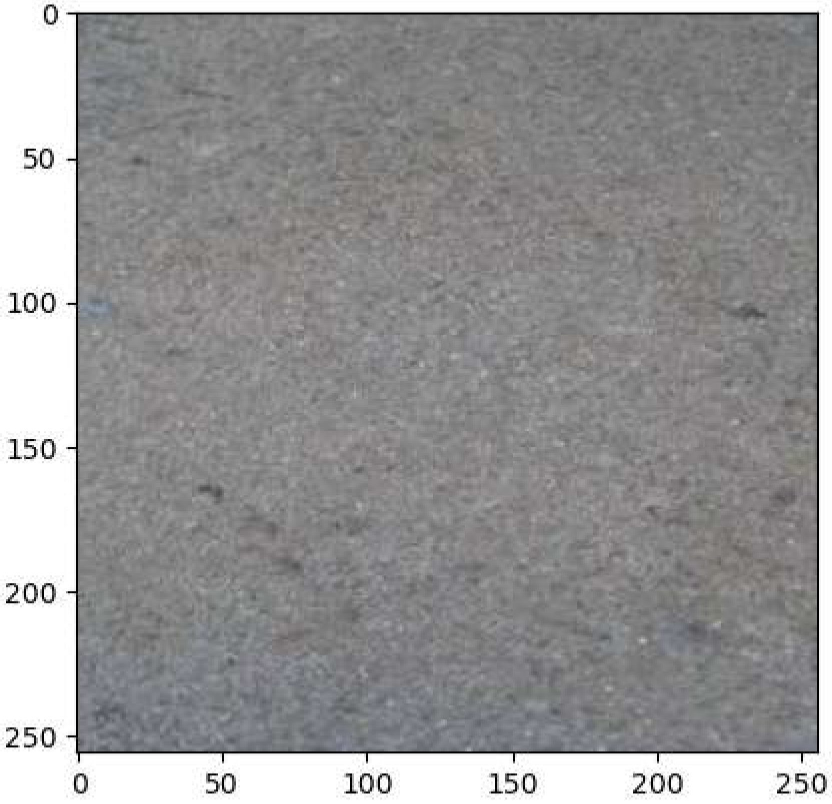
0
50
100
150
200
250
0 50 100 150 200 250

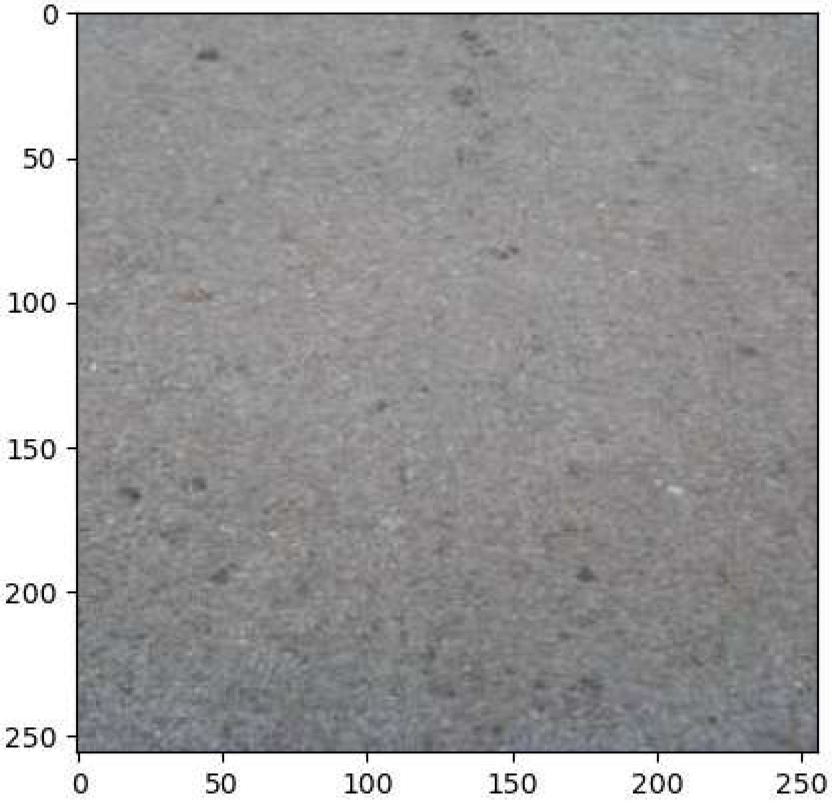
0
50
100
150
200
250
0 50 100 150 200 250

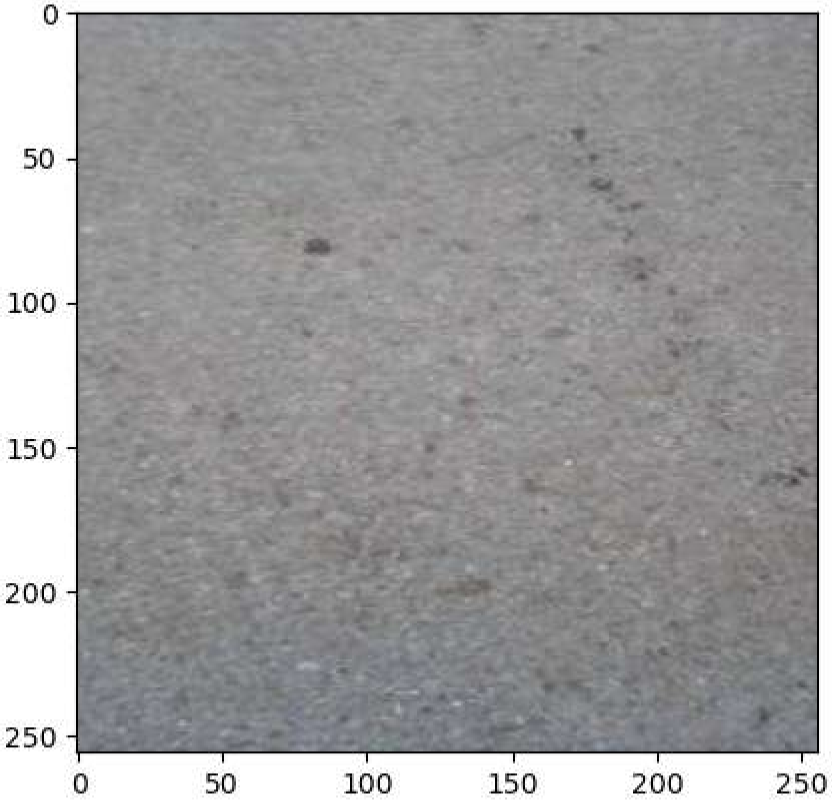
0
50
100
150
200
250
0
50
100
150
200
250

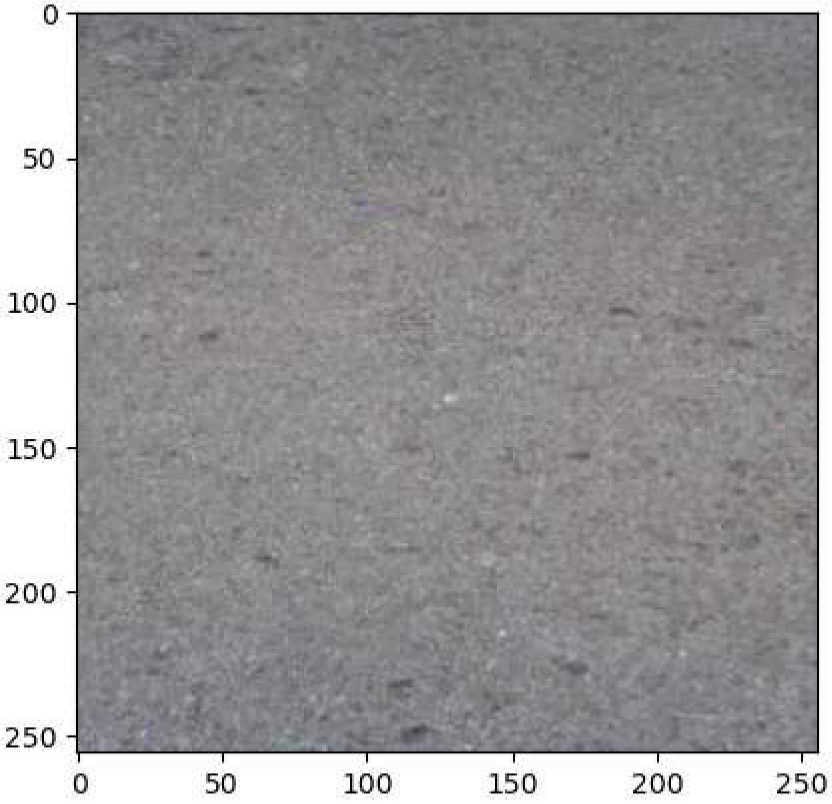
0
50
100
150
200
250
0
50
100
150
200
250

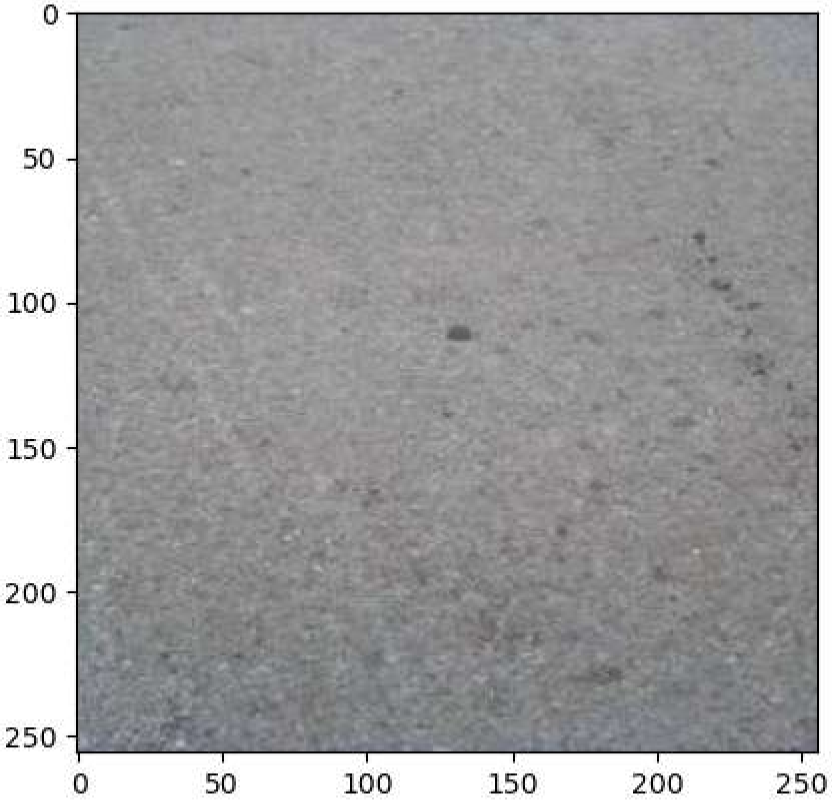
0
50
100
150
200
250
0
50
100
150
200
250

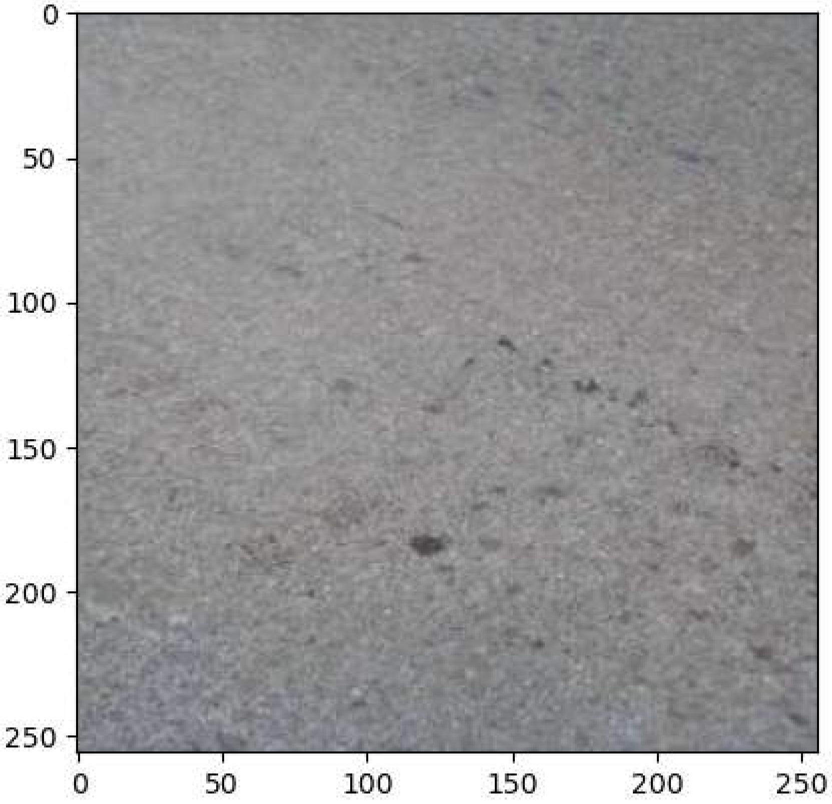
0
50
100
150
200
250
0
50
100
150
200
250

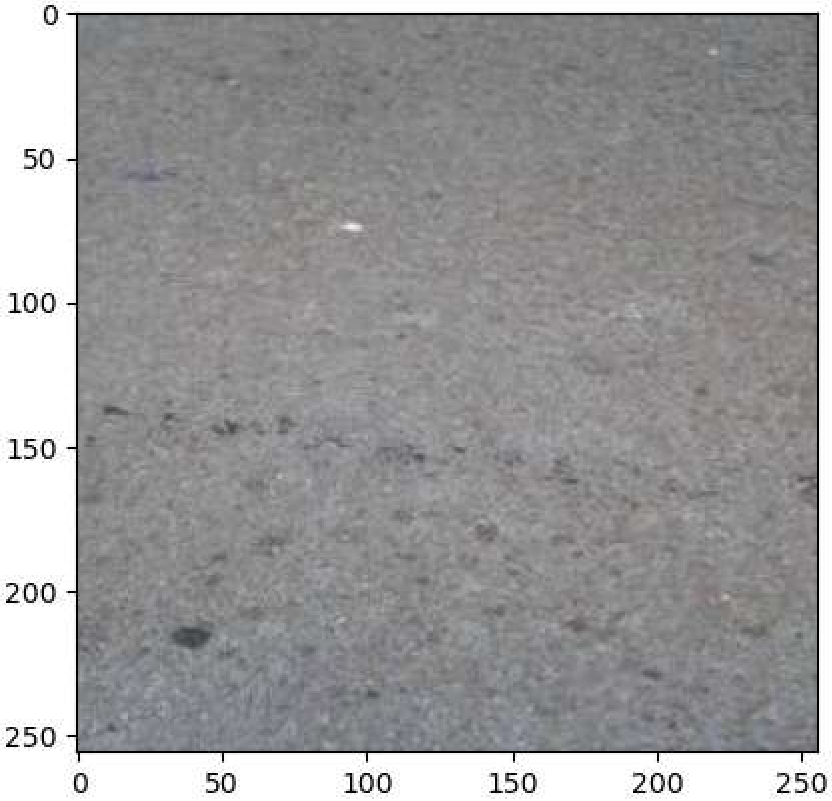
0
50
100
150
200
250
0
50
100
150
200
250

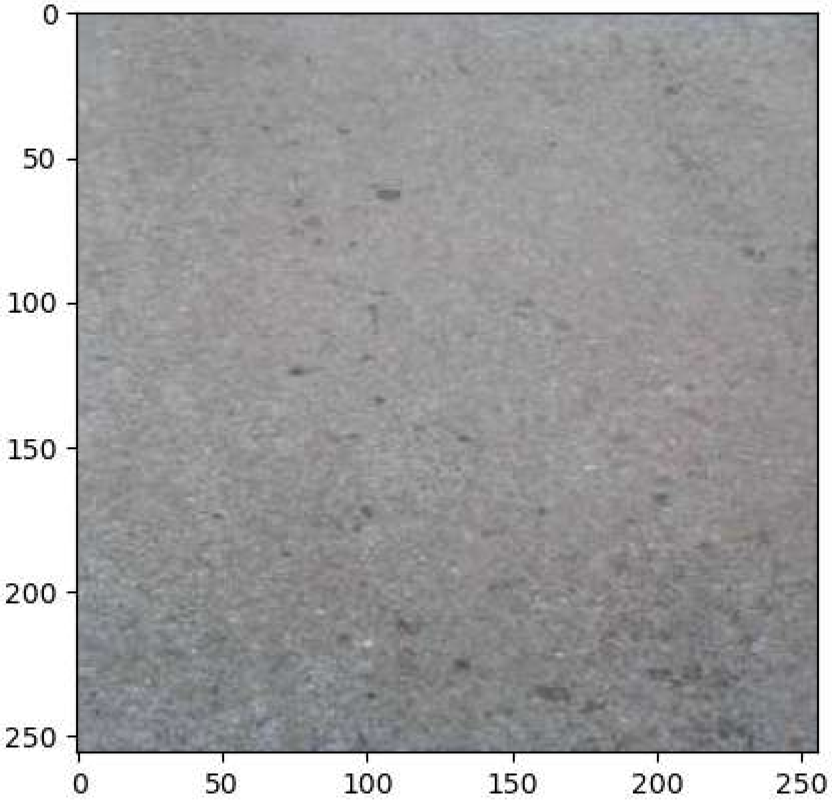
0
50
100
150
200
250
0
50
100
150
200
250

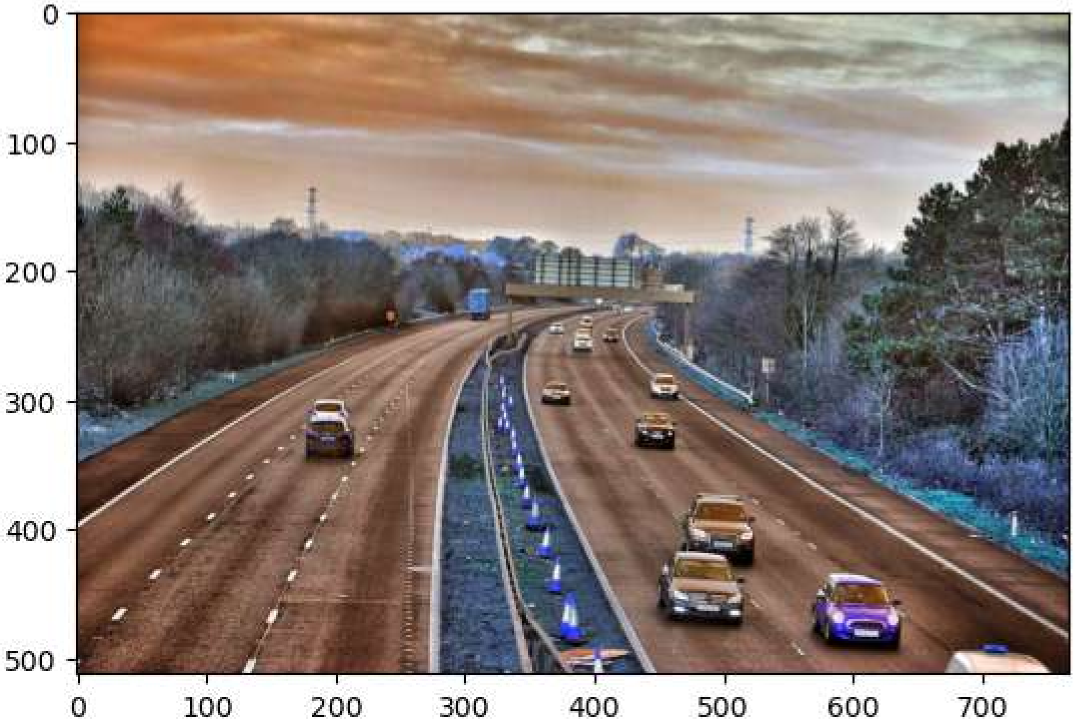
0
100
200
300
400
500
0
100
200
300
400
500
600
700

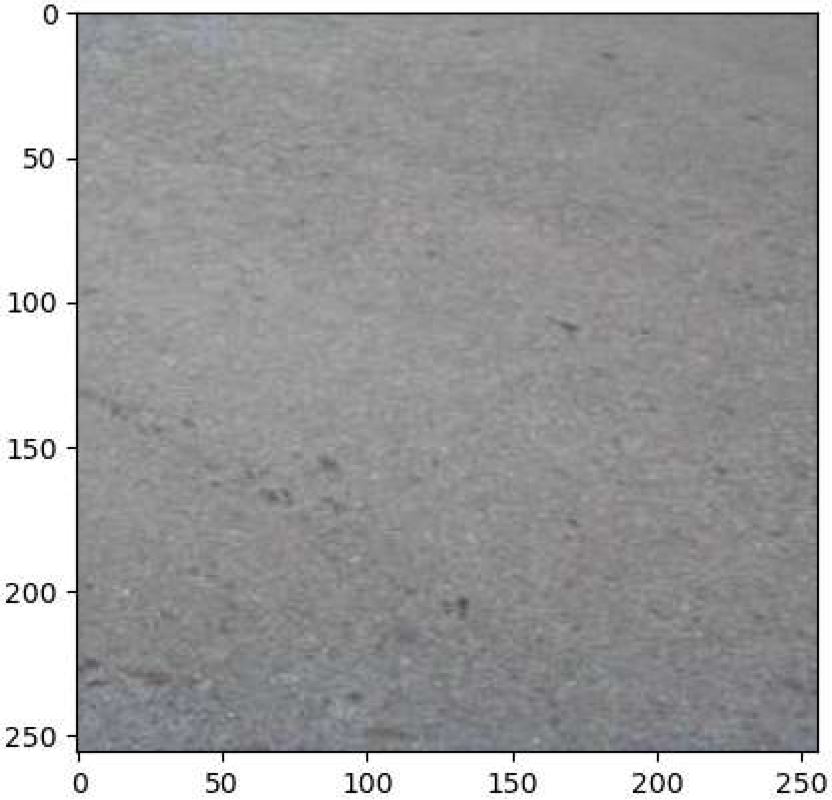
0
50
100
150
200
250
0
50
100
150
200
250

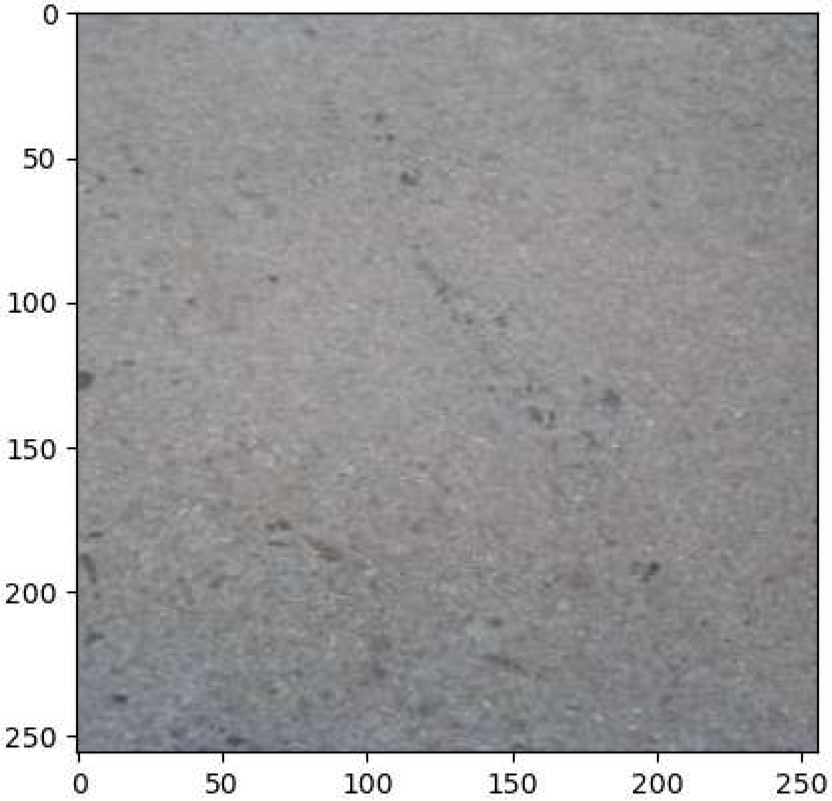
0
50
100
150
200
250
0
50
100
150
200
250

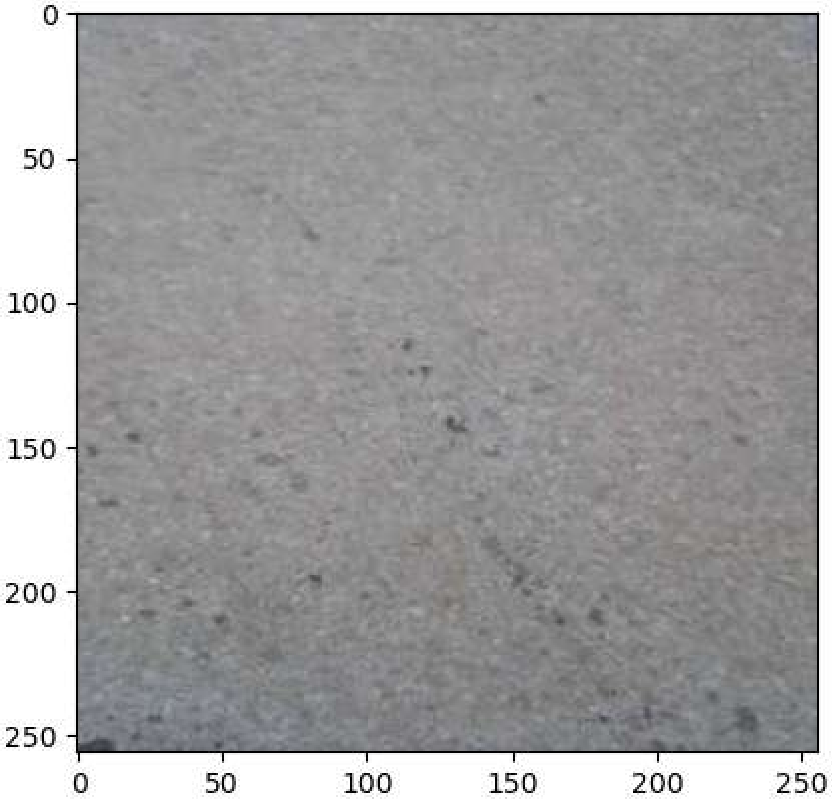
0
50
100
150
200
250
0
50
100
150
200
250

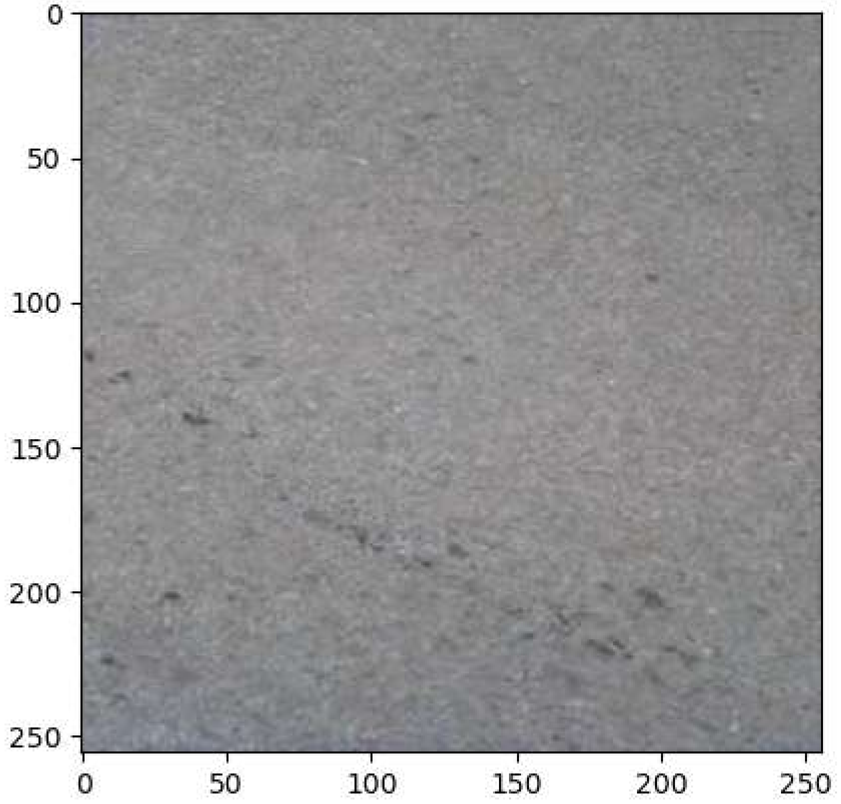
0
50
100
150
200
250
0 50 100 150 200 250

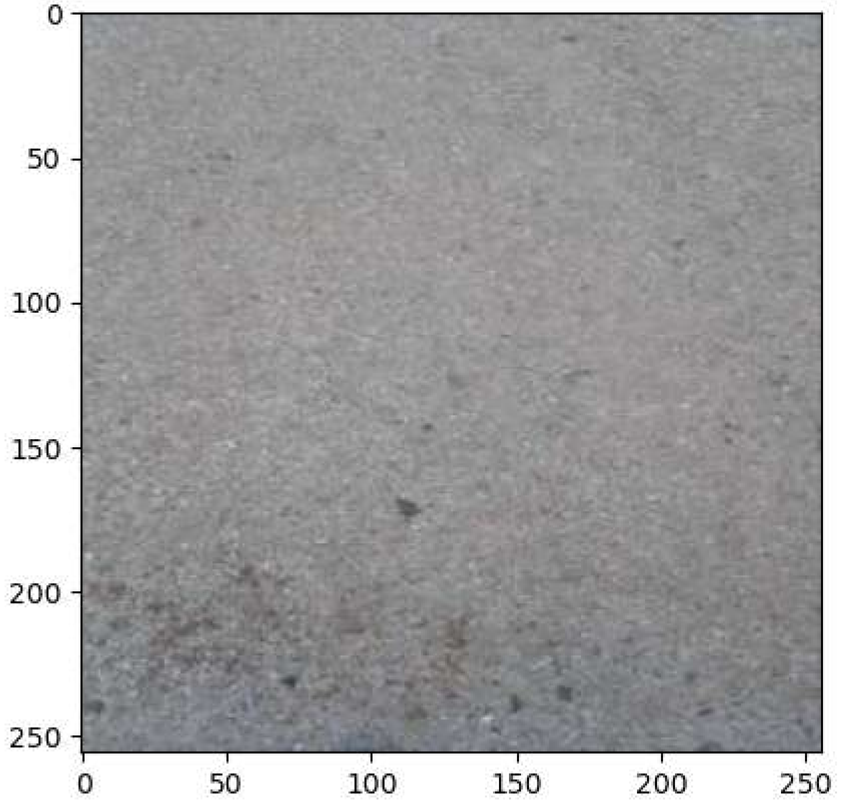
0
50
100
150
200
250
0 50 100 150 200 250

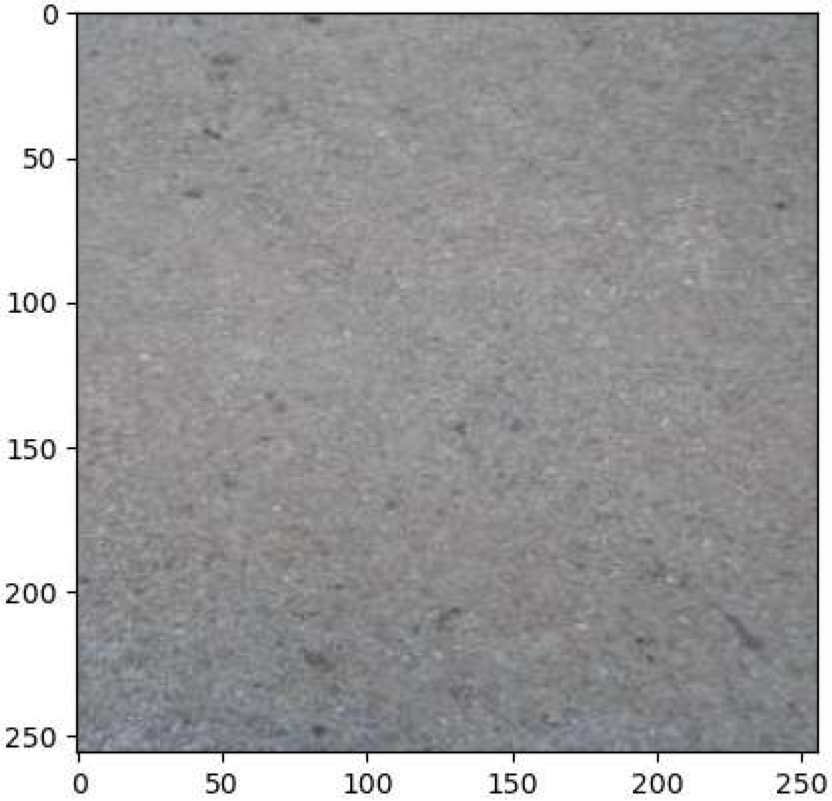
0
50
100
150
200
250
0
50
100
150
200
250

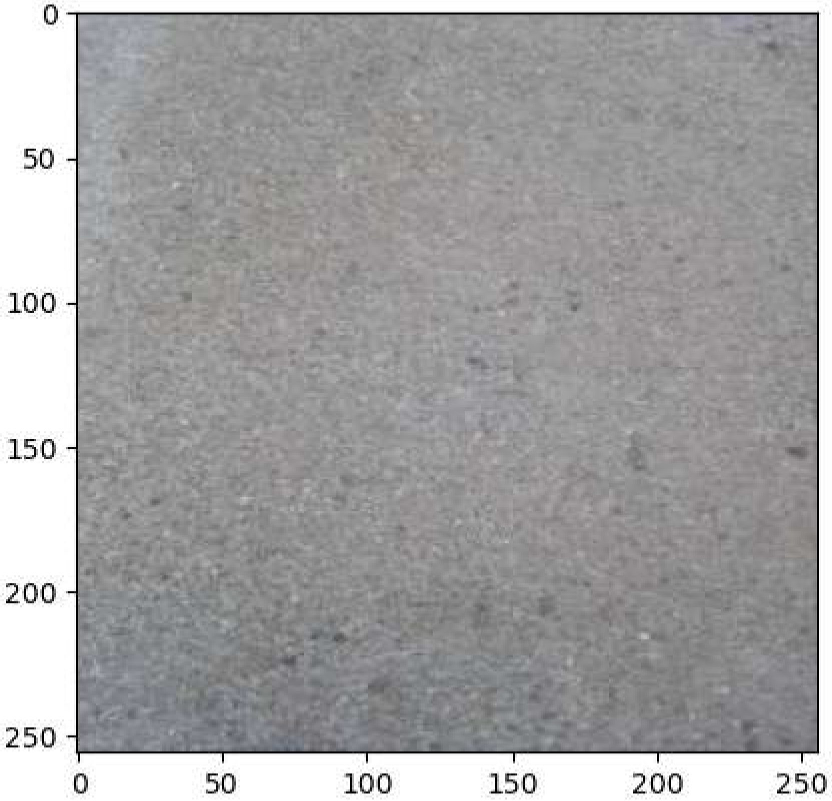
0
50
100
150
200
250
0
50
100
150
200
250

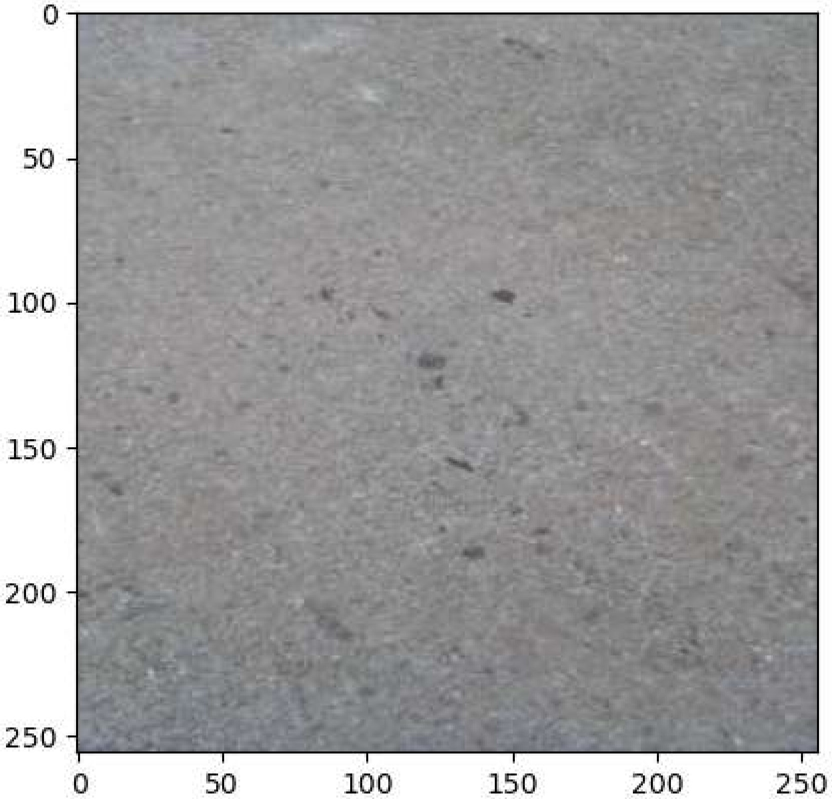

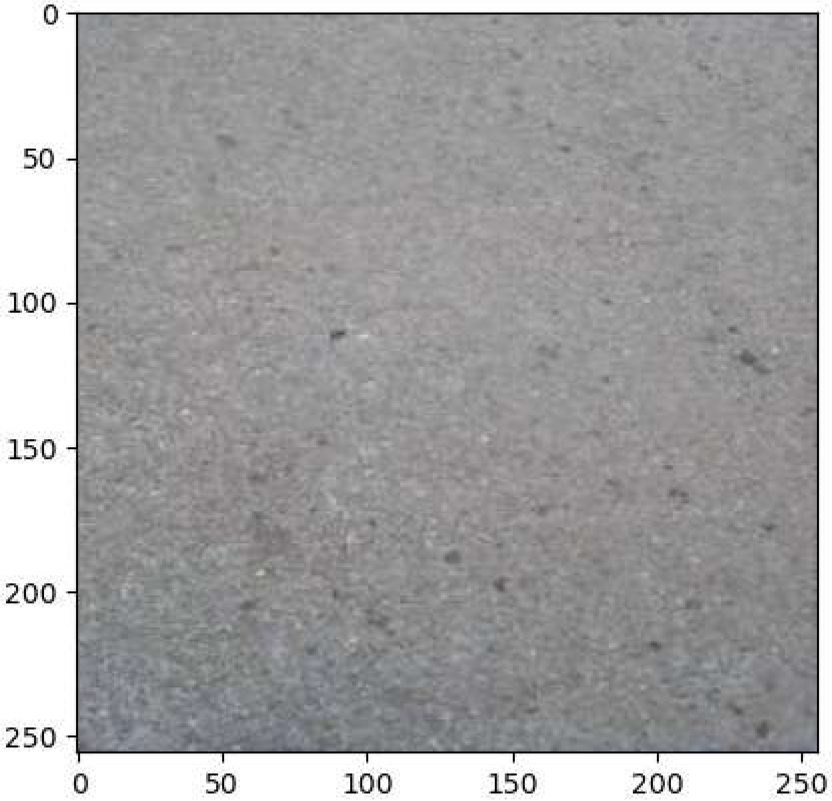

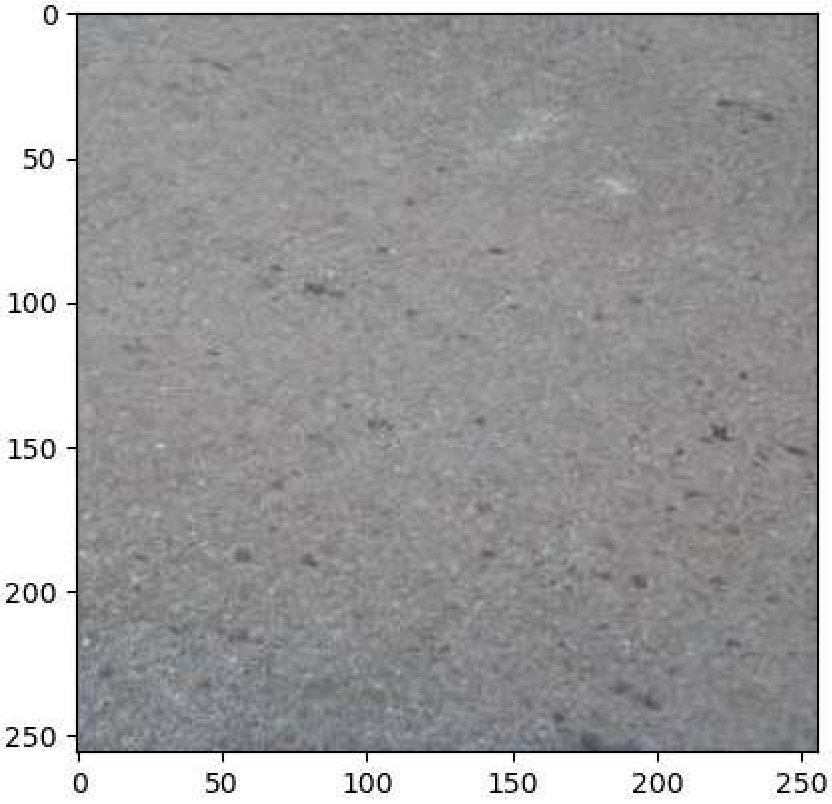
0
50
100
150
200
250
0
50
100
150
200
250

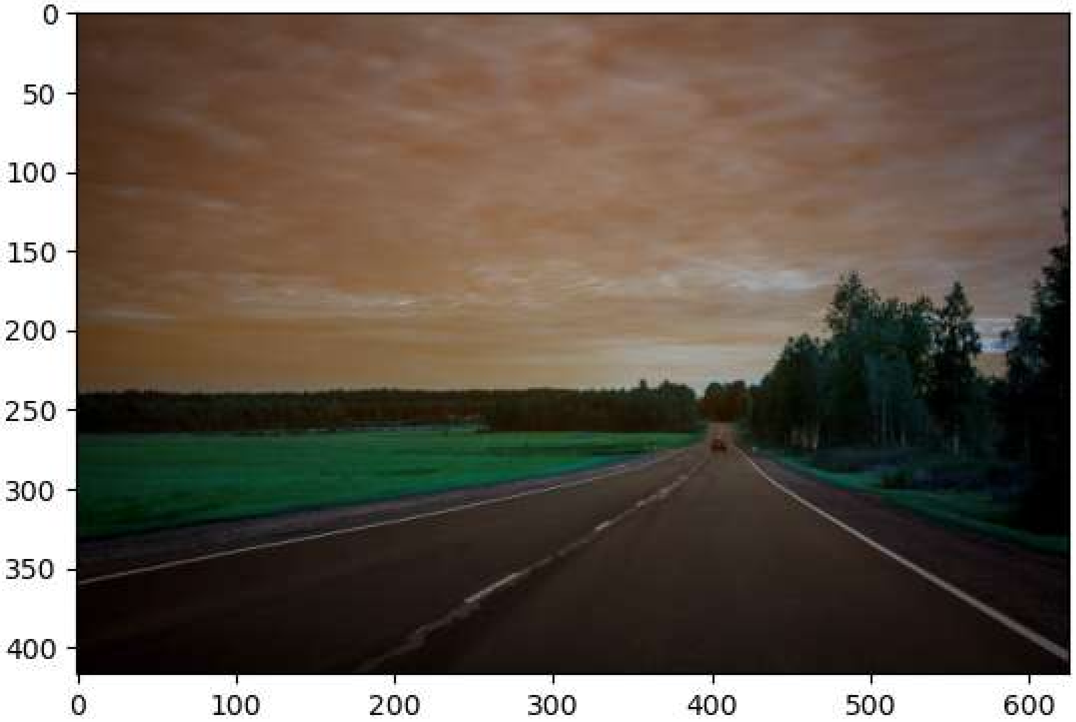
0
50
100
150
200
250
300
350
400
0
100
200
300
400
500
600

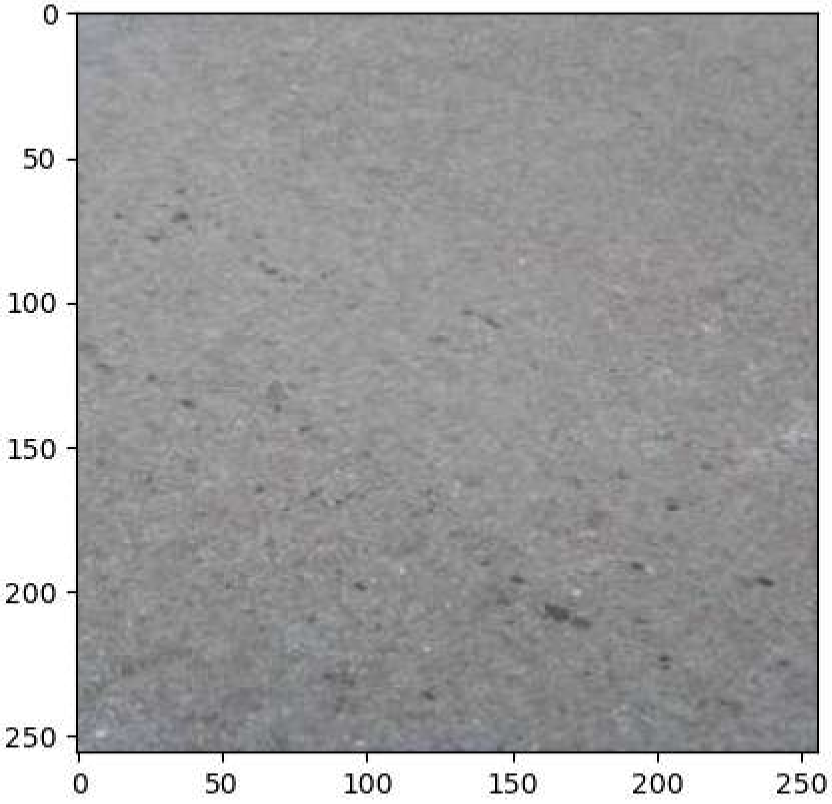
0
50
100
150
200
250
0 50 100 150 200 250

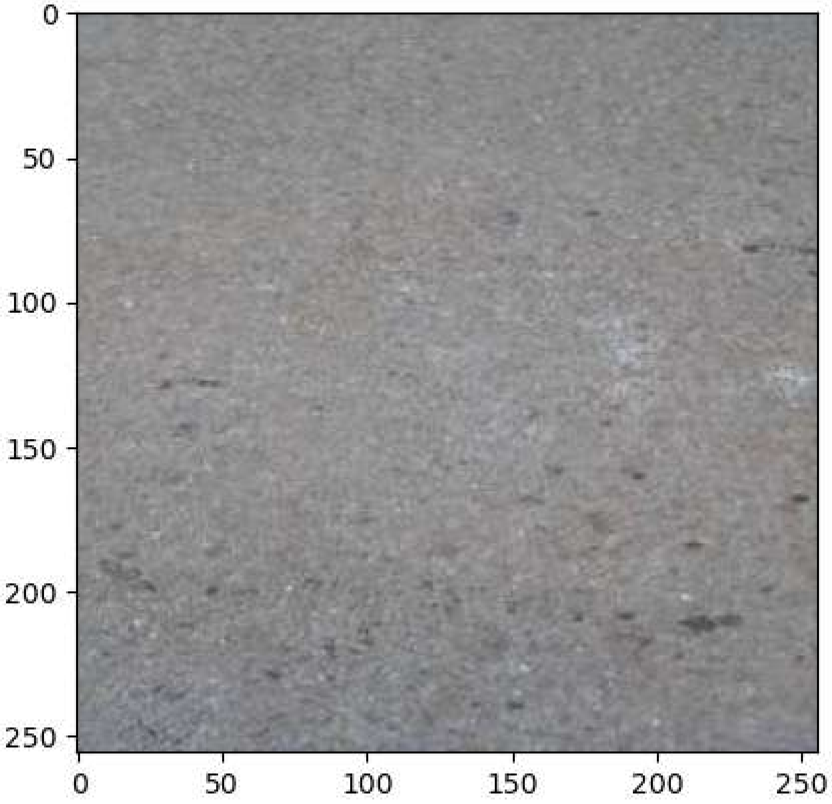
0
50
100
150
200
250
0 50 100 150 200 250

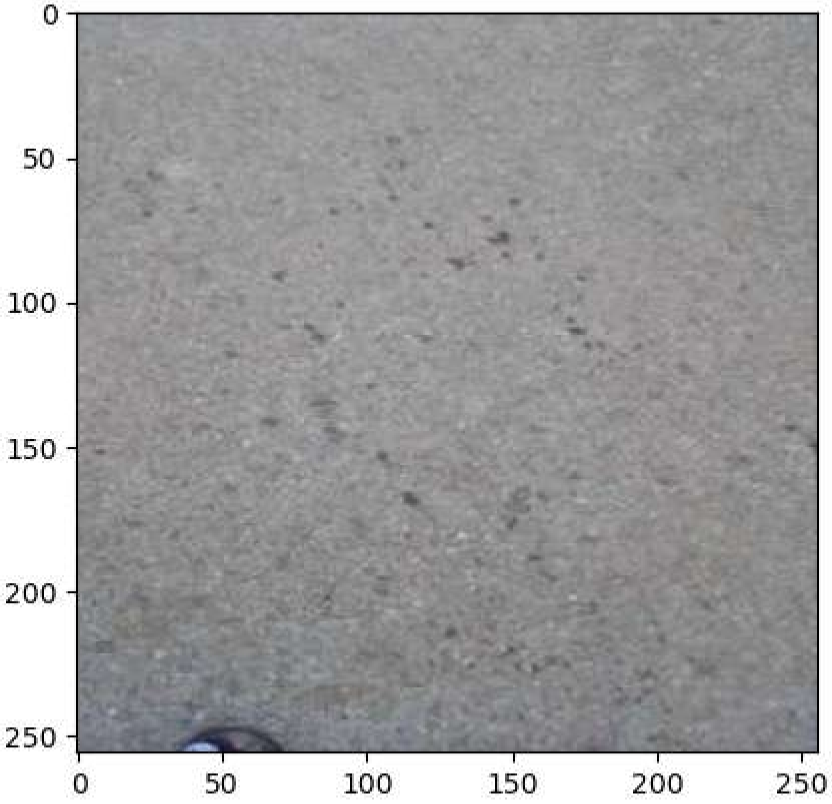
0
50
100
150
200
250
0
50
100
150
200
250

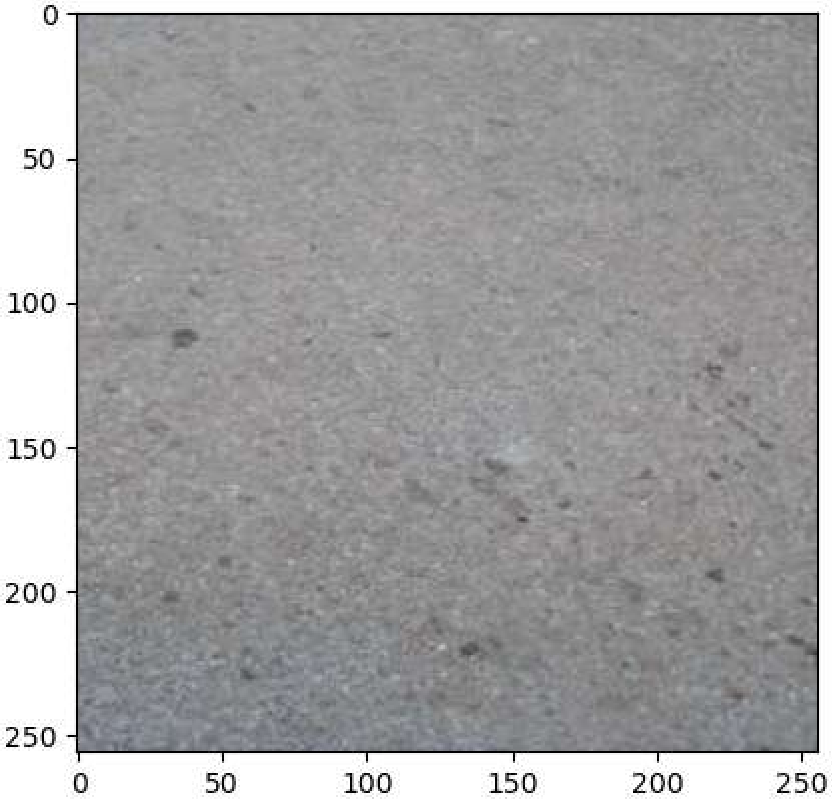
0
50
100
150
200
250
0
50
100
150
200
250

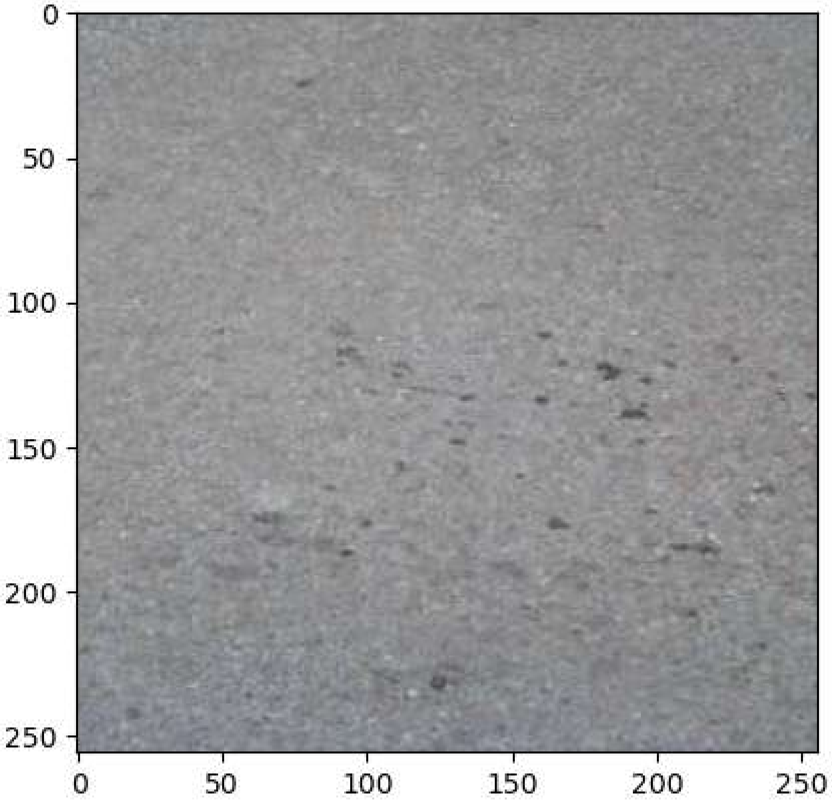
0
50
100
150
200
250
0 50 100 150 200 250

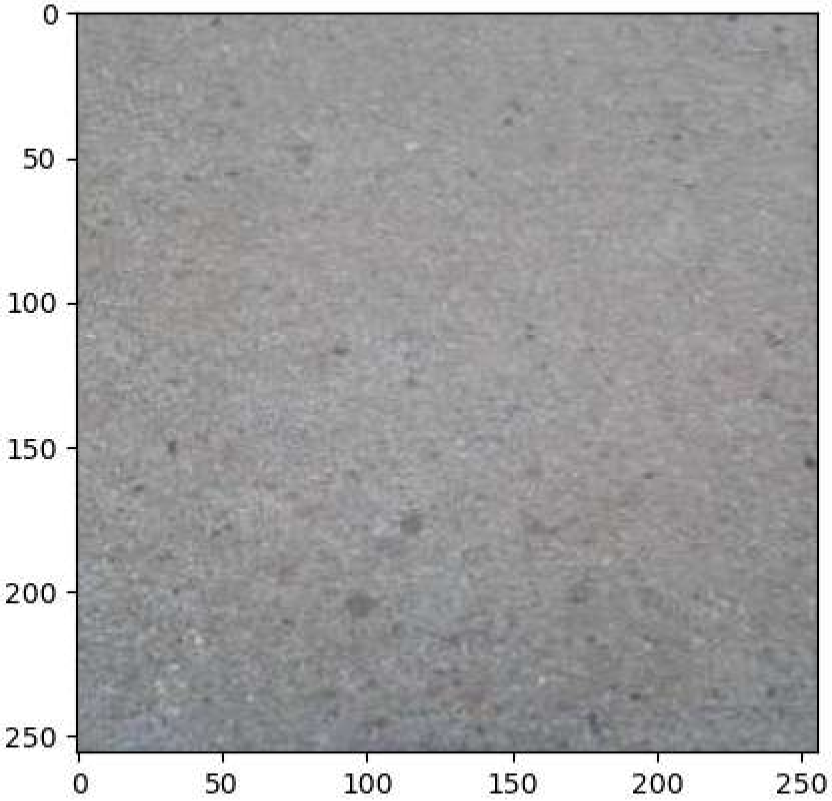
0
50
100
150
200
250
0 50 100 150 200 250

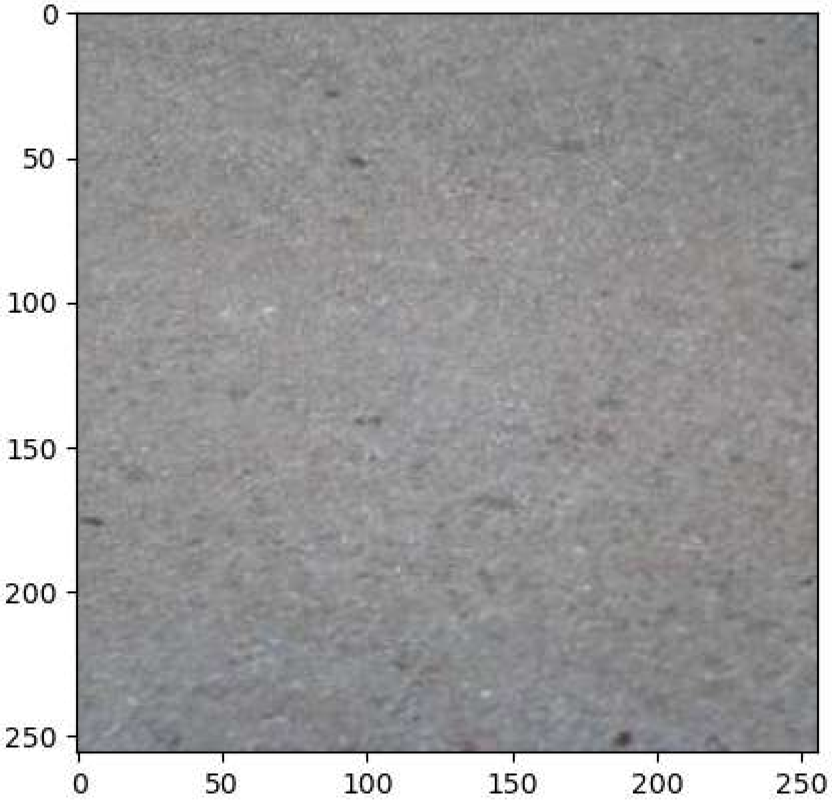
0
50
100
150
200
250
0
50
100
150
200
250

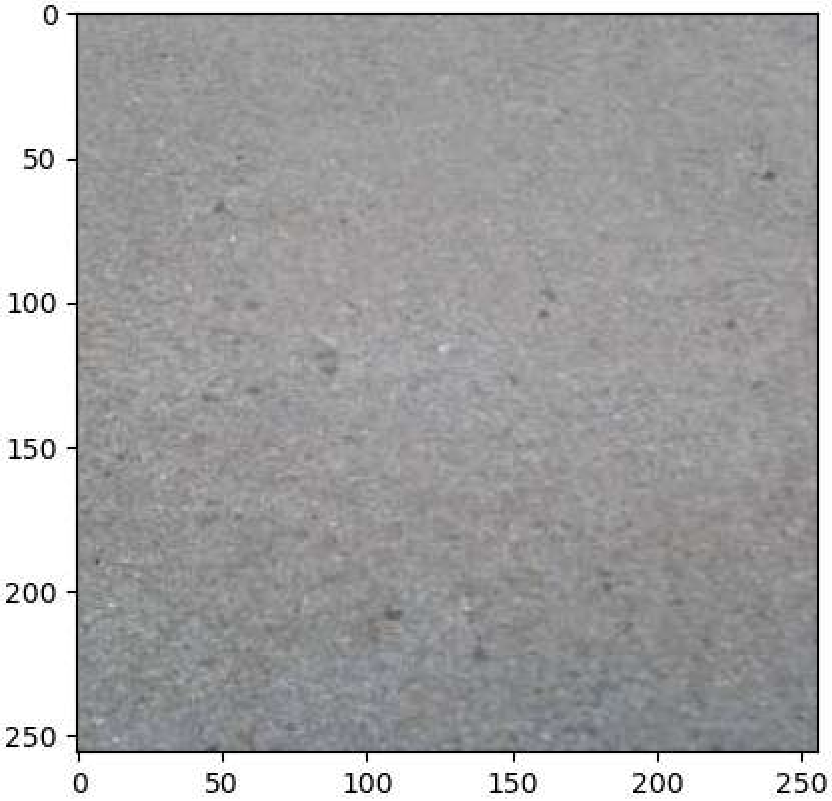
0
50
100
150
200
250
0
50
100
150
200
250

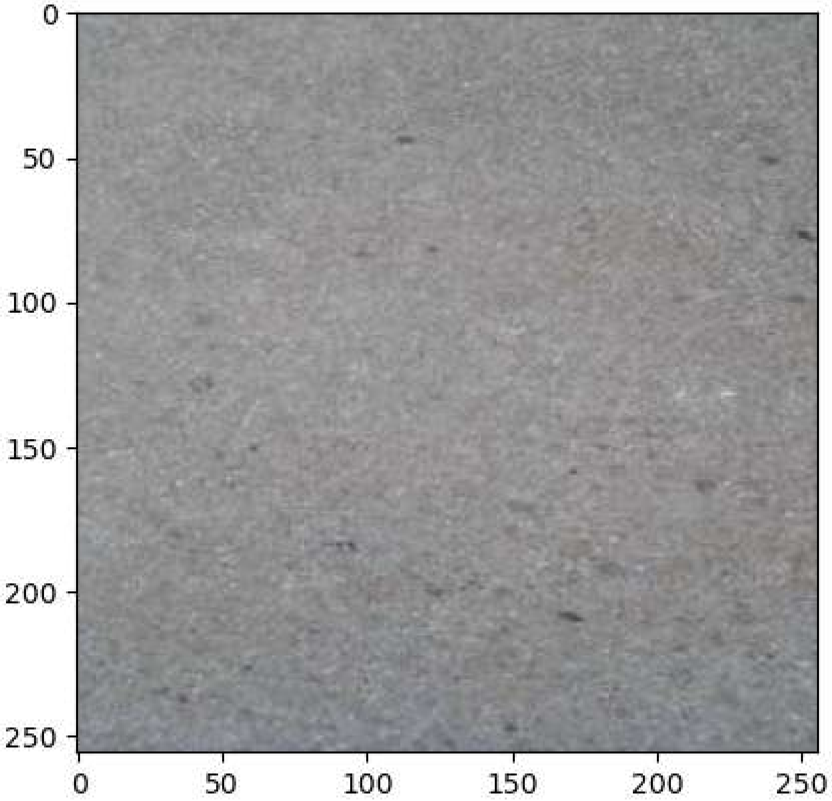
0
50
100
150
200
250
0
50
100
150
200
250

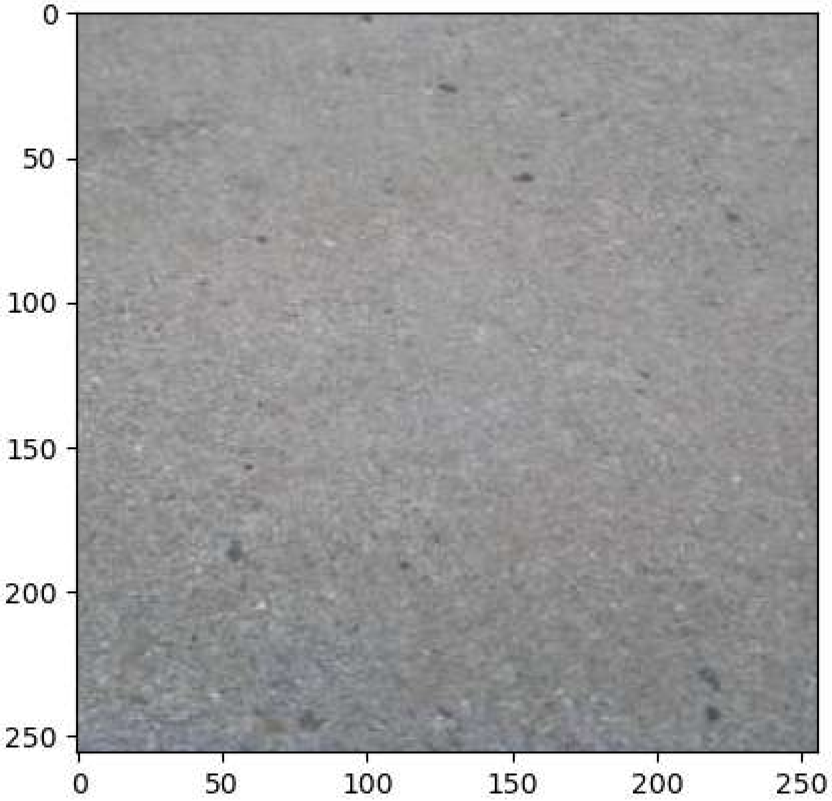
0
50
100
150
200
250
0
50
100
150
200
250

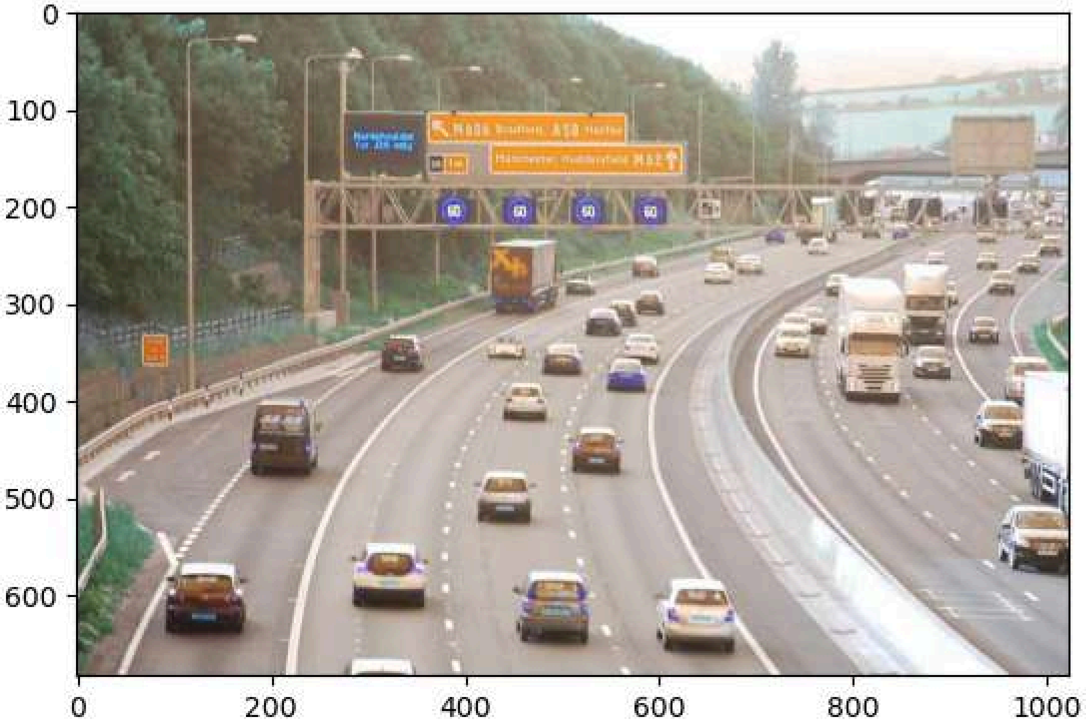
0
100
200
300
400
500
600
0
200
400
600
800
1000

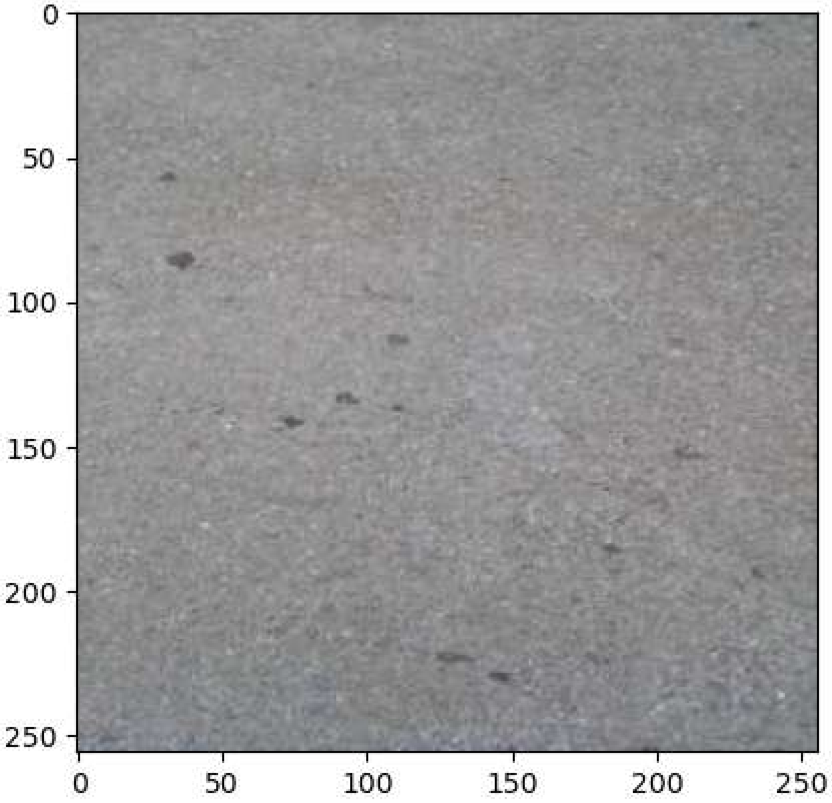
0
50
100
150
200
250
0
50
100
150
200
250

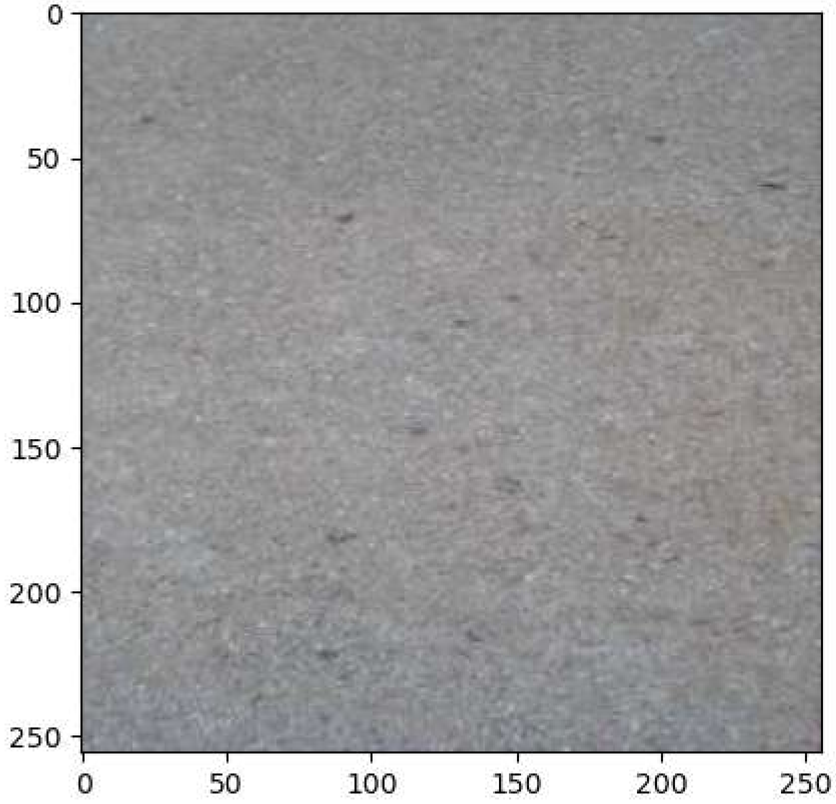
0
50
100
150
200
250
0
50
100
150
200
250

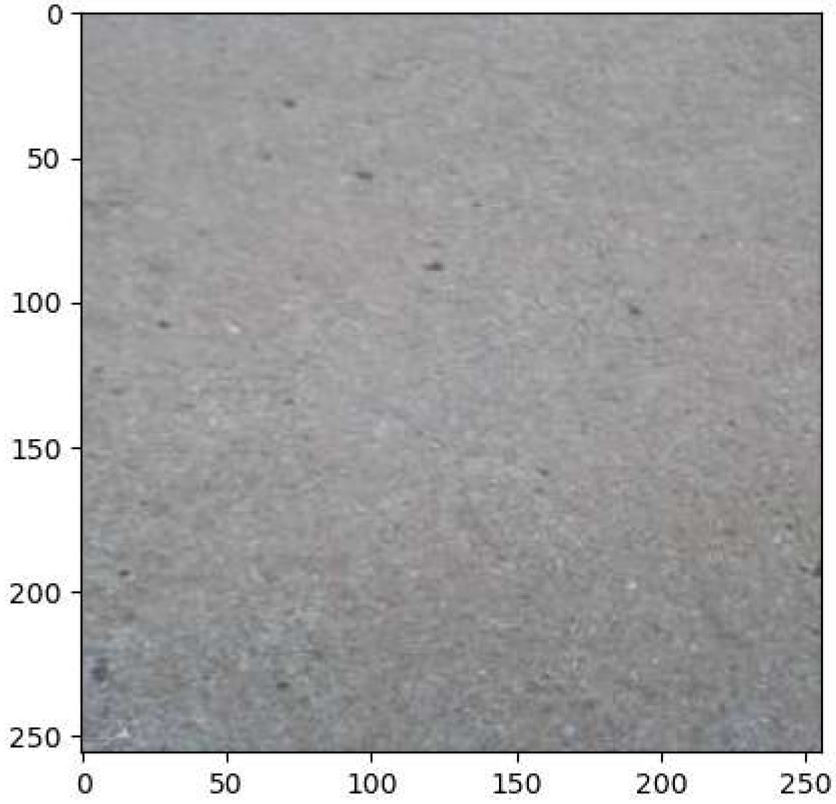
0
50
100
150
200
250
0
50
100
150
200
250

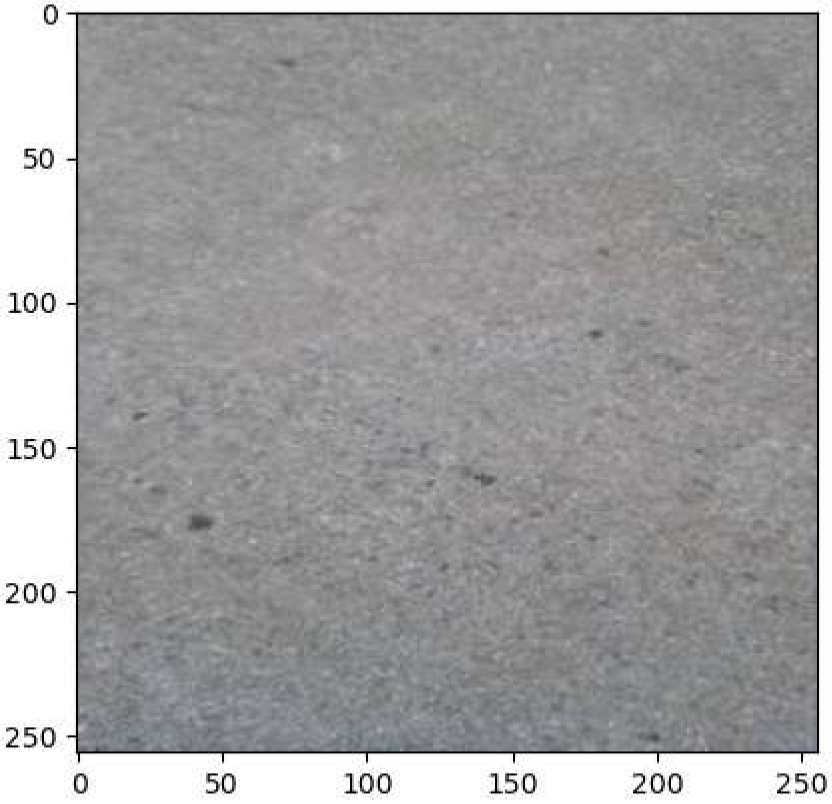
0
50
100
150
200
250
0
50
100
150
200
250

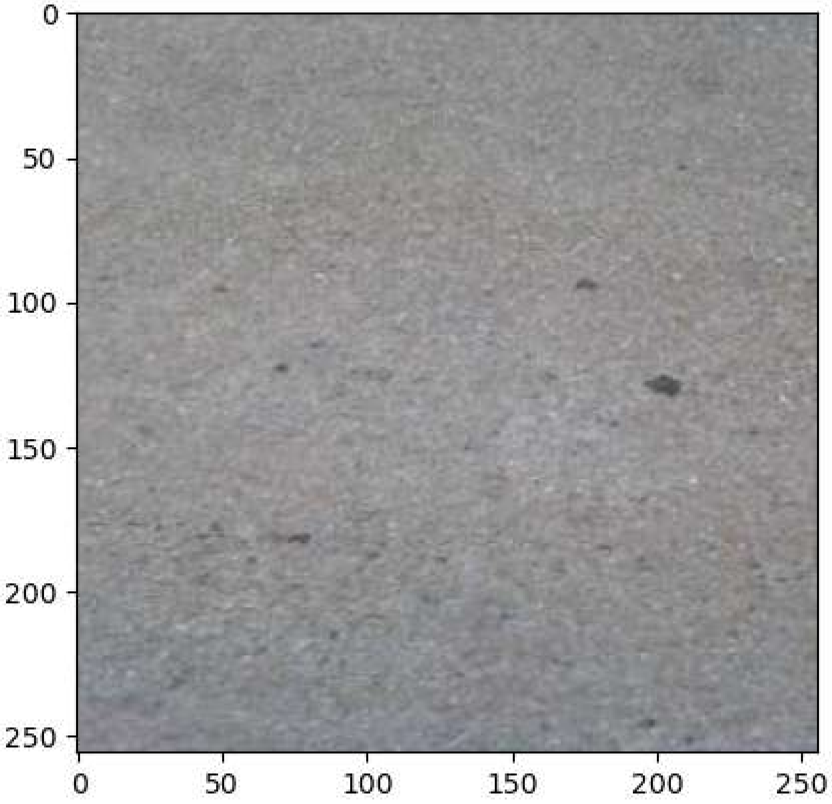
0
50
100
150
200
250
0
50
100
150
200
250

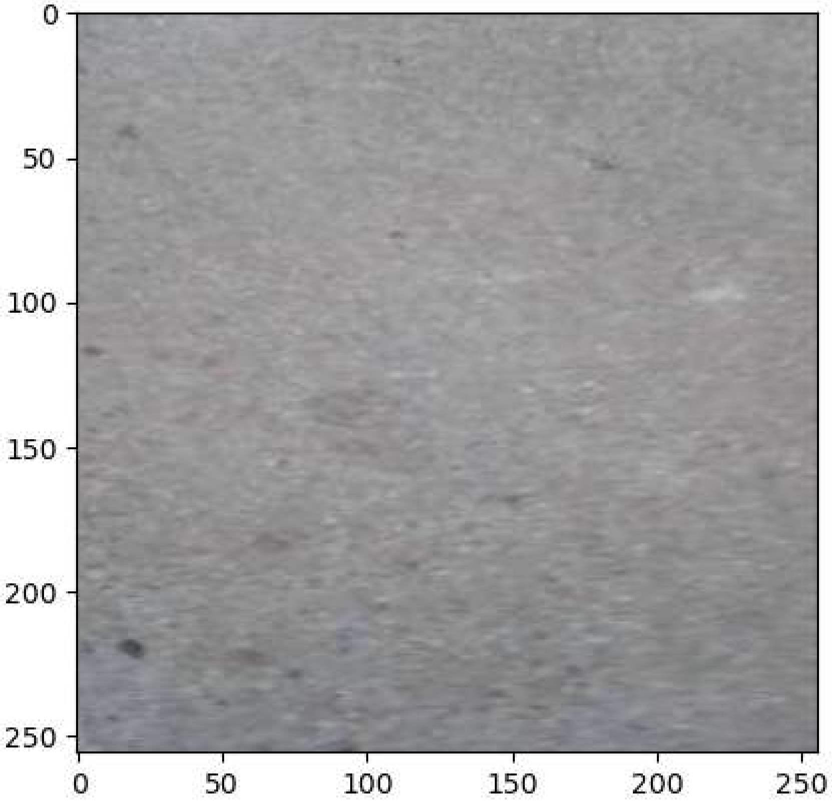
0
50
100
150
200
250
0
50
100
150
200
250

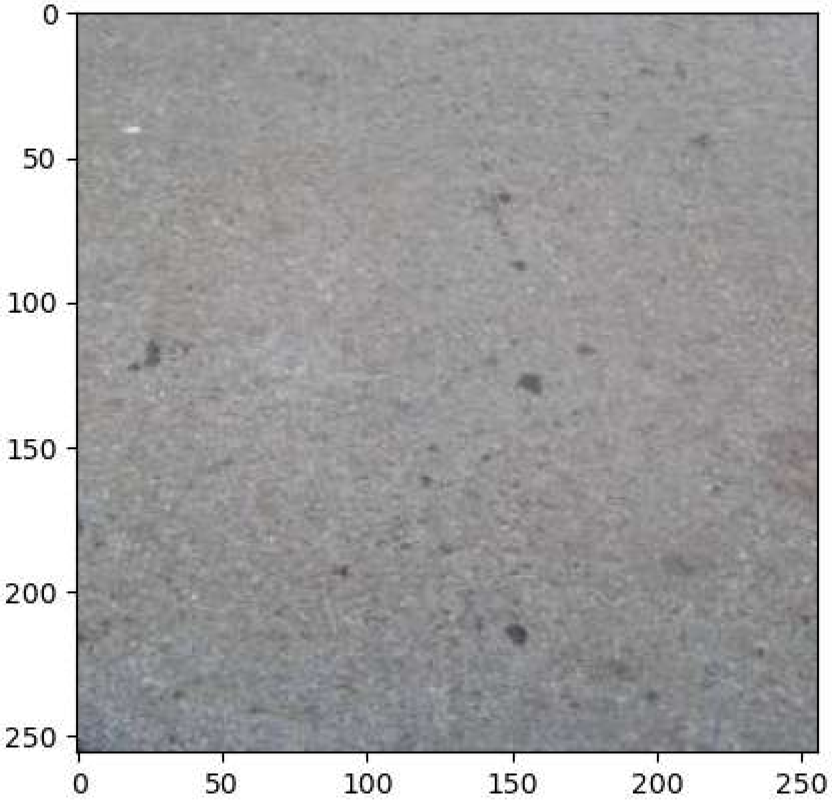
0
50
100
150
200
250
0
50
100
150
200
250

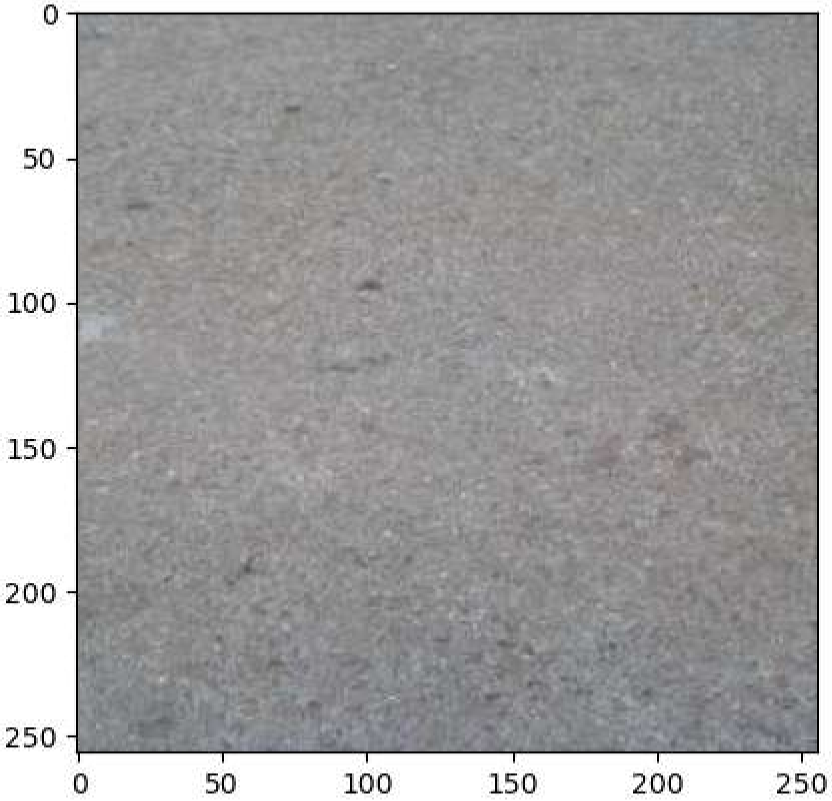
0
50
100
150
200
250
0
50
100
150
200
250

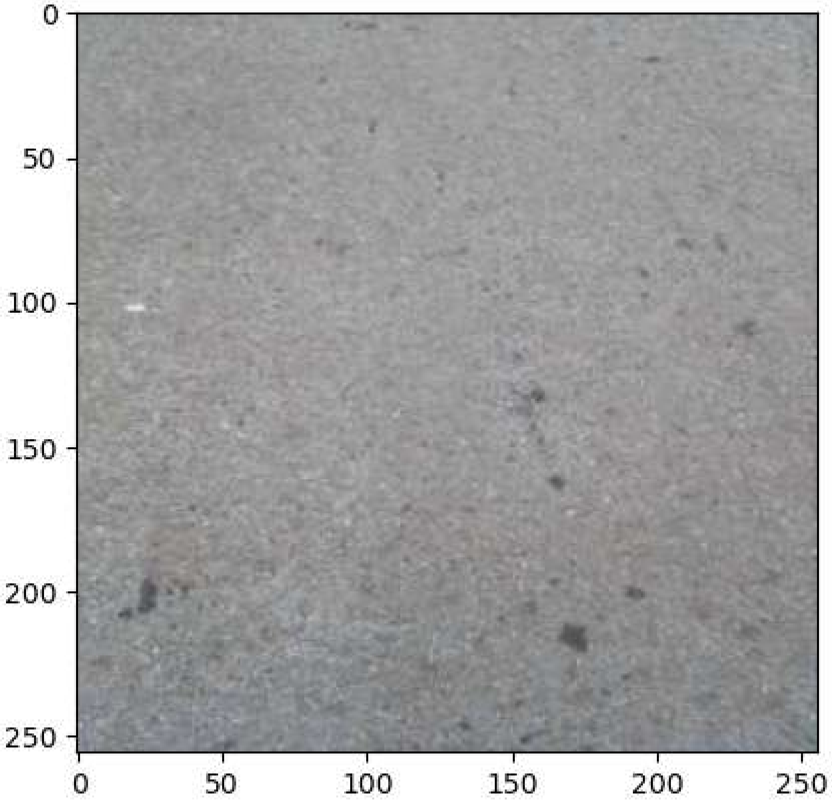
0
50
100
150
200
250
0
50
100
150
200
250

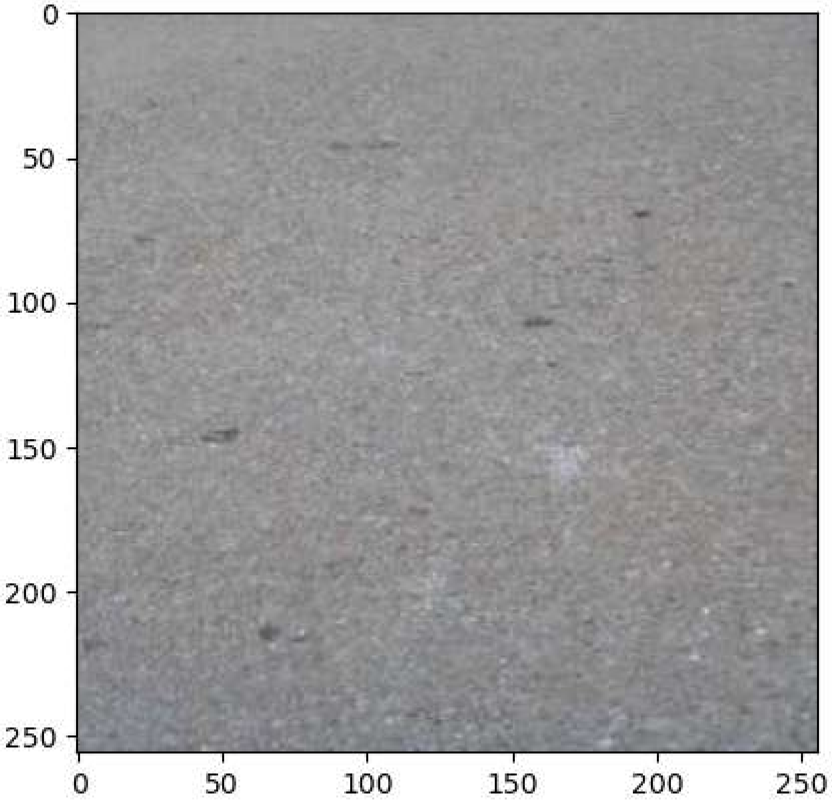
0
50
100
150
200
250
0 50 100 150 200 250

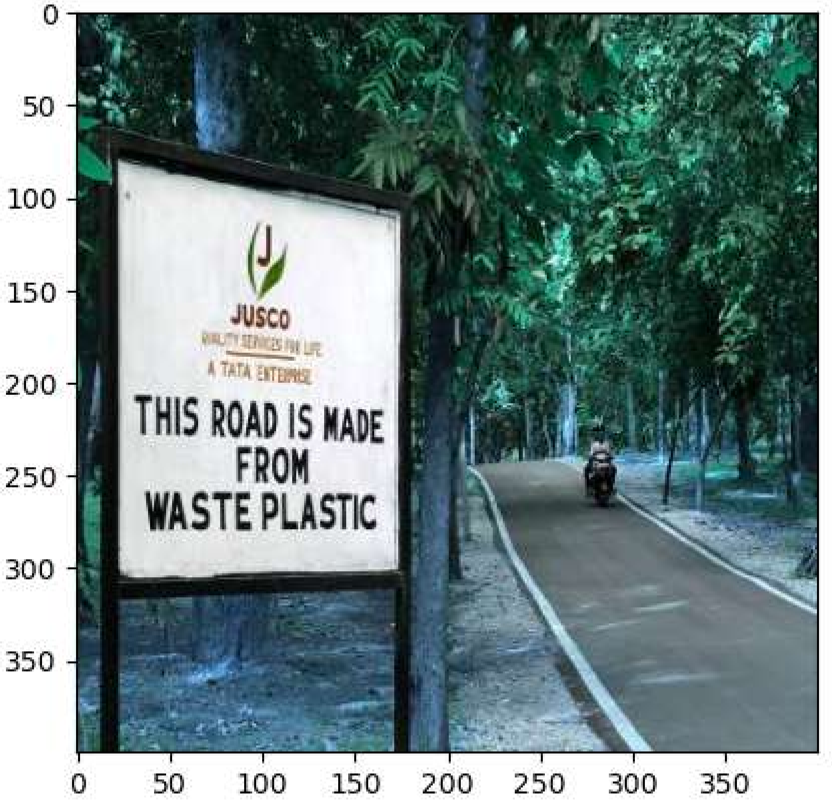
JUSCO
THIS ROAD IS MADE
FROM
WASTE PLASTIC
0
50
100
150
200
250
300
350
0 50 100 150 200 250 300 350

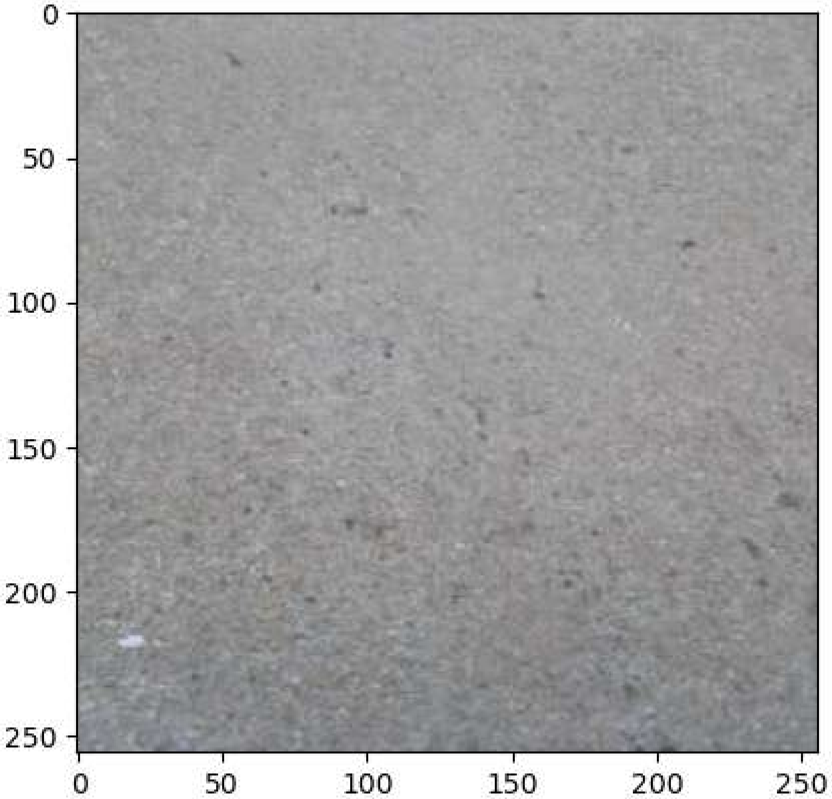
0
50
100
150
200
250
0 50 100 150 200 250

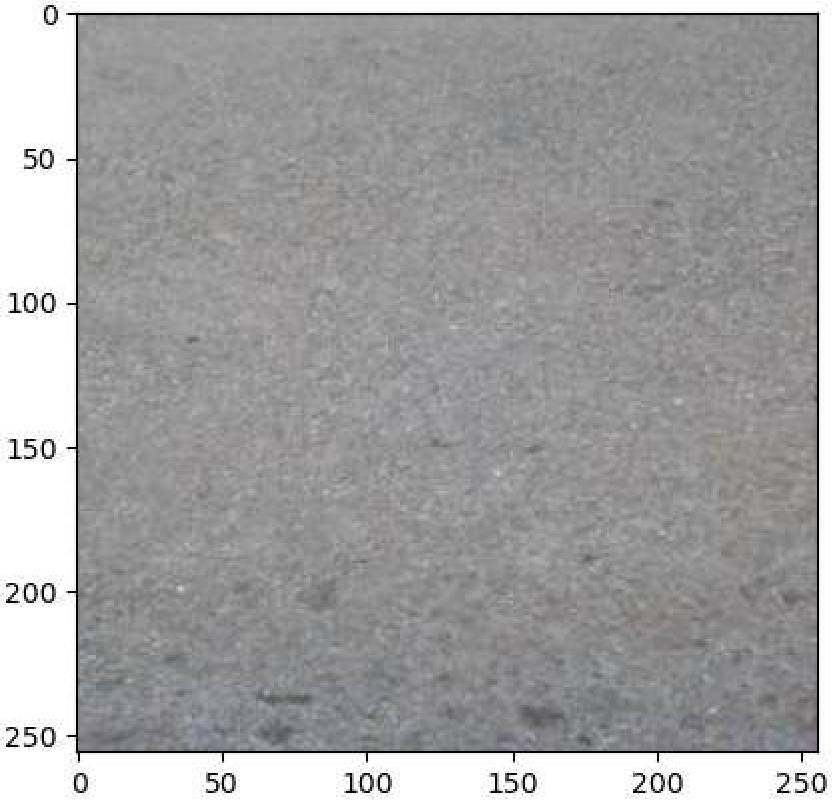
0
50
100
150
200
250
0 50 100 150 200 250

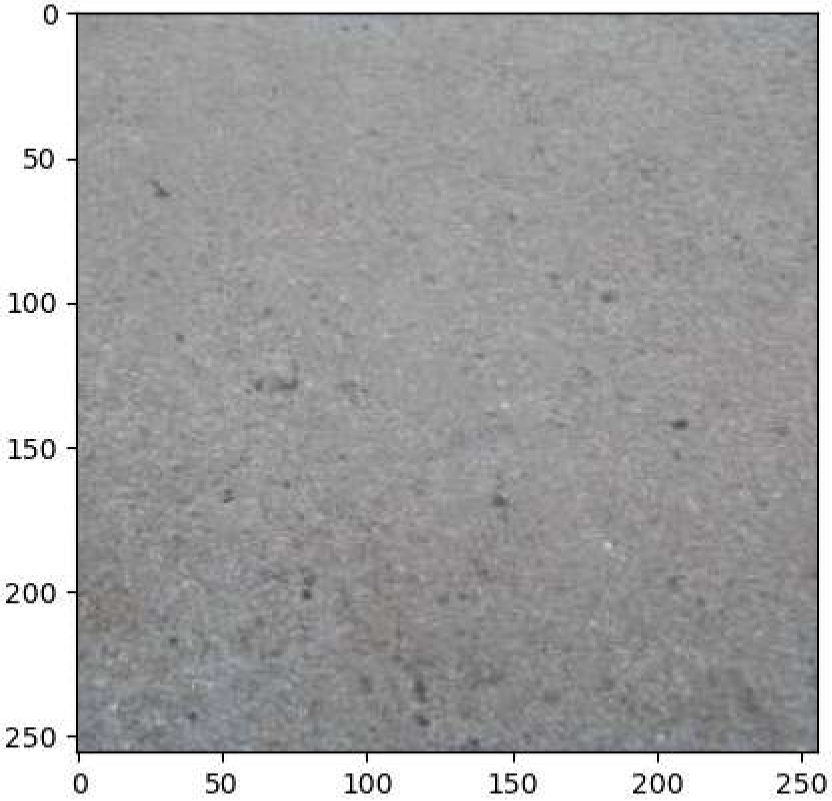
0
50
100
150
200
250
0
50
100
150
200
250

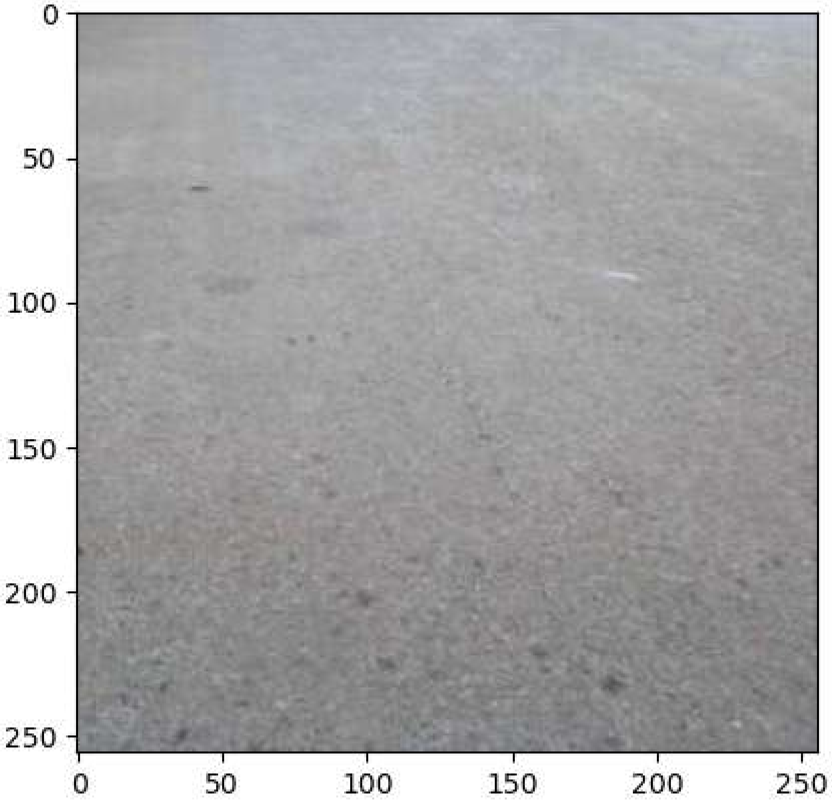
0
50
100
150
200
250
0
50
100
150
200
250

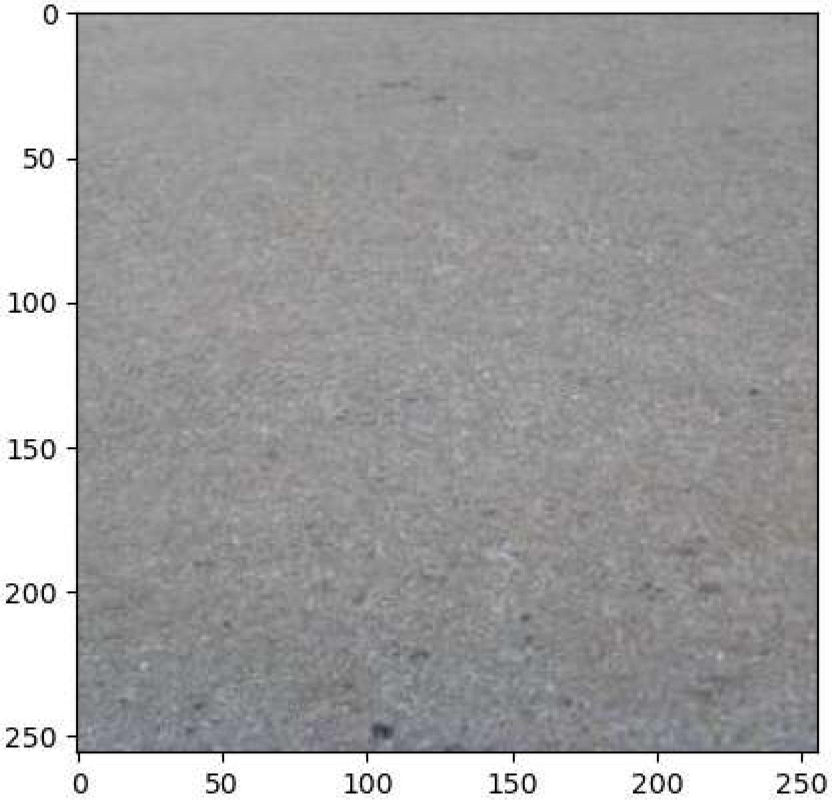
0
50
100
150
200
250
0
50
100
150
200
250

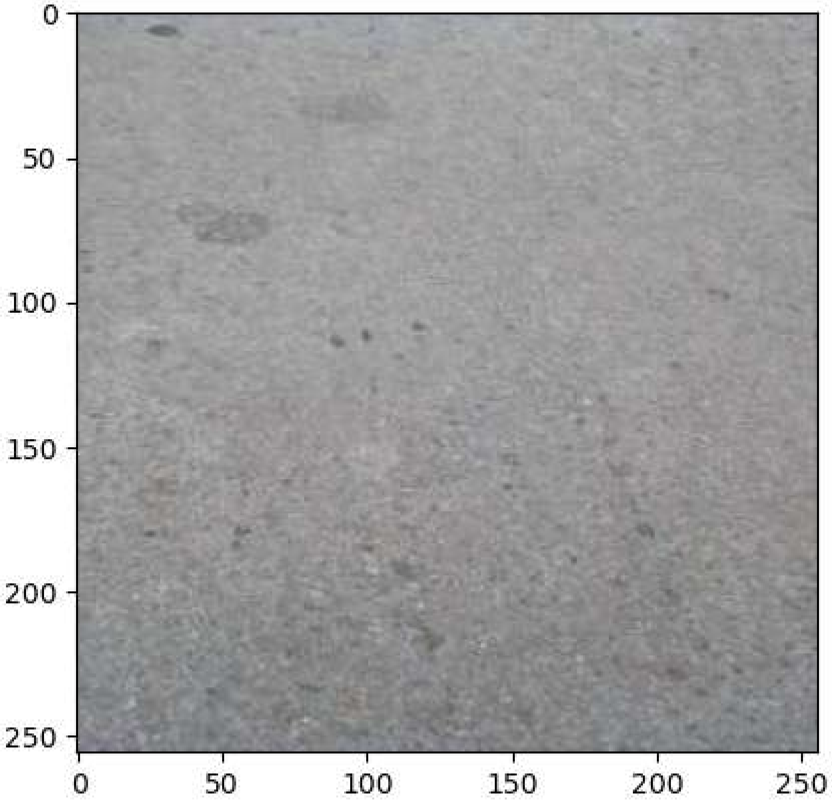
0
50
100
150
200
250
0
50
100
150
200
250

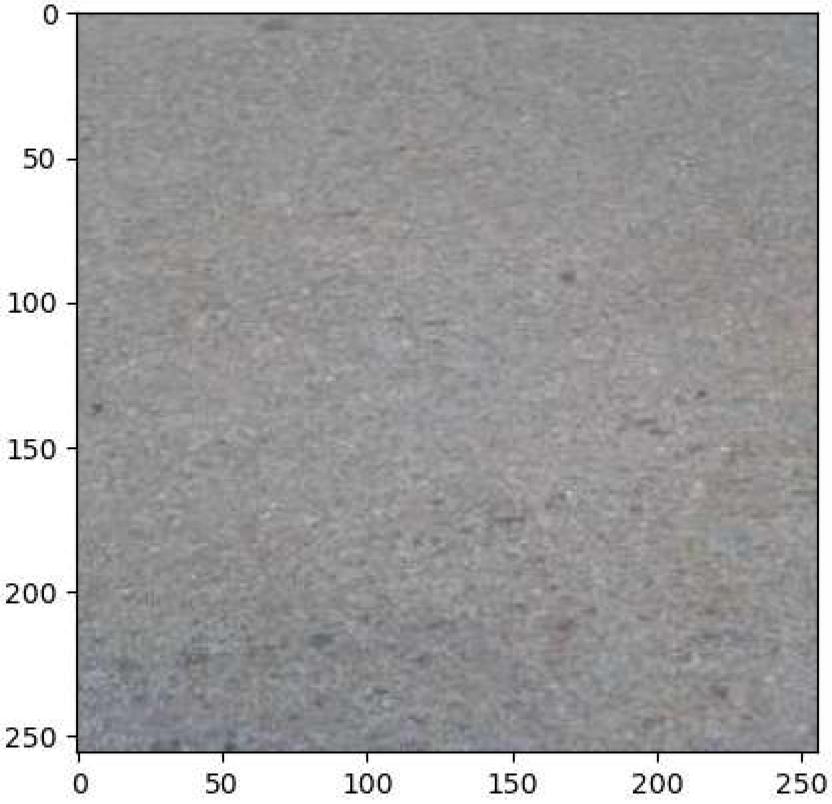
0
50
100
150
200
250
0
50
100
150
200
250

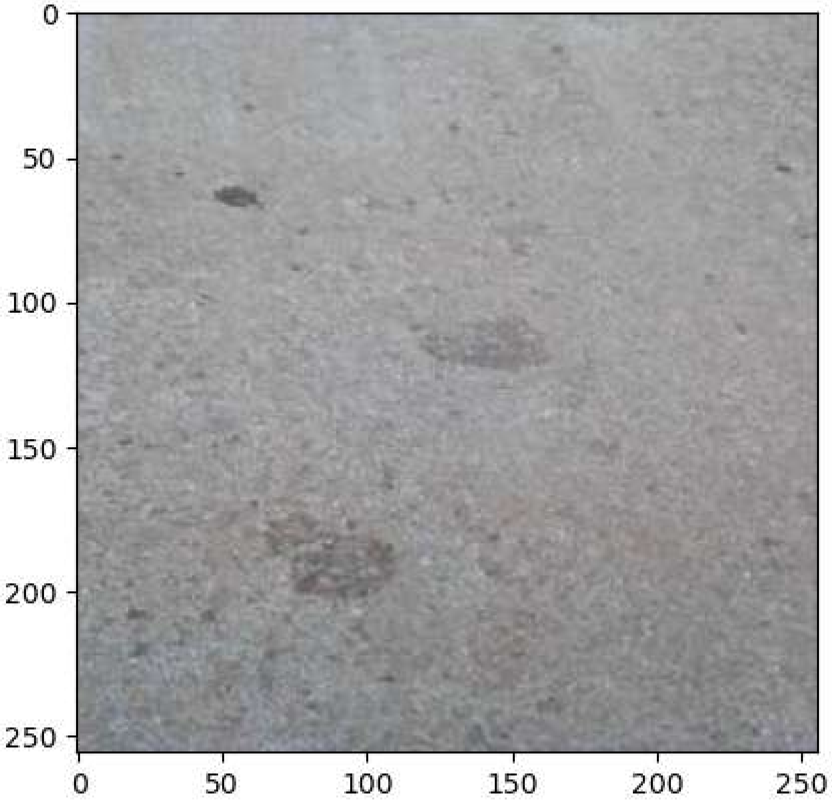
0
50
100
150
200
250
0
50
100
150
200
250

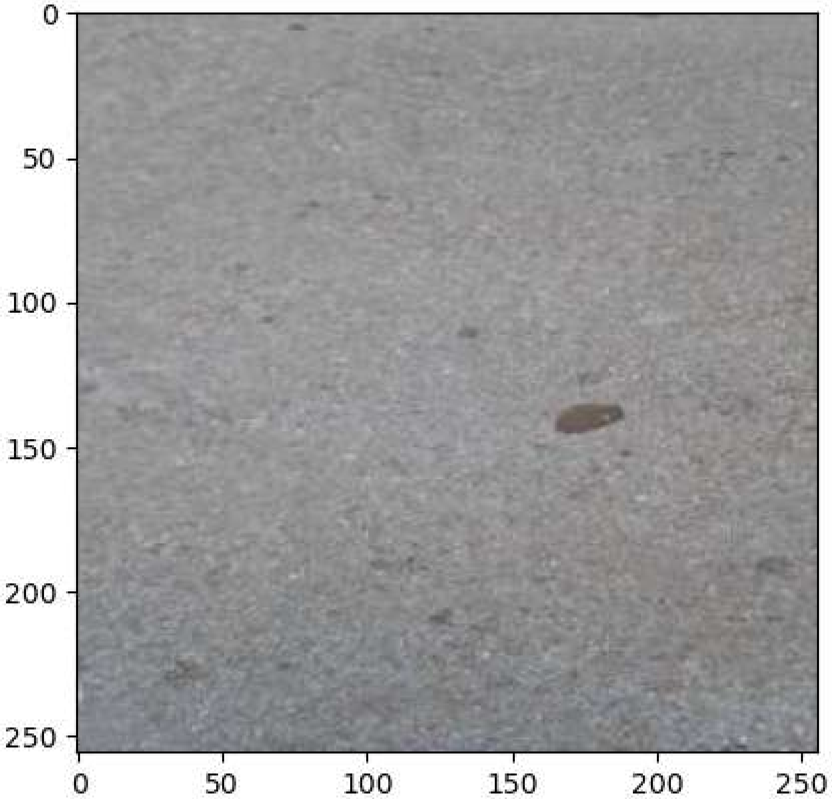
0
50
100
150
200
250
0
50
100
150
200
250

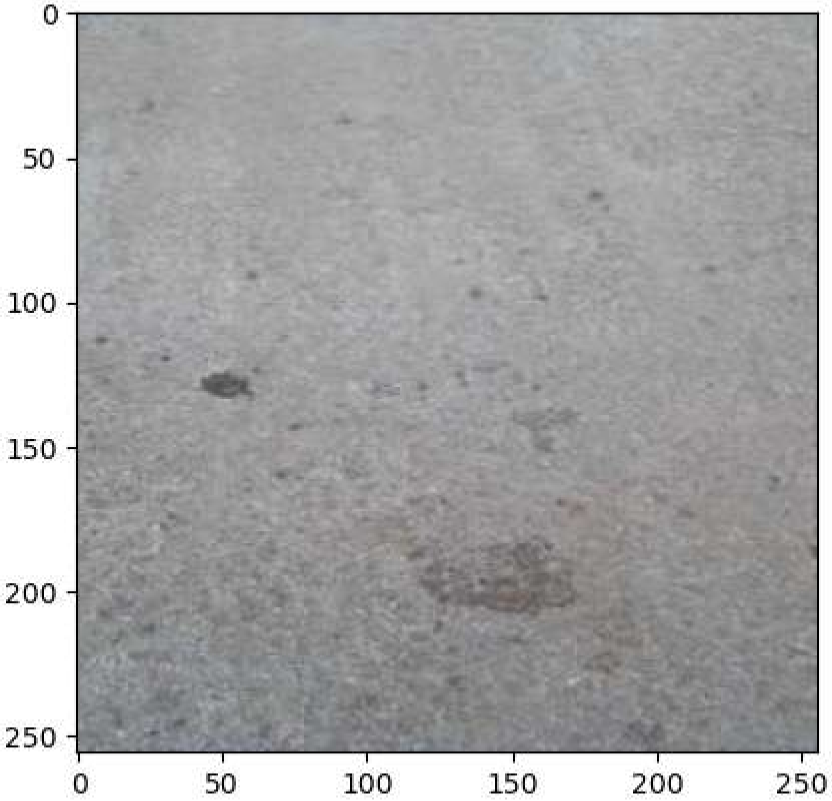
0
50
100
150
200
250
0
50
100
150
200
250

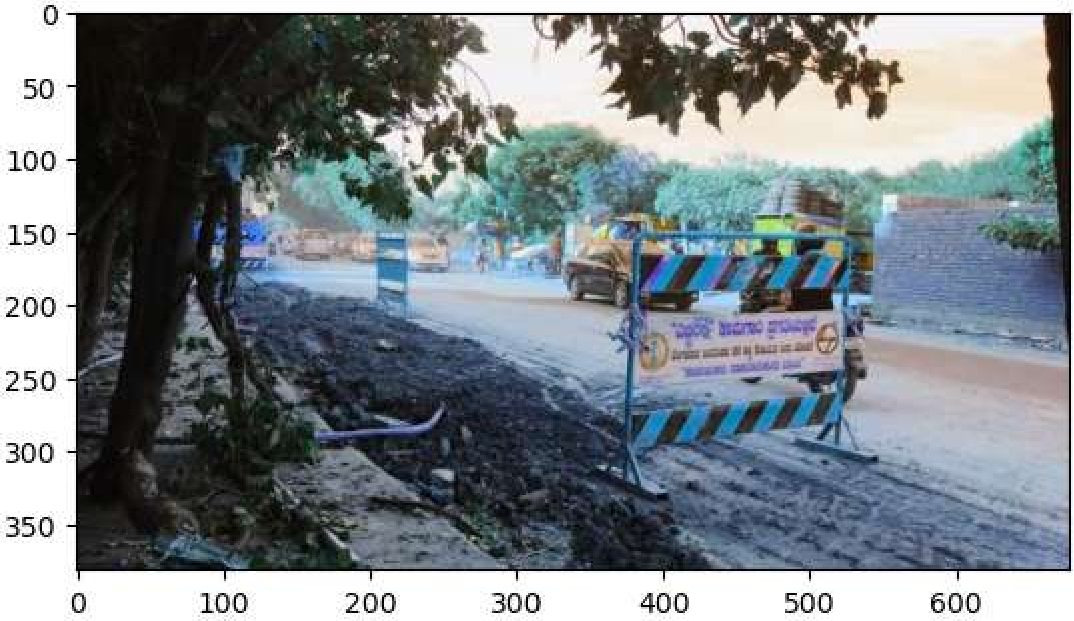
0
50
100
150
200
250
300
350
0 100 200 300 400 500 600

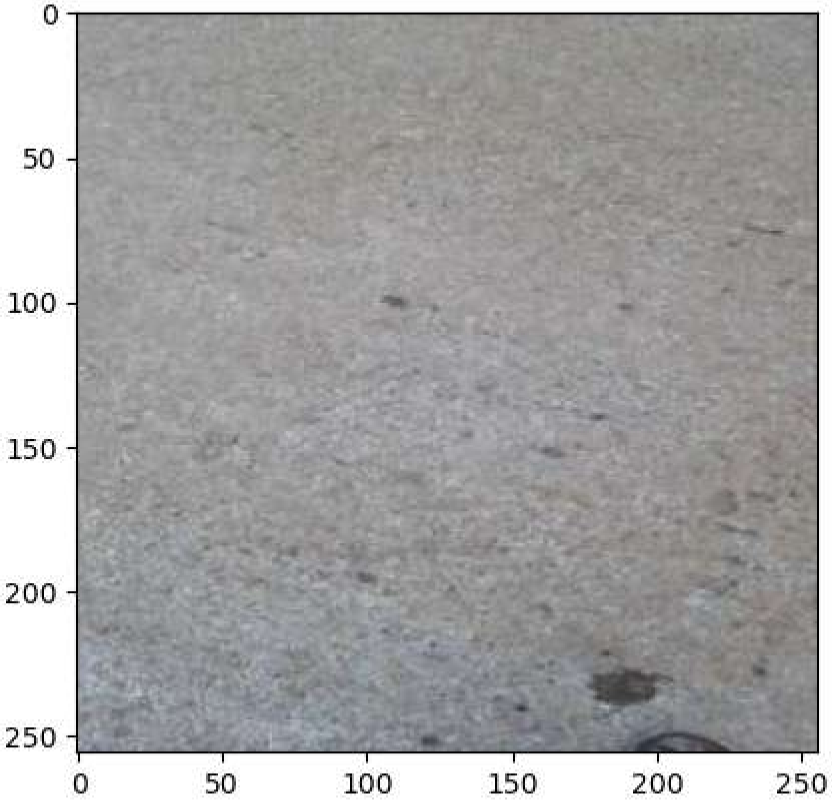
0
50
100
150
200
250
0 50 100 150 200 250

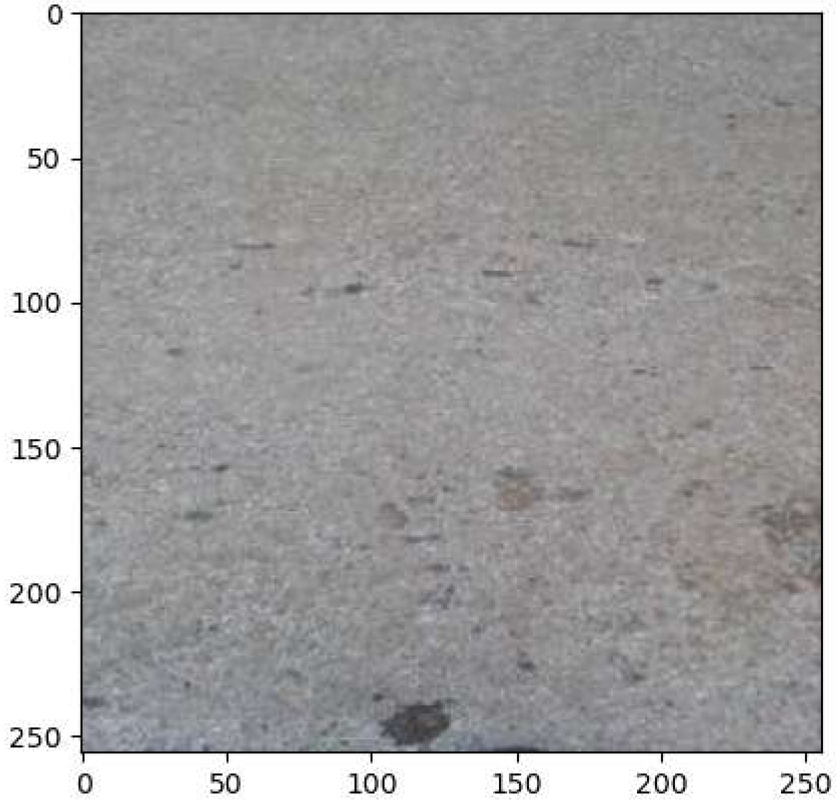
0
50
100
150
200
250
0
50
100
150
200
250

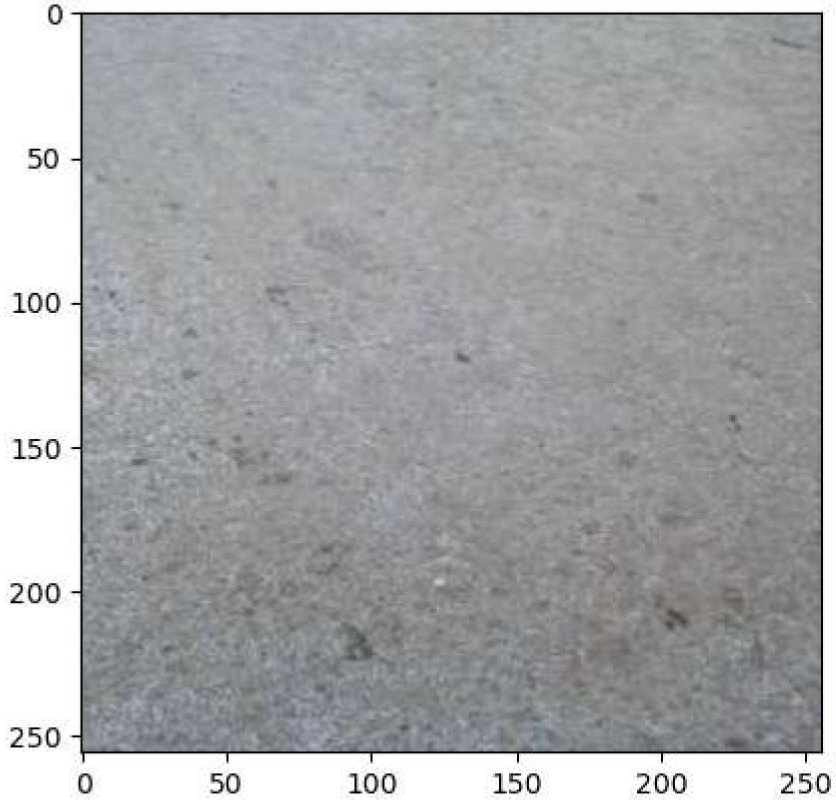
0
50
100
150
200
250
0
50
100
150
200
250

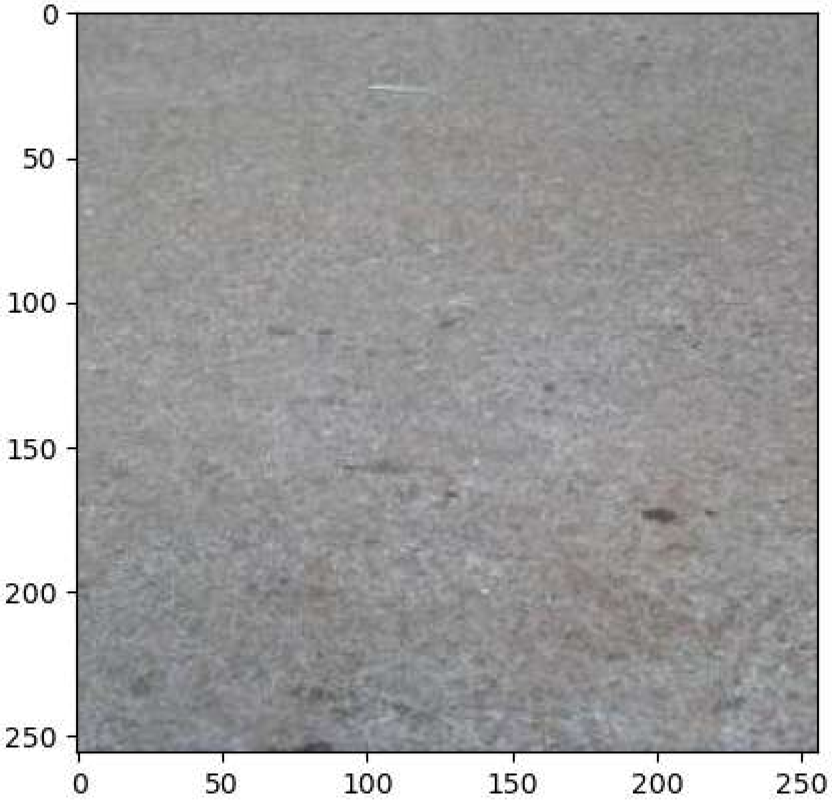
0
50
100
150
200
250
0
50
100
150
200
250

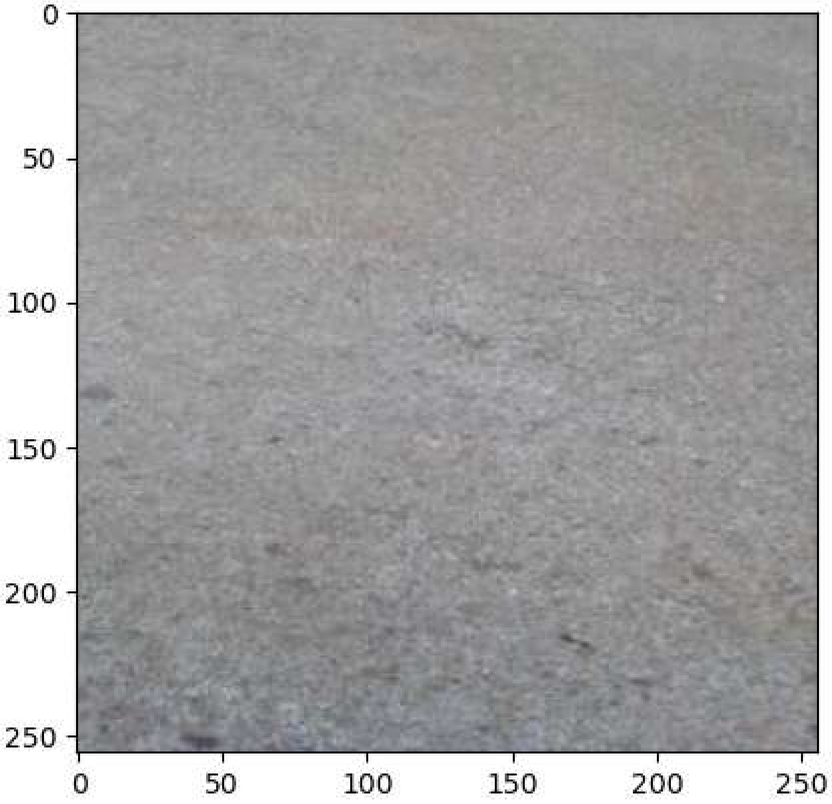
0
50
100
150
200
250
0
50
100
150
200
250

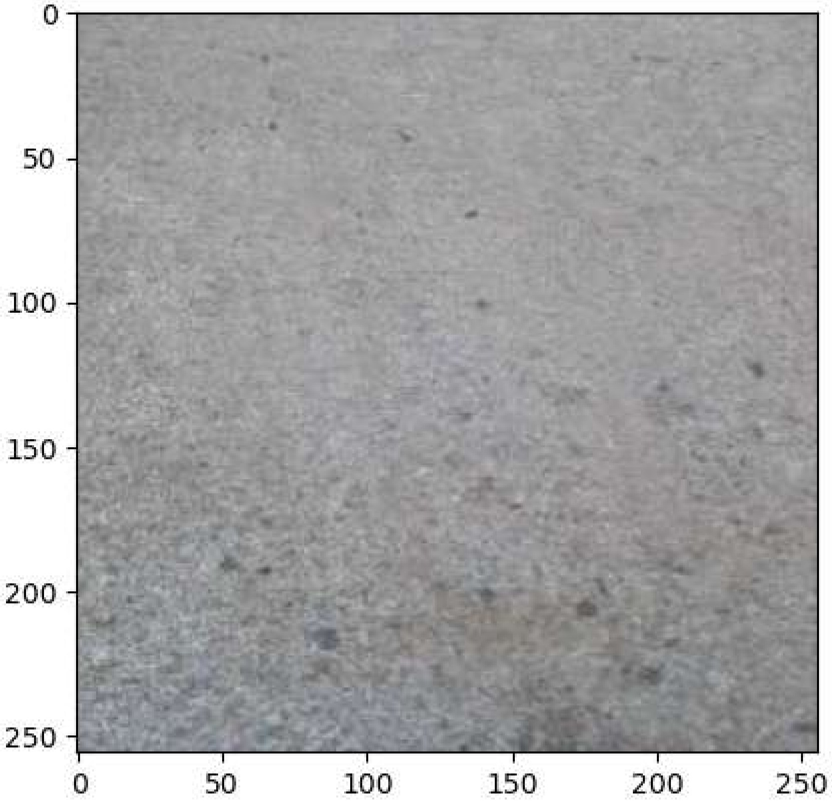
0
50
100
150
200
250
0 50 100 150 200 250

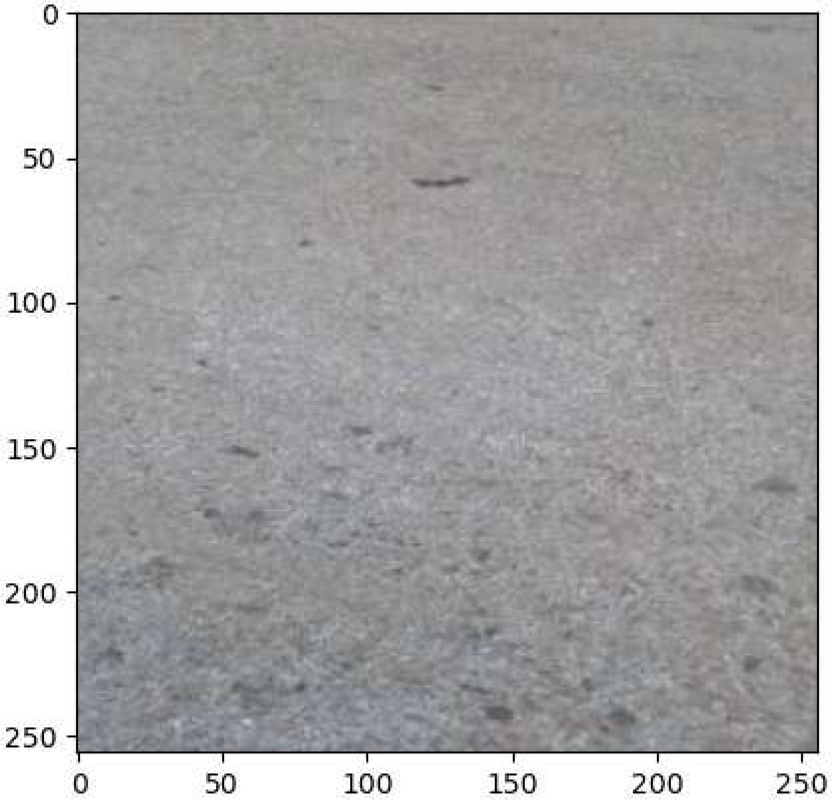
0
50
100
150
200
250
0 50 100 150 200 250

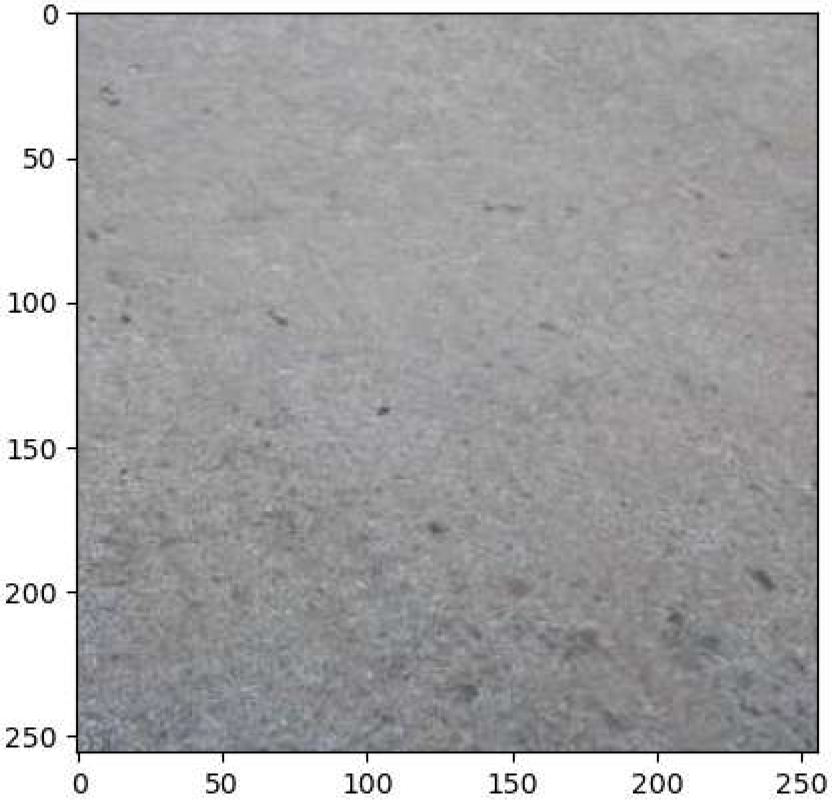
0
50
100
150
200
250
0 50 100 150 200 250

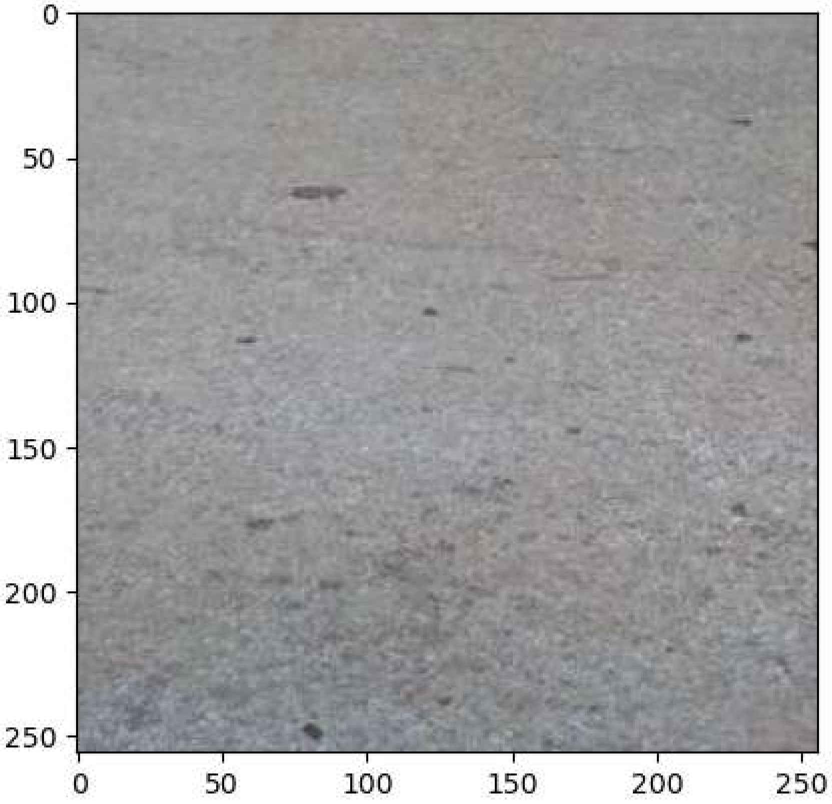
0
50
100
150
200
250
0 50 100 150 200 250

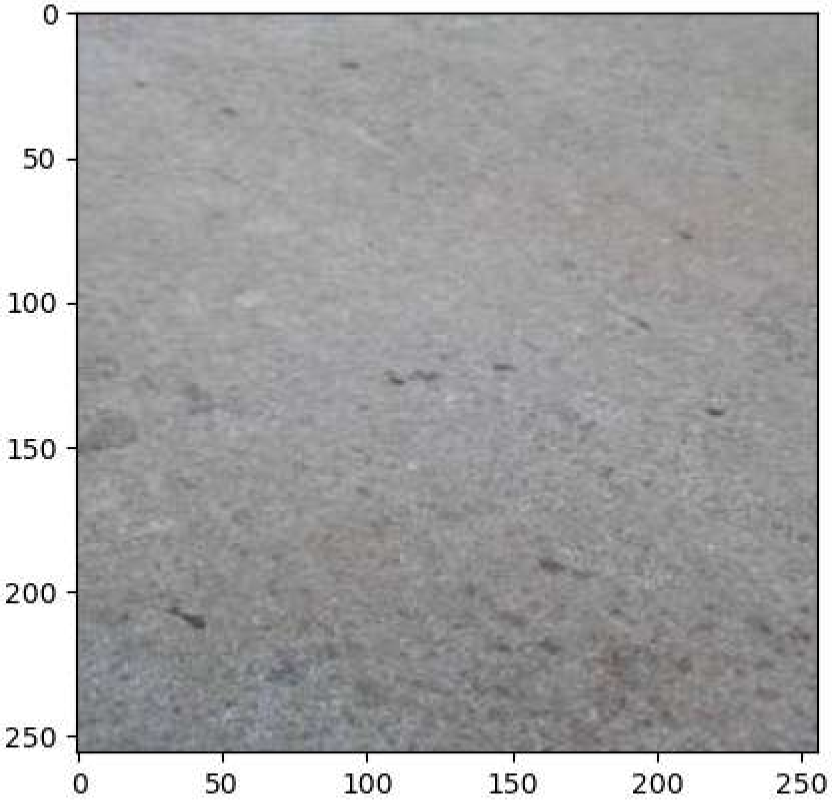
0
50
100
150
200
250
0
50
100
150
200
250

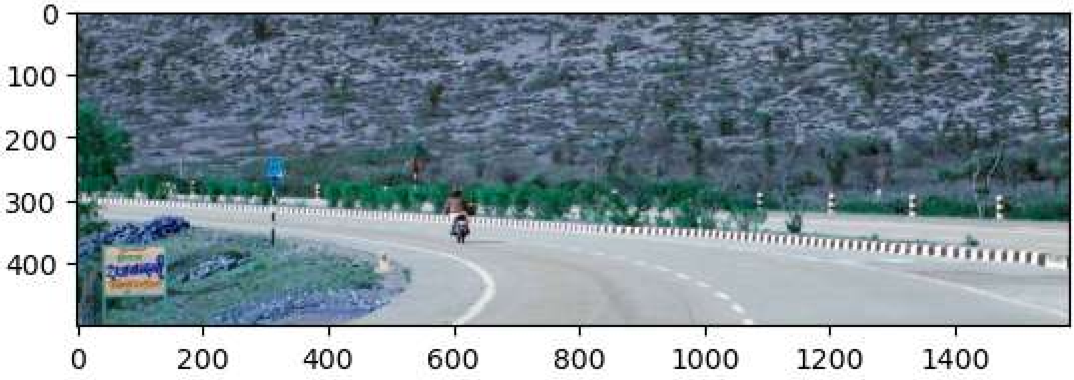
0
100
200
300
400
0
200
400
600
800
1000
1200
1400

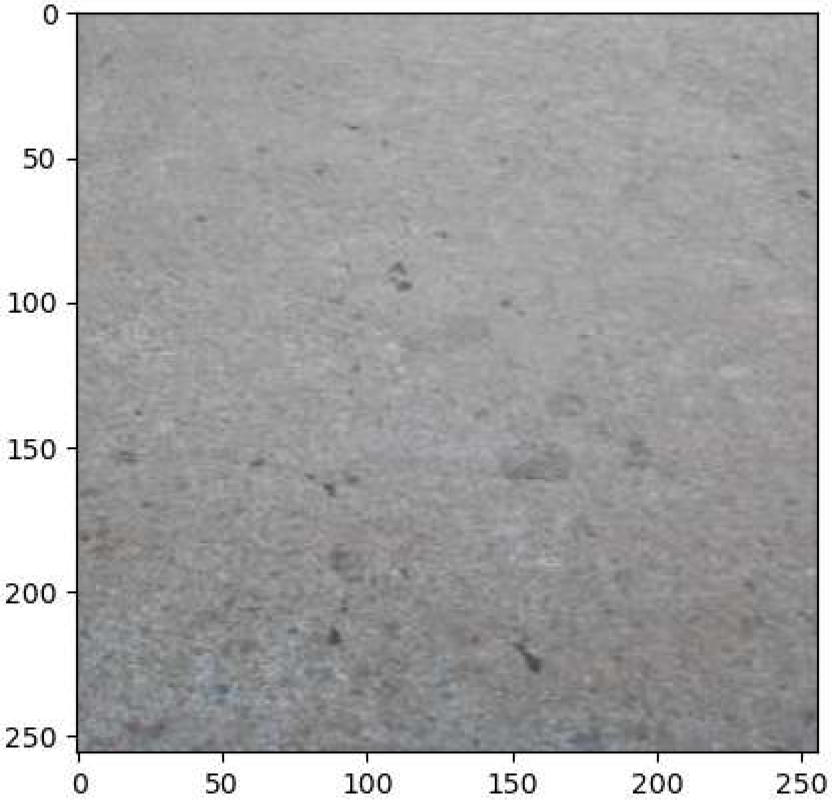
0
50
100
150
200
250
0
50
100
150
200
250

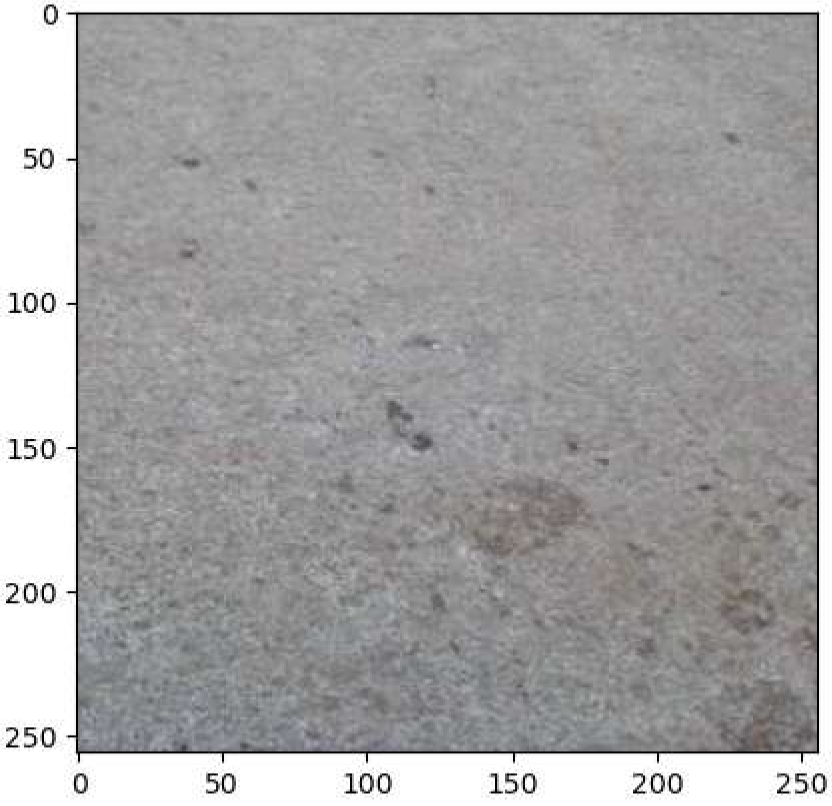
0
50
100
150
200
250
0
50
100
150
200
250

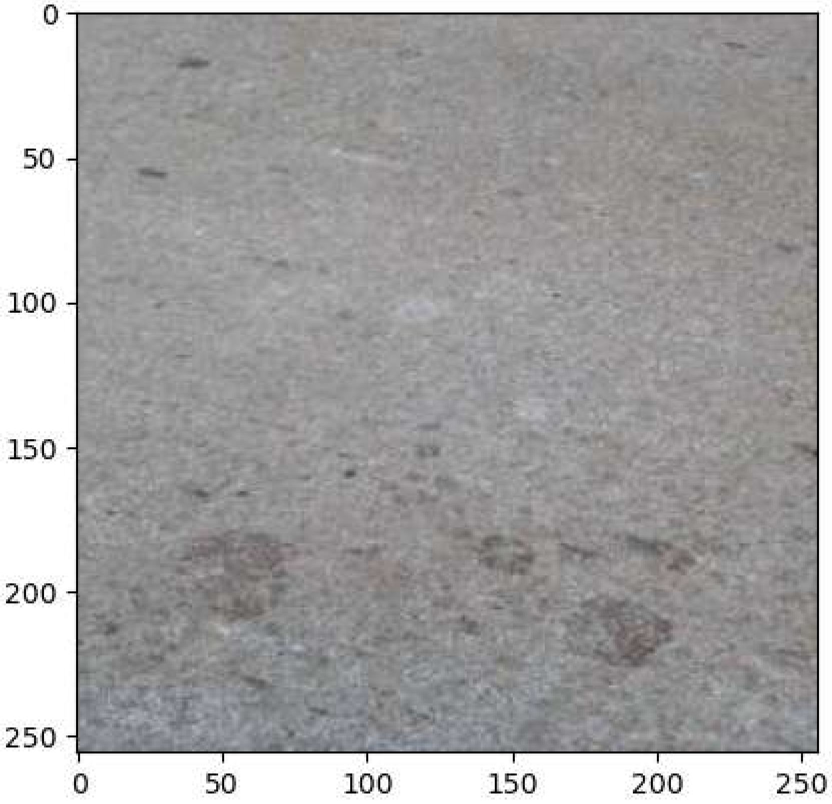
0
50
100
150
200
250
0
50
100
150
200
250

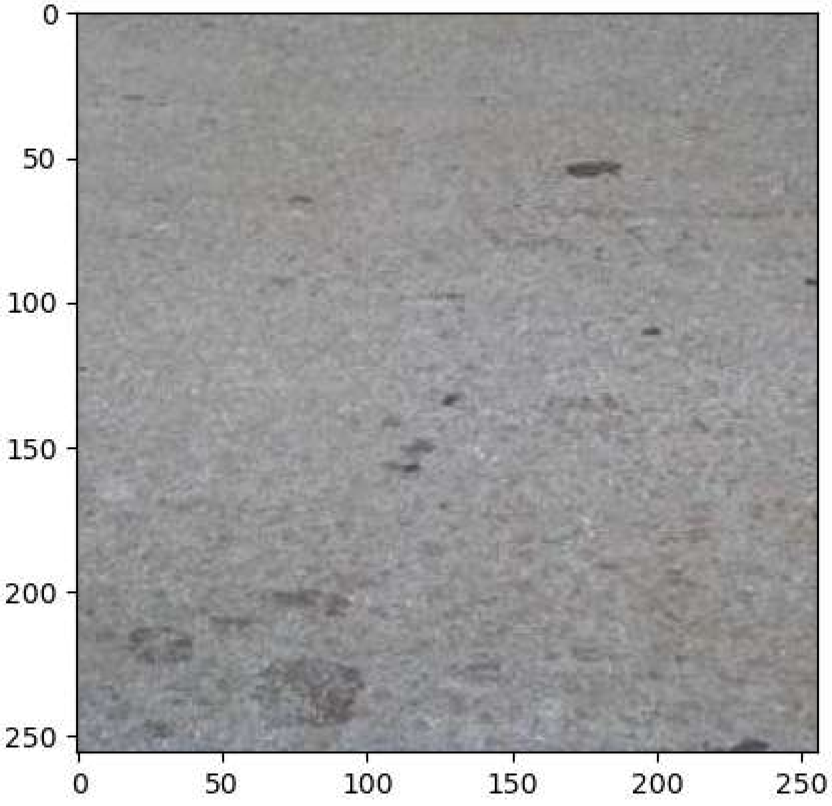
0
50
100
150
200
250
0
50
100
150
200
250

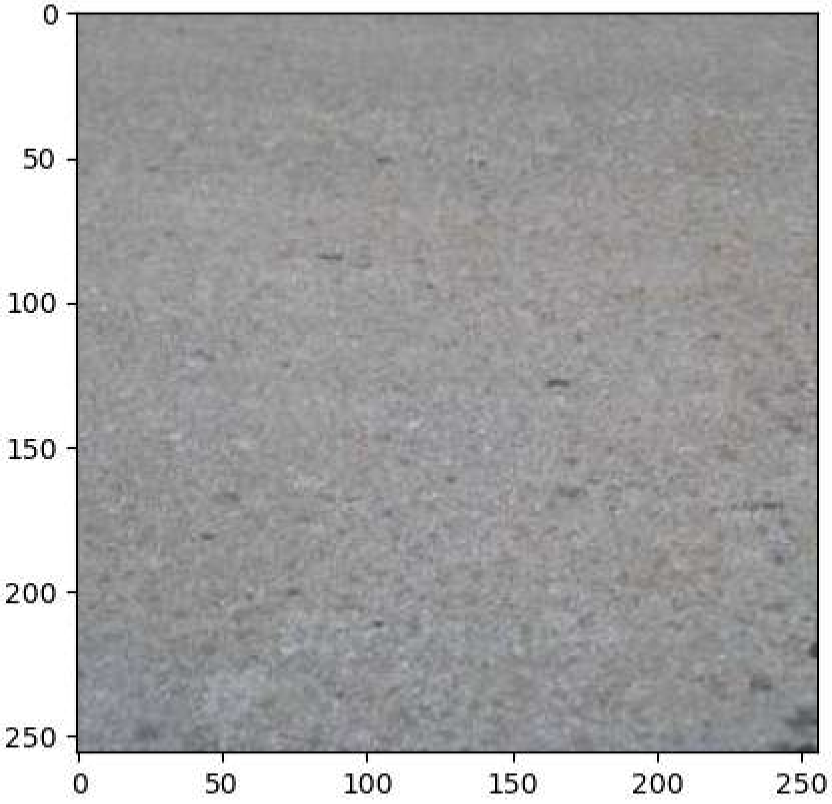
0
50
100
150
200
250
0
50
100
150
200
250

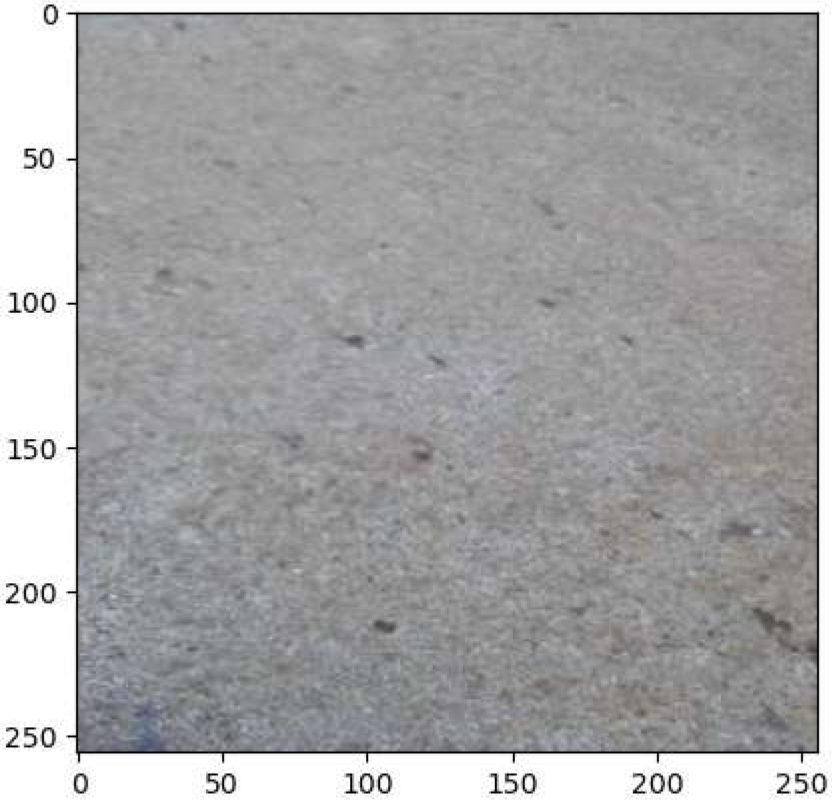
0
50
100
150
200
250
0
50
100
150
200
250

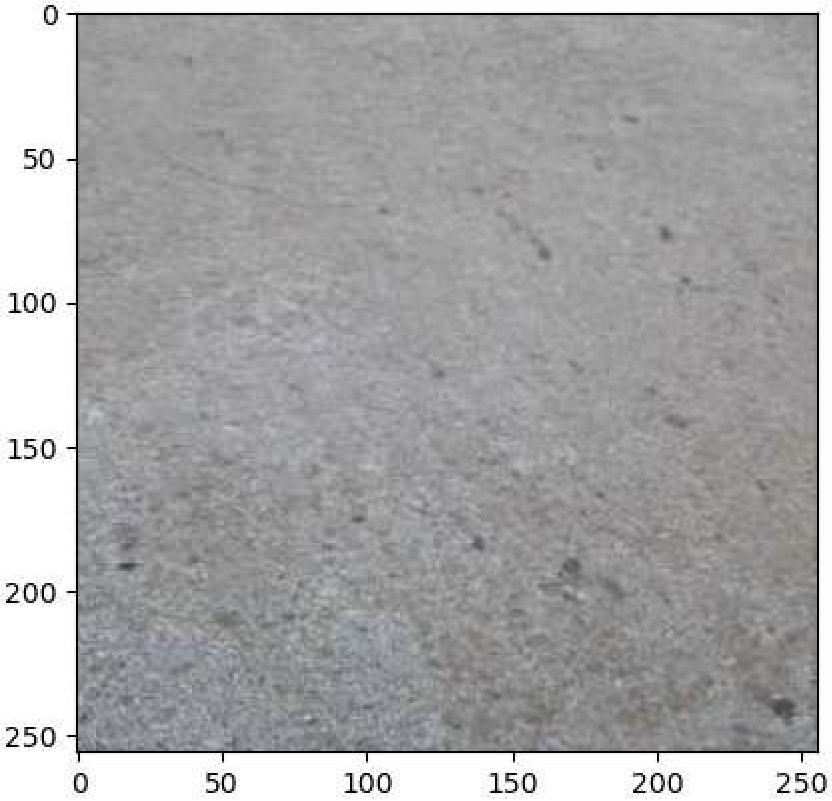
0
50
100
150
200
250
0 50 100 150 200 250

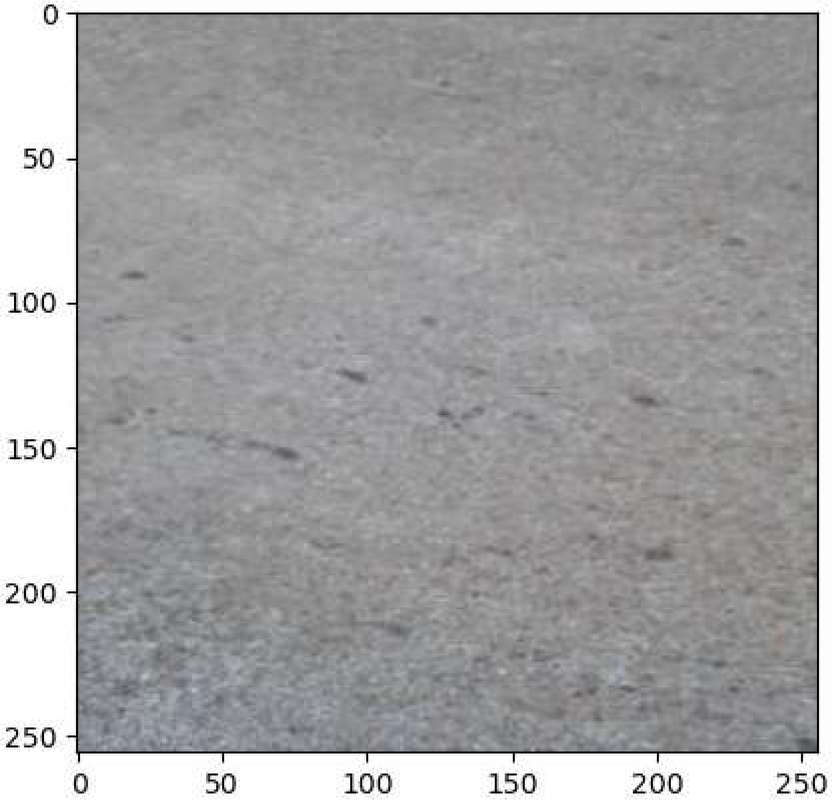
0
50
100
150
200
250
0 50 100 150 200 250

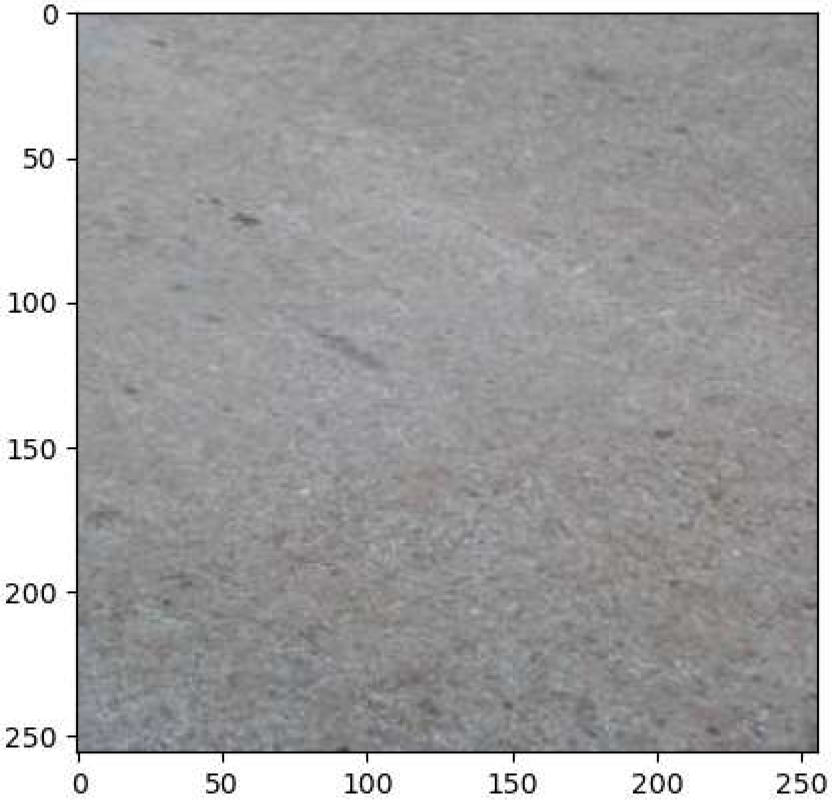

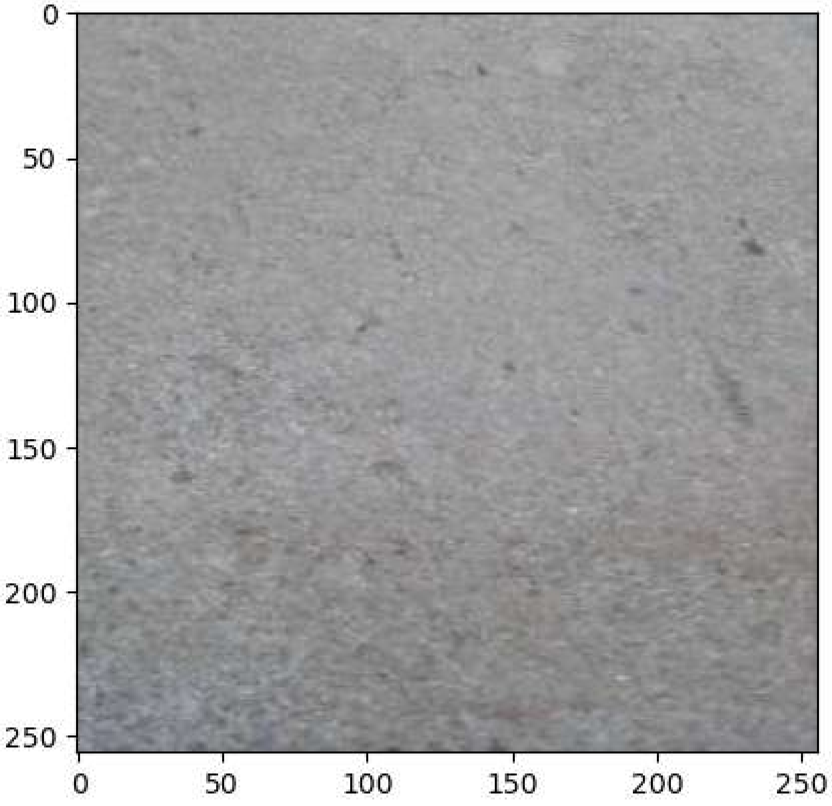

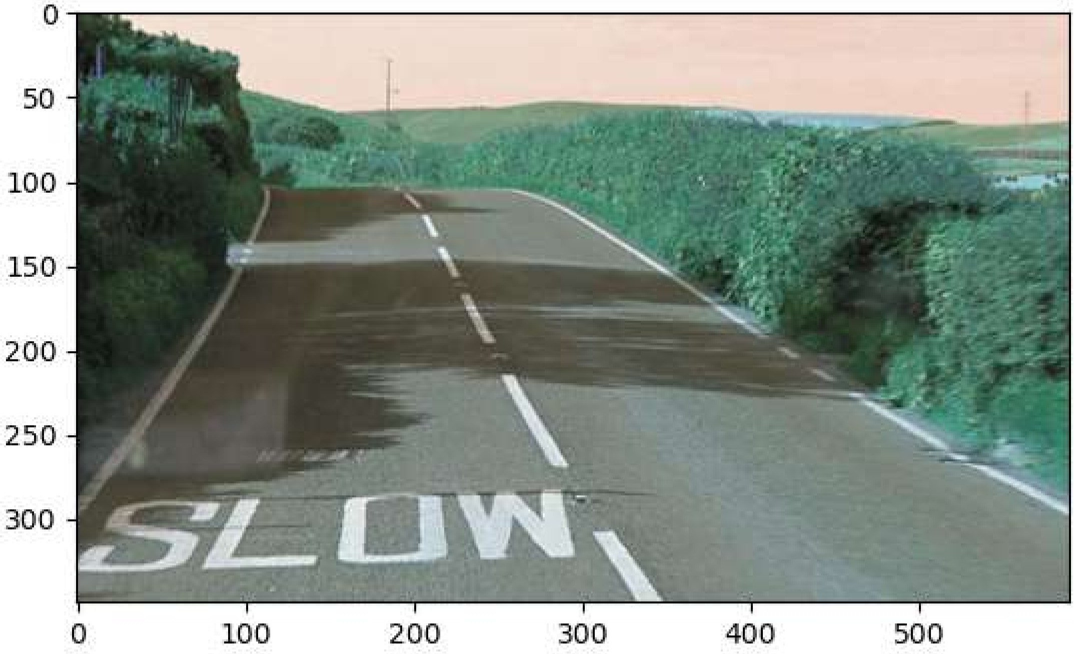
0
50
100
150
200
250
300
SLOW
0 100 200 300 400 500

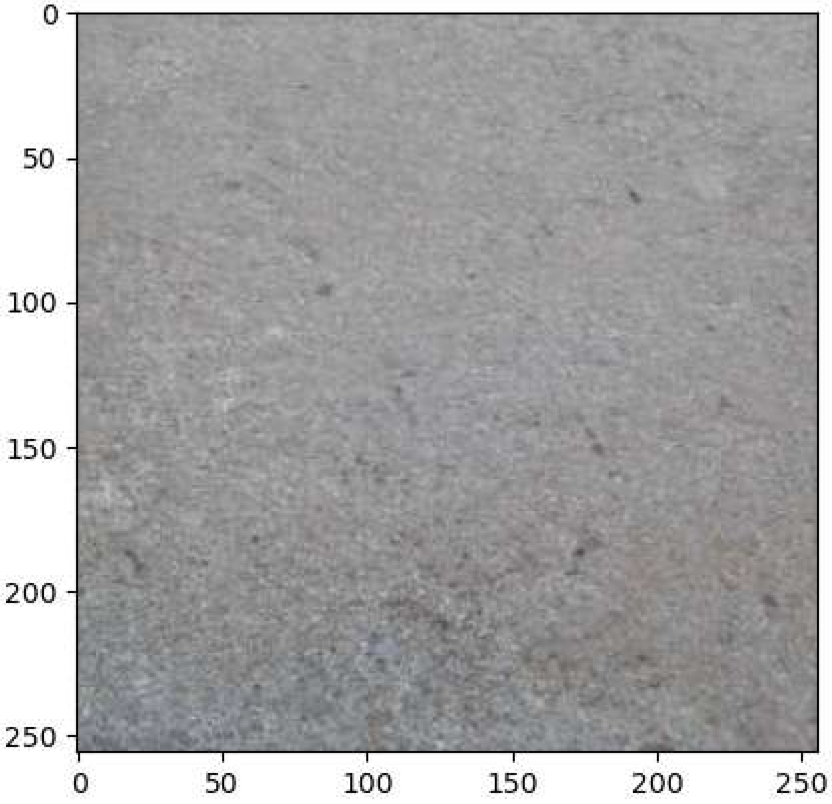
0
50
100
150
200
250
0 50 100 150 200 250

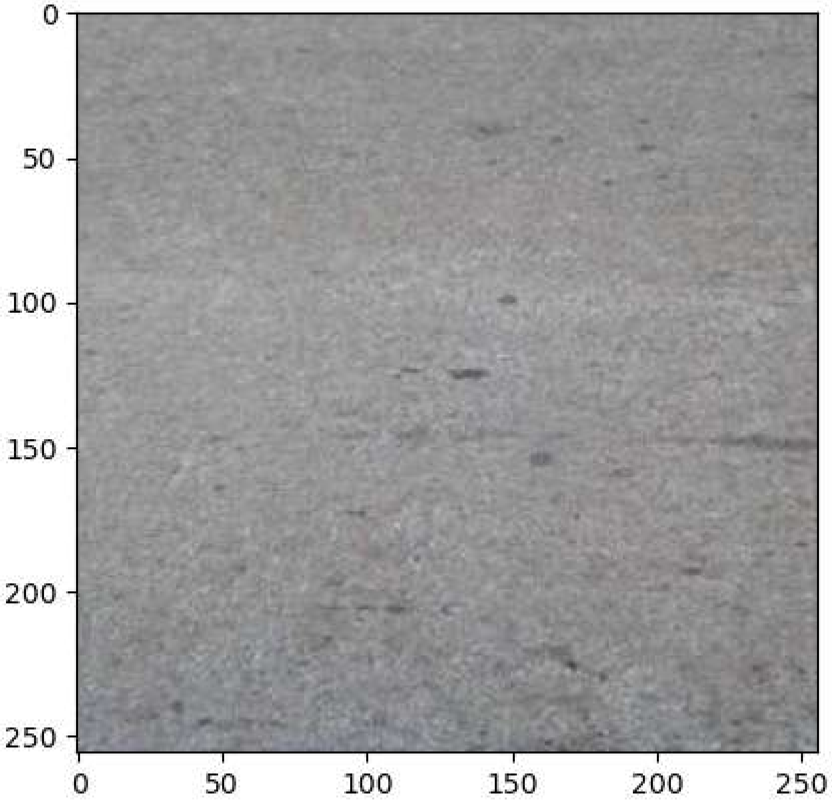
0
50
100
150
200
250
0 50 100 150 200 250

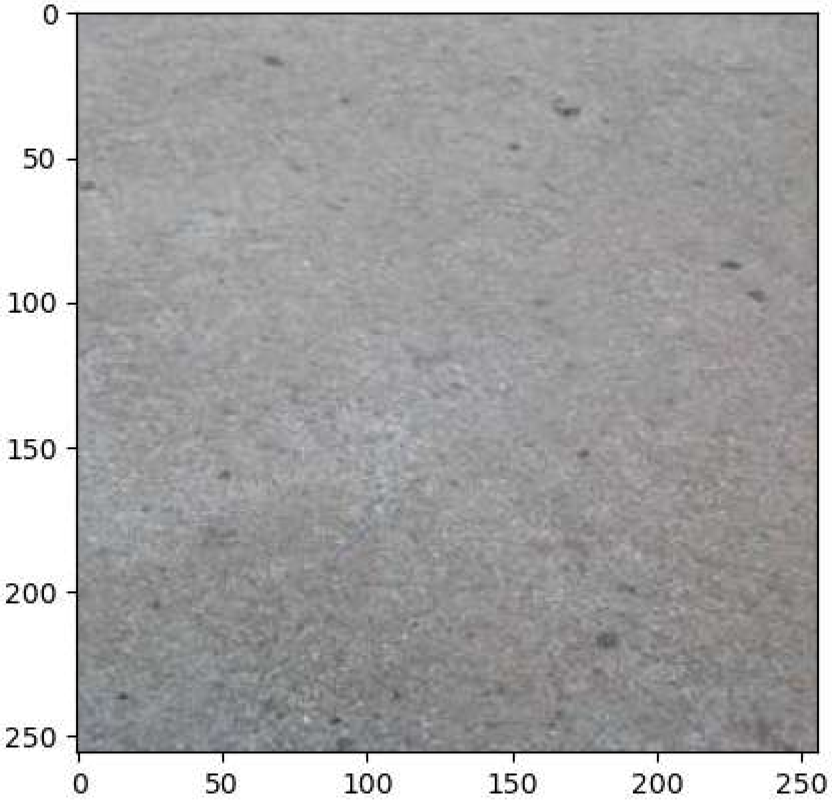
0
50
100
150
200
250
0 50 100 150 200 250

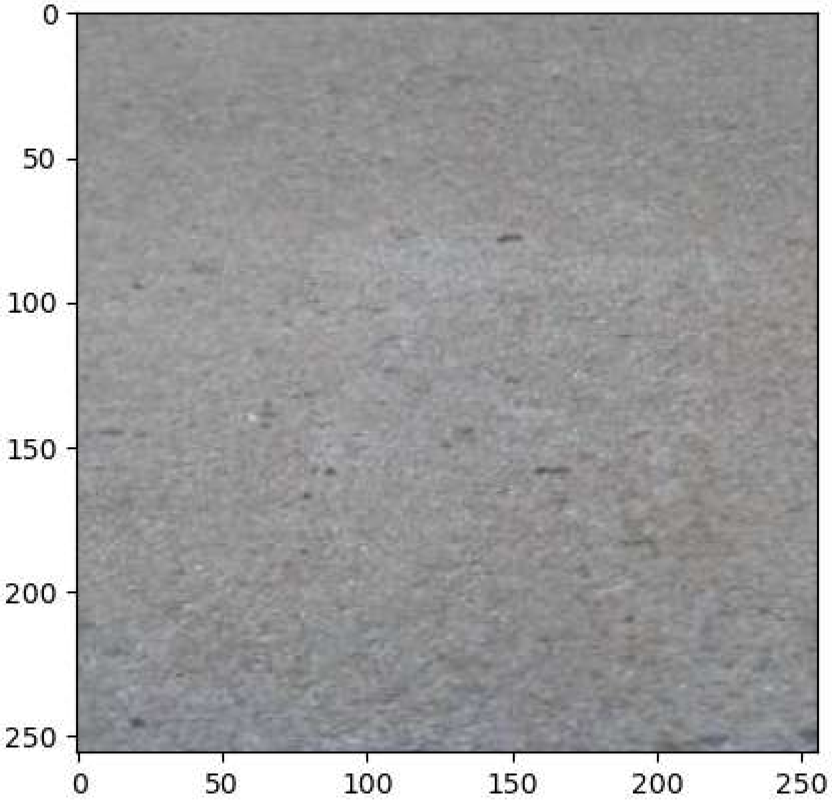

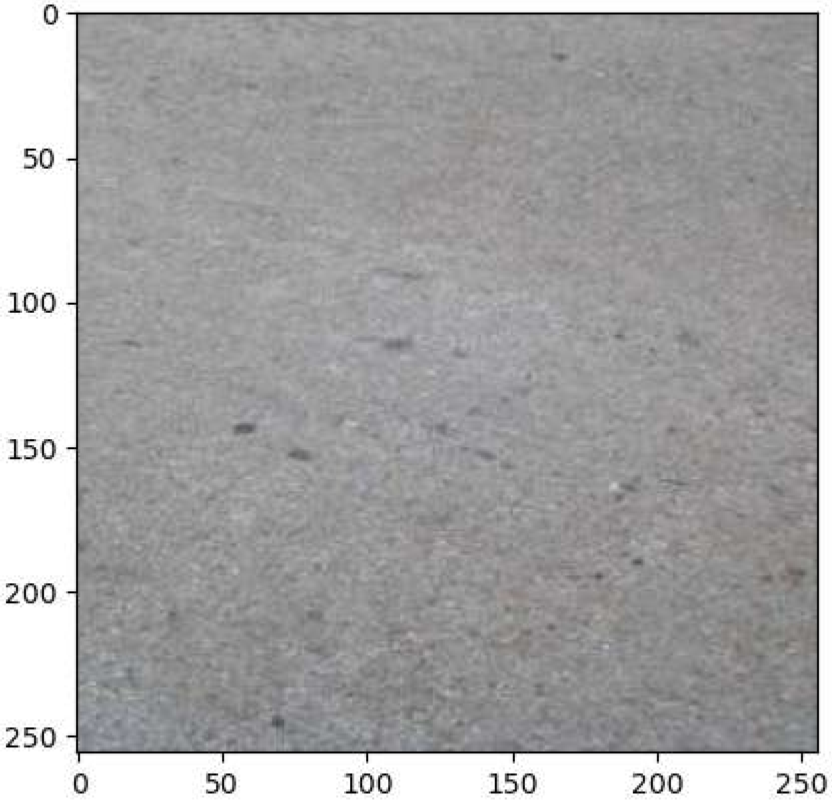

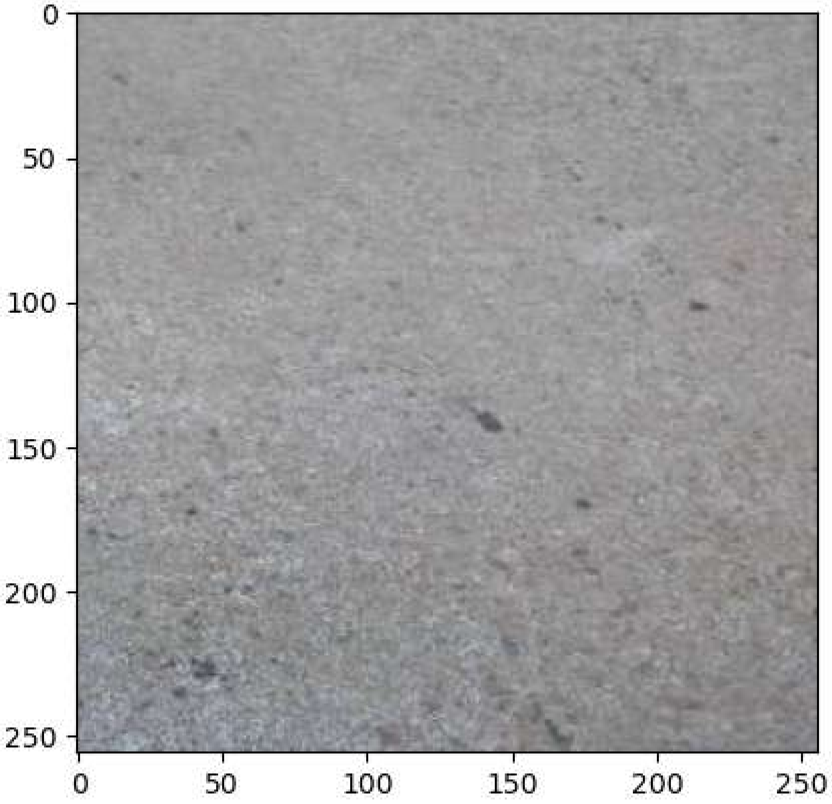
0
50
100
150
200
250
0
50
100
150
200
250

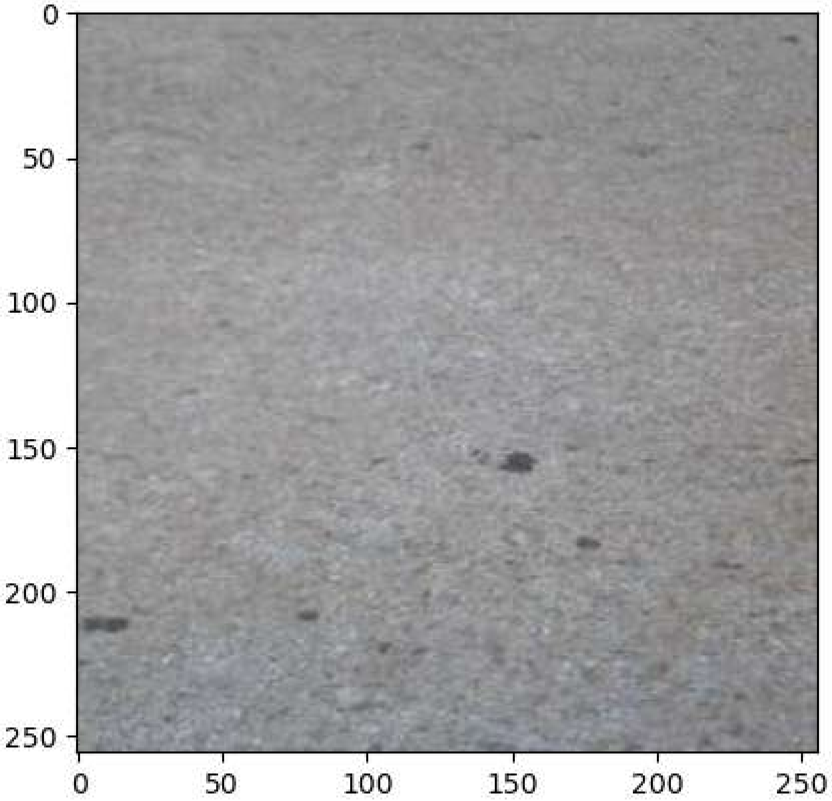
0
50
100
150
200
250
0
50
100
150
200
250

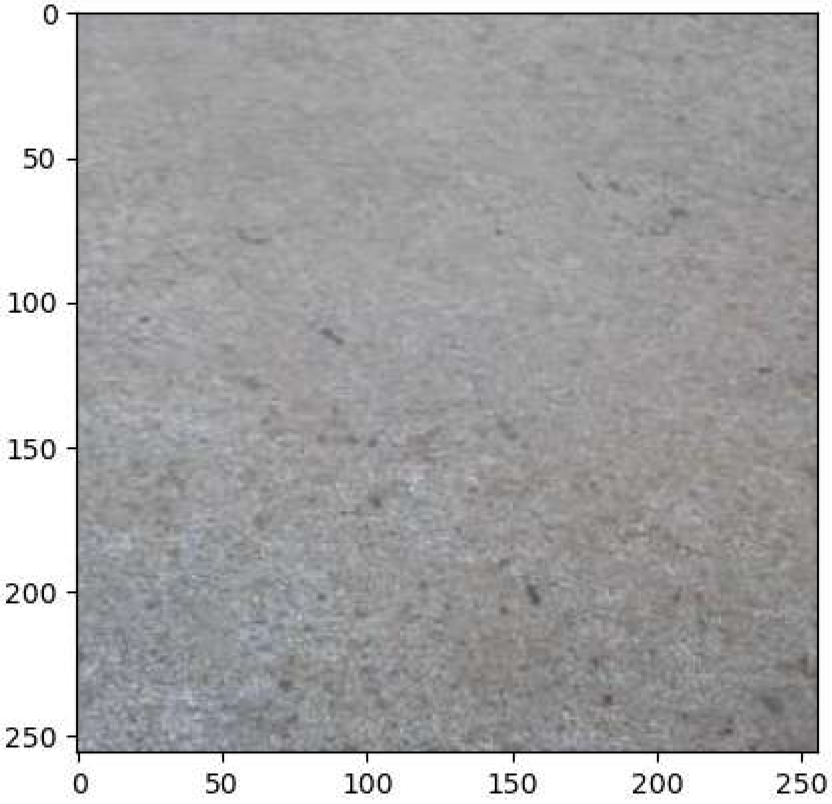
0
50
100
150
200
250
0
50
100
150
200
250

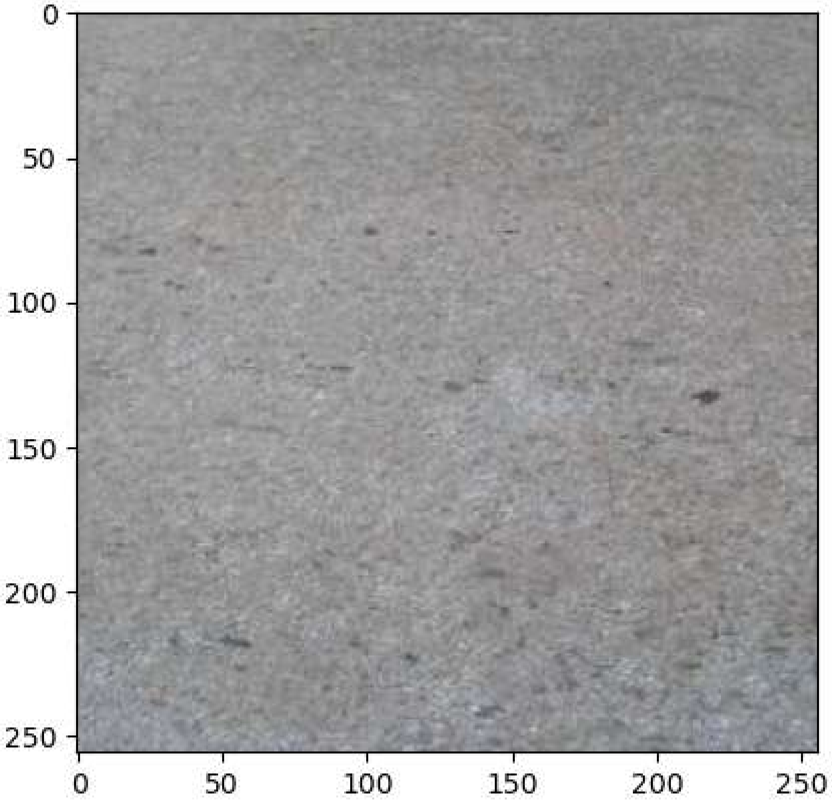
0
50
100
150
200
250
0
50
100
150
200
250

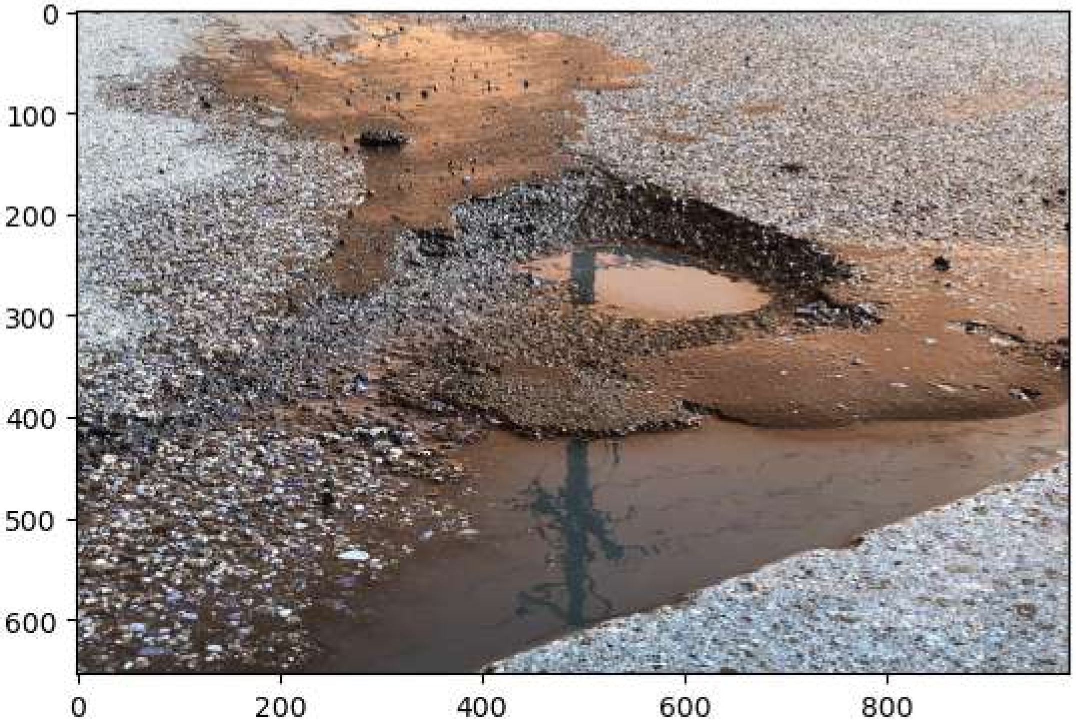
0
100
200
300
400
500
600
0
200
400
600
800

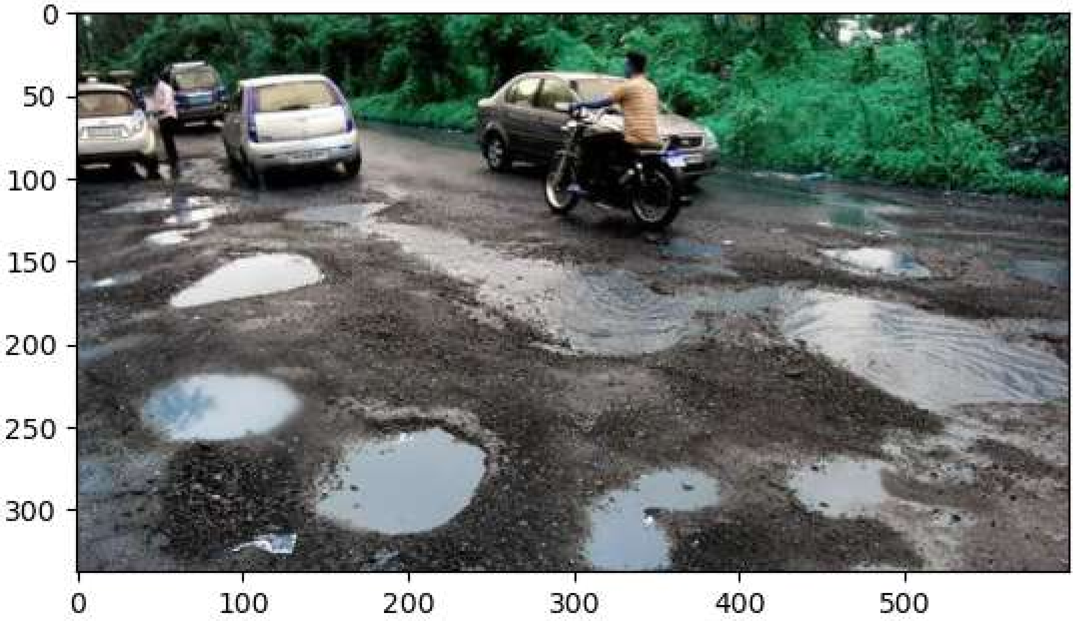
0
50
100
150
200
250
300
0
100
200
300
400
500

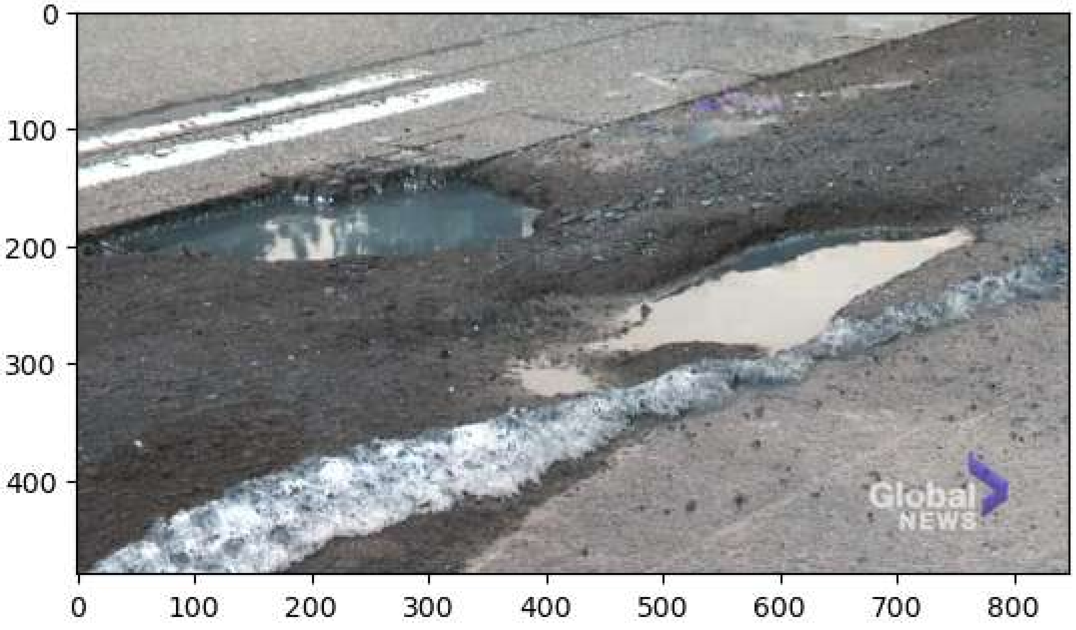
0
100
200
300
400
Global
NEWS
0
100
200
300
400
500
600
700
800

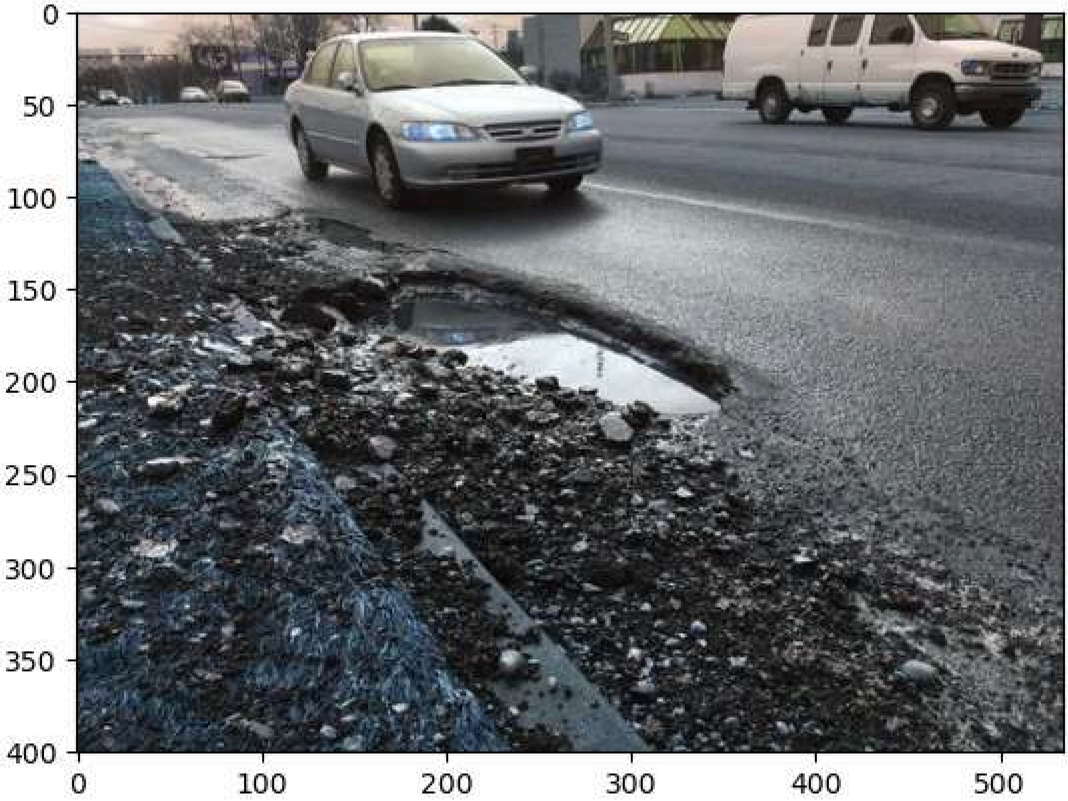
0
50
100
150
200
250
300
350
400
0 100 200 300 400 500

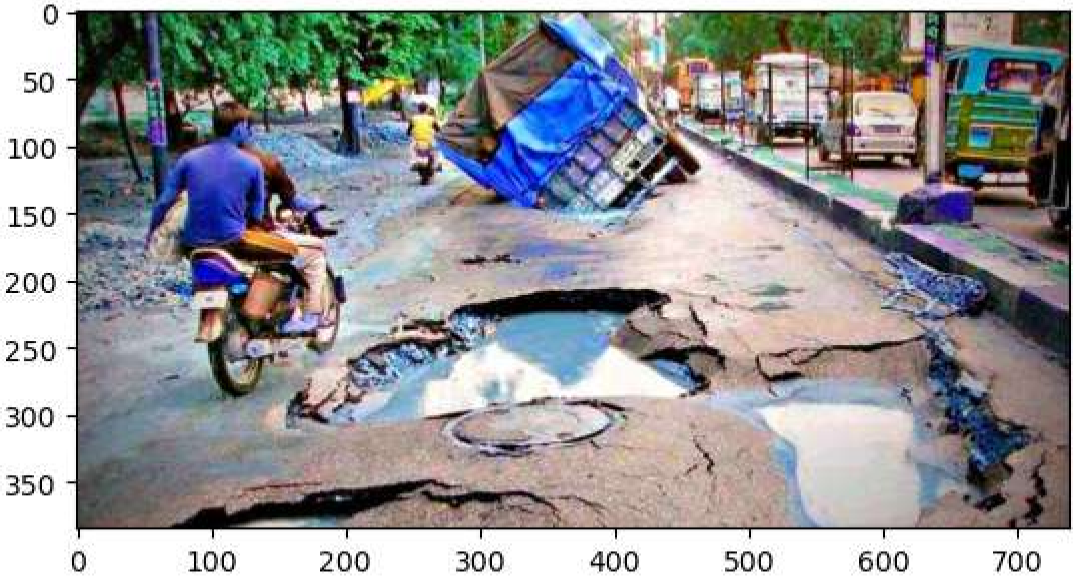
0
50
100
150
200
250
300
350
0 100 200 300 400 500 600 700

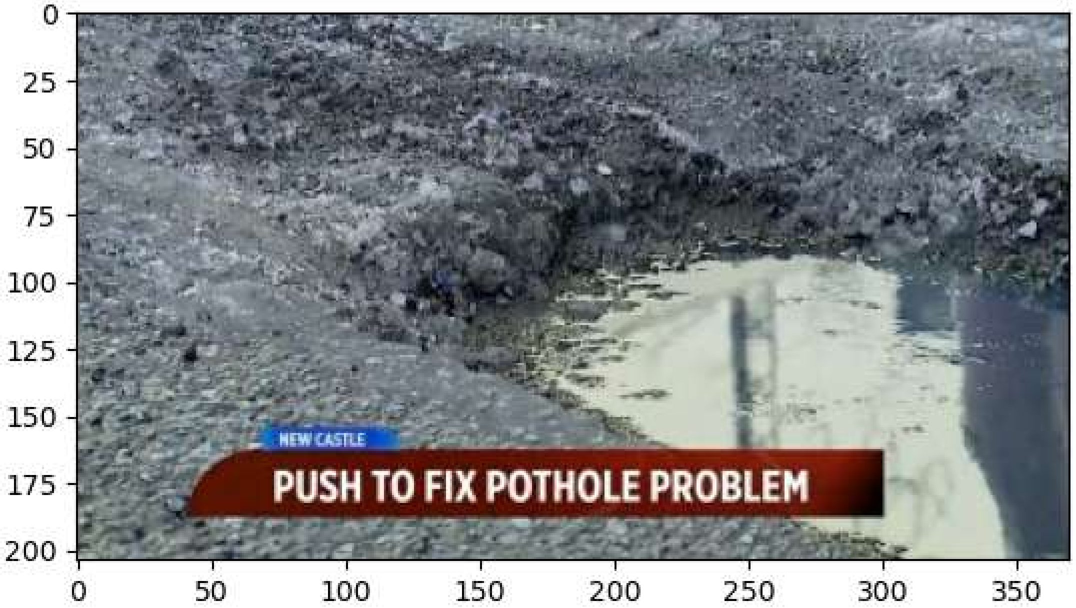
0
25
50
75
100
125
150
175
200
NEW CASTLE
PUSH TO FIX POTHOLE PROBLEM
0
50
100
150
200
250
300
350

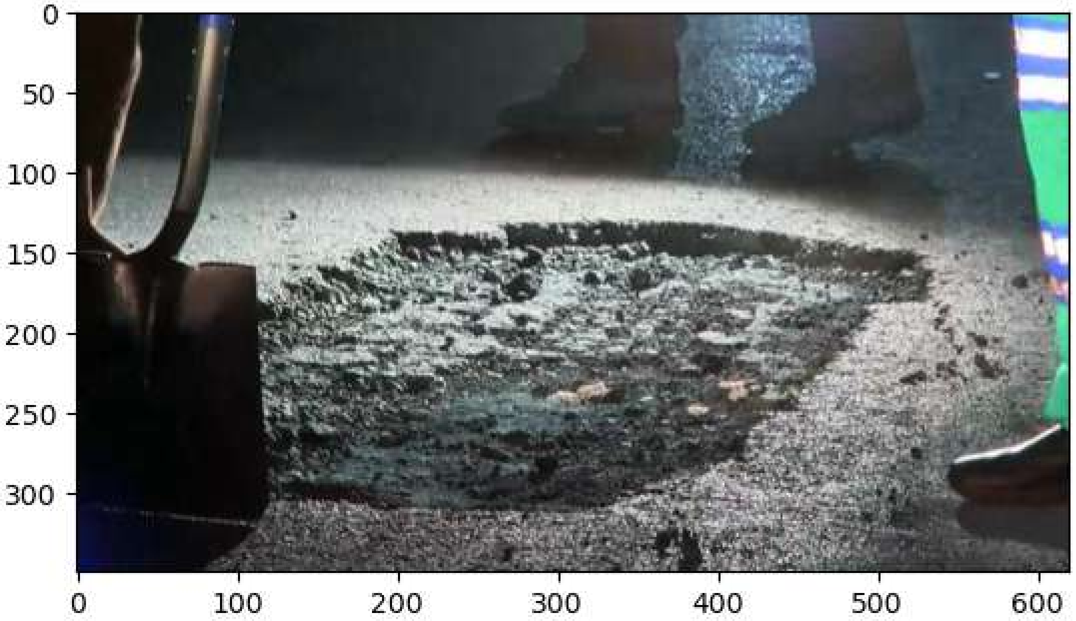
0
50
100
150
200
250
300
0
100
200
300
400
500
600

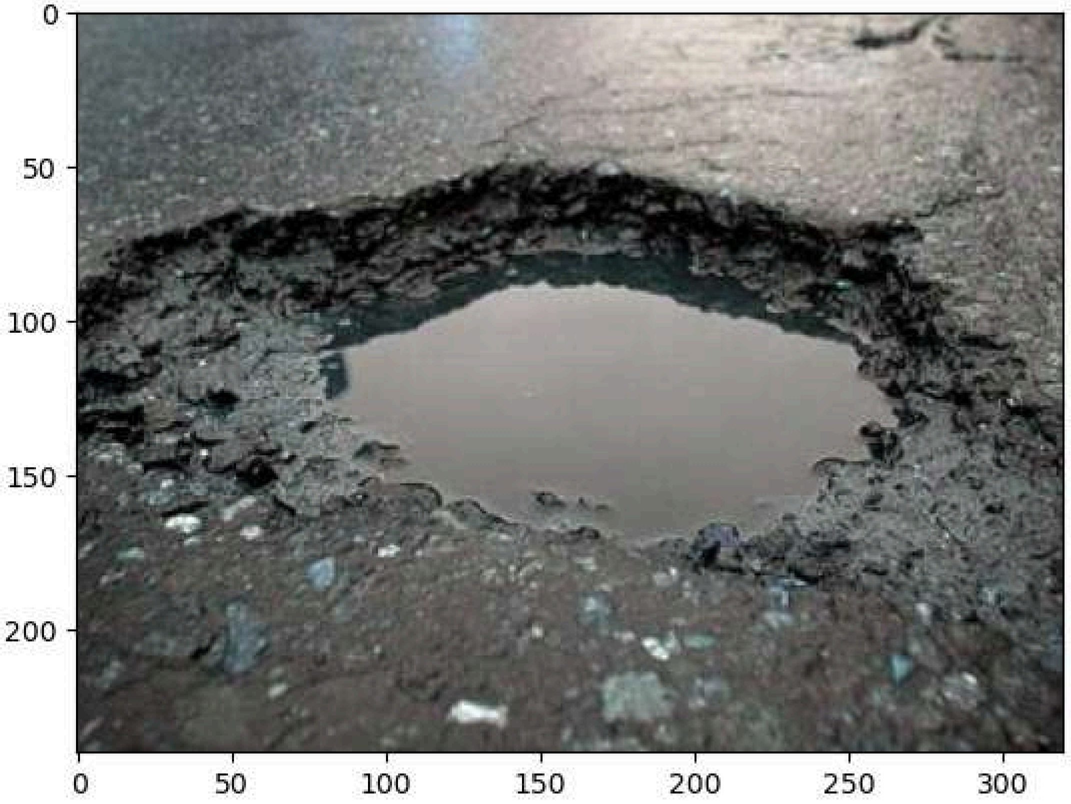
0
50
100
150
200
0
50
100
150
200
250
300

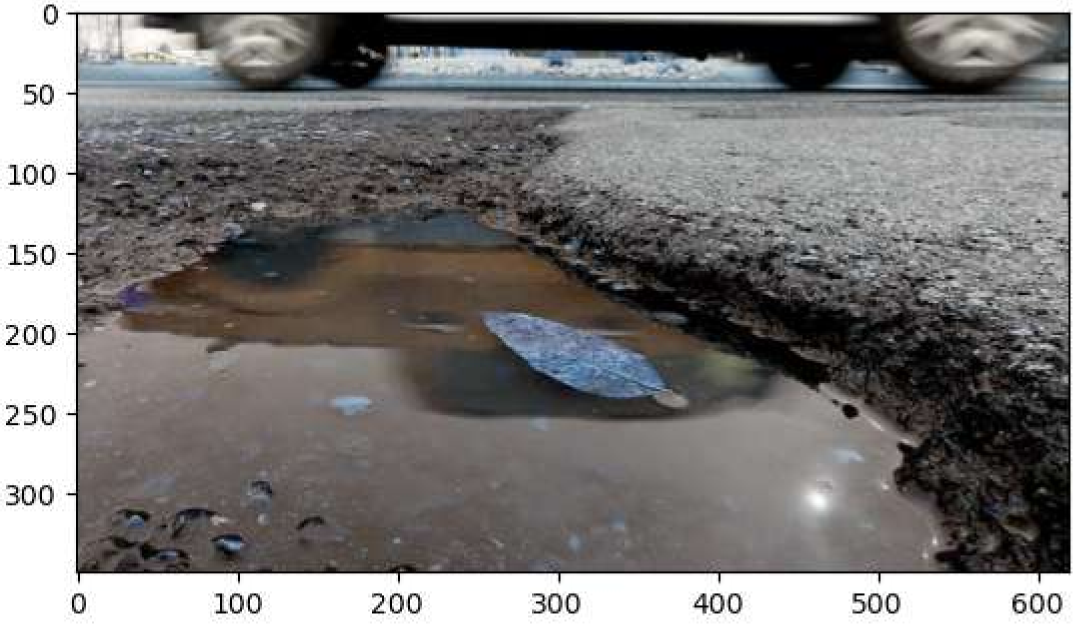
0
50
100
150
200
250
300
0
100
200
300
400
500
600

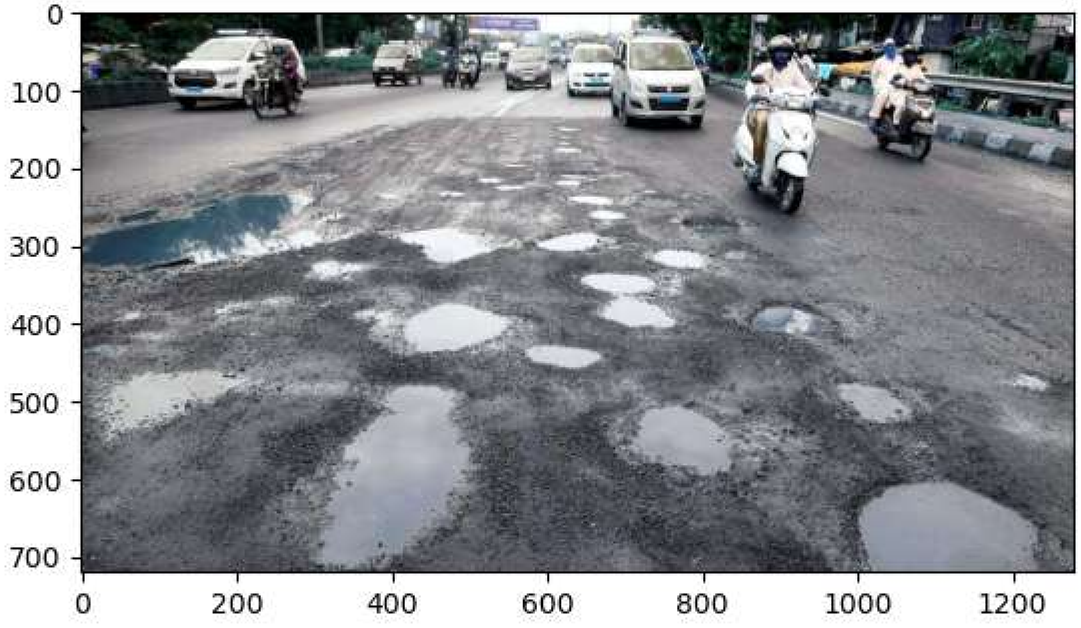
0
100
200
300
400
500
600
700
0
200
400
600
800
1000
1200

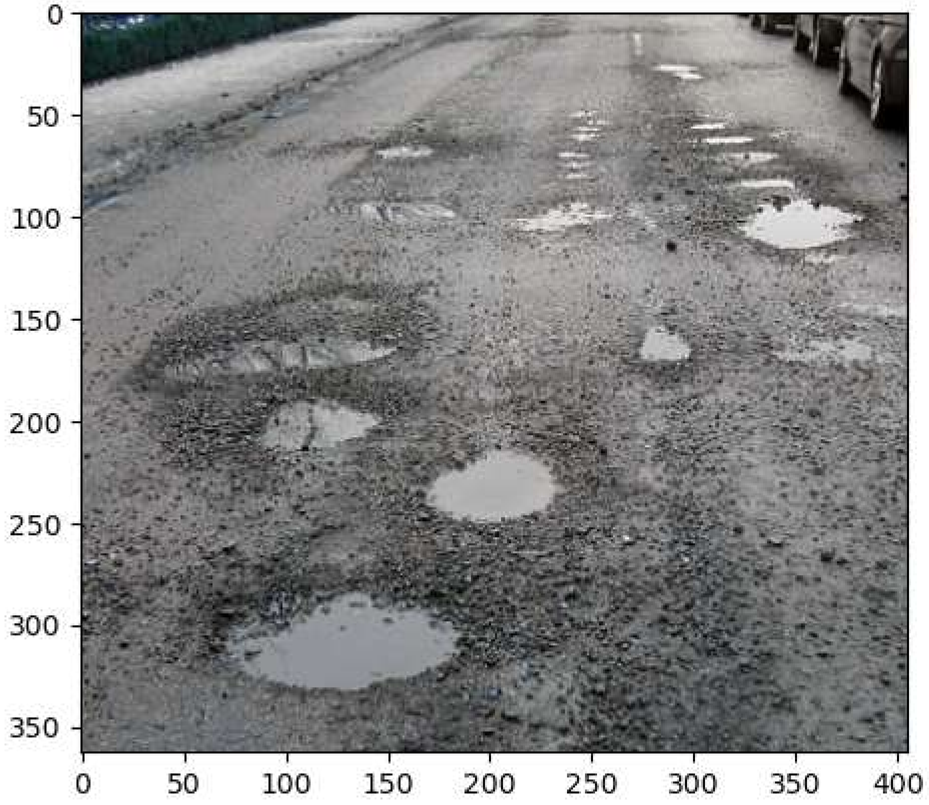
0
50
100
150
200
250
300
350
0
50
100
150
200
250
300
350
400

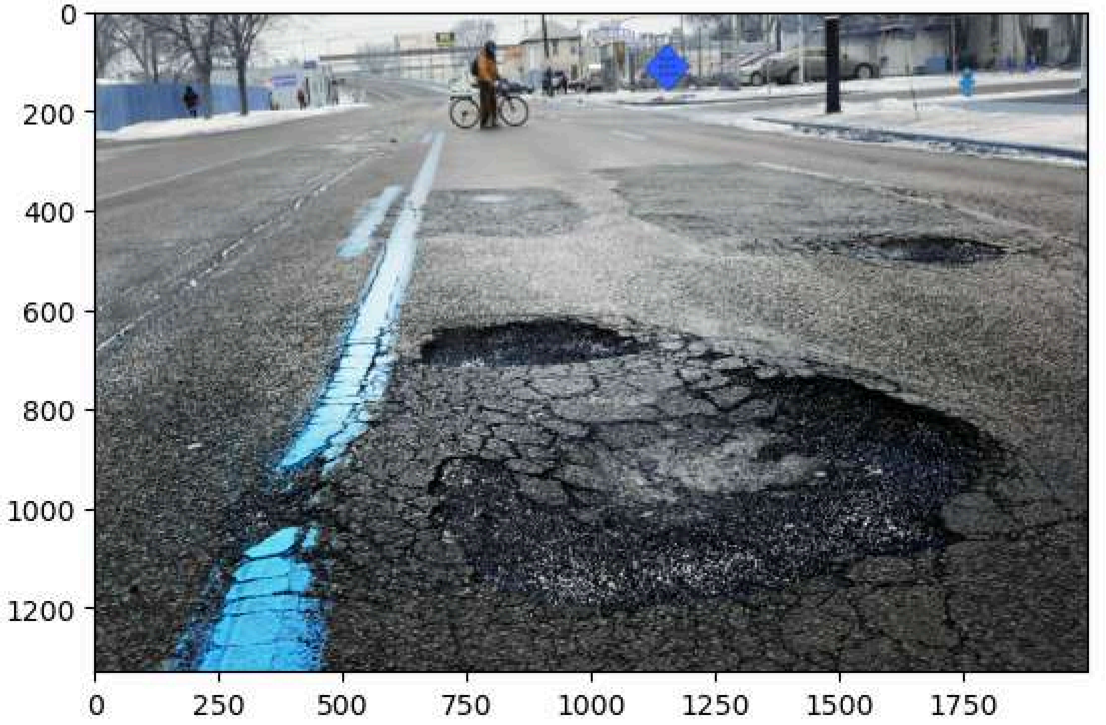
0
200
400
600
800
1000
1200
0 250 500 750 1000 1250 1500 1750

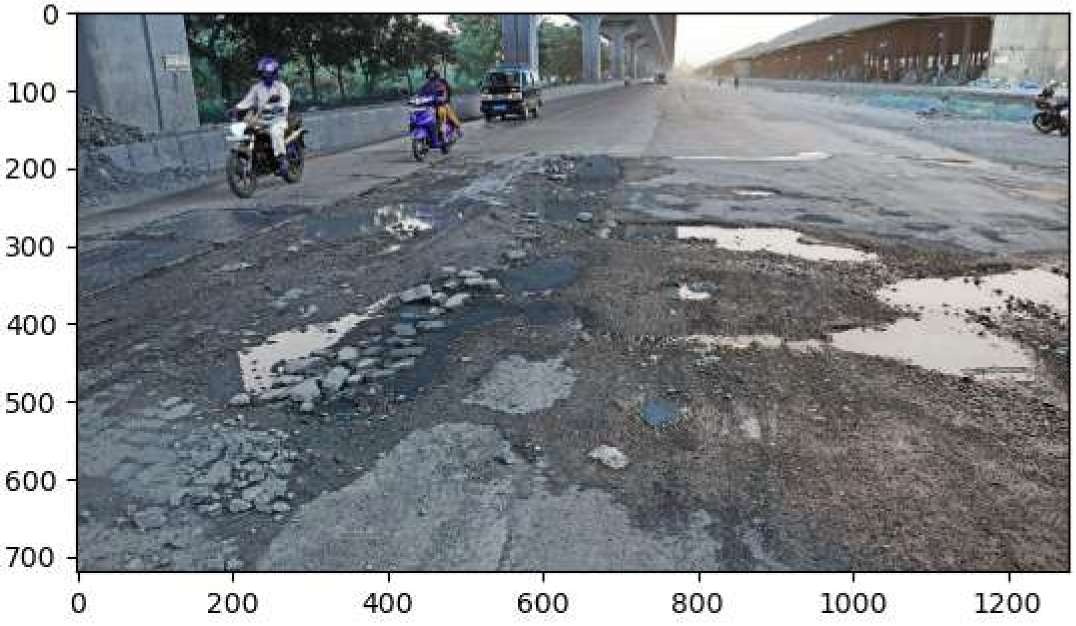
0
100
200
300
400
500
600
700
0 200 400 600 800 1000 1200

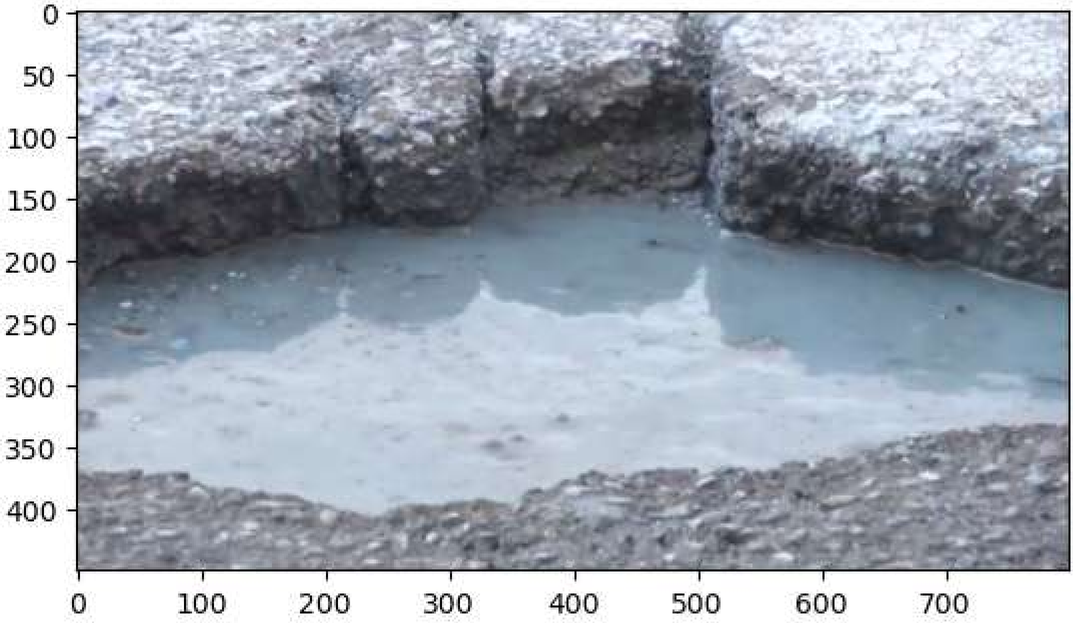
0
50
100
150
200
250
300
350
400
0 100 200 300 400 500 600 700

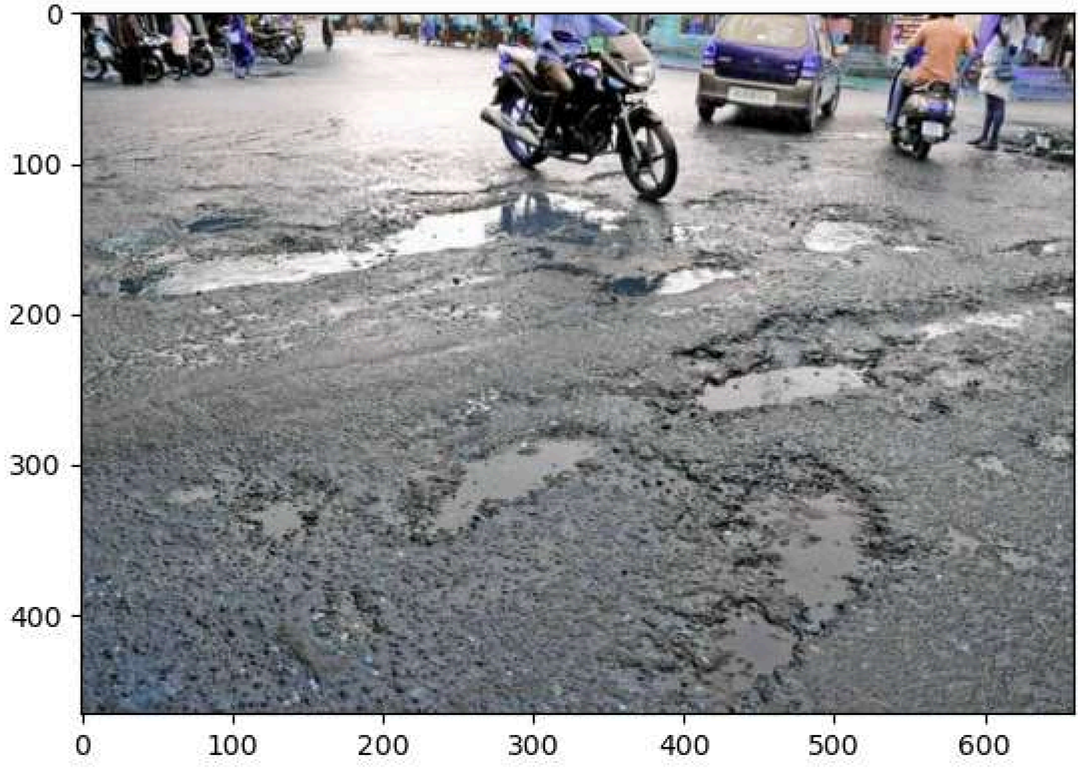
0
100
200
300
400
0 100 200 300 400 500 600

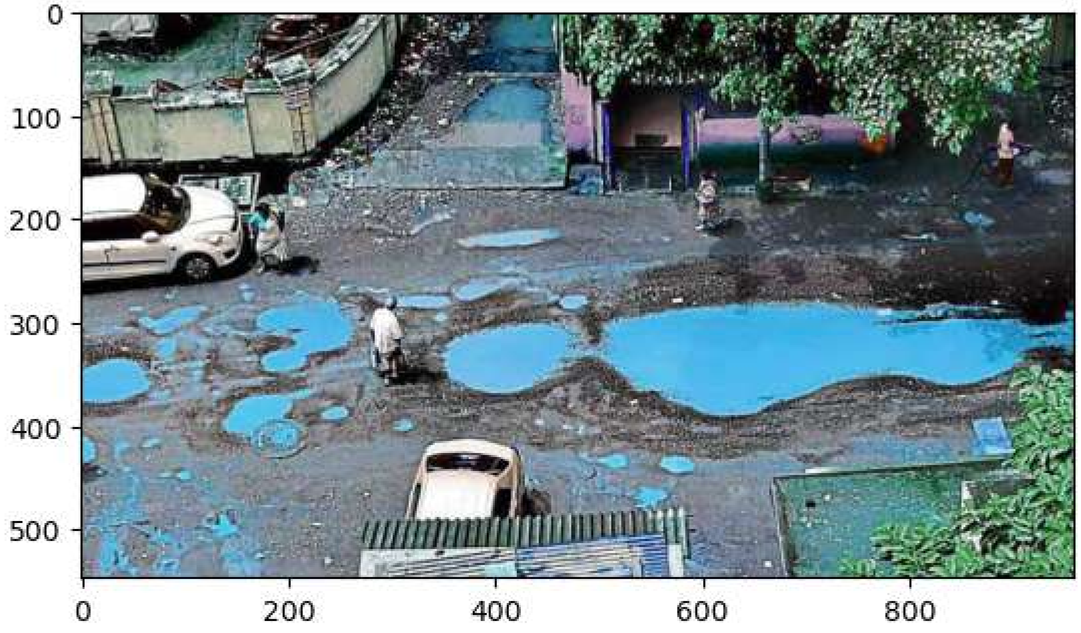
0
100
200
300
400
500
0 200 400 600 800

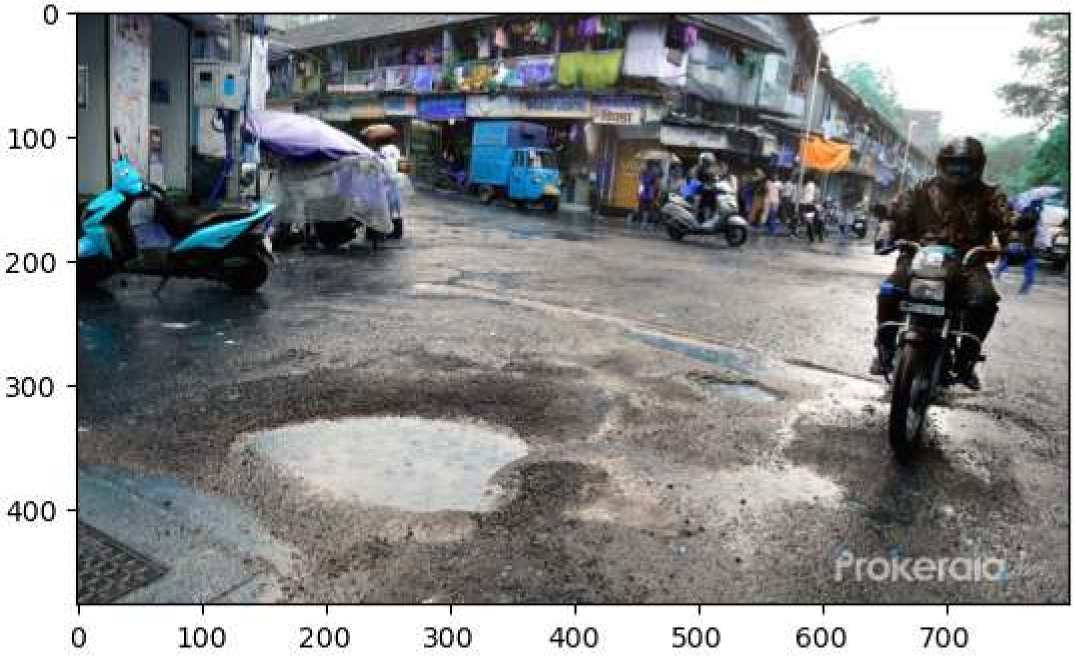
0
100
200
300
400
0
100
200
300
400
500
600
700
Prokerala

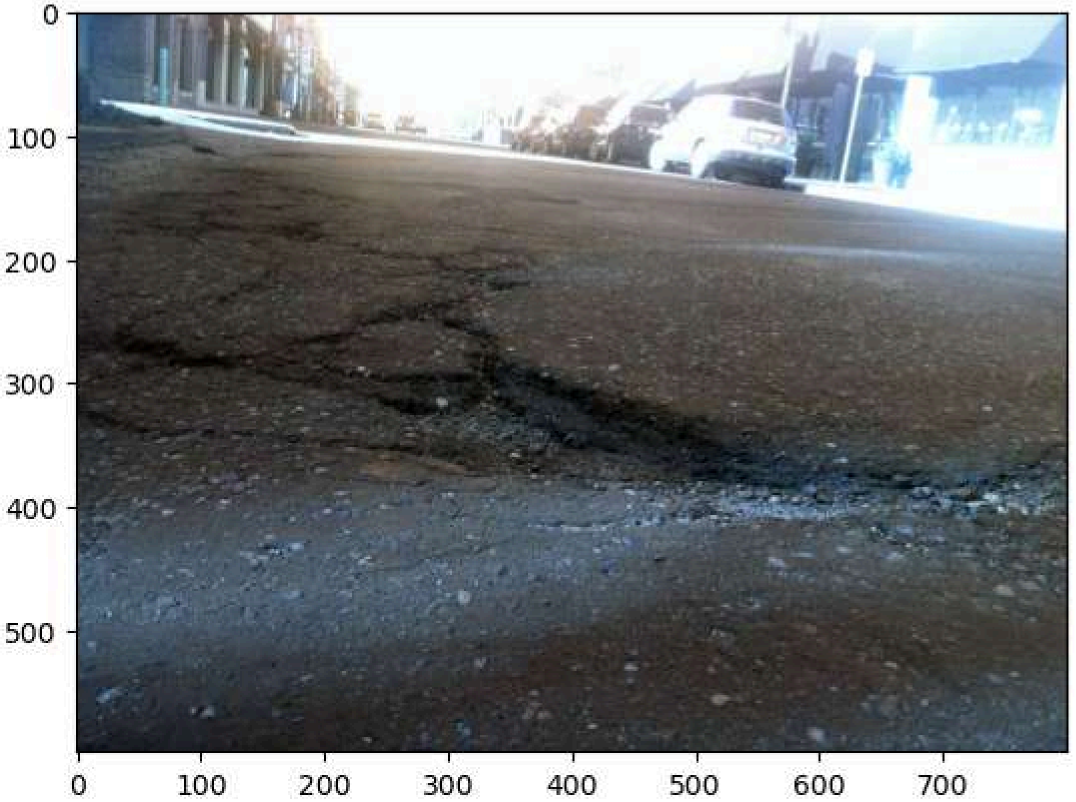
0
100
200
300
400
500
0
100
200
300
400
500
600
700

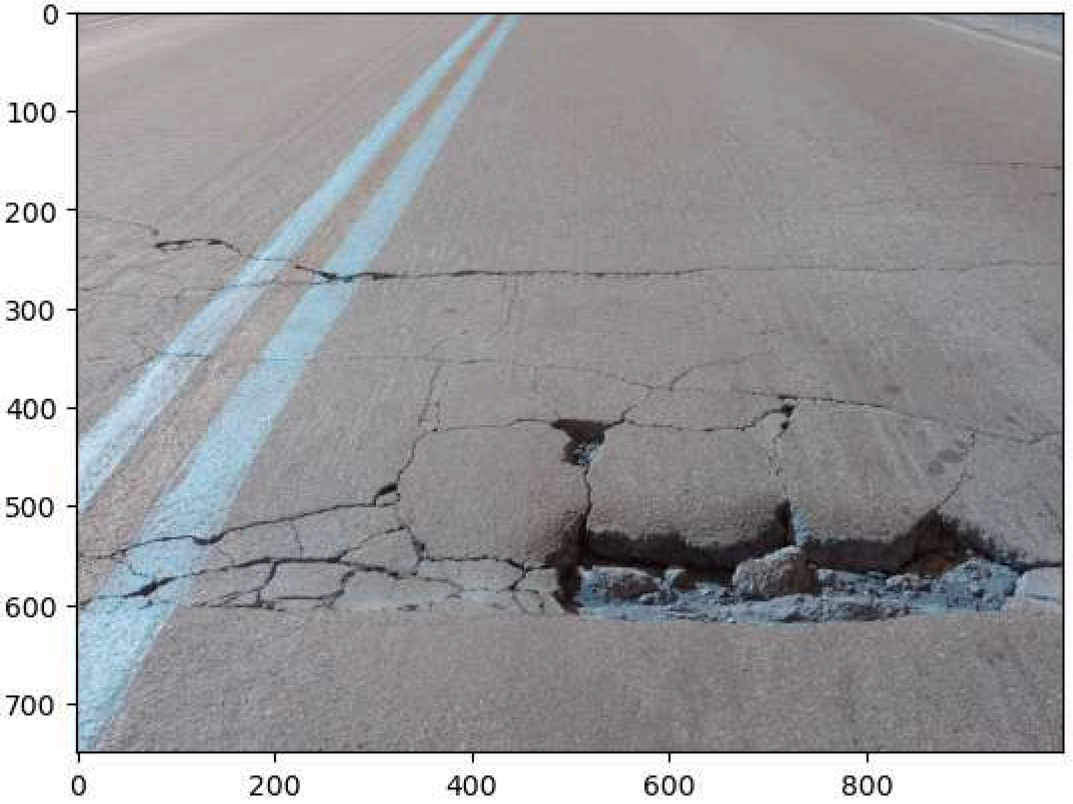
0
100
200
300
400
500
600
700
0
200
400
600
800

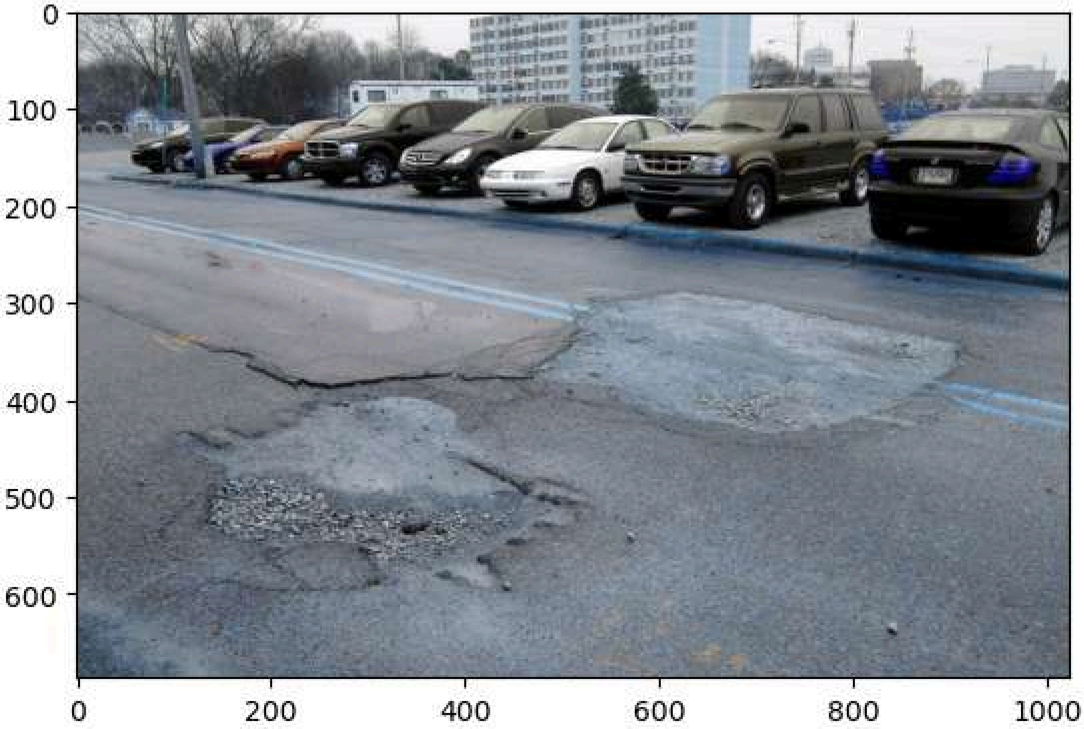
0
100
200
300
400
500
600
0
200
400
600
800
1000

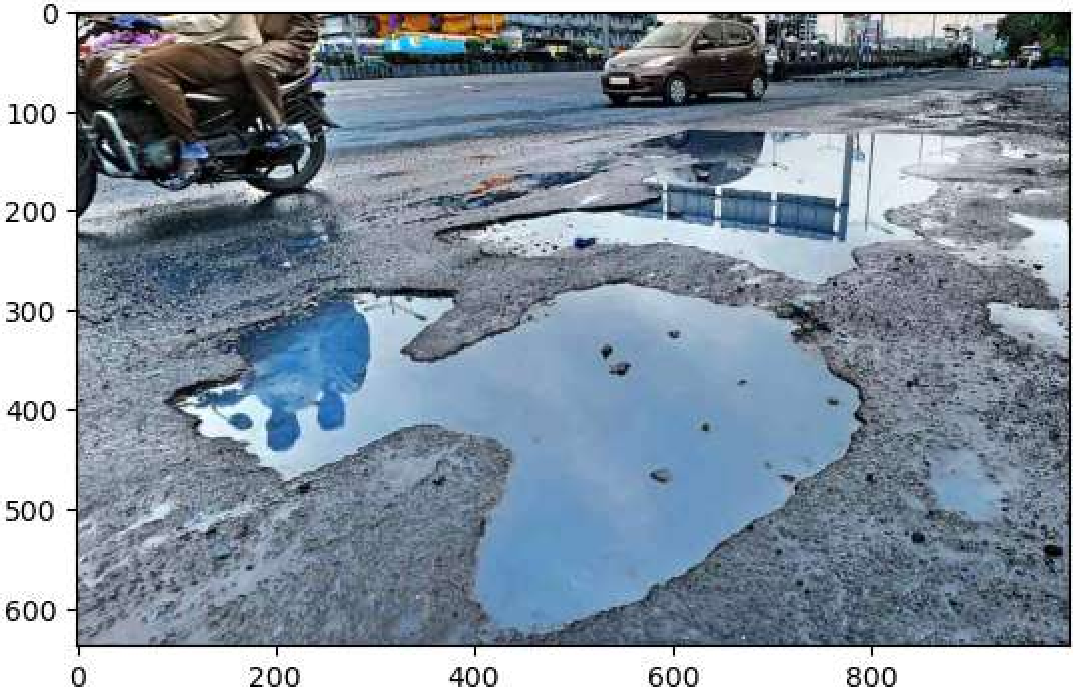
0
100
200
300
400
500
600
0
200
400
600
800

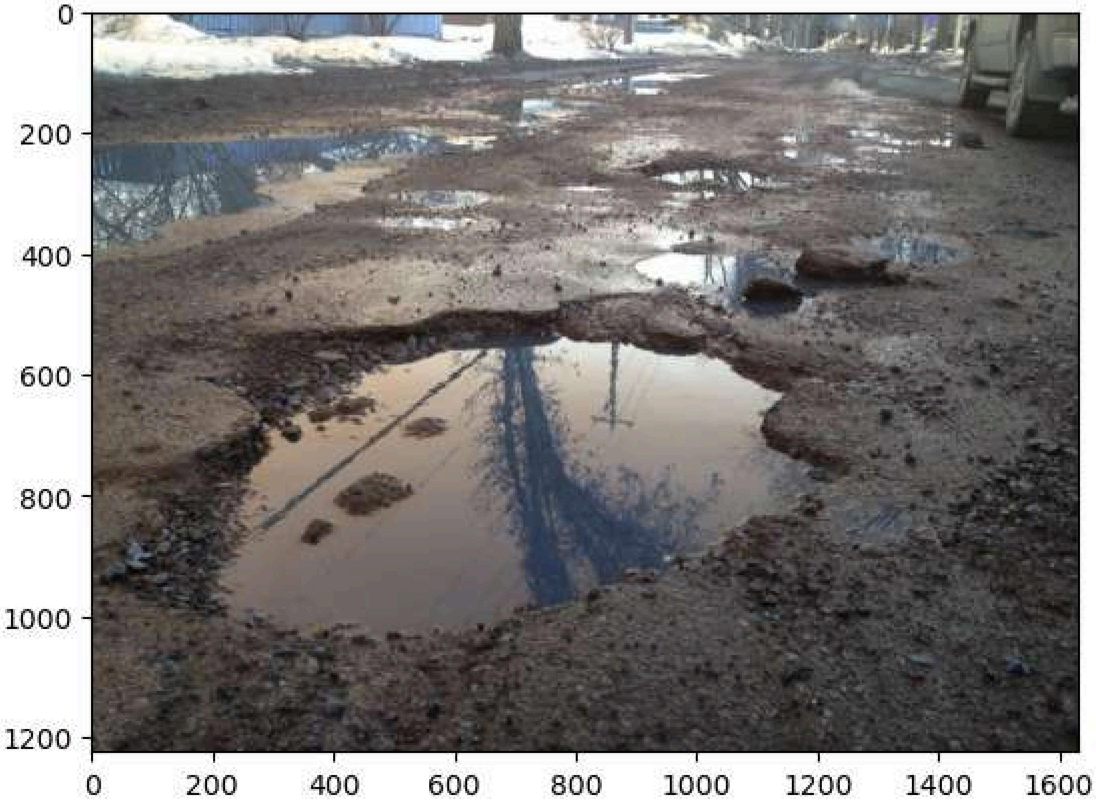
0
200
400
600
800
1000
1200
0
200
400
600
800
1000
1200
1400
1600

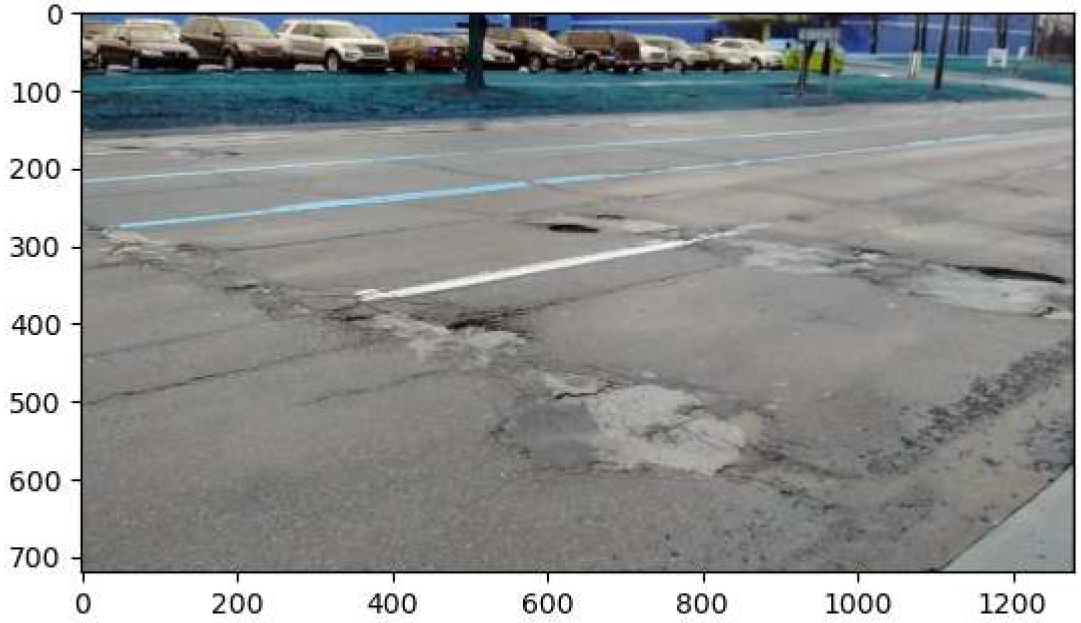
0
100
200
300
400
500
600
700
0
200
400
600
800
1000
1200

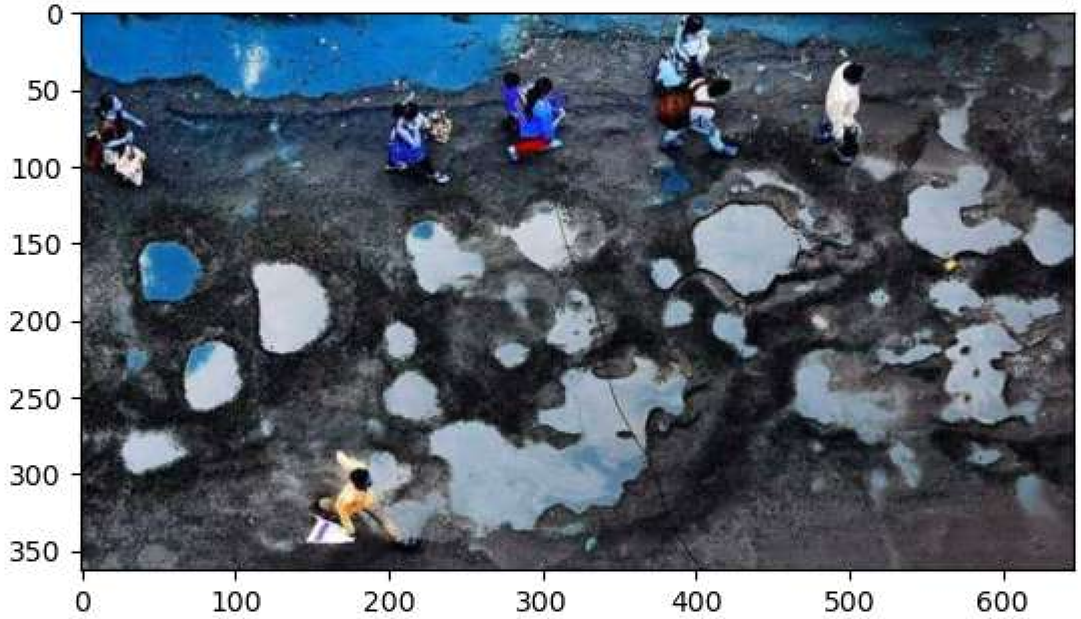
0
50
100
150
200
250
300
350
0
100
200
300
400
500
600

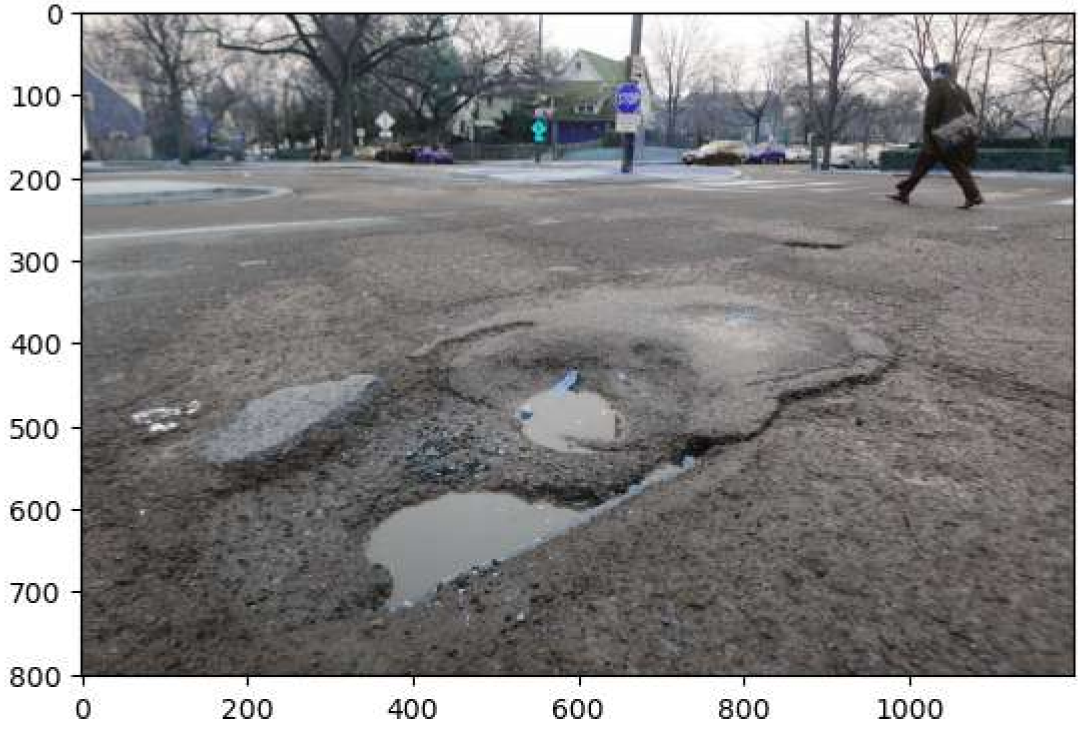
0
100
200
300
400
500
600
700
800
0
200
400
600
800
1000

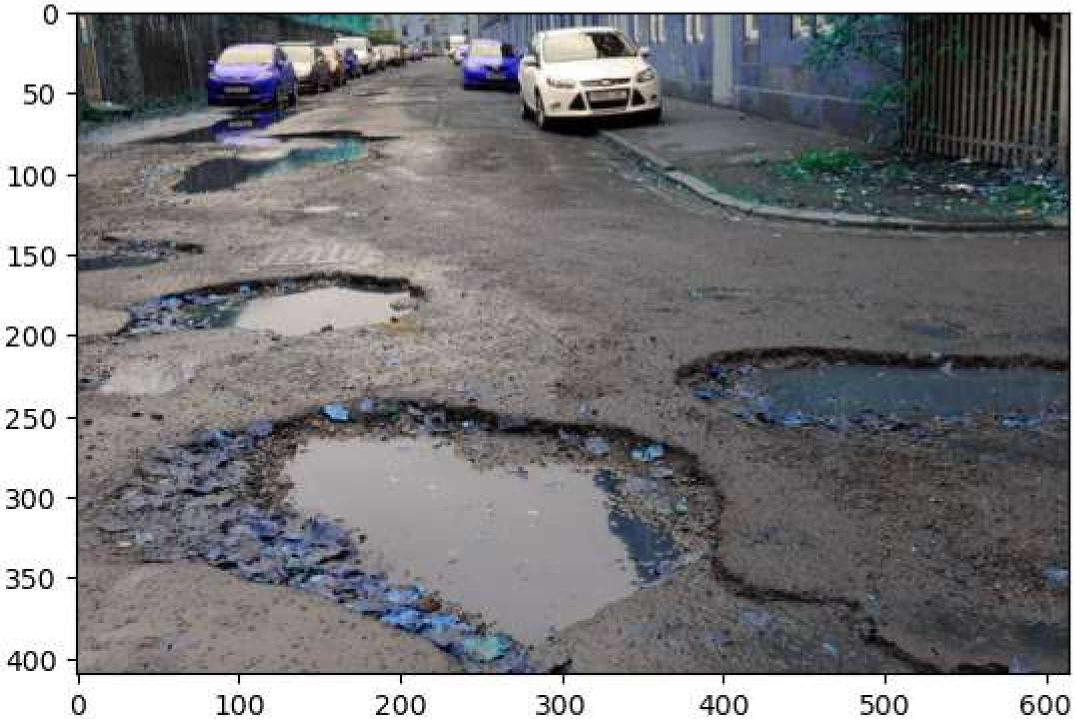
0
50
100
150
200
250
300
350
400
0
100
200
300
400
500
600

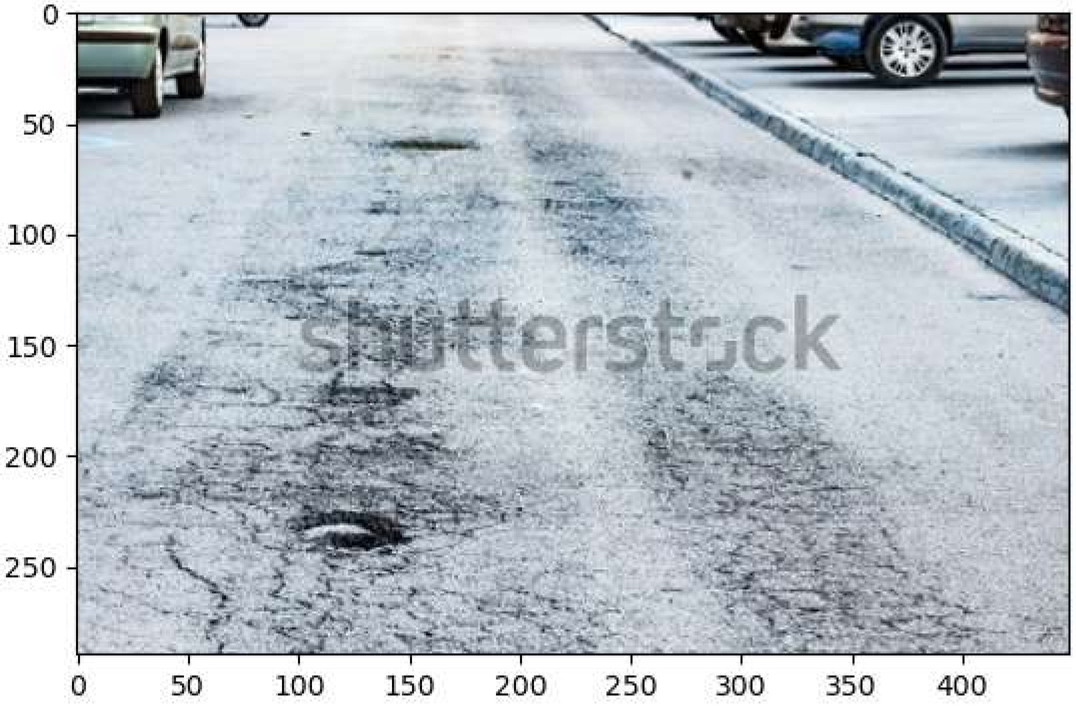
0
50
100
150
200
250
shutterstock
0
50
100
150
200
250
300
350
400

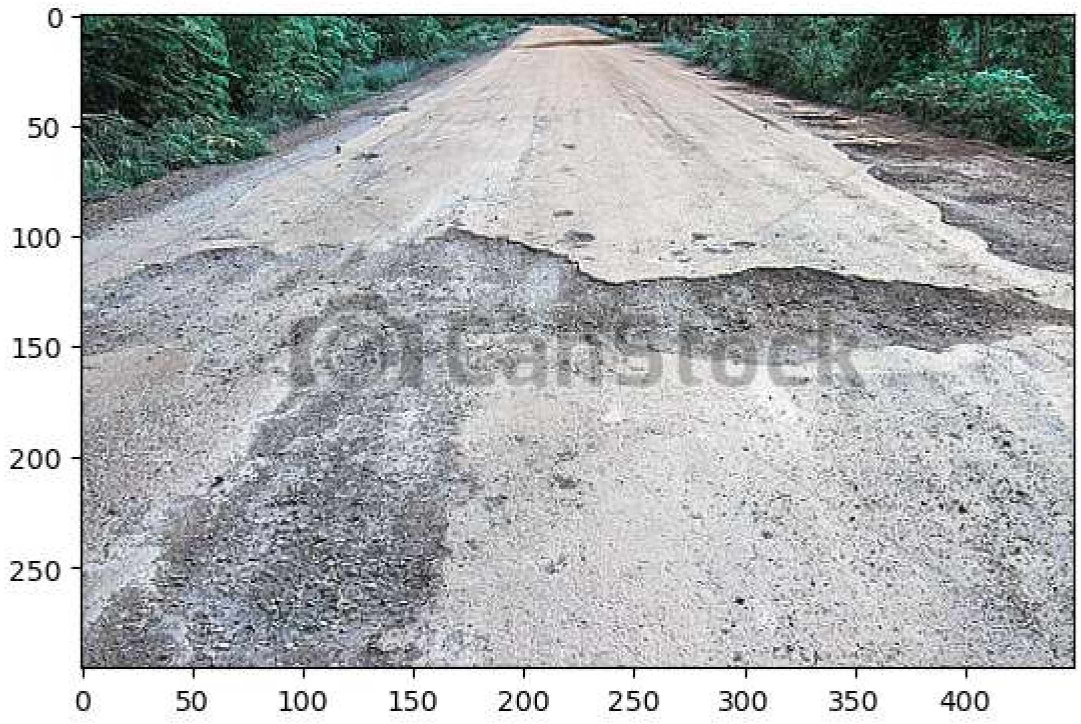
0
50
100
150
200
250
0
50
100
150
200
250
300
350
400

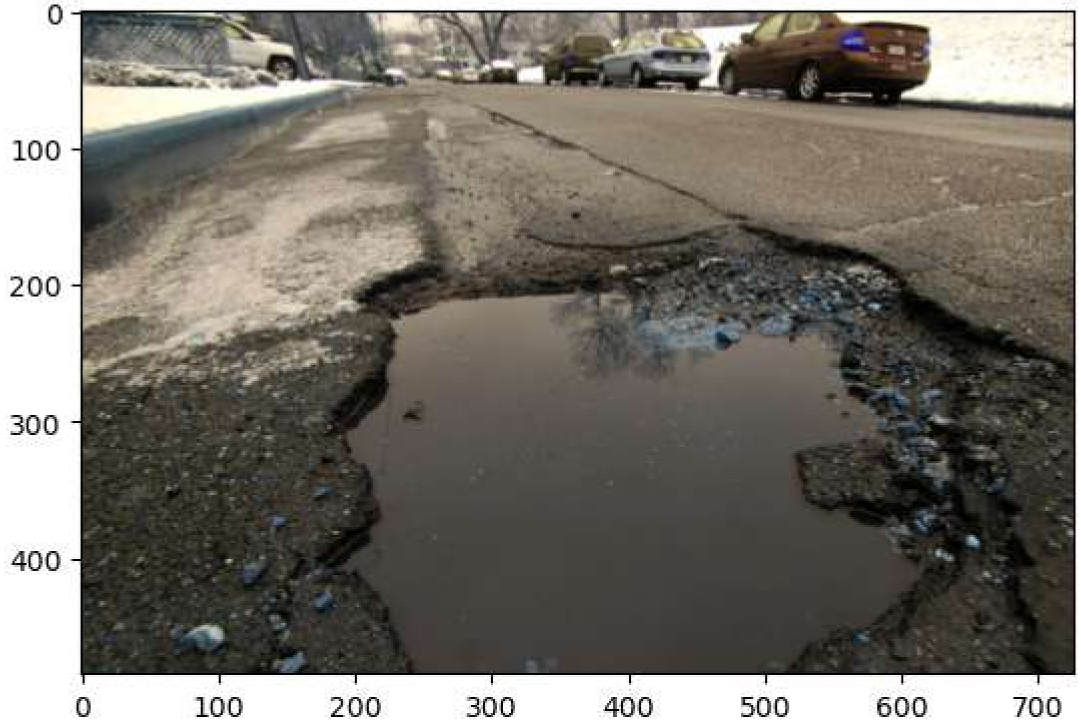
0
100
200
300
400
0
100
200
300
400
500
600
700

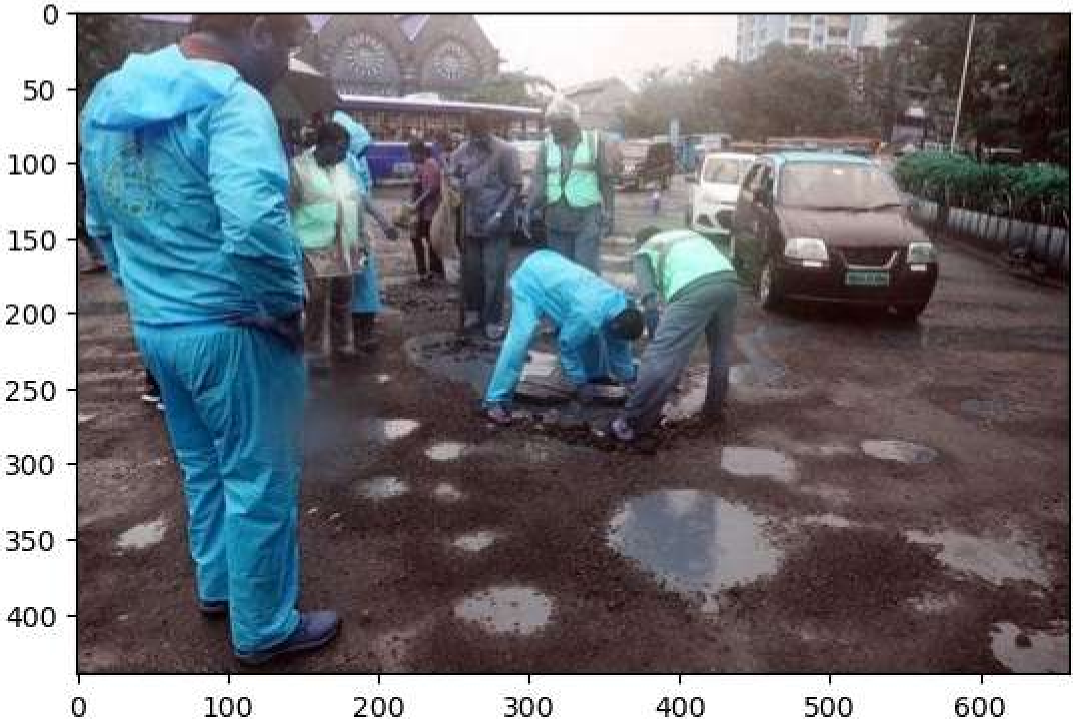
0
50
100
150
200
250
300
350
400
0
100
200
300
400
500
600

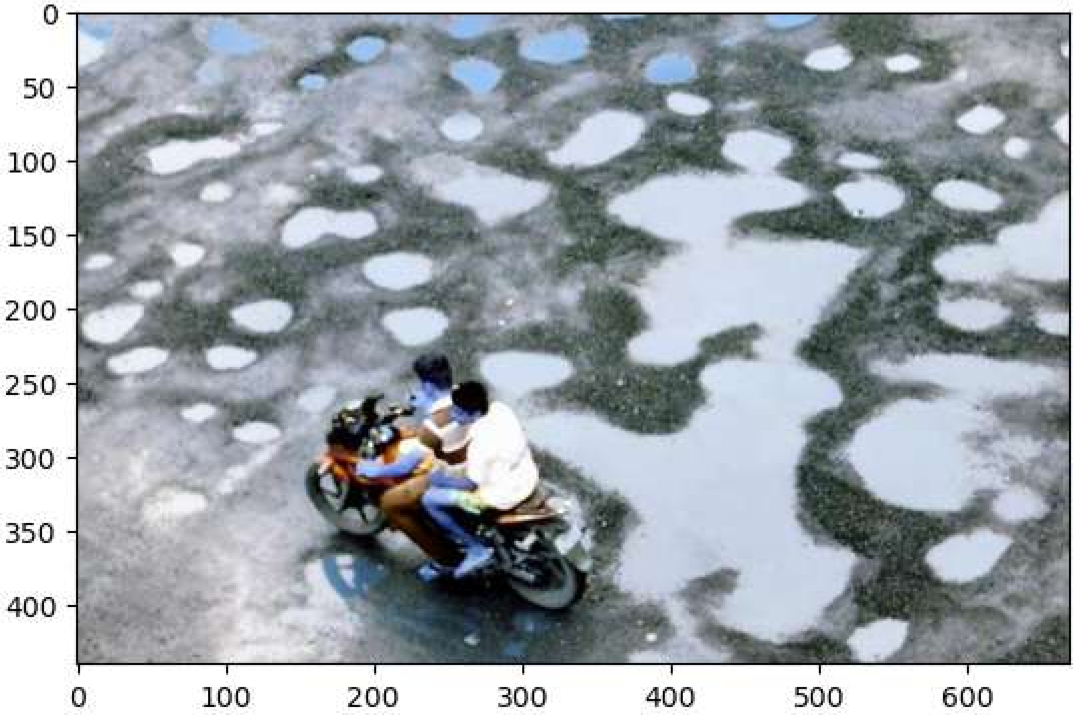
0
50
100
150
200
250
300
350
400
0
100
200
300
400
500
600

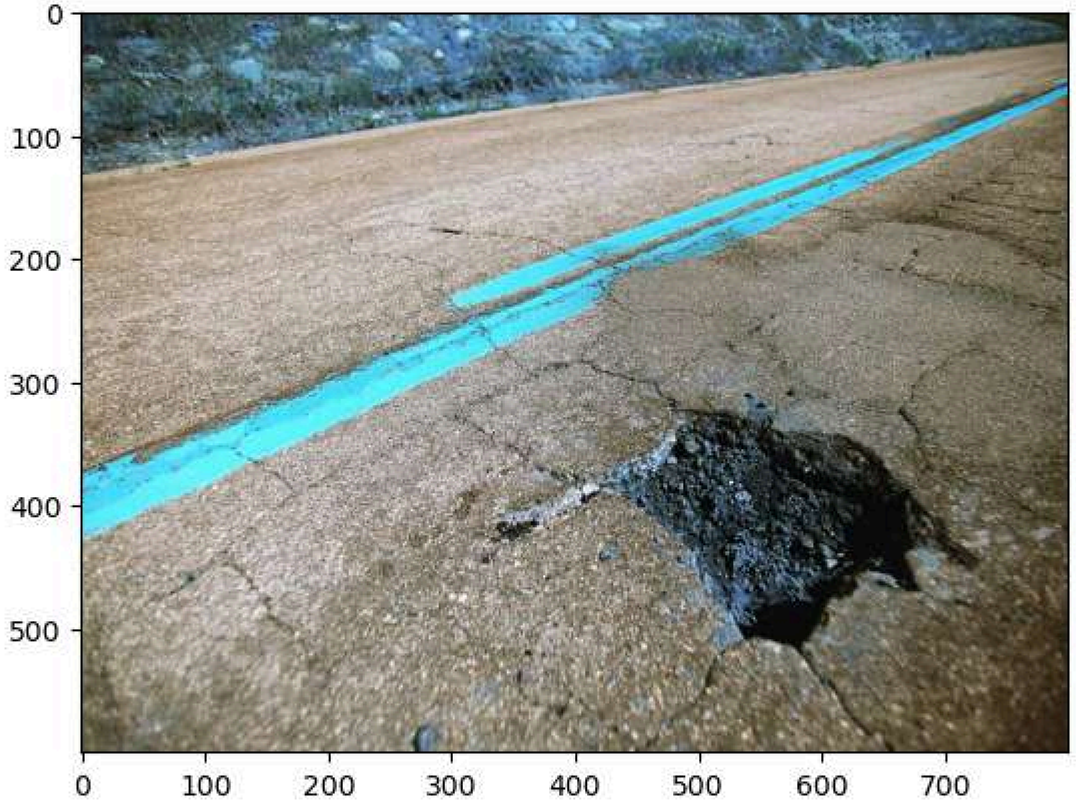
0
100
200
300
400
500
0
100
200
300
400
500
600
700

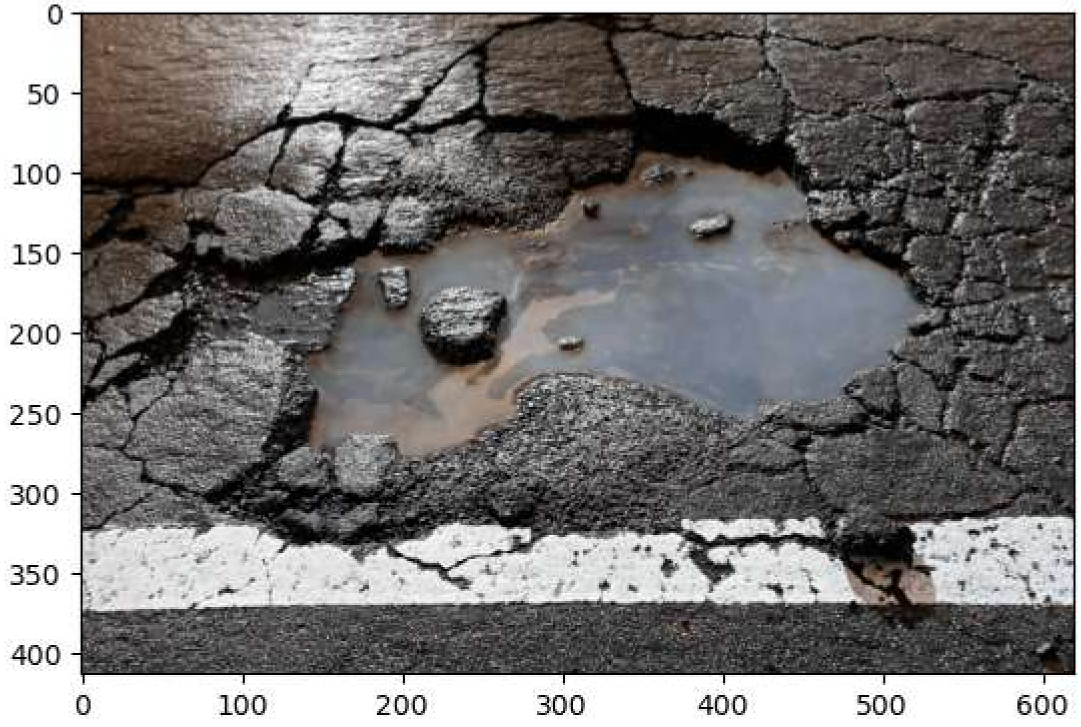
0
50
100
150
200
250
300
350
400
0
100
200
300
400
500
600

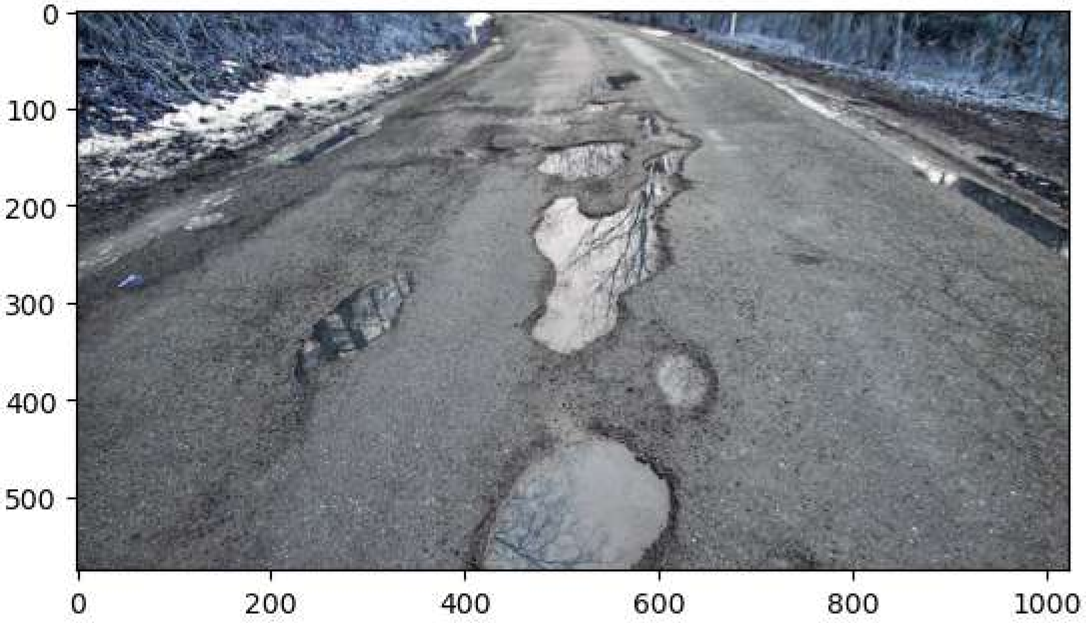
0
100
200
300
400
500
0
200
400
600
800
1000

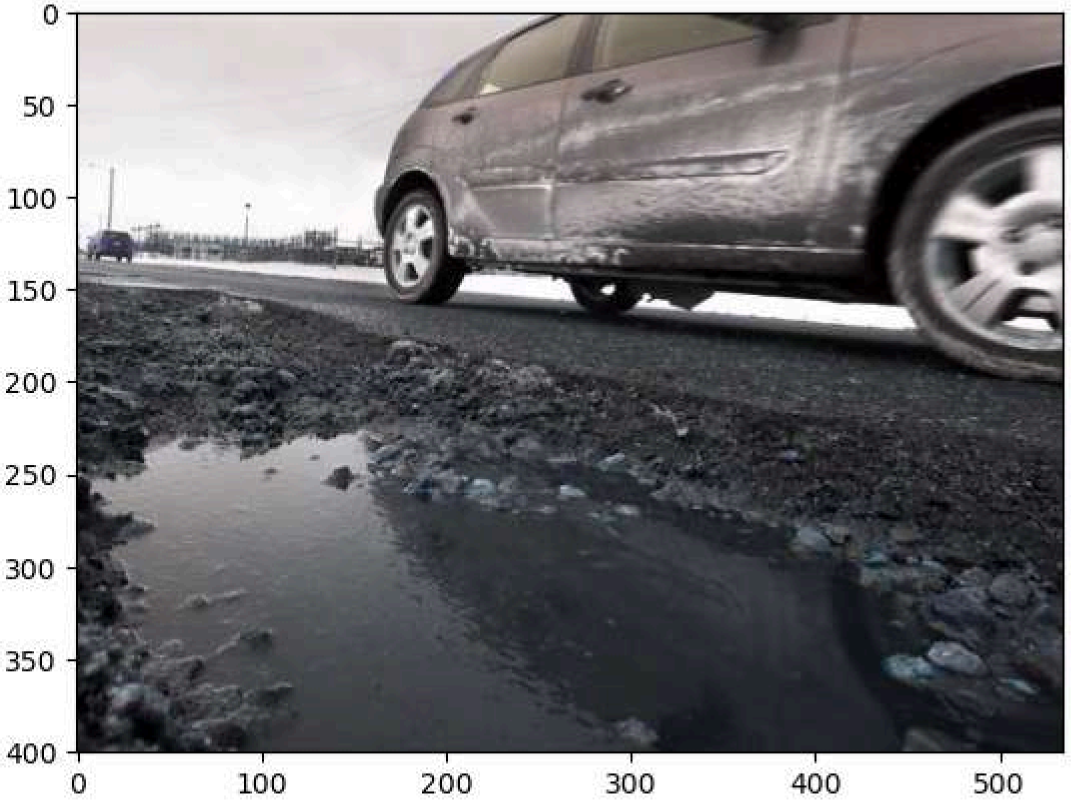
0
50
100
150
200
250
300
350
400
0
100
200
300
400
500

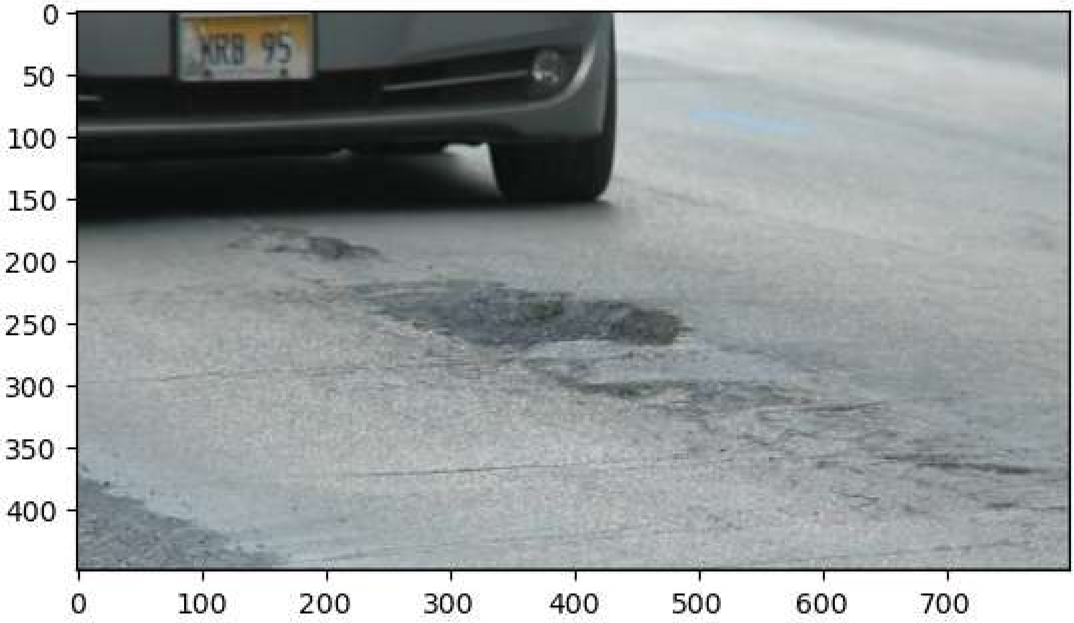
0
50
100
150
200
250
300
350
400
0 100 200 300 400 500 600 700

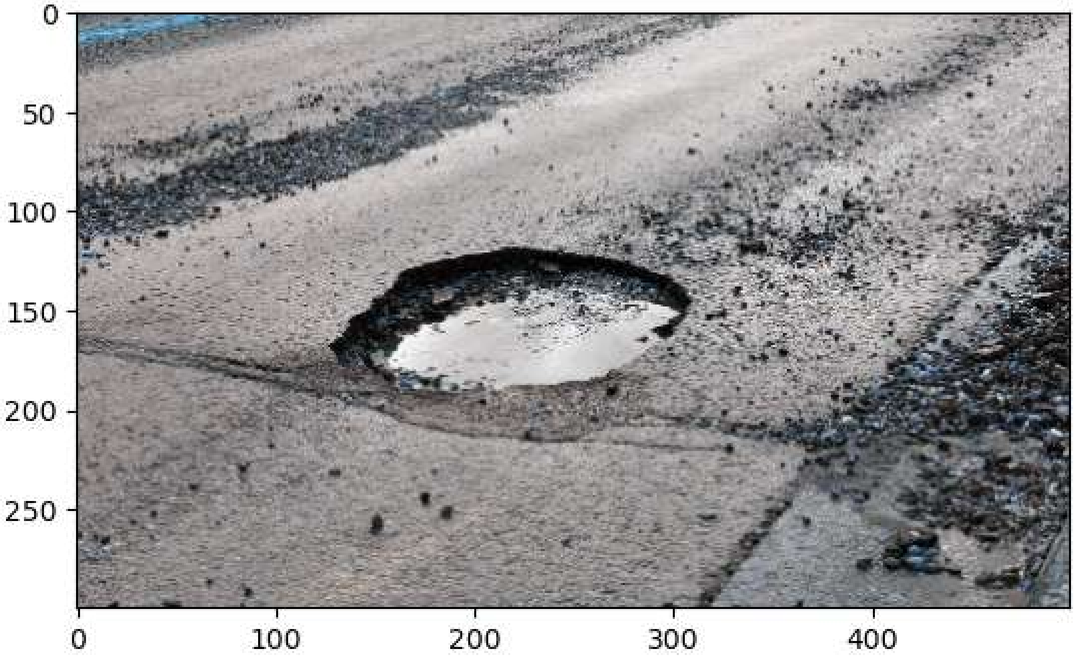
0
50
100
150
200
250
0 100 200 300 400

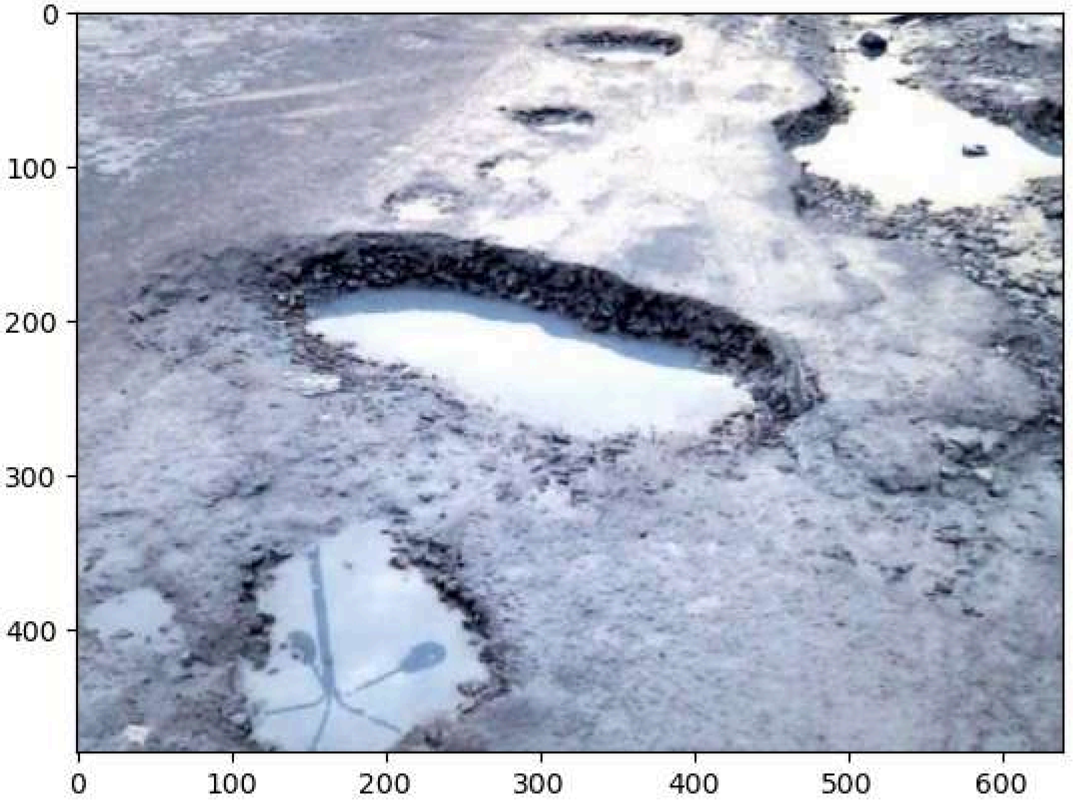
0
100
200
300
400
0 100 200 300 400 500 600

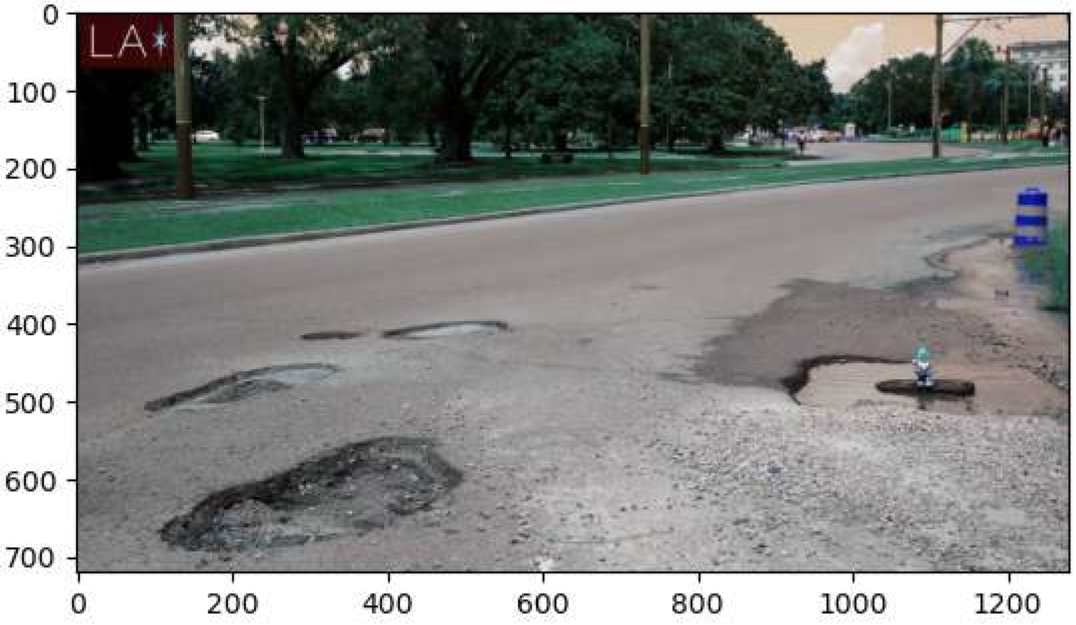
LA
0
100
200
300
400
500
600
700
0 200 400 600 800 1000 1200

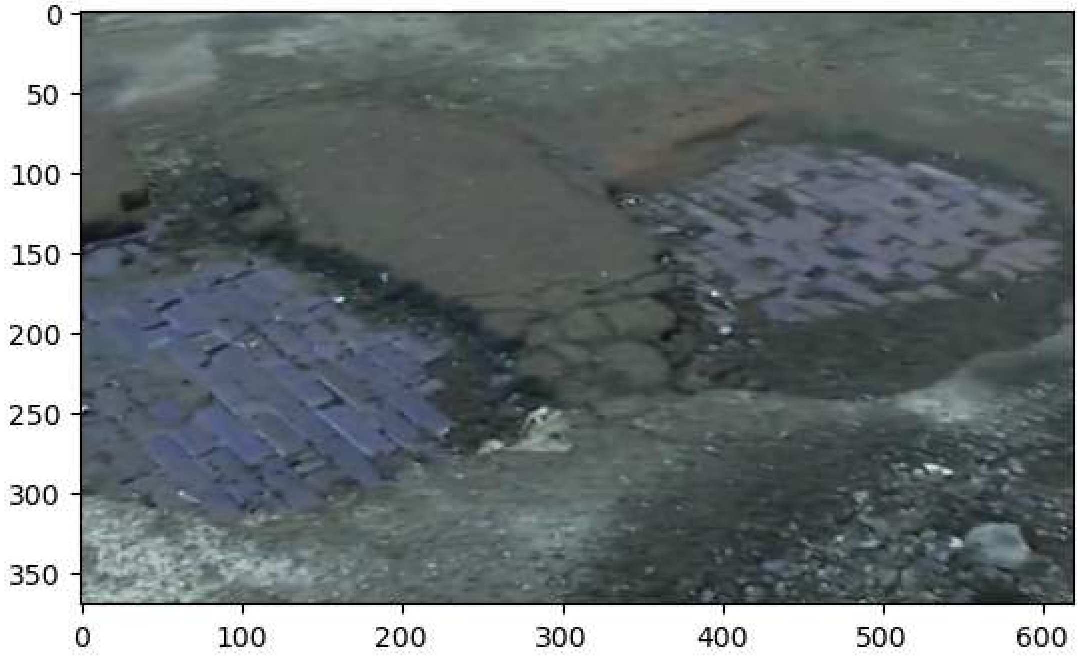
0
50
100
150
200
250
300
350
0 100 200 300 400 500 600

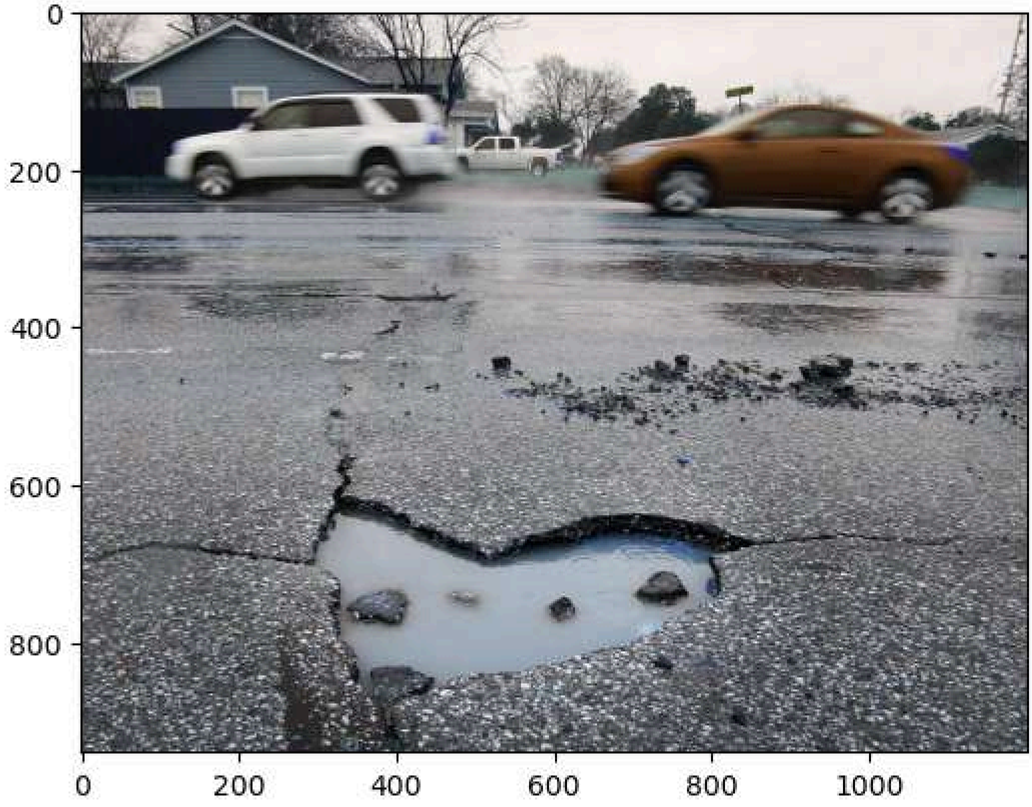
0
200
400
600
800
0 200 400 600 800 1000

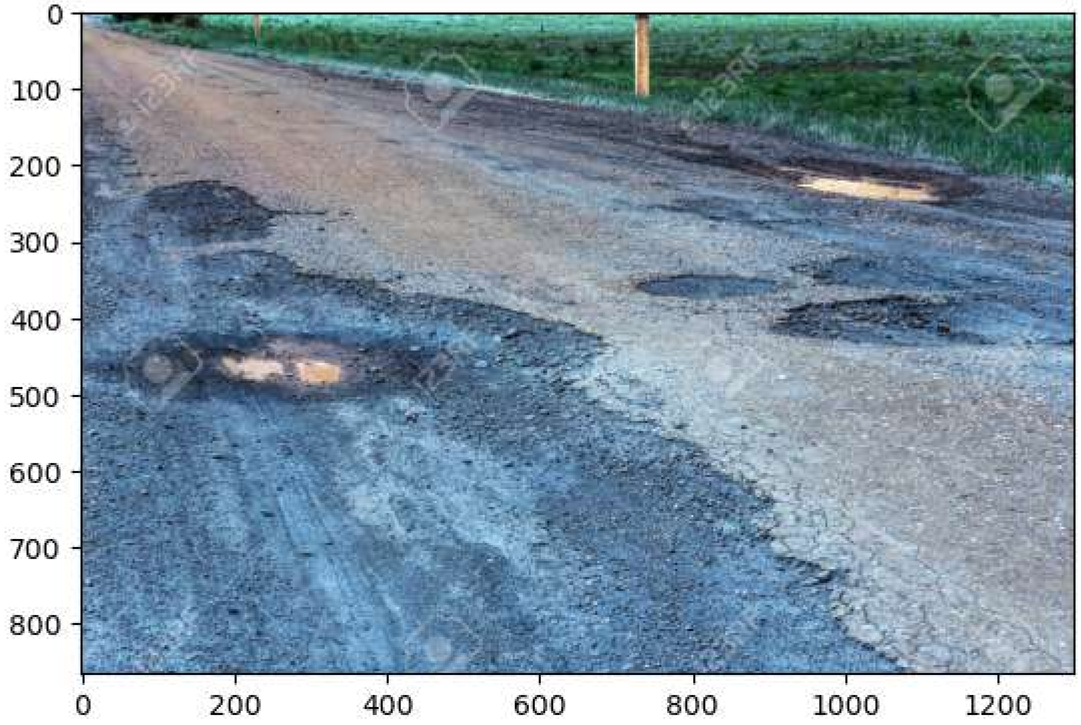
0
100
200
300
400
500
600
700
800
0
200
400
600
800
1000
1200

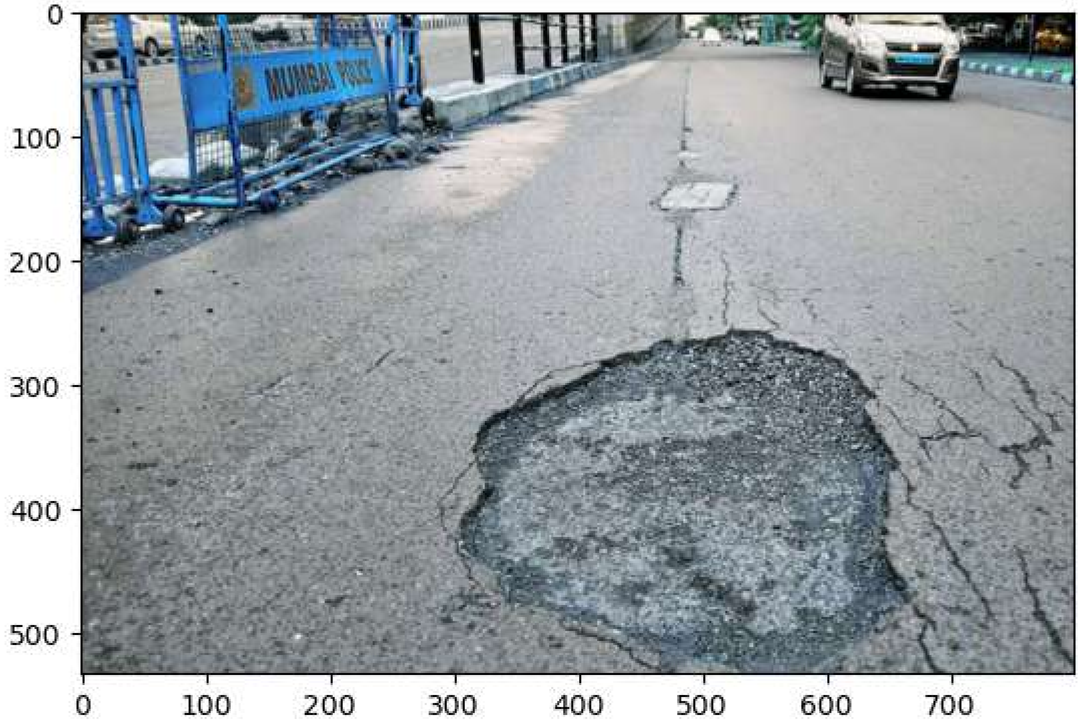
MUMBAI POLICE
0
100
200
300
400
500
0
100
200
300
400
500
600
700

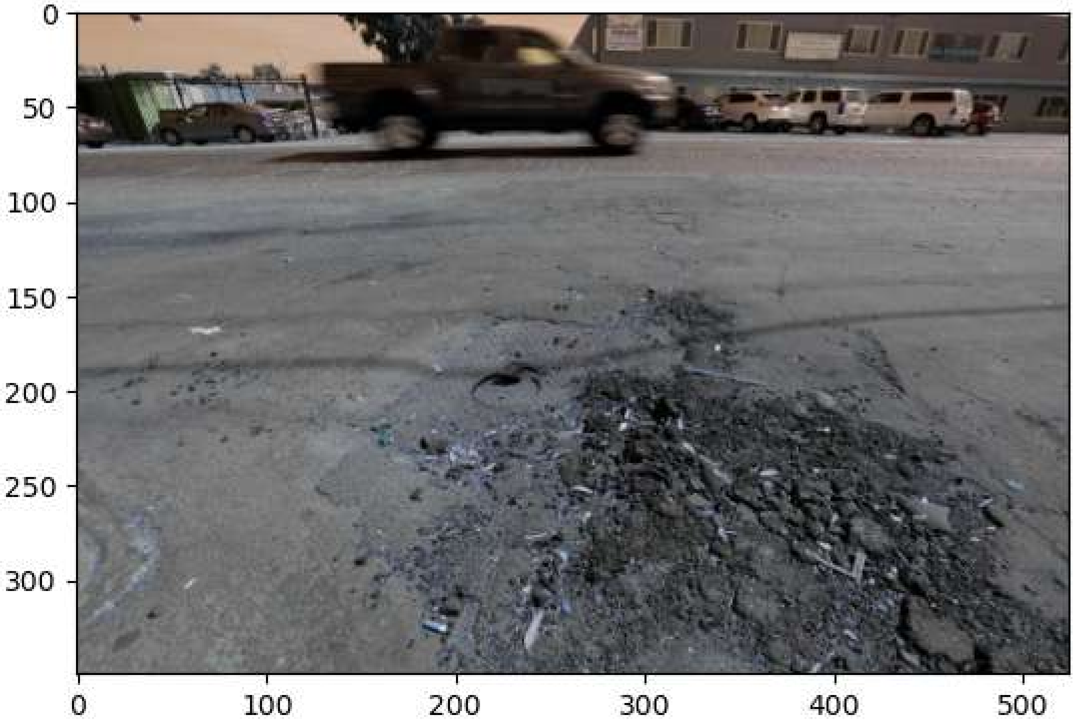
0
50
100
150
200
250
300
0
100
200
300
400
500

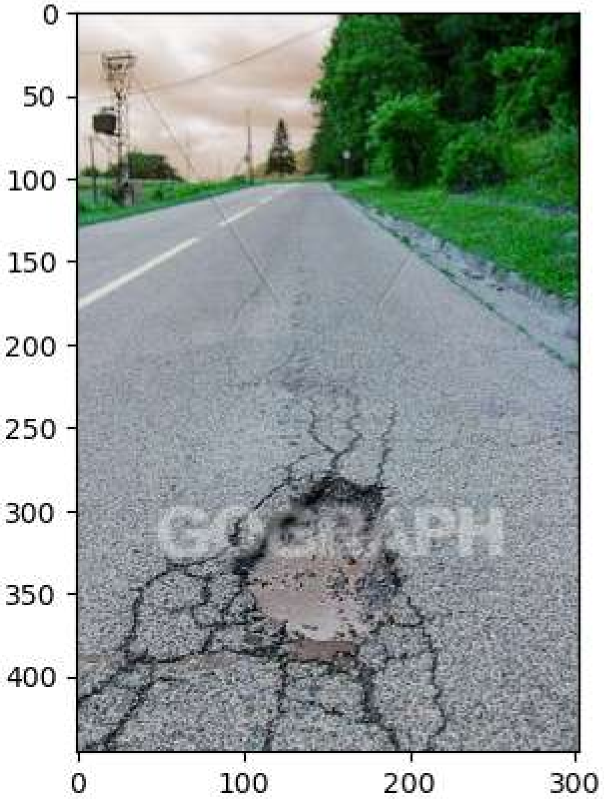
0
50
100
150
200
250
300
350
400
0
100
200
300

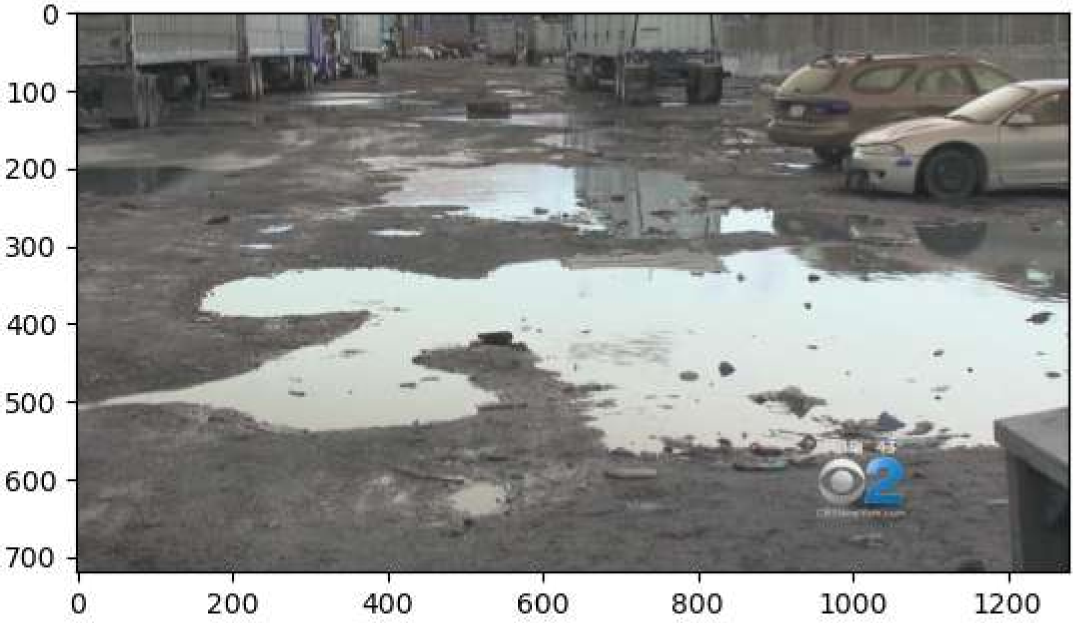
0
100
200
300
400
500
600
700
0
200
400
600
800
1000
1200

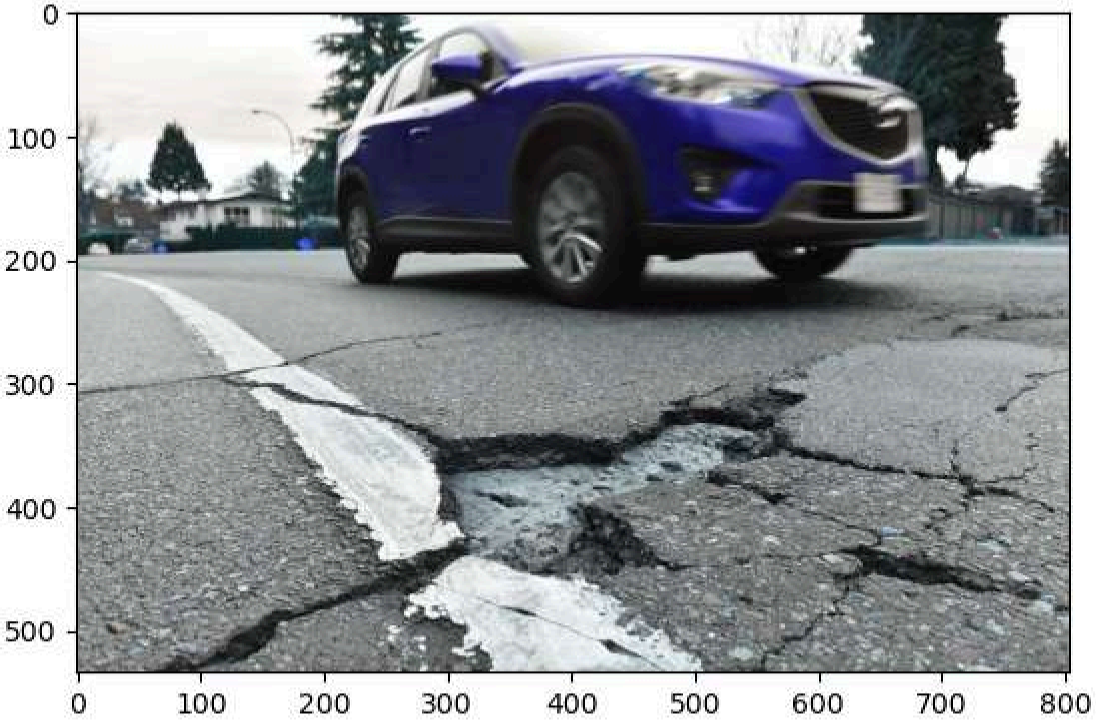
0
100
200
300
400
500
0
100
200
300
400
500
600
700
800

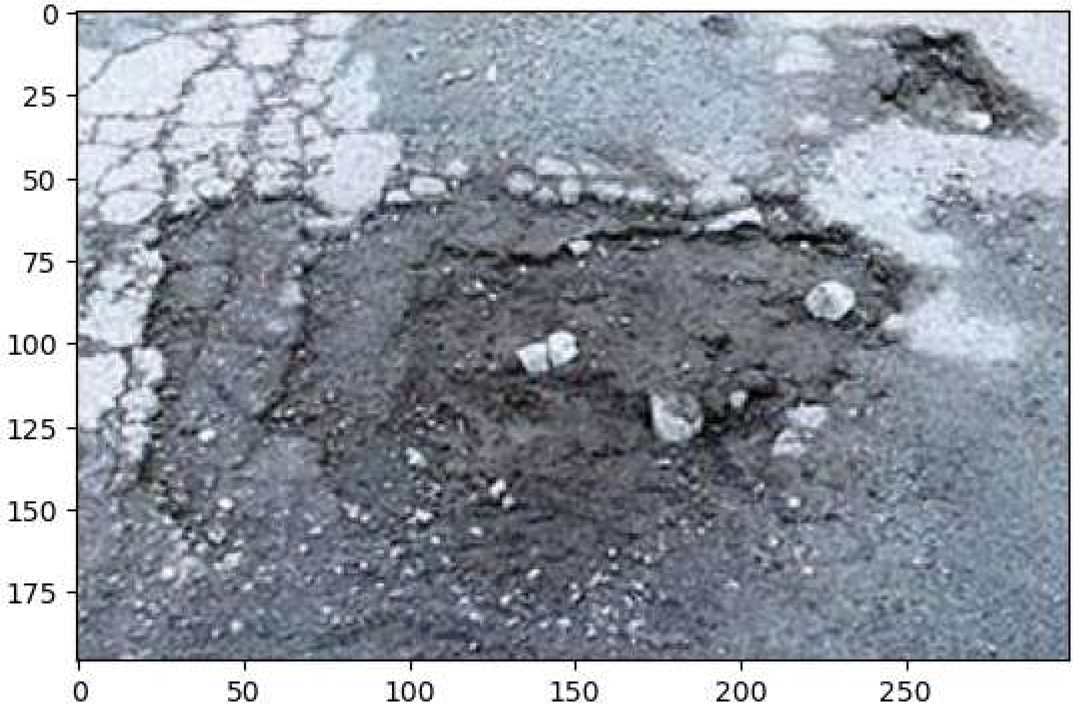
0
25
50
75
100
125
150
175
0
50
100
150
200
250

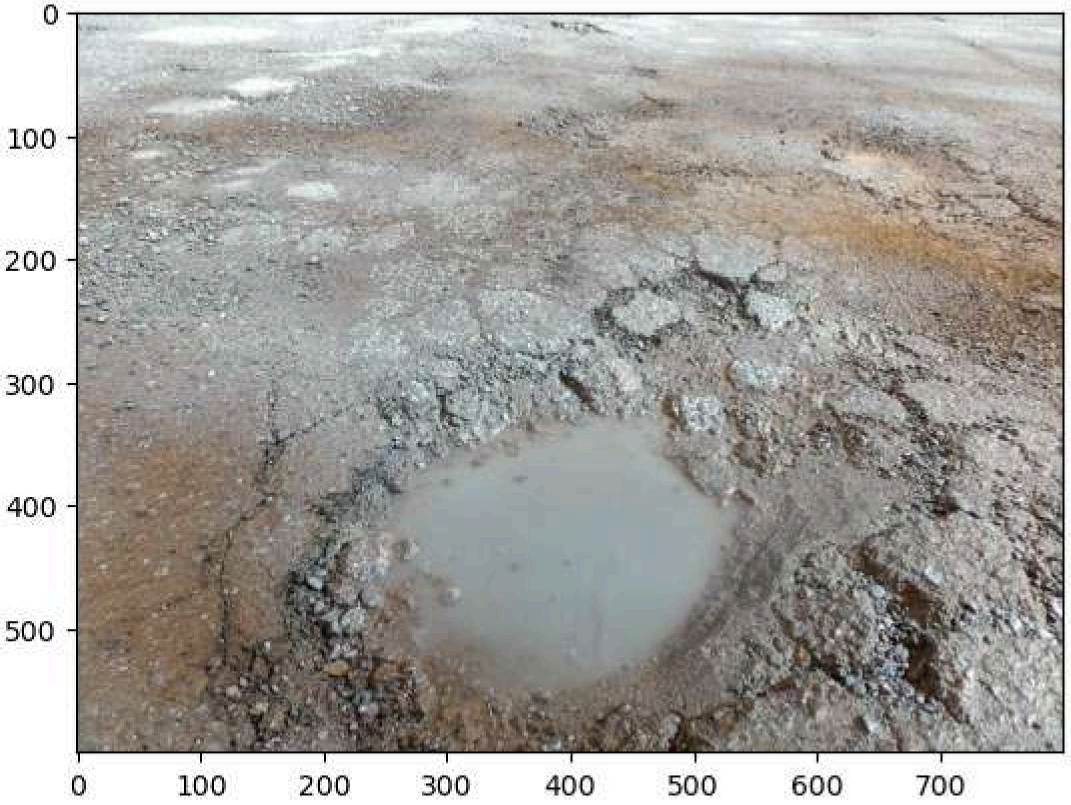
0
100
200
300
400
500
0
100
200
300
400
500
600
700

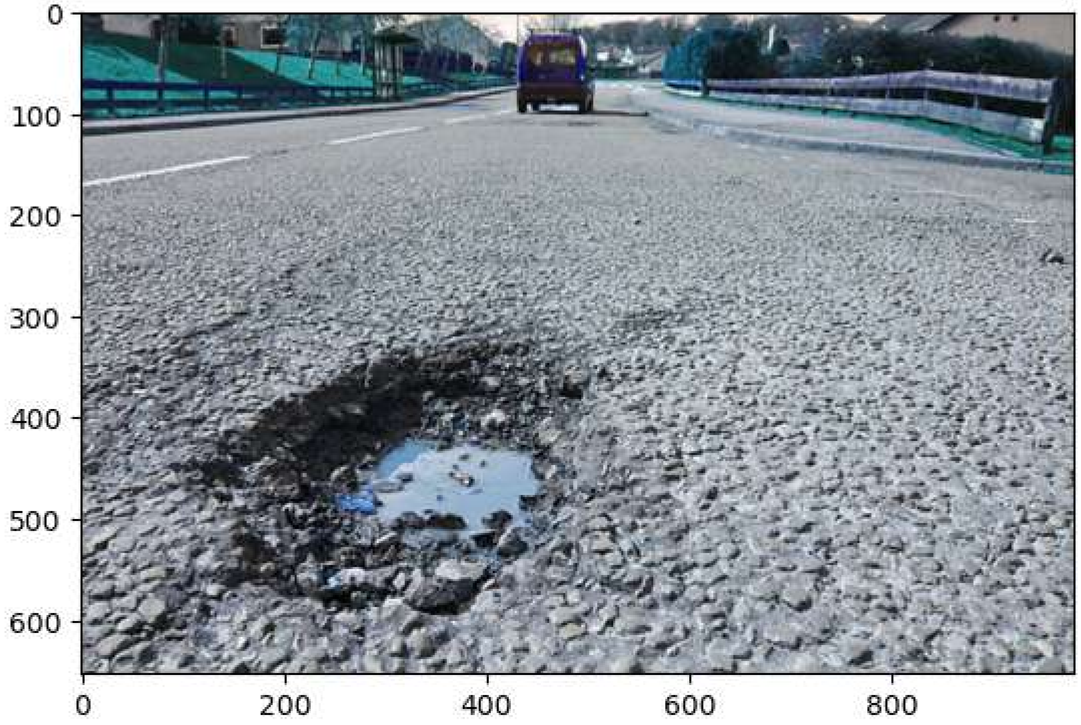
0
100
200
300
400
500
600
0
200
400
600
800

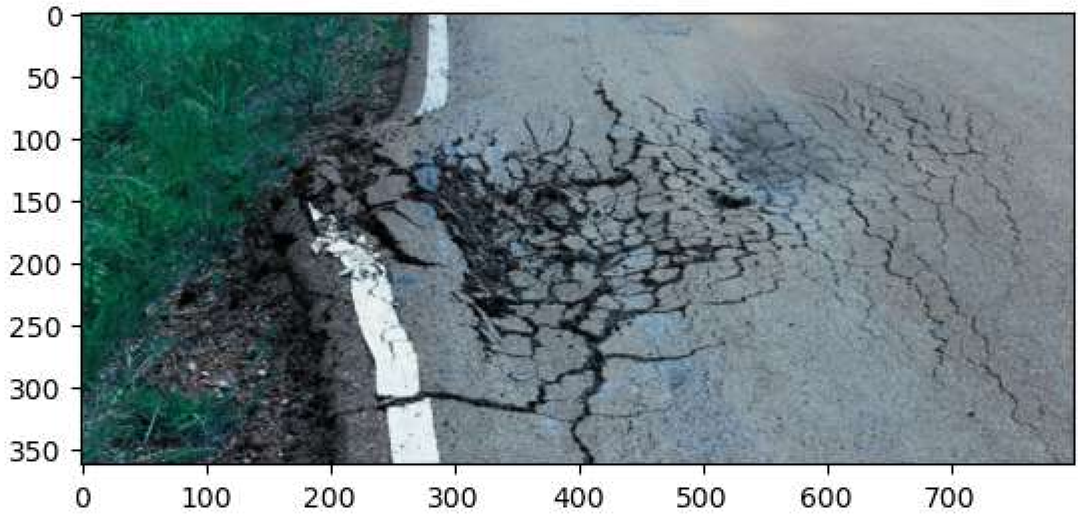
0
50
100
150
200
250
300
350
0
100
200
300
400
500
600
700

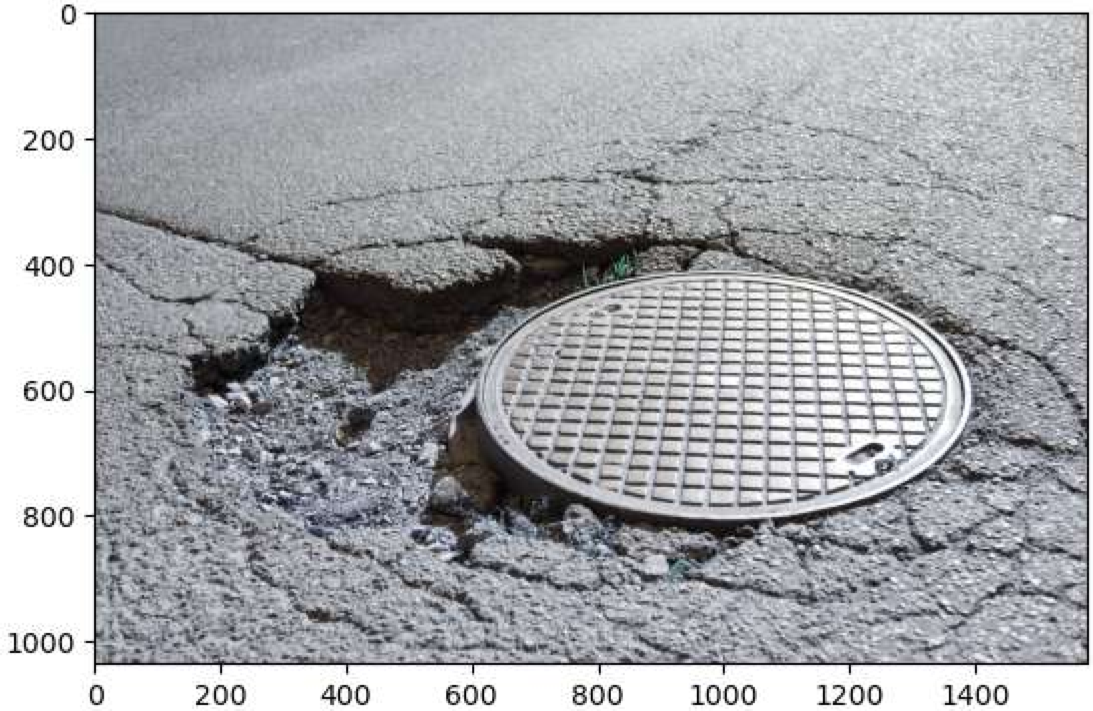
0
200
400
600
800
1000
0
200
400
600
800
1000
1200
1400

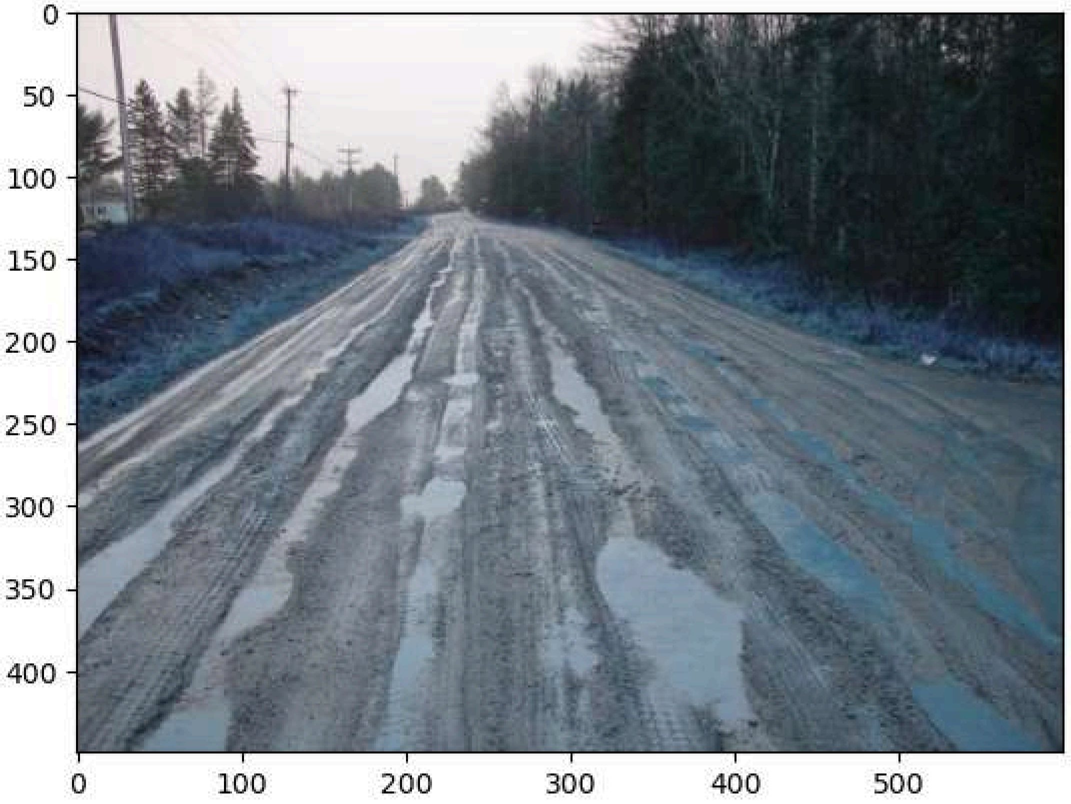
0
50
100
150
200
250
300
350
400
0
100
200
300
400
500

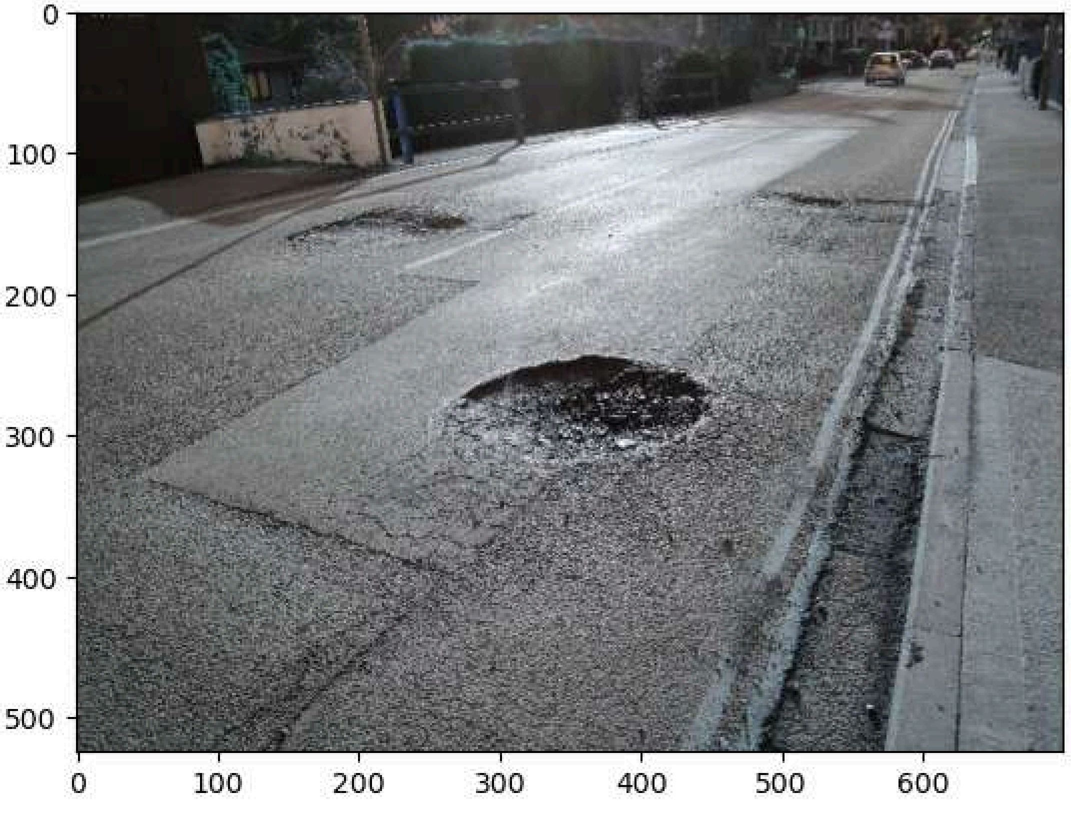
0
100
200
300
400
500
0
100
200
300
400
500
600

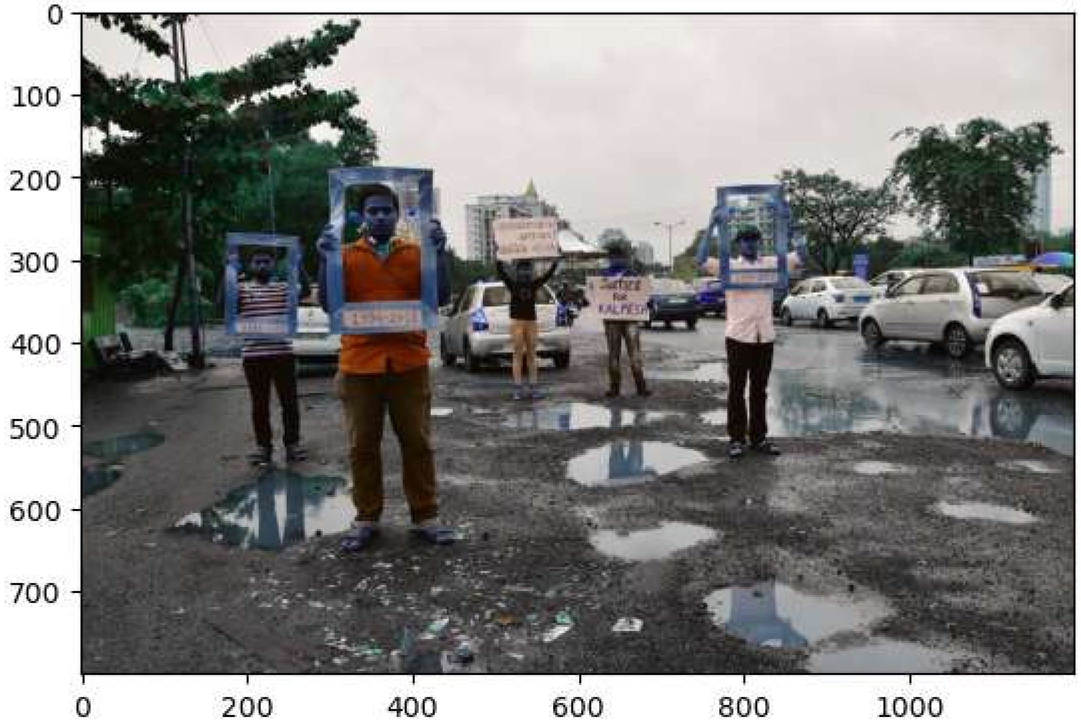
0
100
200
300
400
500
600
700
0
200
400
600
800
1000

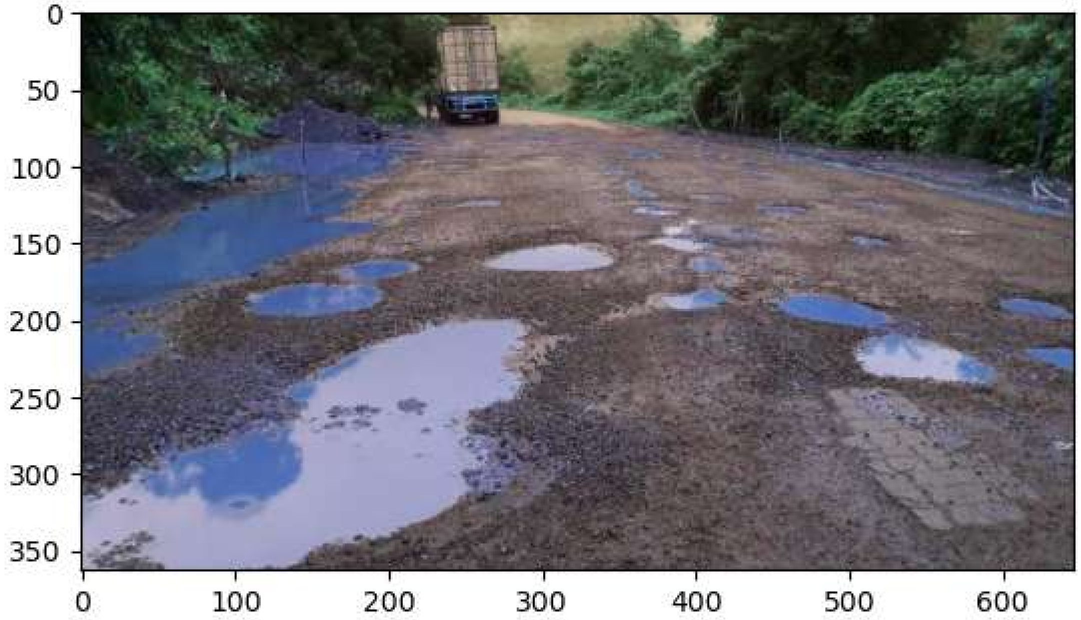
0
50
100
150
200
250
300
350
0
100
200
300
400
500
600

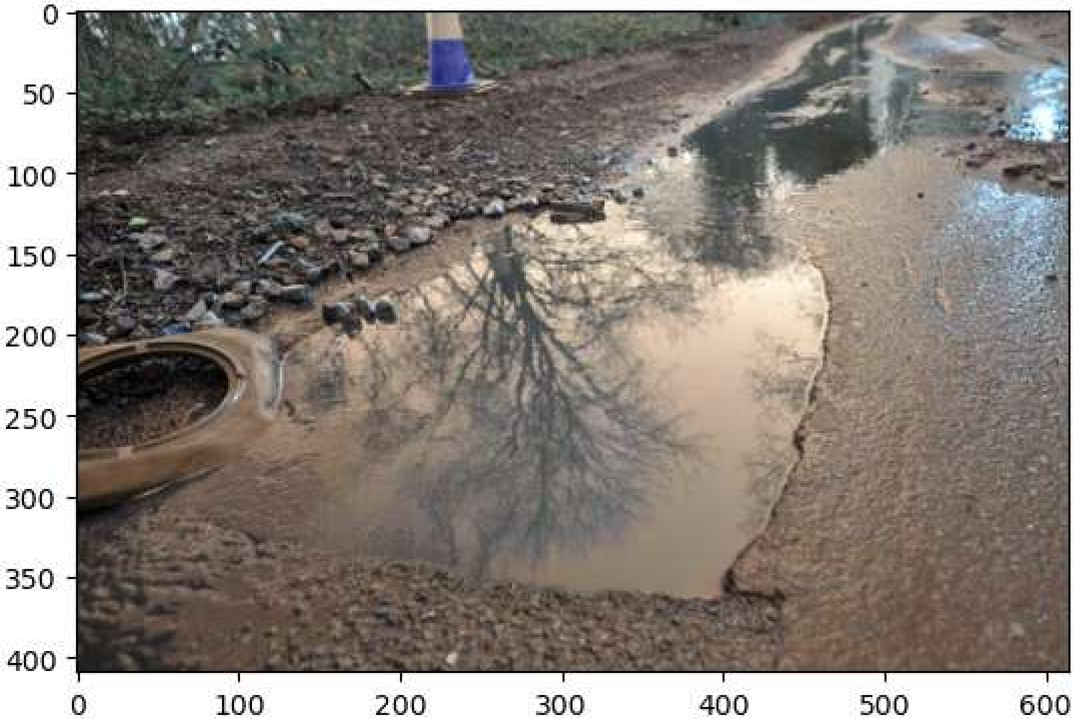
0
50
100
150
200
250
300
350
400
0
100
200
300
400
500
600

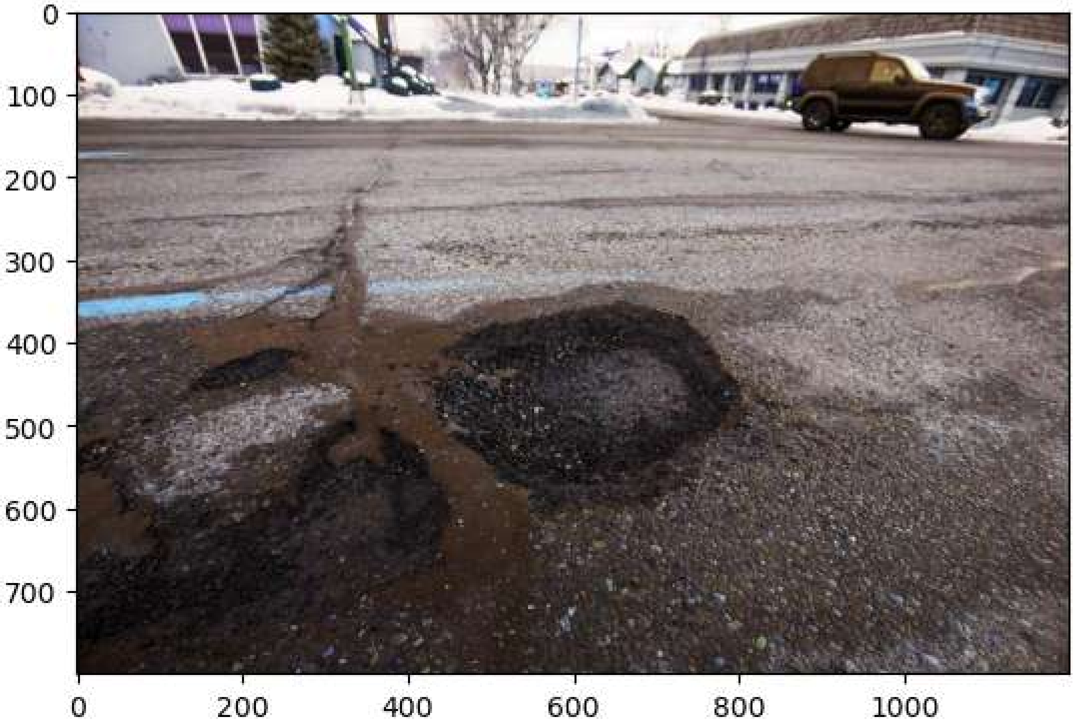
0
100
200
300
400
500
600
700
0
200
400
600
800
1000

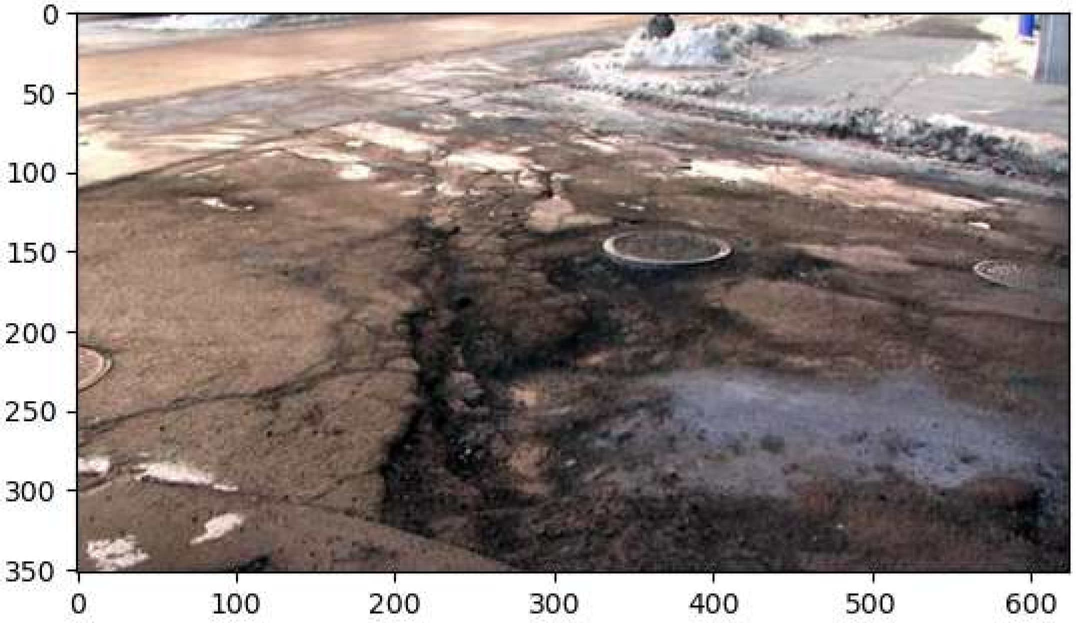
0
50
100
150
200
250
300
350
0 100 200 300 400 500 600

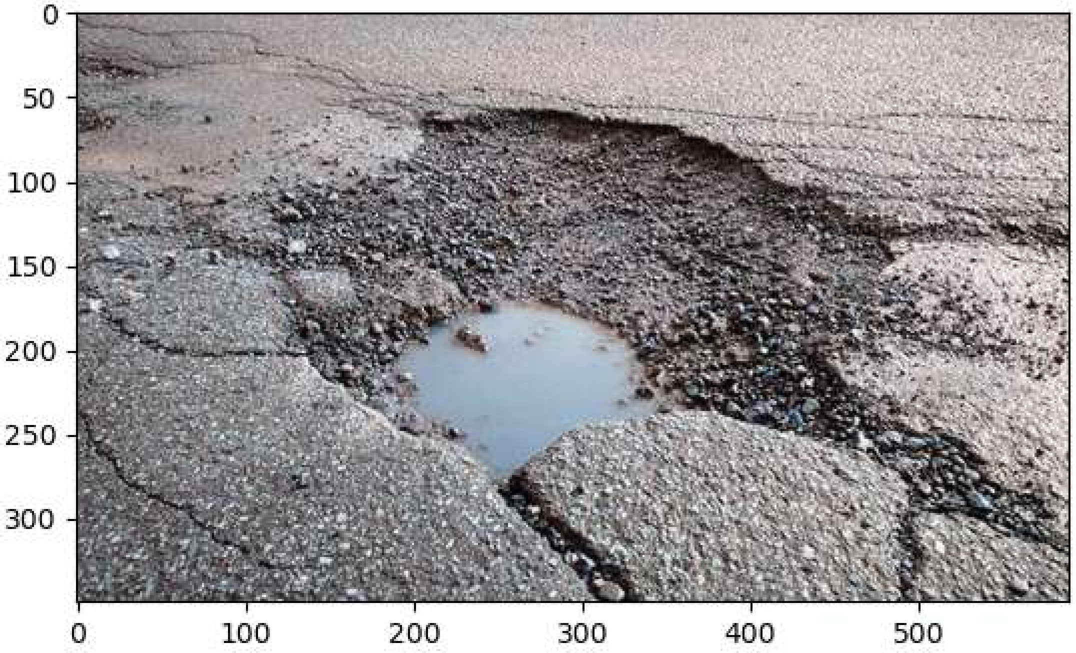
0
50
100
150
200
250
300
0 100 200 300 400 500

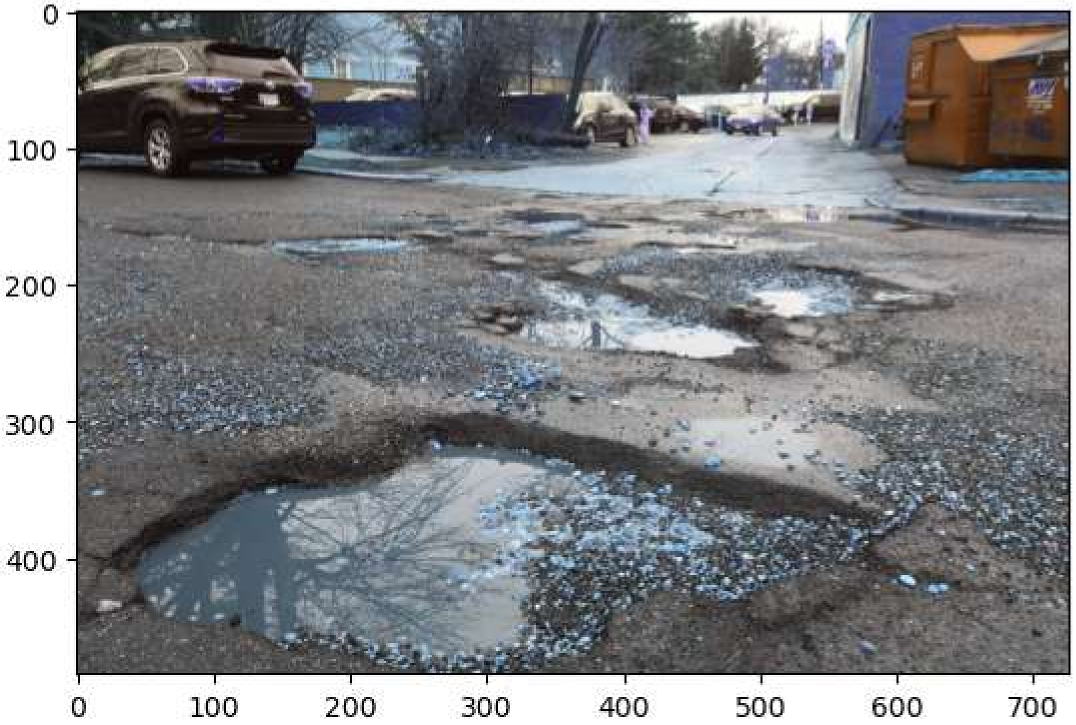
0
100
200
300
400
0
100
200
300
400
500
600
700

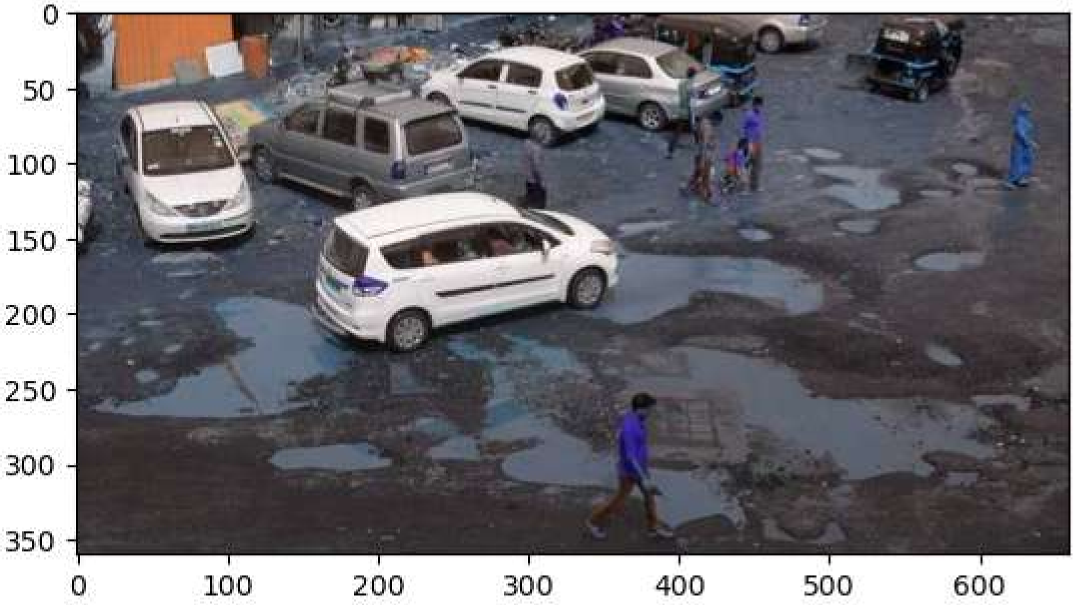
0
50
100
150
200
250
300
350
0
100
200
300
400
500
600

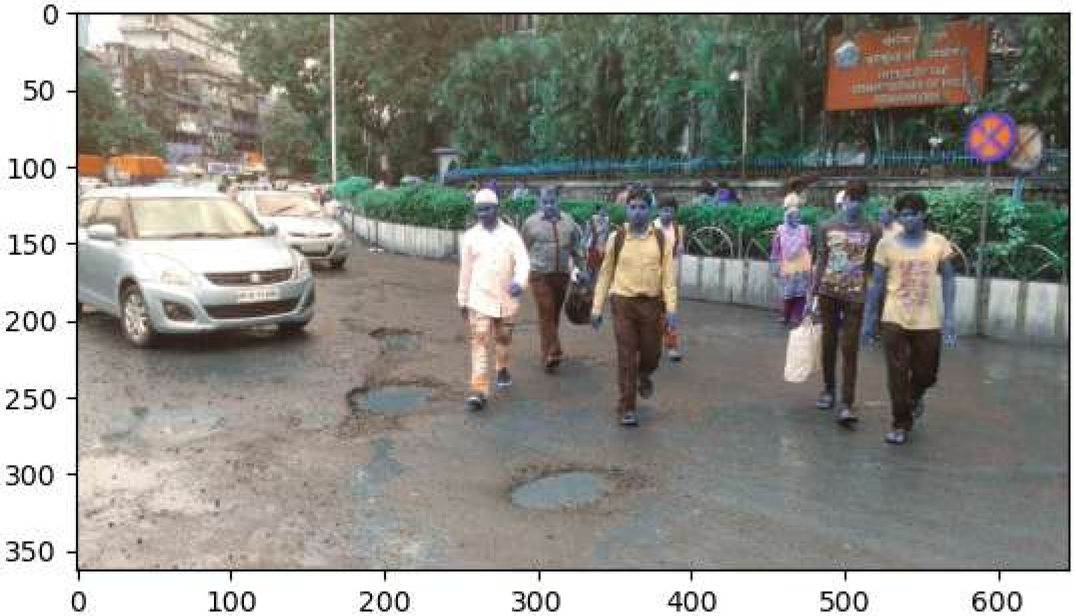
0
50
100
150
200
250
300
350
0
100
200
300
400
500
600

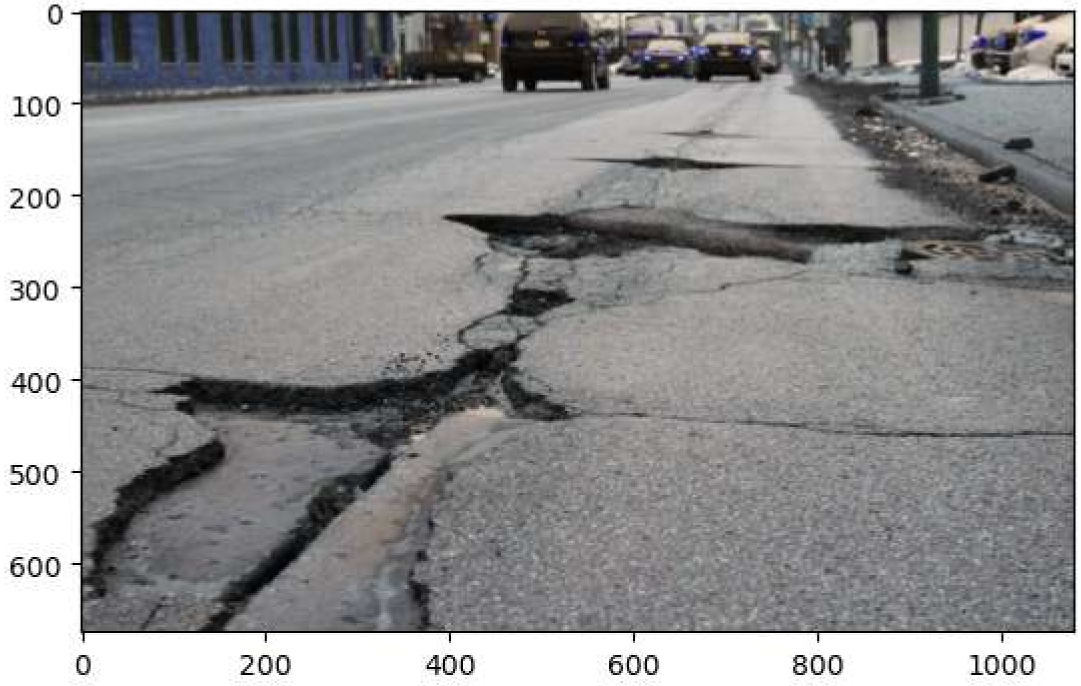
0
100
200
300
400
500
600
0 200 400 600 800 1000

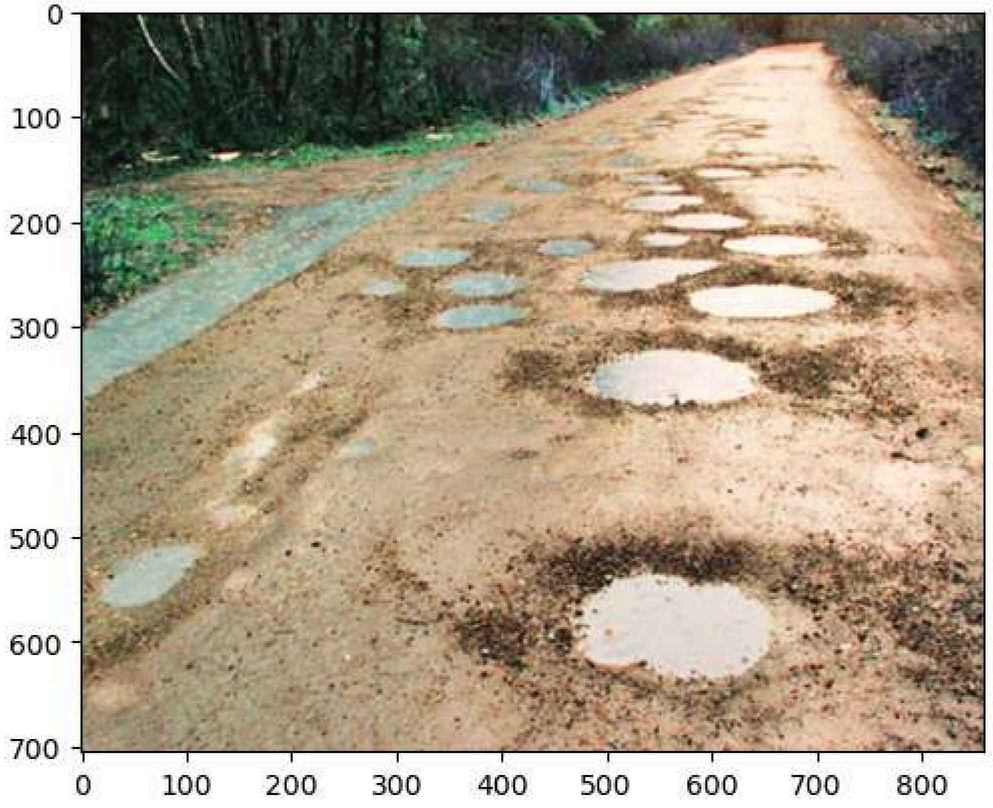
0
100
200
300
400
500
600
700
0 100 200 300 400 500 600 700 800

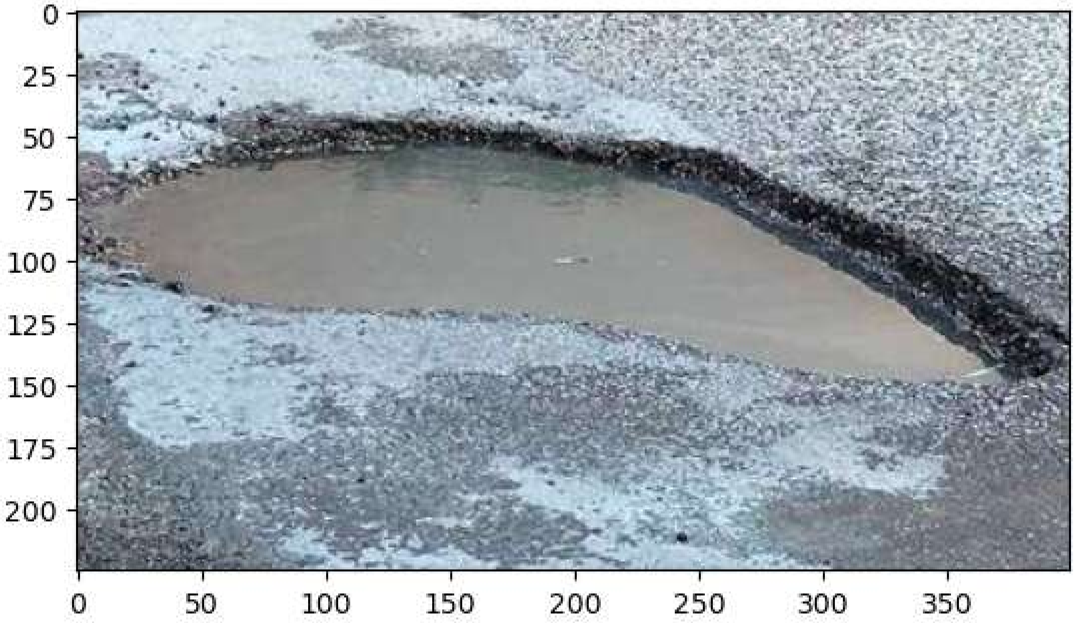
0
25
50
75
100
125
150
175
200
0 50 100 150 200 250 300 350

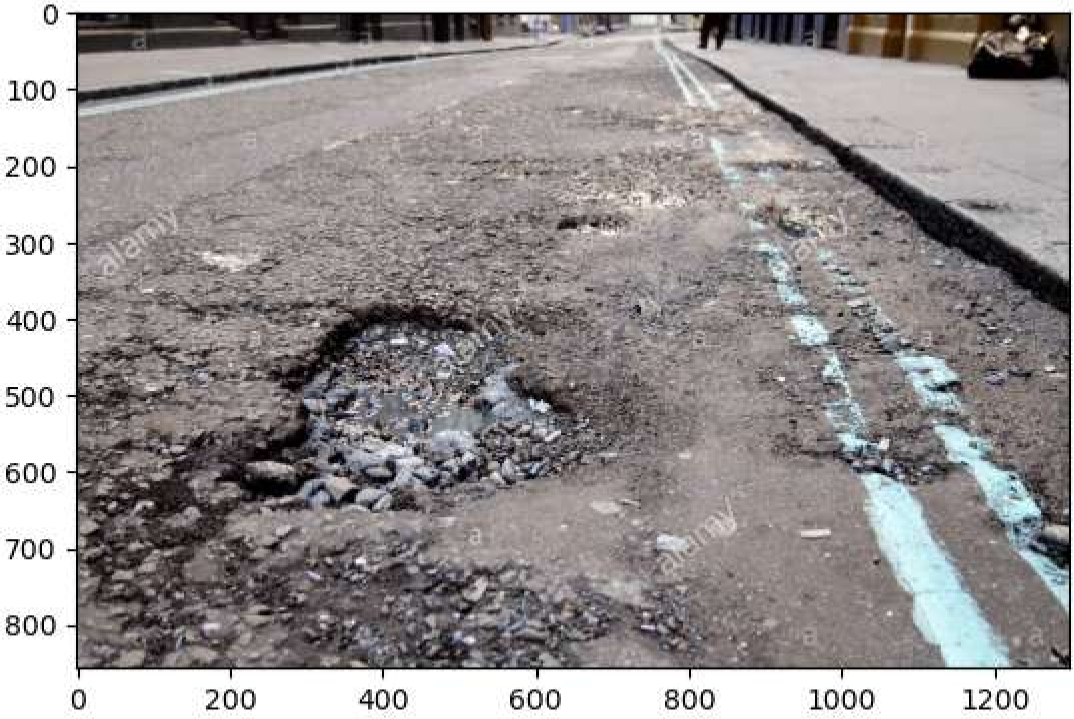
0
100
200
300
400
500
600
700
800
0 200 400 600 800 1000 1200
alamy

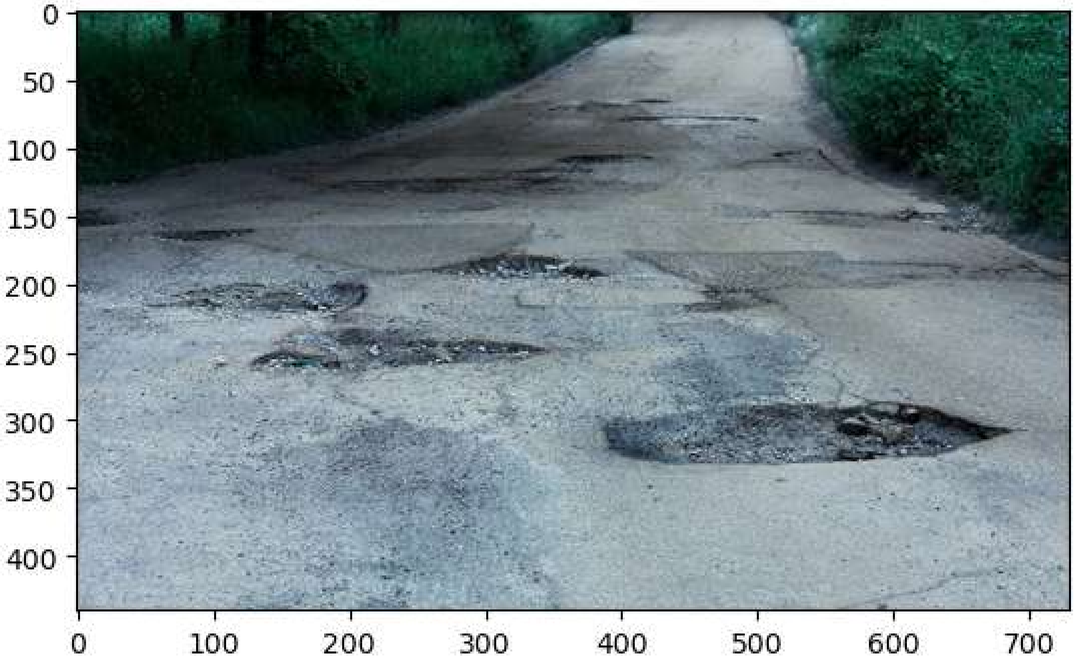
0
50
100
150
200
250
300
350
400
0 100 200 300 400 500 600 700

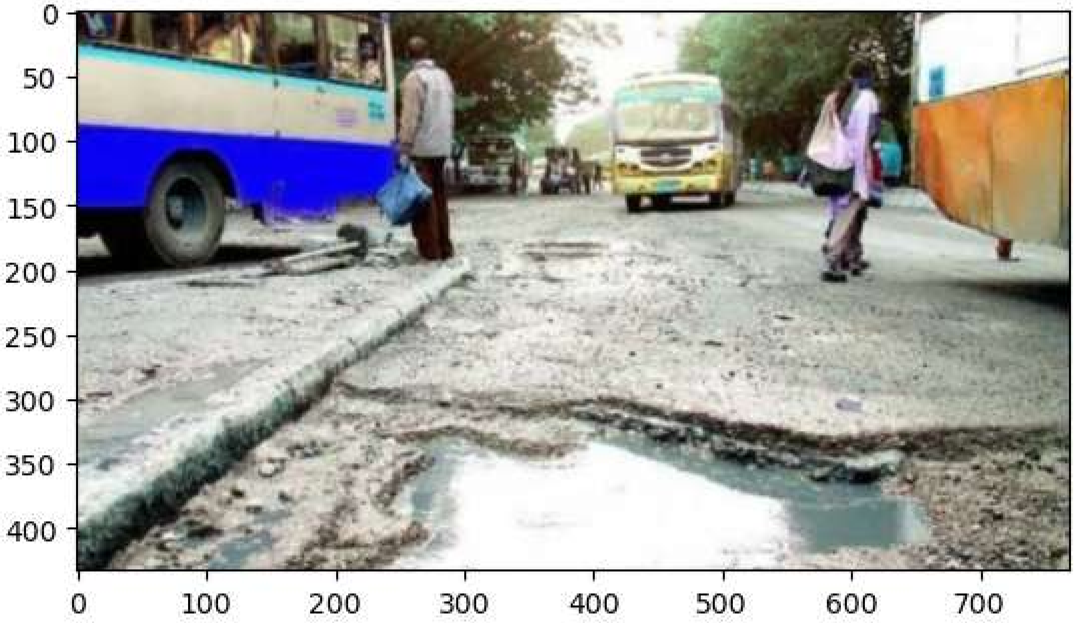
0
50
100
150
200
250
300
350
400
0 100 200 300 400 500 600 700

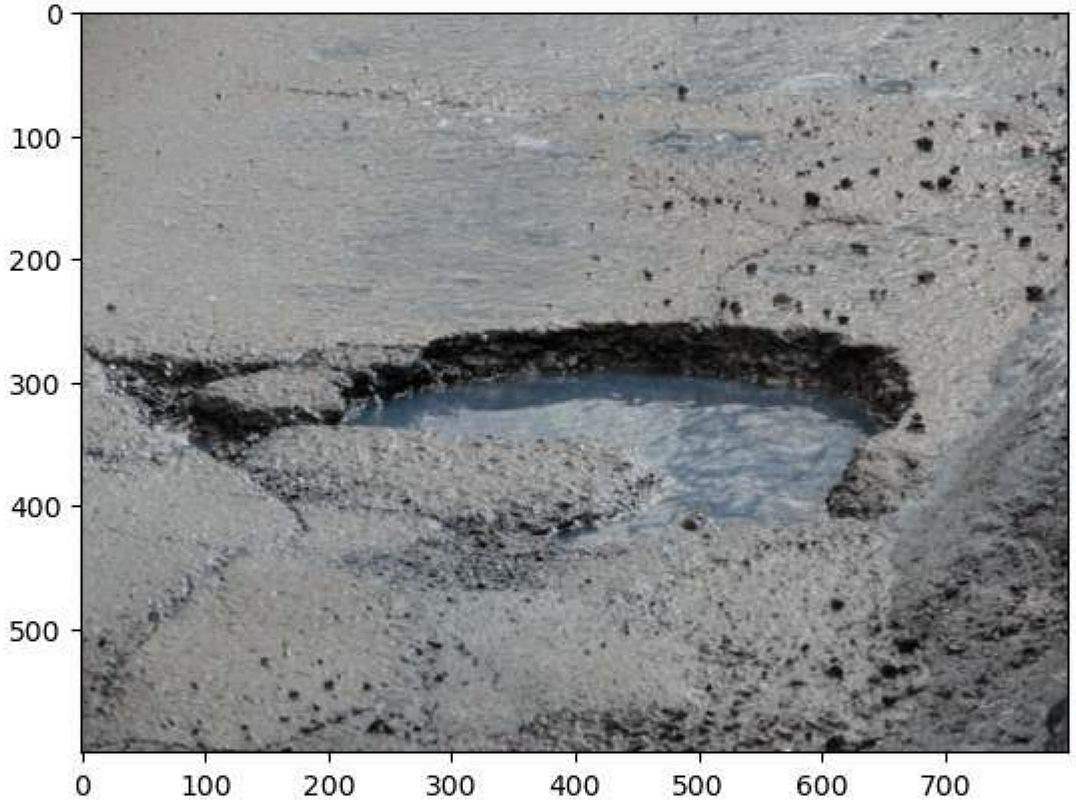
0
100
200
300
400
500
0
100
200
300
400
500
600
700

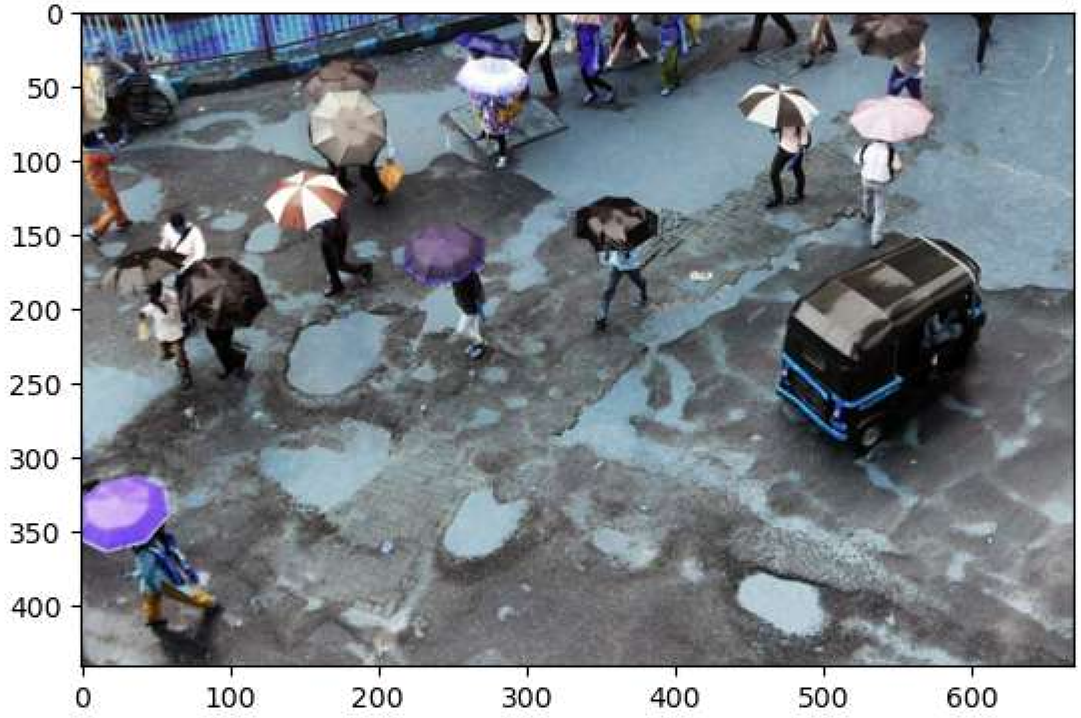
0
50
100
150
200
250
300
350
400
0
100
200
300
400
500
600

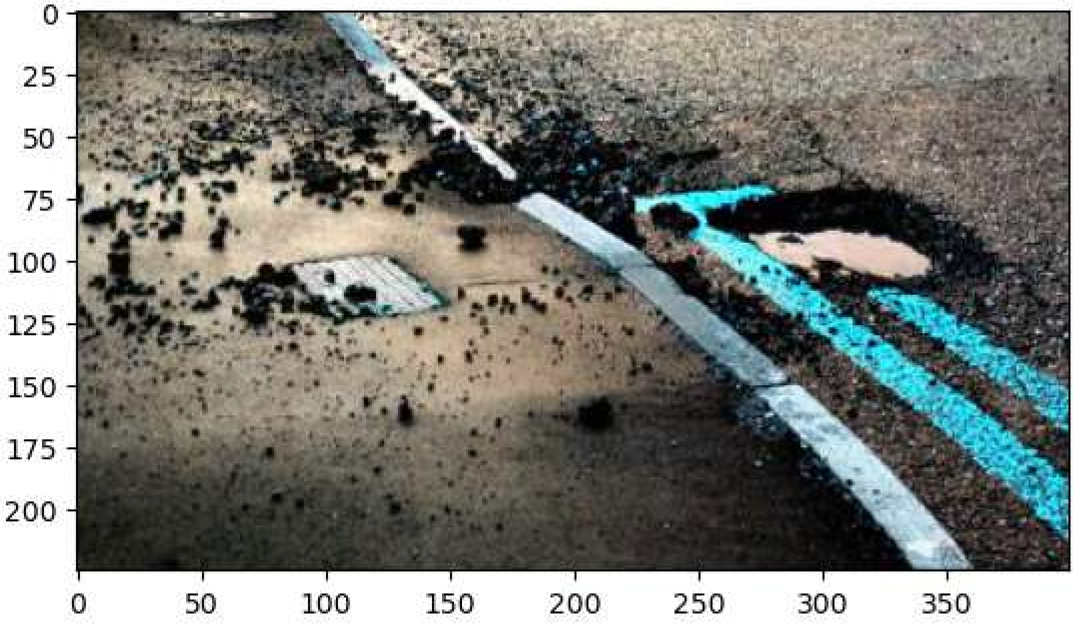
0
25
50
75
100
125
150
175
200
0 50 100 150 200 250 300 350

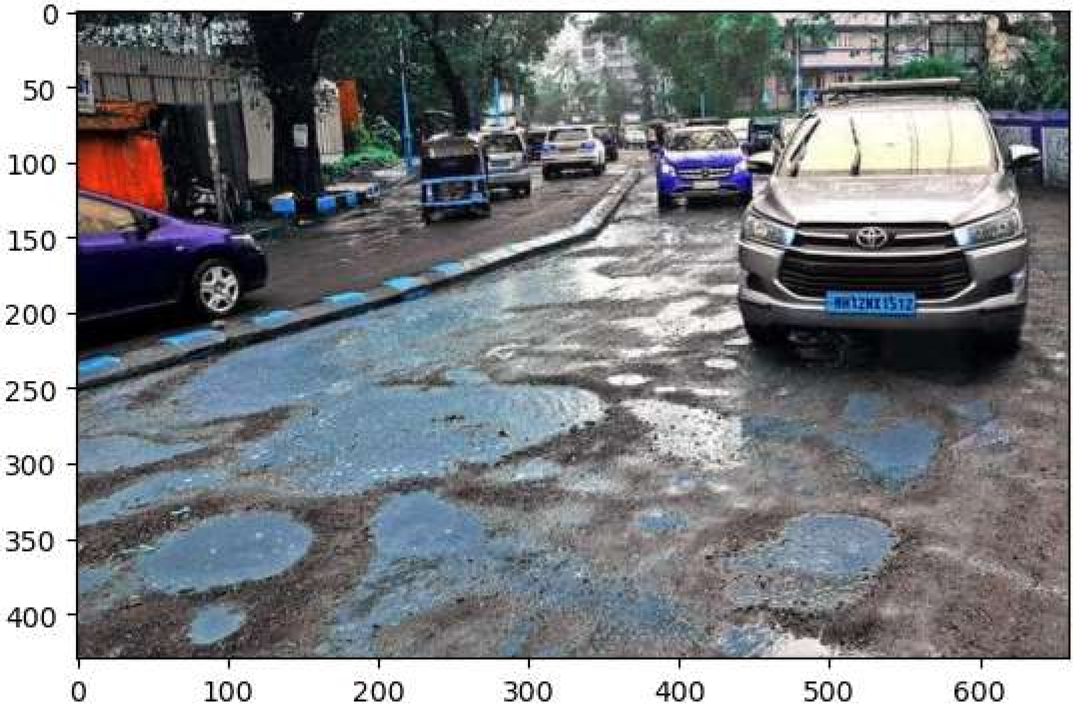
0
50
100
150
200
250
300
350
400
0 100 200 300 400 500 600

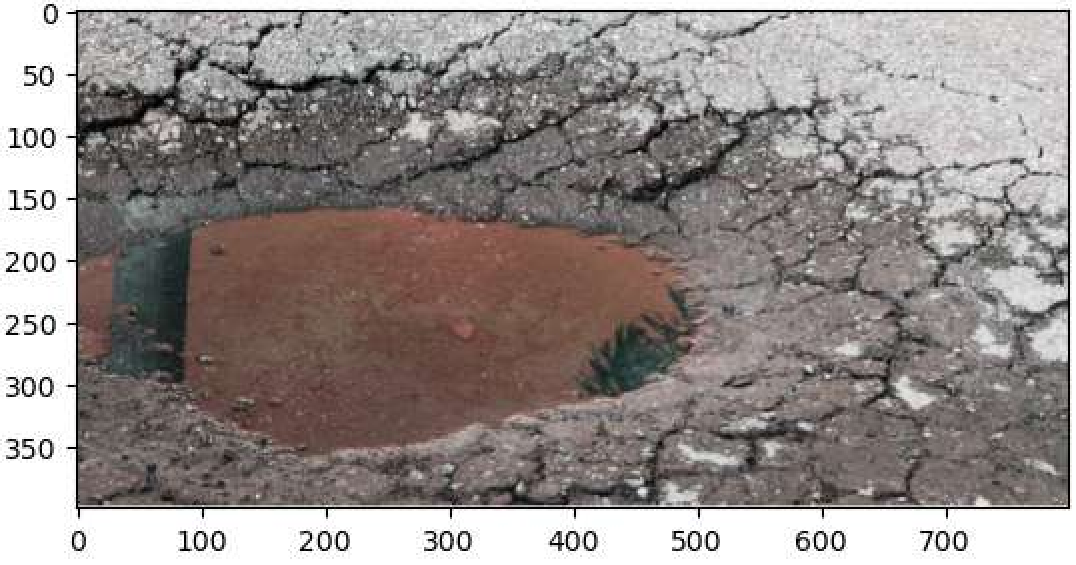
0
50
100
150
200
250
300
350
0 100 200 300 400 500 600 700

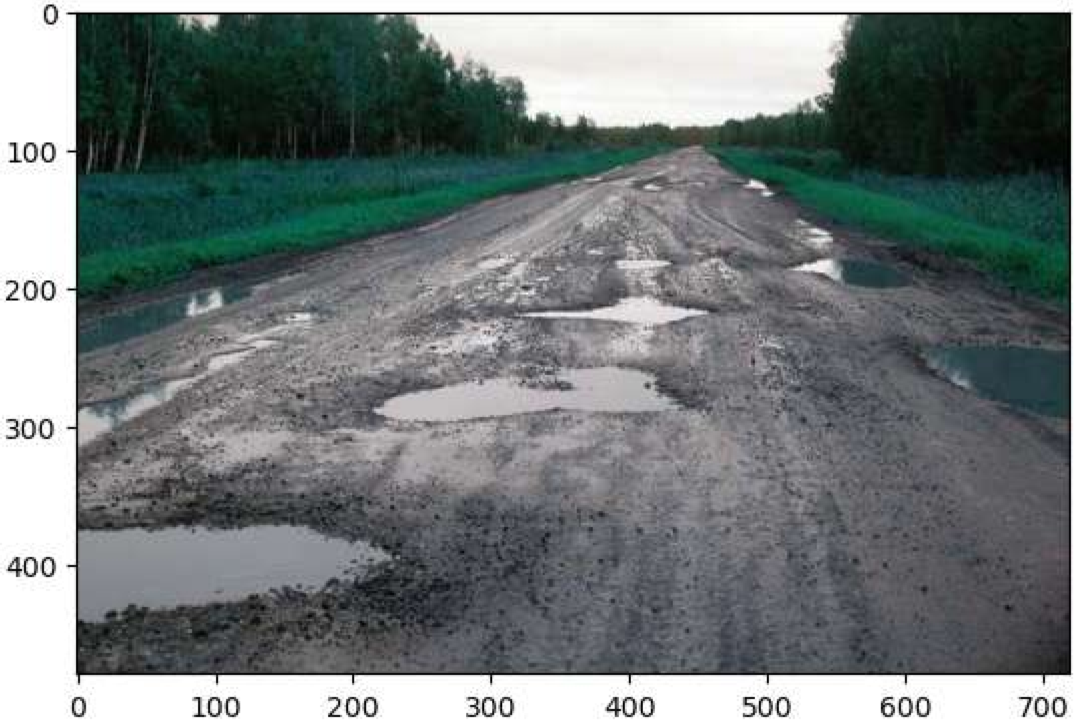
0
100
200
300
400
0 100 200 300 400 500 600 700

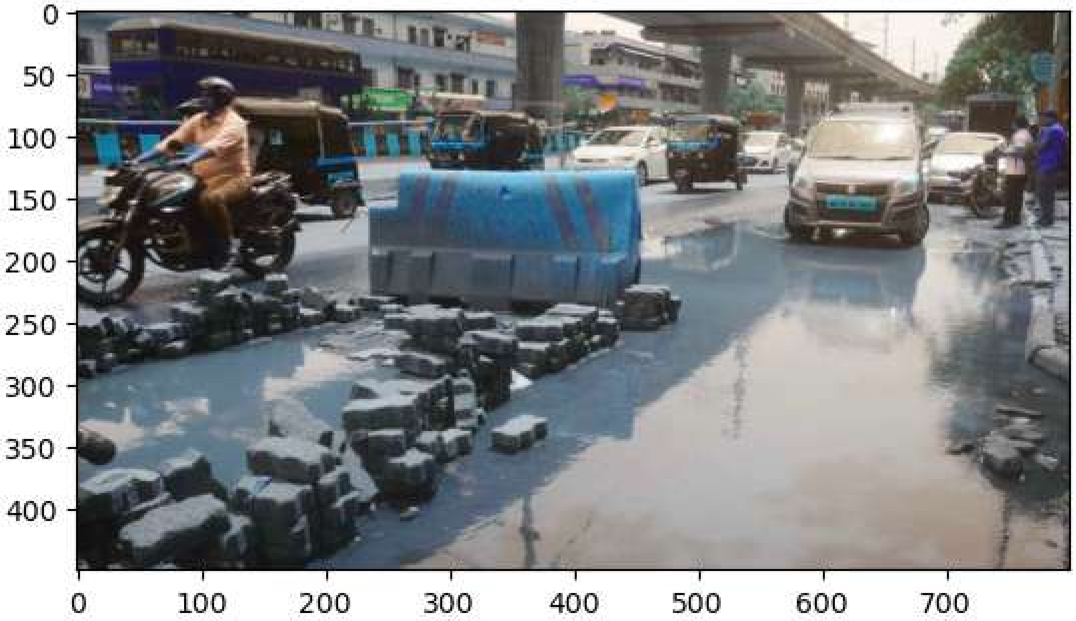
0
50
100
150
200
250
300
350
400
0 100 200 300 400 500 600 700

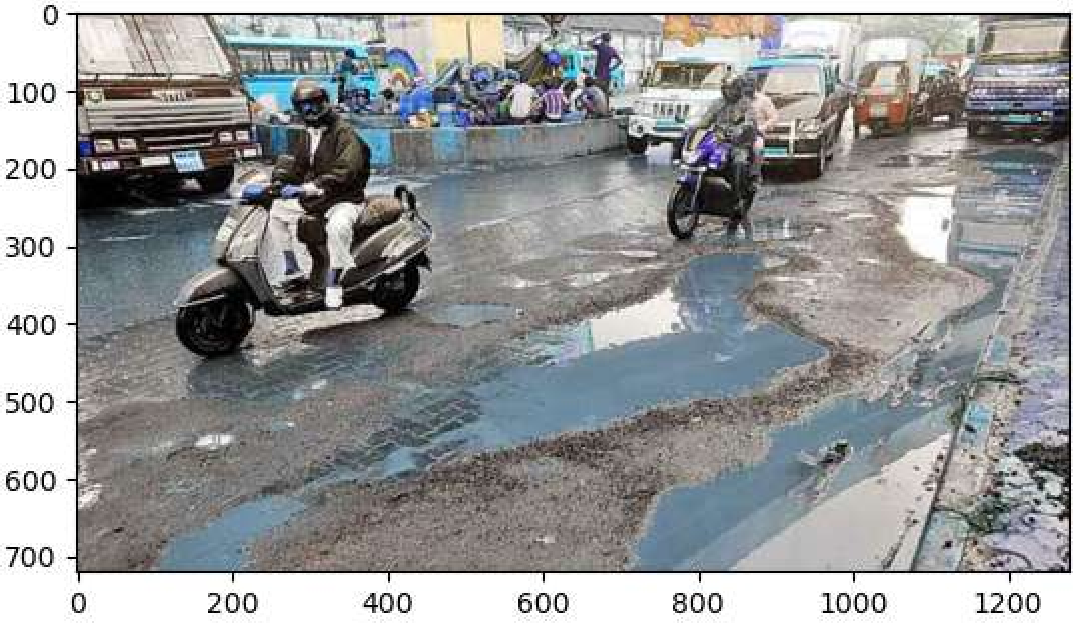
0
100
200
300
400
500
600
700
0 200 400 600 800 1000 1200

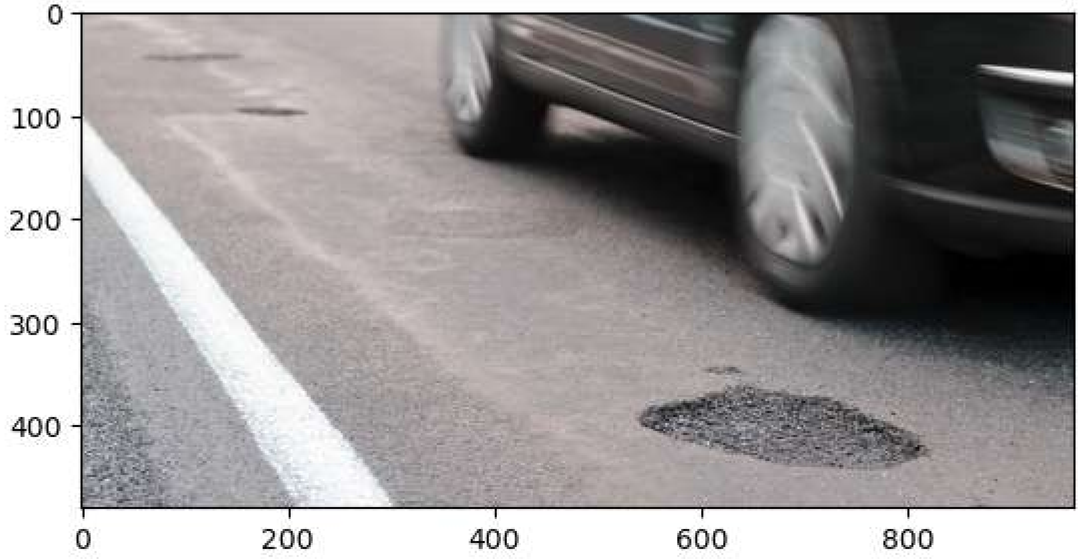
0
100
200
300
400
0 200 400 600 800

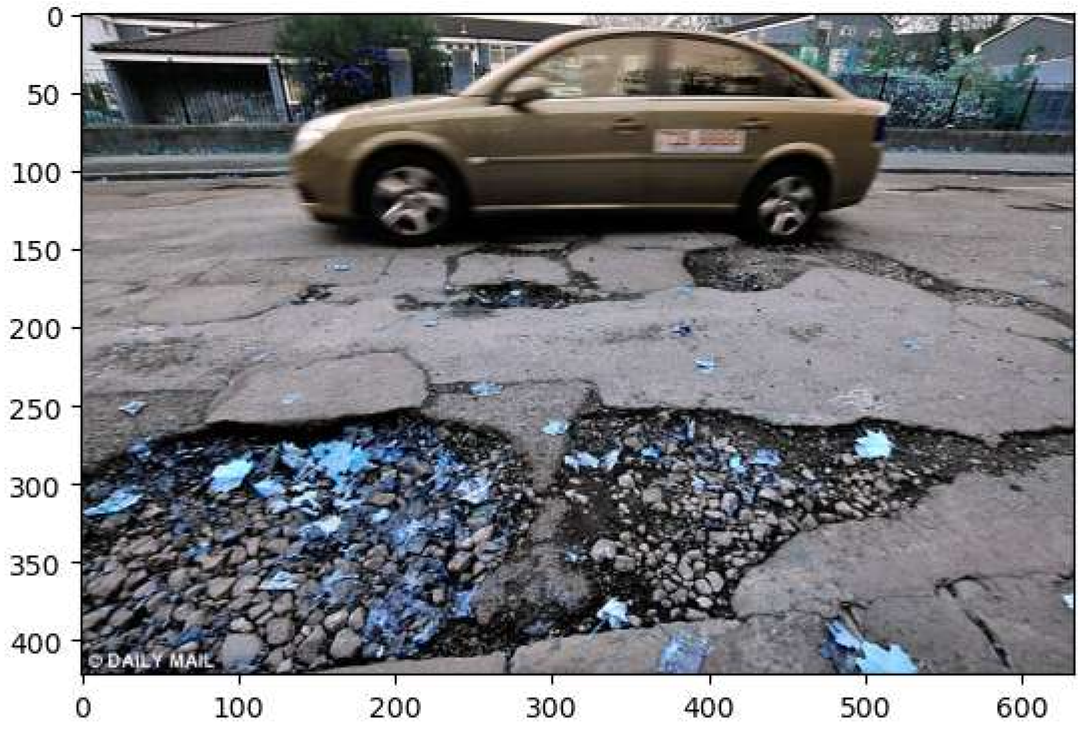
0
50
100
150
200
250
300
350
400
© DAILY MAIL
0 100 200 300 400 500 600

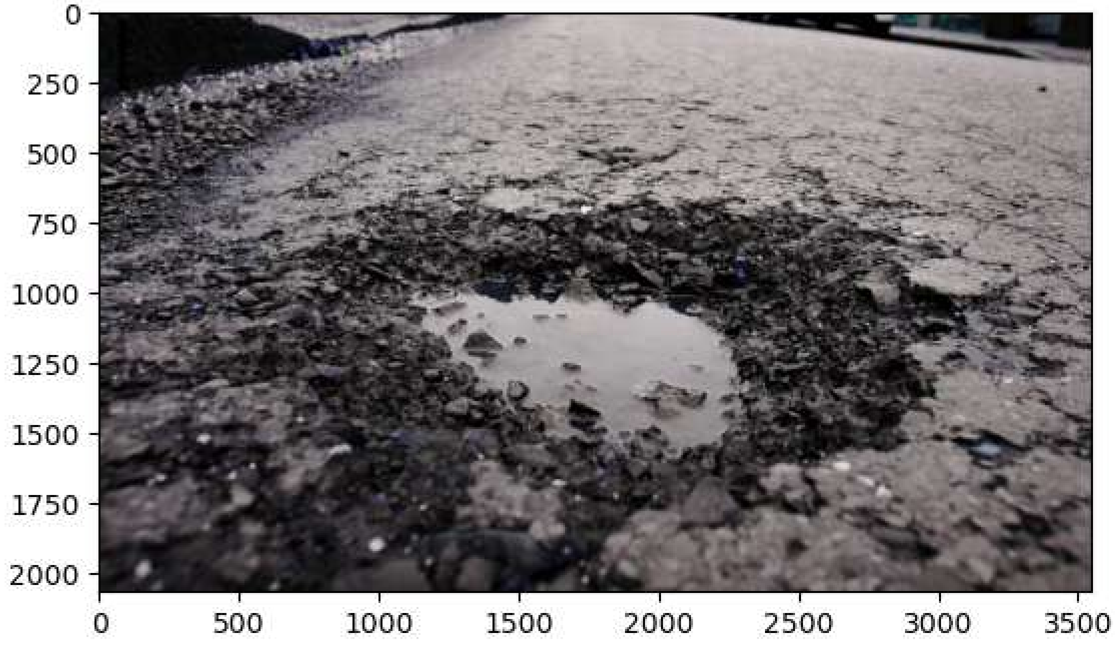
0
250
500
750
1000
1250
1500
1750
2000
0 500 1000 1500 2000 2500 3000 3500

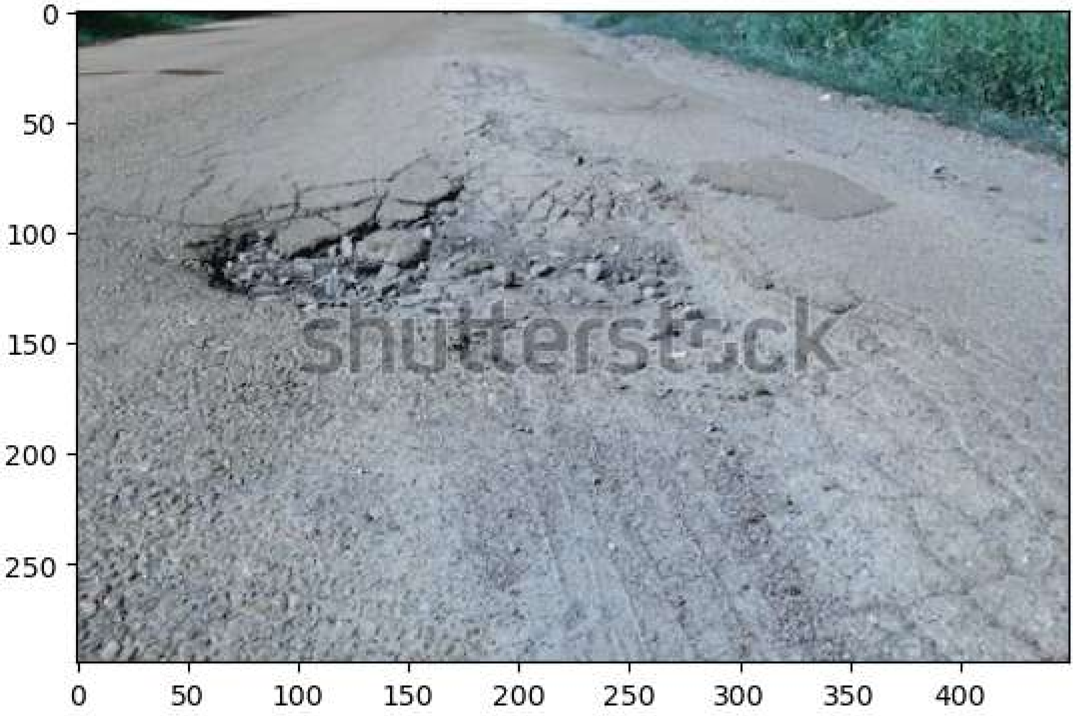
0
50
100
150
200
250
0
50
100
150
200
250
300
350
400
shutterstock

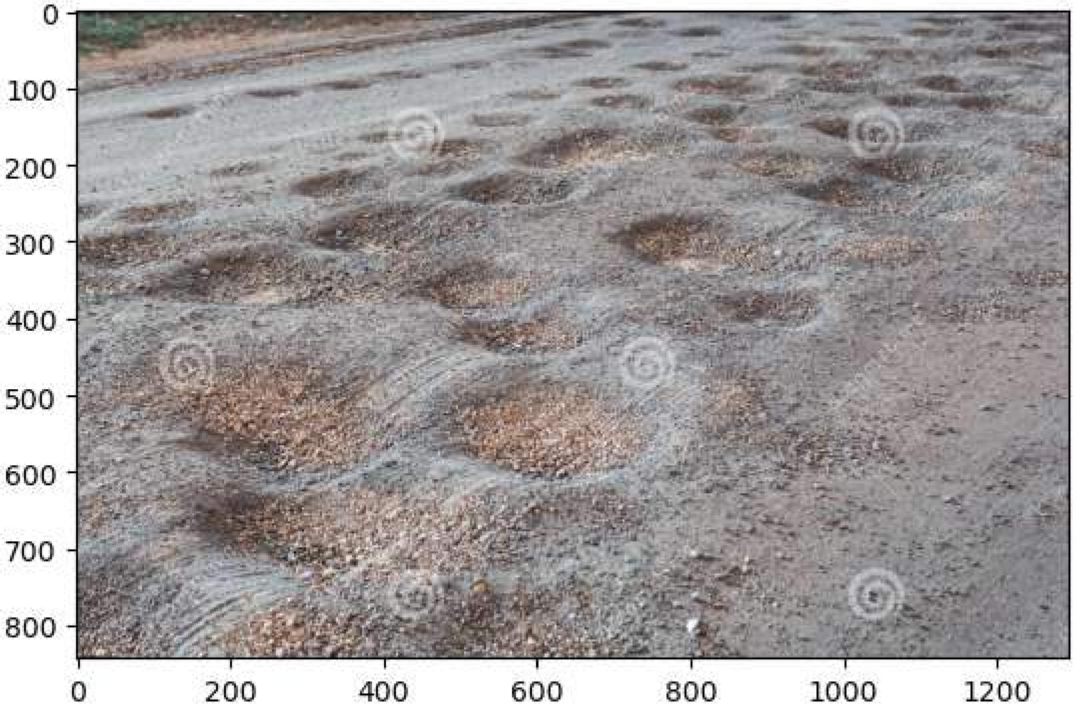
0
100
200
300
400
500
600
700
800
0
200
400
600
800
1000
1200

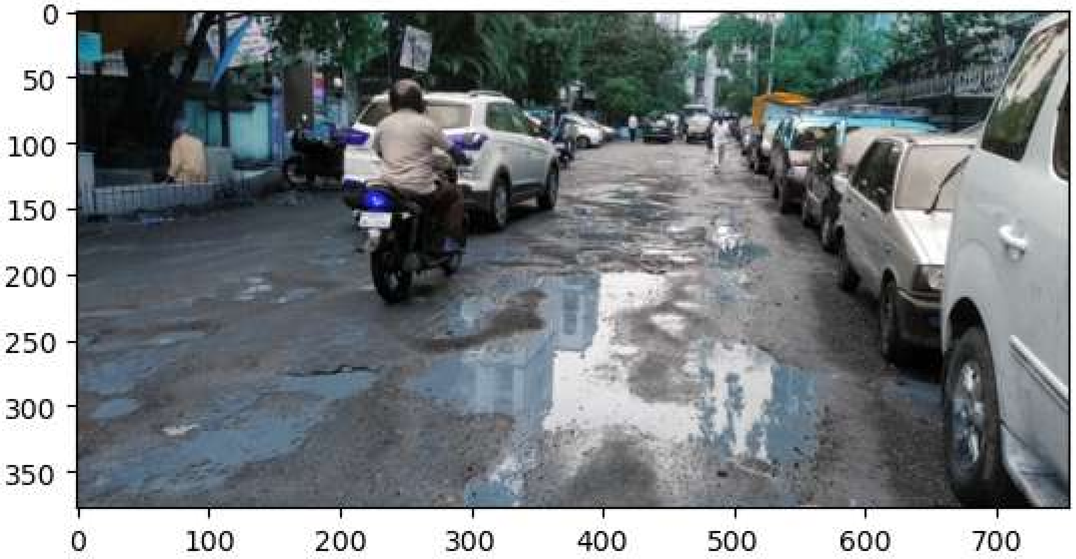
0
50
100
150
200
250
300
350
0
100
200
300
400
500
600
700

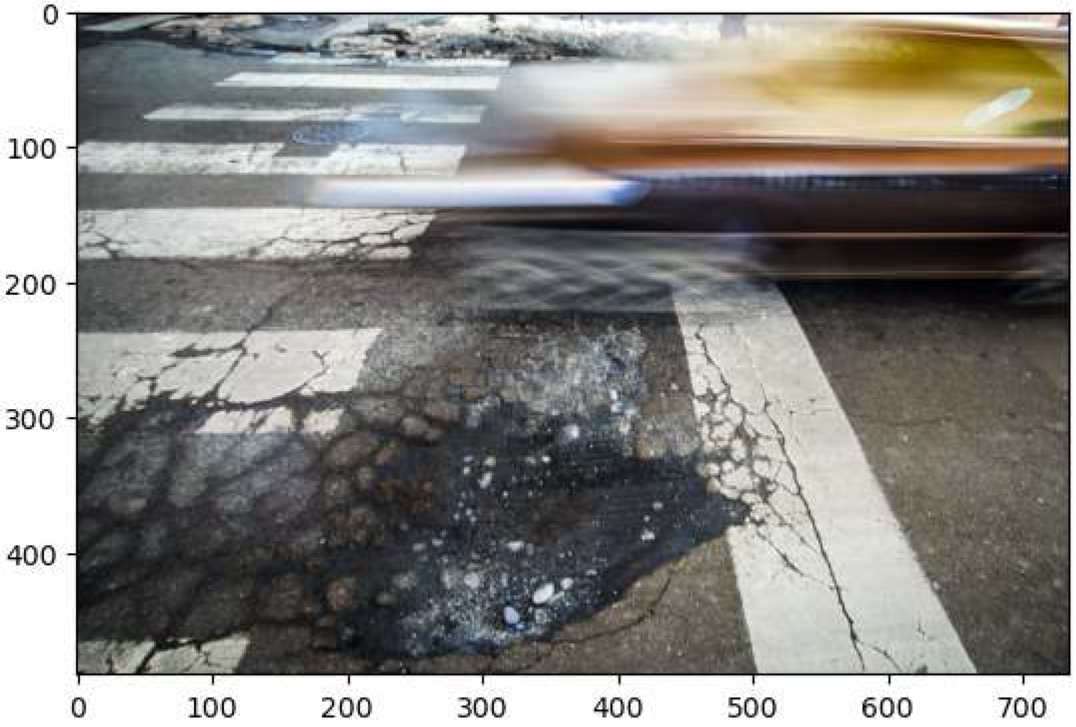
0
100
200
300
400
0
100
200
300
400
500
600
700

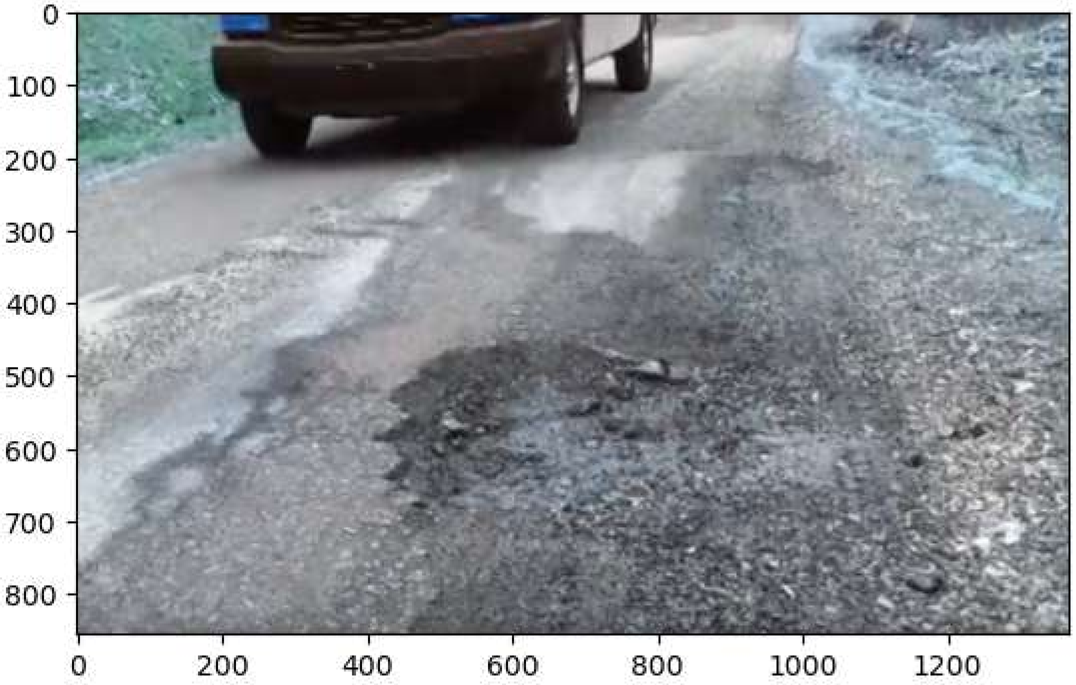
0
100
200
300
400
500
600
700
800
0
200
400
600
800
1000
1200

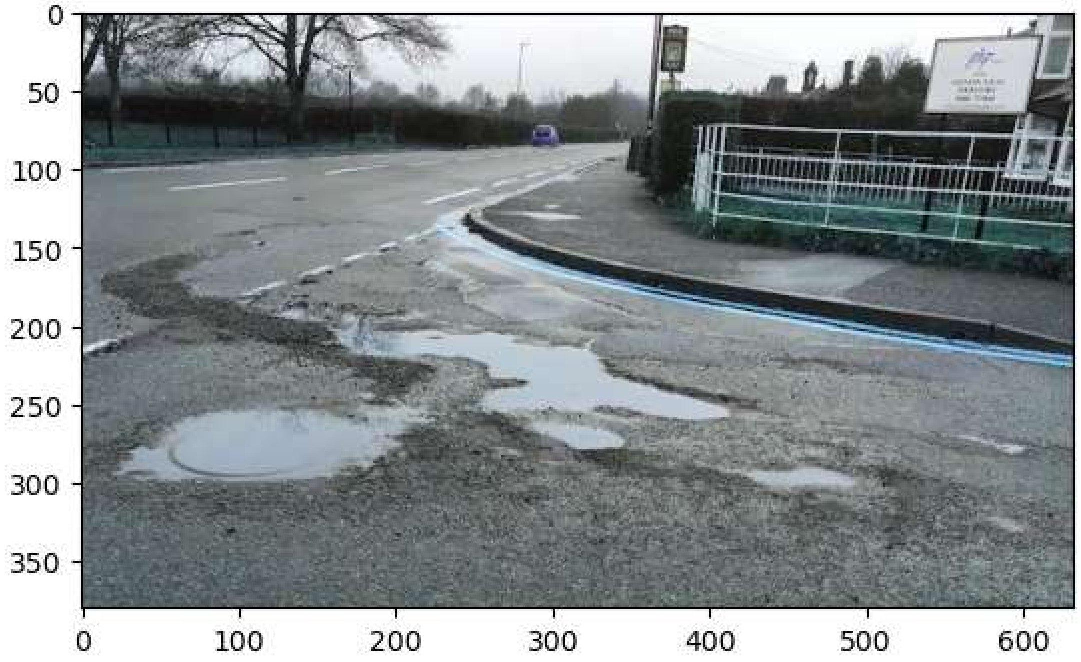
0
50
100
150
200
250
300
350
0 100 200 300 400 500 600

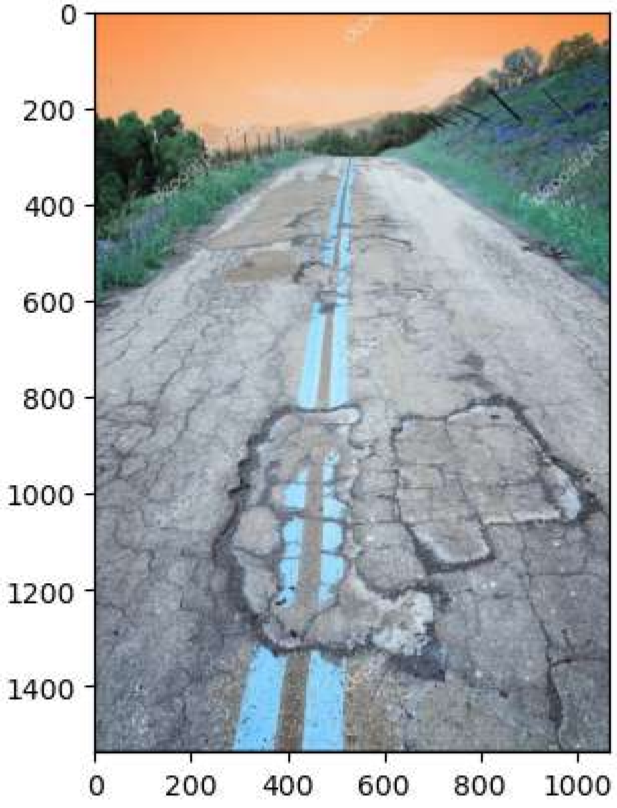
0
200
400
600
800
1000
1200
1400
0 200 400 600 800 1000

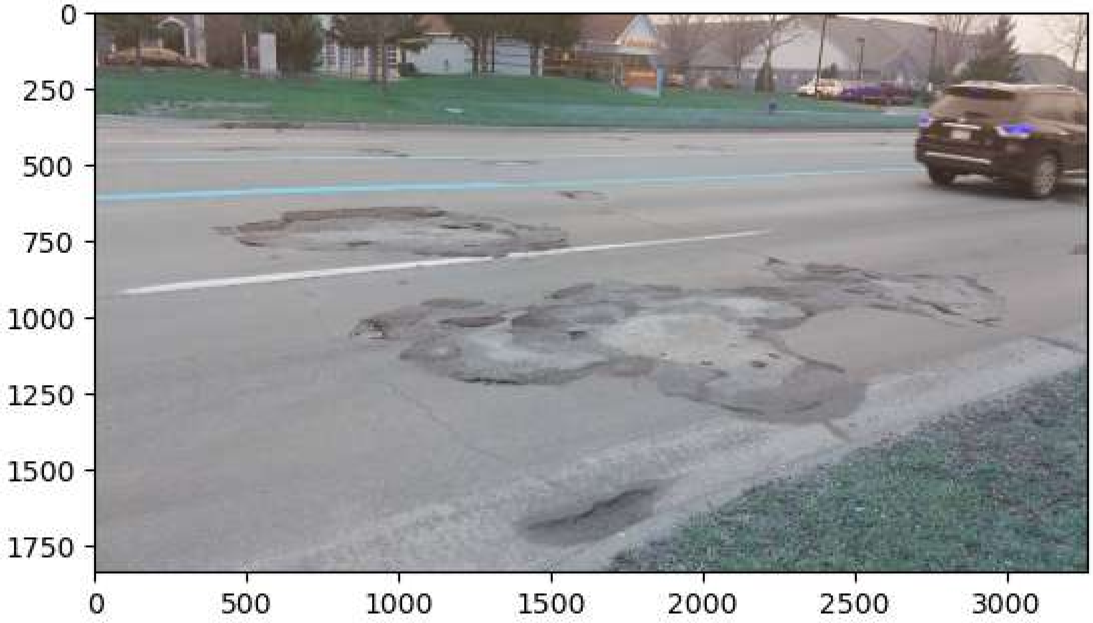
0
250
500
750
1000
1250
1500
1750
0
500
1000
1500
2000
2500
3000

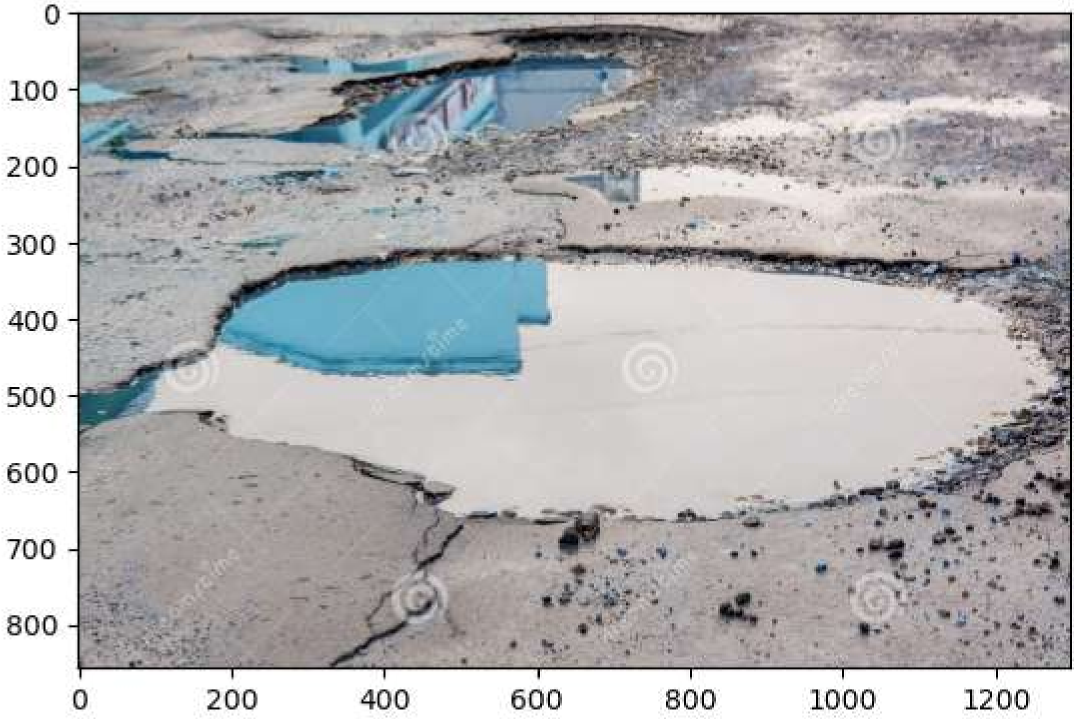
0
100
200
300
400
500
600
700
800
0
200
400
600
800
1000
1200

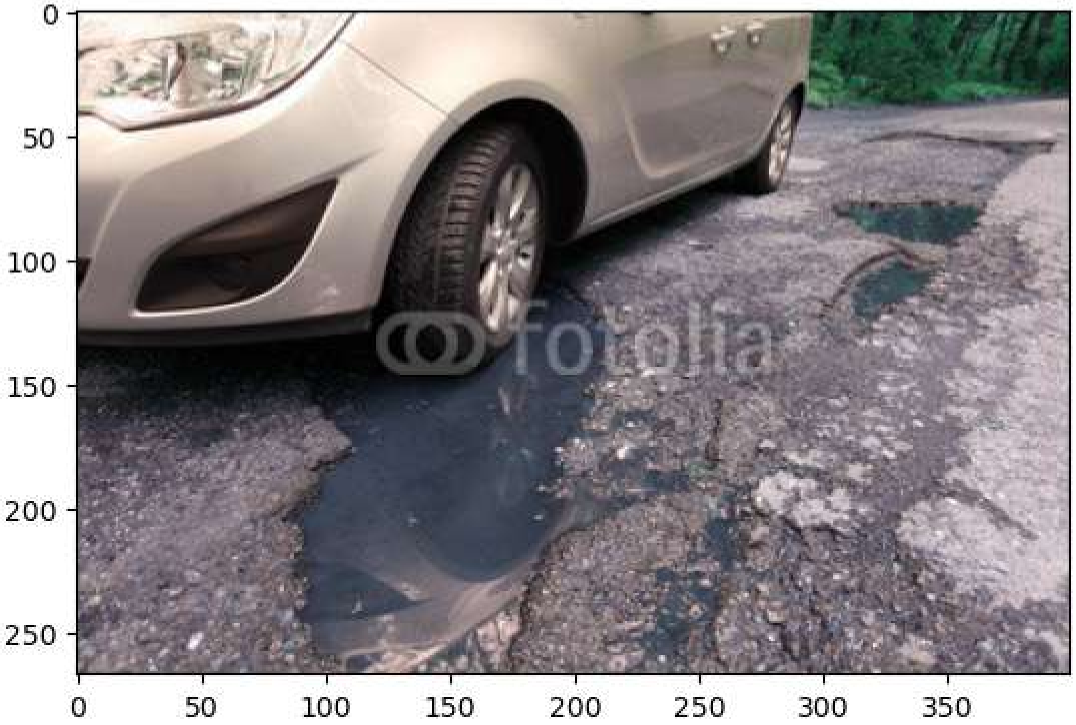
0
50
100
150
200
250
0 50 100 150 200 250 300 350
fotolia

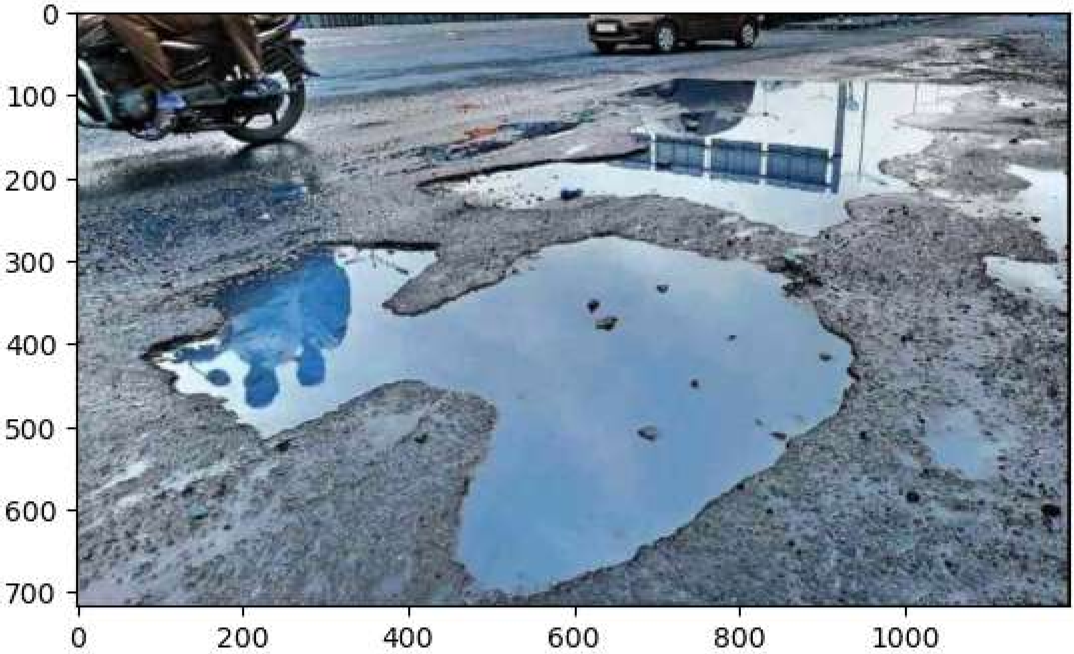
0
100
200
300
400
500
600
700
0 200 400 600 800 1000

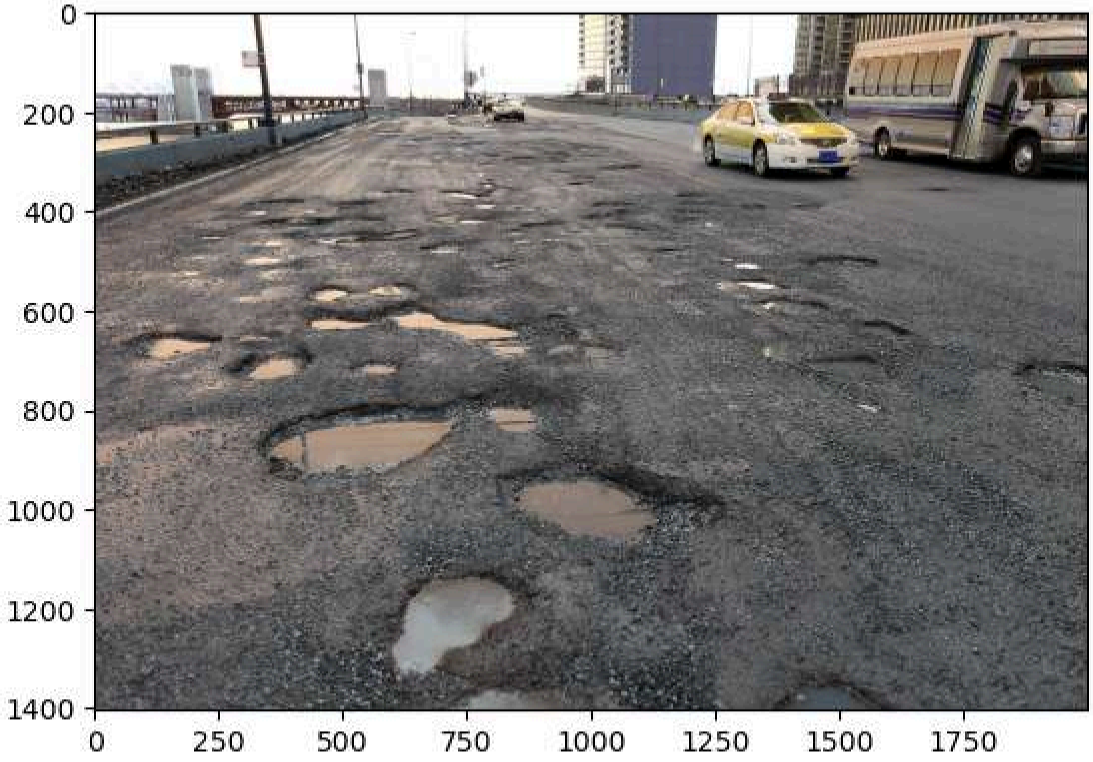
0
200
400
600
800
1000
1200
1400
0
250
500
750
1000
1250
1500
1750

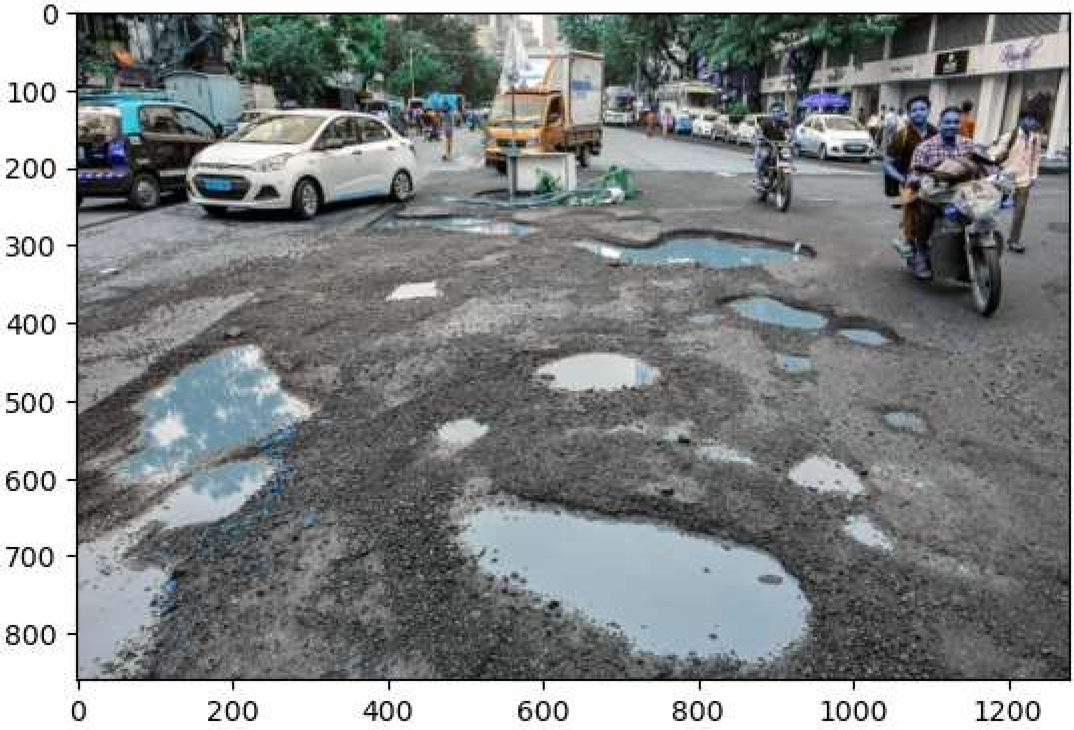
0
100
200
300
400
500
600
700
800
0
200
400
600
800
1000
1200

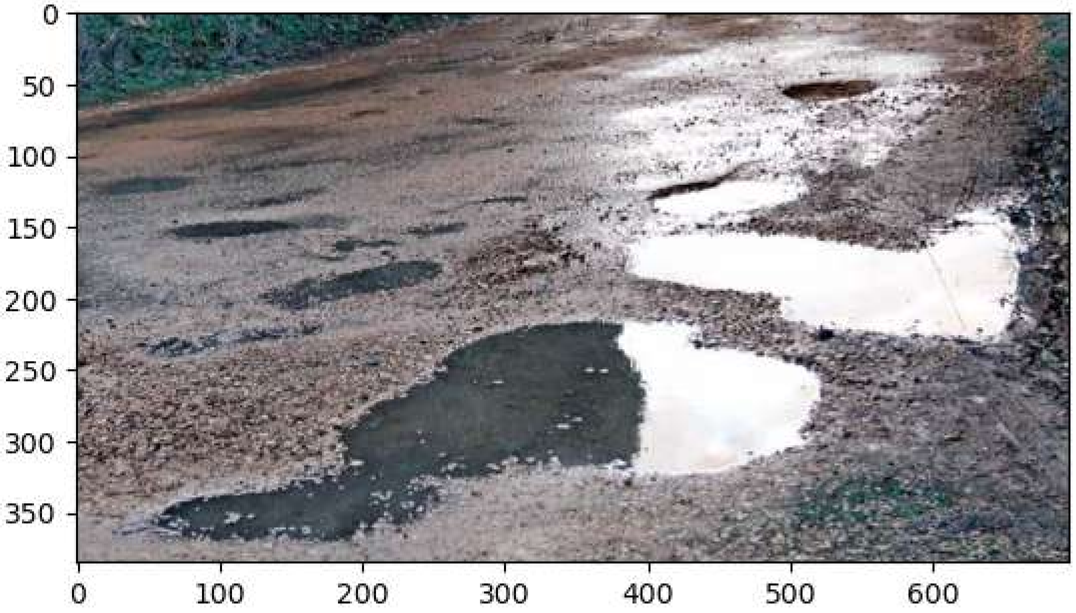
0
50
100
150
200
250
300
350
0 100 200 300 400 500 600

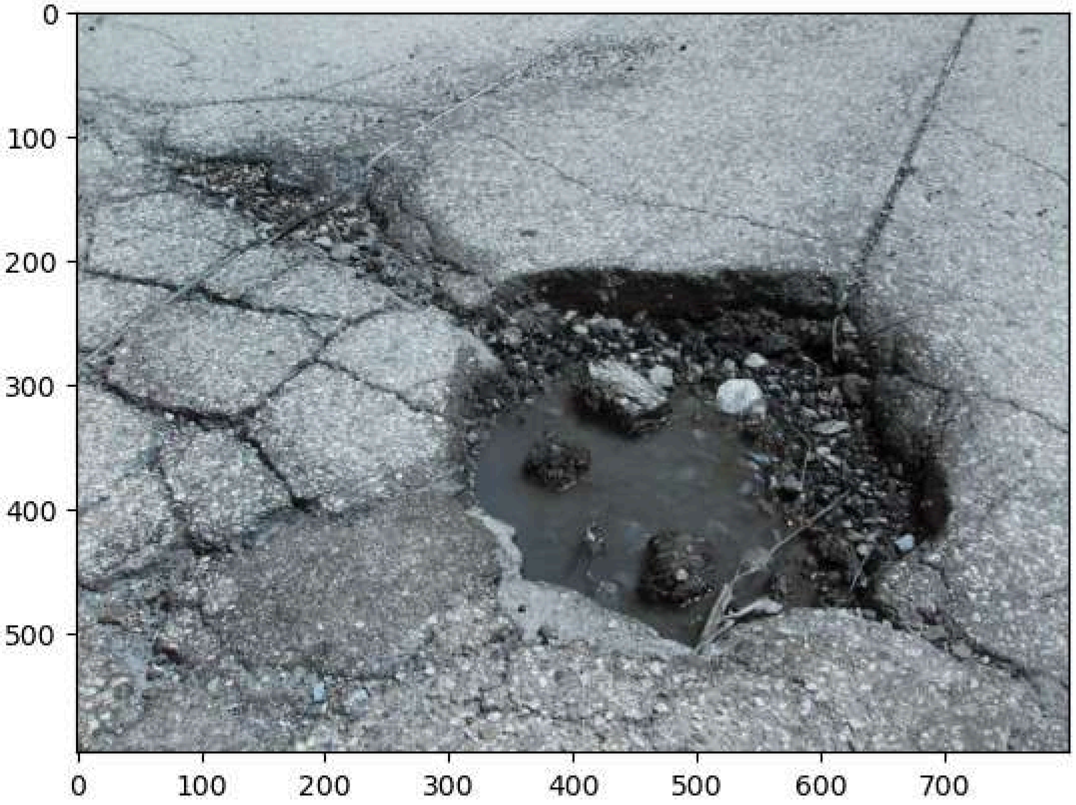
0
100
200
300
400
500
0 100 200 300 400 500 600 700

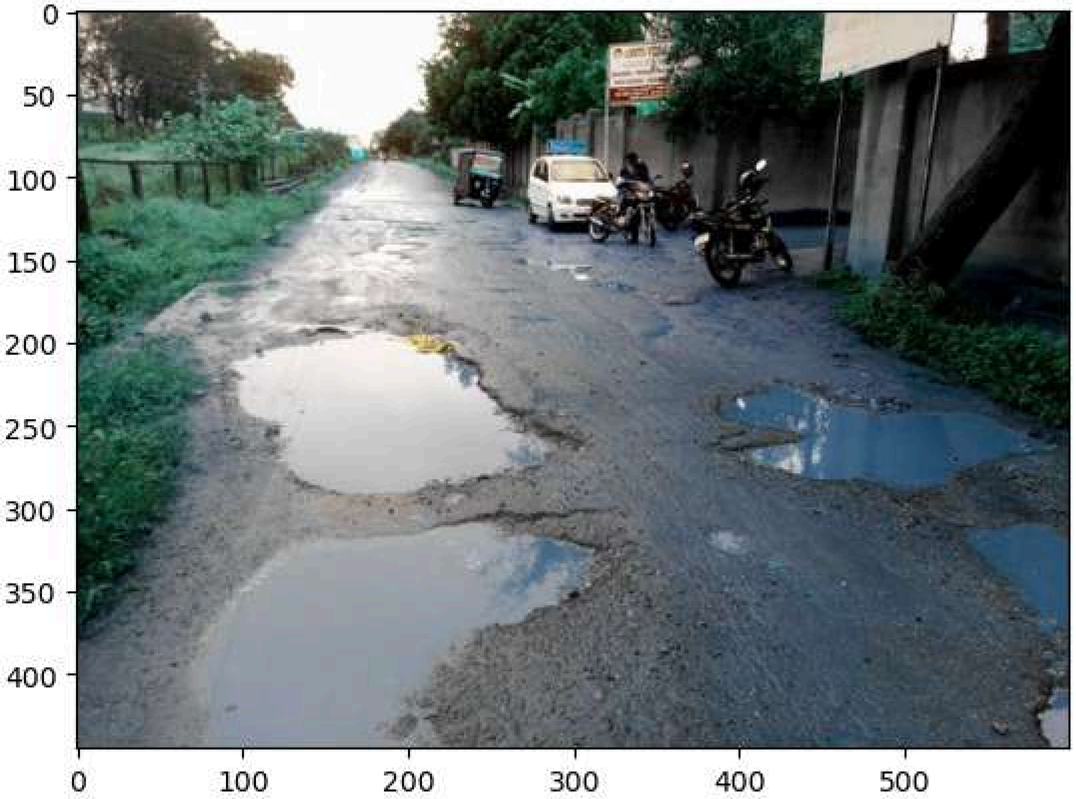
0
50
100
150
200
250
300
350
400
0
100
200
300
400
500

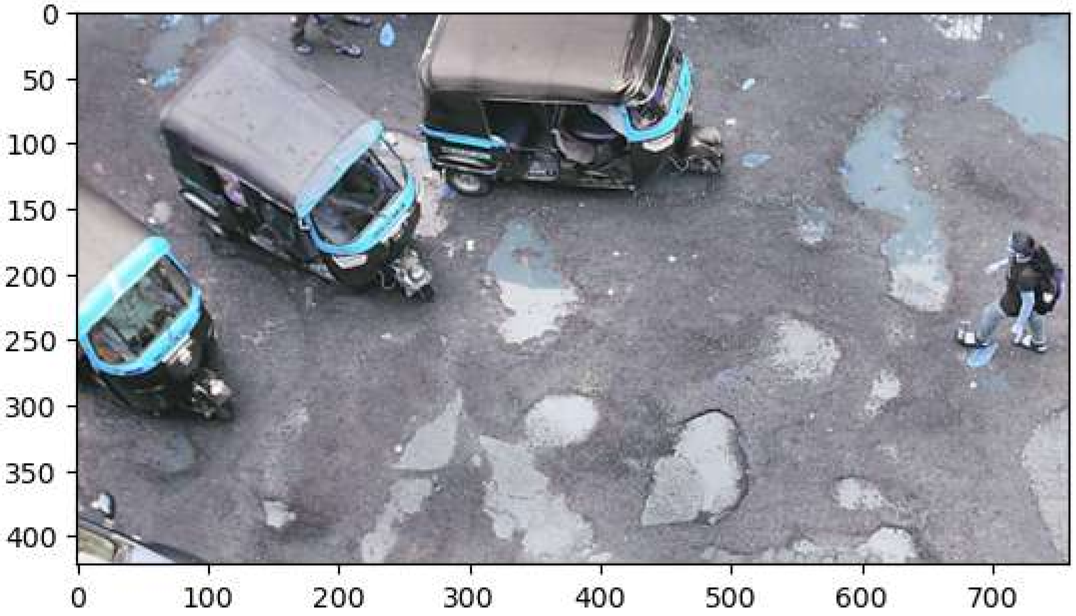
0
50
100
150
200
250
300
350
400
0
100
200
300
400
500
600
700

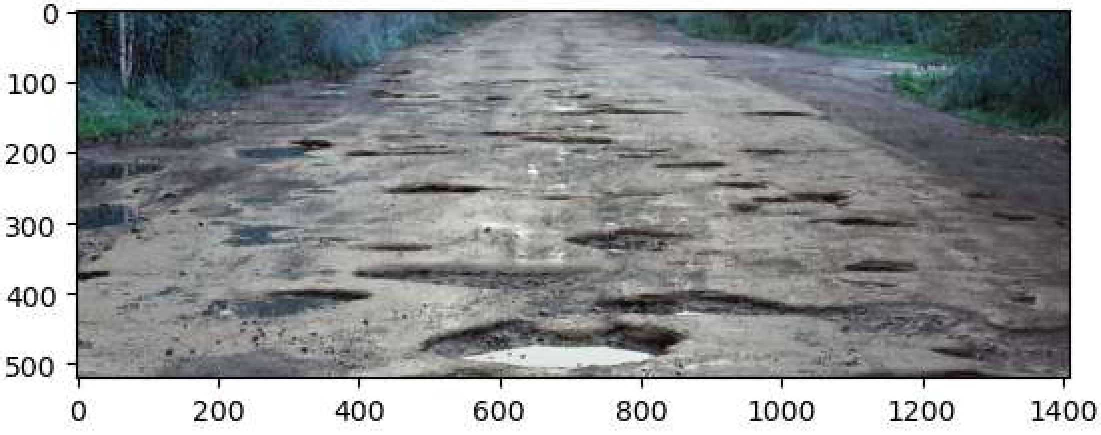
0
100
200
300
400
500
0
200
400
600
800
1000
1200
1400

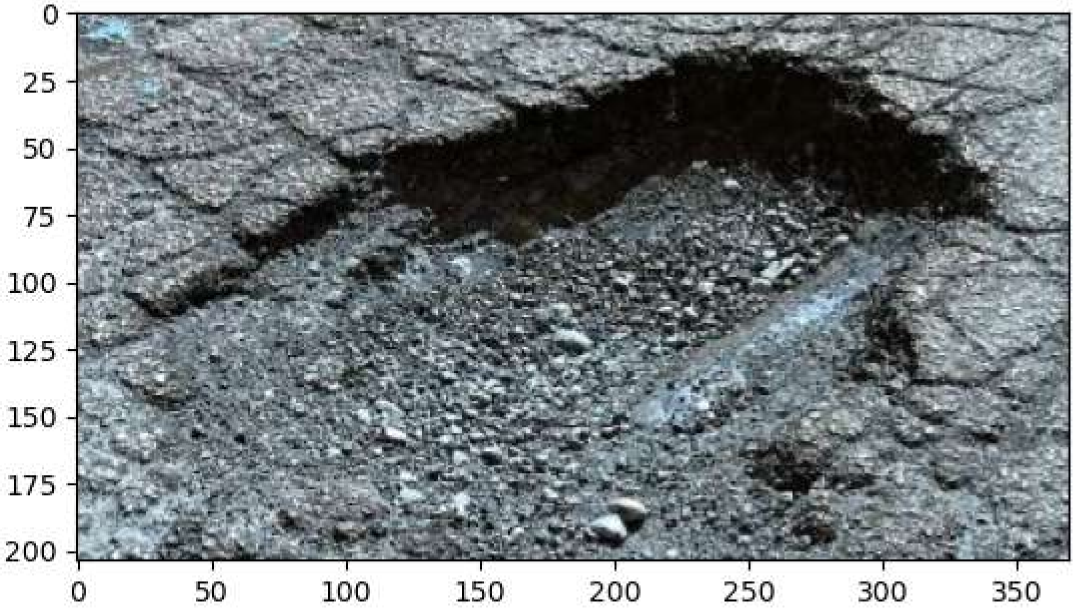

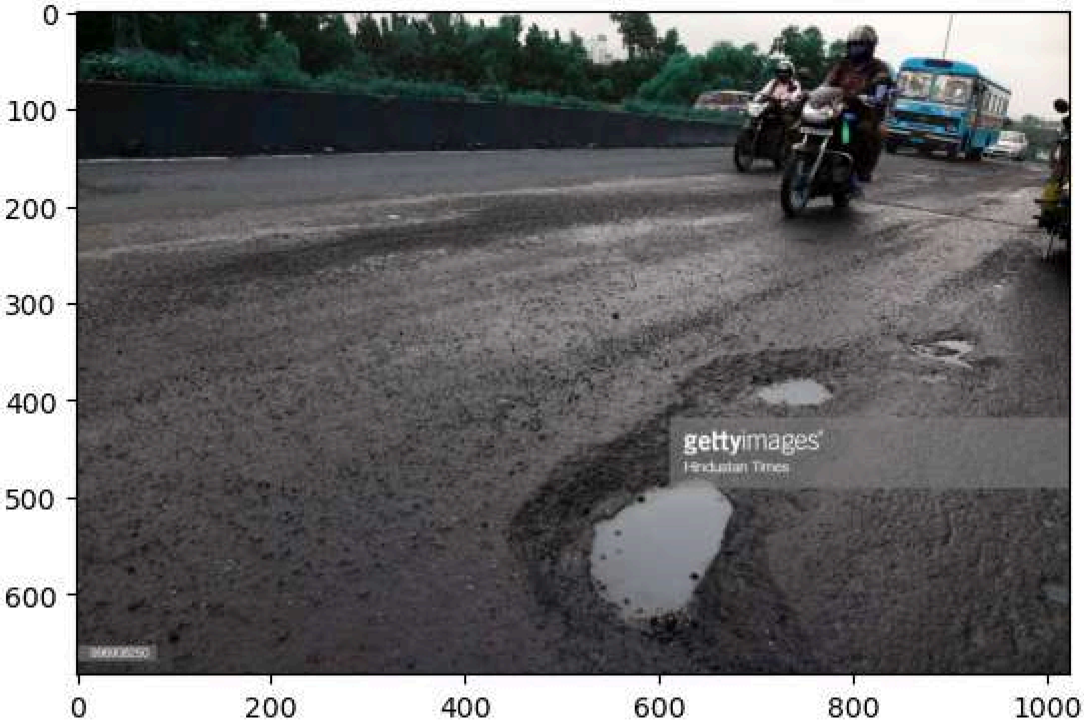

In [ ]:

```
import numpy as np # linear algebra
import os # operating system
```

In [1]:

```
import cv2
import matplotlib.pyplot as plt

def explore_dir(dir_path, count):
    for _, _, filenames in os.walk(dir_path):
        for i in range(count):
            img = cv2.imread(os.path.join(dir_path, filenames[i]))
            plt.imshow(img)
            plt.show()

normal_dir = 'D:/neha1/Normal'
potholes_dir = 'D:/neha1/Pothole'

explore_dir(normal_dir, 90)
explore_dir(potholes_dir, 90)
```

```
---------------------------------------------------------------------------
NameError                                 Traceback (most recent call last)
Cell In[1], line 14
     11 normal_dir = 'D:/neha1/Normal'
     12 potholes_dir = 'D:/neha1/Pothole'
---> 14 explore_dir(normal_dir, 90)
     15 explore_dir(potholes_dir, 90)

Cell In[1], line 5, in explore_dir(dir_path, count)
      4 def explore_dir(dir_path, count):
----> 5     for _, _, filenames in os.walk(dir_path):
      6         for i in range(count):
      7             img = cv2.imread(os.path.join(dir_path, filenames[i]))

NameError: name 'os' is not defined
```

In [ ]:

In [ ]:

In [ ]:

In [ ]:

In [ ]:

In [ ]:

In [ ]:

In [ ]:

In [4]:

```
def count_files(dir_path):
    file_count = 0
    for _, _, filenames in os.walk(dir_path):
        file_count += len(filenames)
    return file_count

print('Normal images: ', count_files(normal_dir))
print('Potholes images: ', count_files(potholes_dir))
```

```
---------------------------------------------------------------------------
NameError                                 Traceback (most recent call last)
Cell In[4], line 7
      4         file_count += len(filenames)
      5     return file_count
----> 7 print('Normal images: ', count_files(normal_dir))
      8 print('Potholes images: ', count_files(potholes_dir))

Cell In[4], line 3, in count_files(dir_path)
      1 def count_files(dir_path):
      2     file_count = 0
----> 3     for _, _, filenames in os.walk(dir_path):
      4         file_count += len(filenames)
      5     return file_count

NameError: name 'os' is not defined
```

In [1]:

```
import cv2
import matplotlib.pyplot as plt
```

```
def explore_dir(dir_path, count):
    for _, _, filenames in os.walk(dir_path):
        for i in range(count):
            img = cv2.imread(os.path.join(dir_path, filenames[i]))
            plt.imshow(img)
            plt.show()

normal_dir = 'D:/neha1/Normal'
potholes_dir = 'D:/neha1/Pothole'

explore_dir(normal_dir, 200)
explore_dir(potholes_dir, 200)
```

```
---------------------------------------------------------------------------
NameError                                 Traceback (most recent call last)
Cell In[1], line 14
     11 normal_dir = 'D:/neha1/Normal'
     12 potholes_dir = 'D:/neha1/Pothole'
---> 14 explore_dir(normal_dir, 200)
     15 explore_dir(potholes_dir, 200)

Cell In[1], line 5, in explore_dir(dir_path, count)
      4 def explore_dir(dir_path, count):
----> 5     for _, _, filenames in os.walk(dir_path):
      6         for i in range(count):
      7             img = cv2.imread(os.path.join(dir_path, filenames[i]))

NameError: name 'os' is not defined
```

In [ ]:
